# Unexpected Novel Relational Links Uncovered by Extensive Developmental Profiling of Nuclear Receptor Expression

**DOI:** 10.1371/journal.pgen.0030188

**Published:** 2007-11-09

**Authors:** Stéphanie Bertrand, Bernard Thisse, Raquel Tavares, Laurent Sachs, Arnaud Chaumot, Pierre-Luc Bardet, Héctor Escrivà, Maryline Duffraisse, Oriane Marchand, Rachid Safi, Christine Thisse, Vincent Laudet

**Affiliations:** 1 Molecular Zoology, Institut de Génomique Fonctionelle de Lyon; UMR 5242 du CNRS, INRA, IFR128 BioSciences Lyon-Gerland, Université de Lyon, UCB; Ecole Normale Supérieure de Lyon, France; 2 Institut de Génétique et de Biologie Moléculaire et Cellulaire (IGBMC), CNRS/INSERM/ULP, BP 10142, 67404 Illkirch, CU de Strasbourg, France; 3 CNRS UMR 5166, Département Régulations, Développement et Diversité Moléculaire, Muséum National d'Histoire Naturelle, CP32, Paris, France; 4 CEMAGREF, Laboratoire d'écotoxicologie, Lyon, France; Stanford University Medical Center, United States of America

## Abstract

Nuclear receptors (NRs) are transcription factors that are implicated in several biological processes such as embryonic development, homeostasis, and metabolic diseases. To study the role of NRs in development, it is critically important to know when and where individual genes are expressed. Although systematic expression studies using reverse transcriptase PCR and/or DNA microarrays have been performed in classical model systems such as *Drosophila* and mouse, no systematic atlas describing NR involvement during embryonic development on a global scale has been assembled. Adopting a systems biology approach, we conducted a systematic analysis of the dynamic spatiotemporal expression of all NR genes as well as their main transcriptional coregulators during zebrafish development (101 genes) using whole-mount in situ hybridization. This extensive dataset establishes overlapping expression patterns among NRs and coregulators, indicating hierarchical transcriptional networks. This complete developmental profiling provides an unprecedented examination of expression of NRs during embryogenesis, uncovering their potential function during central nervous system and retina formation. Moreover, our study reveals that tissue specificity of hormone action is conferred more by the receptors than by their coregulators. Finally, further evolutionary analyses of this global resource led us to propose that neofunctionalization of duplicated genes occurs at the levels of both protein sequence and RNA expression patterns. Altogether, this expression database of NRs provides novel routes for leading investigation into the biological function of each individual NR as well as for the study of their combinatorial regulatory circuitry within the superfamily.

## Author Summary

NRs are key molecules controlling development, metabolism, and reproduction in metazoans. Since NRs are implicated in many human diseases such as cancer, metabolic syndrome, and hormone resistance, they are important pharmaceutical targets and are under intense scrutiny to better understand their biological functions. In the present study, we determined the expression patterns of all NR genes as well as their main transcriptional coregulators during zebrafish development. We used zebrafish because the transparency of its embryo allows us to perform whole-mount in situ hybridization from early development to late organogenesis. This complete developmental profiling offers an unprecedented view of NR expression during embryogenesis, uncovering their potential function during central nervous system and retina formation. We observed that in contrast to NR genes, only a few coregulators exhibit a restricted expression pattern, suggesting that tissue specificity of hormone action is conferred more by the receptors than by their coregulators. Lastly, by evolutionary analysis of expression pattern divergence of duplicated genes, we observed that neofunctionalization occurs at the levels of both protein sequence and mRNA expression patterns. Taken together, our data provide the starting point for functional analysis of an entire gene family during development and call for the study of the intersection between metabolism and development.

## Introduction

Diverse processes such as reproduction, development, metabolism, and cancer are genetically regulated to a large extent by nuclear hormone receptors (NRs), a prominent transcription factor superfamily [1]. Several small lipophilic molecules, including steroids, thyroid hormones, and retinoids, function by binding members of this superfamily. In addition, a significant fraction of NRs (approximately 50% in human) are defined as orphan receptors since the identity of their ligand, if one exists, is unknown [2]. With a few exceptions, such as DAX and SHP in vertebrates, all NRs show a common structural organization with a highly conserved DNA-binding domain, and a less conserved ligand-binding domain. Regardless of their status as orphan or liganded receptors, they interact with hormone response elements in gene promoters or enhancers to regulate transcription [2]. NRs repress or activate the transcription of target genes through varied interactions with numerous transcriptional coregulators, which, together with other transcription factors, mediate chromatin modifications, leading to the repression or activation of target genes [3].

**Figure 1:**
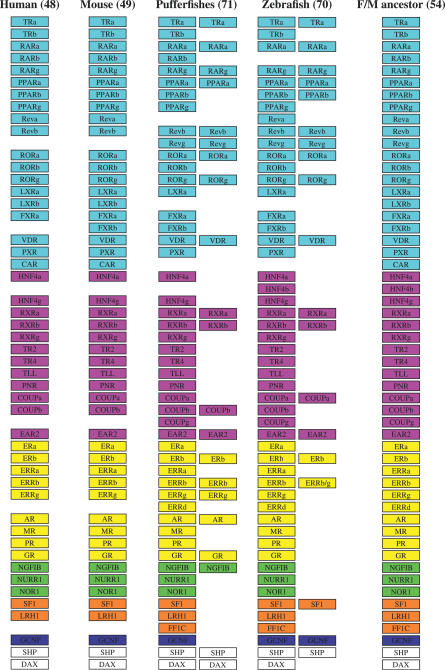
NR Complement in Human, Mouse, Zebrafish, Pufferfish, and the Inferred Complement in the Common Ancestor of Actinopterygian Fish and Mammals (Indicated by “F/M Ancestor”) Each color corresponds to a specific NR subfamily: light blue, purple, yellow, orange, dark blue, and white for subfamilies 1, 2, 3, 4, 5, 6, and 0, respectively.

The conservation of several domains of NRs allows for relatively easy isolation of their sequences and permits efficient phylogenetic reconstruction of the superfamily [4,5]. This is why several global studies of the whole superfamily have been performed in terms of structural genomics [6–8]. Apart from having implications in evolutionary biology, these comparative approaches have provided an important source of information on the function of human NRs. For example, interspecific comparison of amino acid residues of the ligand-binding domain can help identifying key functional residues required for ligand recognition [9–11]. The number of NR genes present in complete genome sequences has been used as a tool to trace gene duplication and gene loss events that have shaped the structure of the superfamily [4]. Indeed, the number of NR genes varies considerably in metazoan genomes: in humans, 48 receptors were found, 49 in mouse, 21 in *Drosophila*, 17 in *Ciona*, 33 in sea urchin, and more than 270 in Caenorhabditis elegans [4,6,7,12,13]. In two species of pufferfish, Takifugu rubripes and Tetraodon nigroviridis, at least 71 NR genes were found, thus highlighting the impact of the ancestral fish-specific genome duplication that took place early in evolution of actinopterygian fish [14,15] (Figure 1).

In addition to this structural and evolutionary information, several resources are now available to provide functional information on NRs (e.g., NURSA, http://www.nursa.org/; NUREBASE, http://www.ens-lyon.fr/LBMC/laudet/nurebase/nurebase.html; and NucleaRDB, http://www.receptors.org/NR/). Several bioinformatic and experimental searches for hormone response elements have led to a better understanding of the transcriptional hierarchies controlled by NRs and their ligands [16–18]. Systematic analysis of NR interactions with themselves and with their coregulators allowed for precise elucidation of each receptor's interactome [19,20]. More recently, systematic expression studies using reverse transcriptase PCR (RT-PCR) and/or DNA microarrays have been performed in classical model systems such as *Drosophila* and mouse [21–24]. However, for studying the implications of NRs in development, it is critically important to know when and where individual genes are expressed. This is why we have established the complete spatiotemporal profiles of the expression of all NR genes during embryonic development using the zebrafish as a model system, because the optical transparancy of its embryo allows studies of gene expression with a cellular resolution using whole-mount in situ hybridization [25].

Other studies have been performed on NR expression during embryonic development in vertebrates, mainly in mouse, rat, chicken, and *Xenopus* [2]. However, most of them are partial and only describe expression by northern blot analysis or by in situ hybridization restricted to one organ or a few developmental stages for a limited number of genes. Moreover, for many NRs, expression during development was only studied regarding their roles in the adult, therefore introducing a bias in the interpretation of the data.

To carry out this large-scale project, we isolated all 70 NR genes in zebrafish plus 31 of their coactivators and corepressors. We analyzed the expression of these 101 genes from gastrula to early larval stages by whole-mount in situ hybridization. This allowed us to detect extensive correlation of expression between NR genes and their coregulators. Our results reinforce the notion that NRs are mainly expressed during organogenesis, with few of them expressed at early developmental stages. Our most unexpected finding is that the large majority of NR genes are expressed during central nervous system (CNS) and retina development, since classically, the primary role NRs was thought to be metabolism control in endodermal derivatives [2]. Finally, evolutionary analysis of the NR genes that were retained following the fish-specific genome duplication, shows that neofunctionalization of these genes occurred at the levels of both protein sequence and RNA expression patterns.

Taken together, our data extend and refresh our vision of NR involvement during vertebrate development, calling for a closer look at metabolic pathways and the control of homeostasis in developmental processes.

## Results

### NR Complement in Zebrafish

Using RT-PCR, we isolated probes corresponding to 70 NR genes from Danio rerio, all of which correspond to a distinct locus in the zebrafish genome, which is publicly available. The assignment of each sequence was done for each NR group by phylogenetic analysis (Figure S1). Figure 1 gives the complete list of the 70 NR genes that we found (see also Table S1). When we compared with the mammalian NR complement, we did not find orthologs of RARβ, LXRβ, or CAR using either RT-PCR or database searches. An ortholog of RARβ was found in the complete pufferfish genomes but was apparently lost in zebrafish. Thus far, neither LXRβ nor CAR has been described in any fish. Because it is always difficult to decide on the absence of a gene in a given genome, especially when the complete genome sequence is not published, we performed additional RT-PCR and PCR experiments on various tissues and/or DNA preparations with several primer pairs for these genes. We nevertheless cannot formally rule out that we artifactually missed a specific duplicate.

It is now clearly established that actinopterygians underwent a complete genome duplication [14,26]. Indeed, compared to mammals, 19 genes are duplicated in zebrafish (TRα, RARα, RARγ, PPARα, PPARβ, Rev-erbβ, Rev-erbγ, RORα, RORγ, VDR, RXRα, RXRβ, COUP-TFα, EAR2, one ERRβ or γ, ERβ, SF-1, GCNF,and SHP). Eighty percent of these duplications are shared with pufferfish. For clarity, we name these duplicates with capital letters after the gene name: PPARα-A and PPARα-B are thus the two duplicates of PPARα. Our phylogenetic analysis also reveals five NR paralogues that have no counterparts in mammals. These genes are Rev-erbγ, COUP-TFγ, ERRδ, FF1C, a member of the FTZ-F1 family, and HNF4β. They were also all found in the pufferfish genomes, while HNF4β is present in Xenopus laevis and in chicken.

Many different coactivators and corepressors of NRs have been described and these molecules exhibit highly variable functions, specificities, and modes of action [2,3,27]. Therefore, in contrast to NR genes, we did not attempt to perform an exhaustive analysis and decided to isolate only the most obvious ones. We have isolated representatives of the four main coregulator complexes (Figure S2), namely, the p160 complex (containing the three SRC/p160 factors, CBP/P300, Cited3, and CARM), the SMCC or Mediator complex (with TRAP220), the SWI/SNF complex (Baf53, Baf60 and BRG1), and the corepressor complex containing NCoR, SMRT, and histone deacetylases. Table S1 contains the list of the 31 probes that we have isolated, along with their Genbank accession numbers. As for NR genes, for each coregulator isolated, a tree was constructed to assign clear orthology and in some cases we noticed the presence of actinopterygian-specific duplicates (Figure S1). However, we cannot exclude that duplicates may exist for some coregulators for which only one copy was detected.

### Global Analysis of NR Expression

We have determined the spatiotemporal expression pattern of the 101 zebrafish genes by whole-mount in situ hybridization at seven different developmental stages that are classically studied [28]. Plates describing individual expression patterns are presented in Figure S3, and have been deposited in the ZFIN database (http://zfin.org) and will be available at the Nurebase Web site.

**Figure 2:**
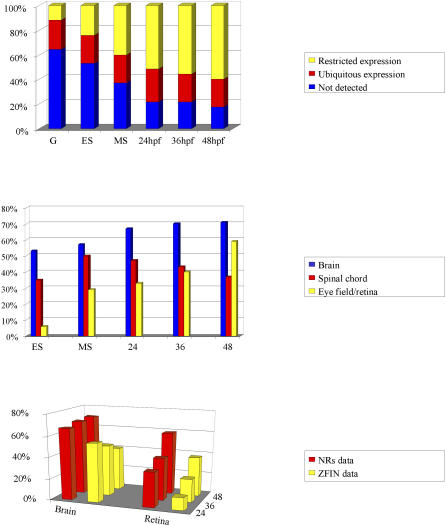
Statistical Analysis of NR Expression Patterns in Zebrafish (A) Proportion (in percent) of NR genes with ubiquitous, restricted, or not-detected expression for each of the studied stages. The proportion of genes with ubiquitous expression during embryonic development is almost constant (approximately 20%), whereas the proportion of genes with a restricted expression pattern increases (from 10% up to 60%). (B) Proportion (in percent) of NR genes with a restricted expression pattern that are expressed in nervous system (brain, spinal cord, and retina) from mid-late somitogenesis (MS) to 48 hpf. At 36 hpf, almost 80% of NR genes with restricted expression patterns are expressed in brain and more than half of them are expressed in the retina at 48hpf. (C) Comparison of the proportion (in percent) of genes expressed in brain and retina from 24 hpf to 48 hpf between NR genes and 1,900 genes, whose expression is described in the ZFIN database. NR genes with a restricted expression pattern show a higher tendency to be expressed in brain and retina.

At a global scale, we can define three different types of expression profiles for NRs during zebrafish embryogenesis: (i) genes not expressed during embryogenesis and larval stages or expressed under the limit of detection of in toto in situ hybridization, (ii) genes expressed ubiquitously, and (iii) genes that exhibit a spatially restricted expression pattern. If we compare the expression profiles of NRs at each developmental stage, we observe that the number of spatially restricted NR expression profiles increases dramatically from gastrula to 48 h post-fertilization (hpf) (from 11% to 60%), whereas the number of ubiquitously expressed genes is almost constant (around 20%; see Figure 2A and Table S3). Therefore, it appears that the vast majority of NR genes are not expressed during early embryogenesis but rather late, i.e., during organogenesis. A similar observation was made in *Ciona*, where only 17% of NR genes are expressed early, whereas 48% were found expressed during later stages [29]. We did not notice any obvious correlation between the phylogenetic position of NR genes, their orphan versus liganded status, and the type of their expression patterns (restricted, ubiquitous, or not expressed).

### Predominant Expression of NR Genes in CNS and Retina

**Figure 3:**
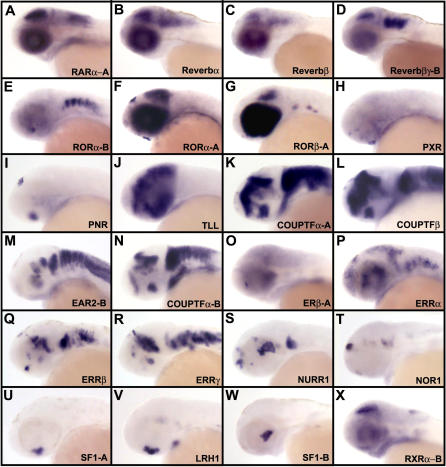
Expression of NR genes in the CNS (A–D) Expression in retina, optic tectum, and hindbrain of RARα-A, Reverbα, Reverbβ, and Reverbγ-B, respectively. RORα-B is expressed in one nucleus in ventral diencephalon and in hindbrain rhombomeres (E), RORα-A in retina, optic tectum, epiphysis, and hypophysis (F), RORβ-A in retina, ventral-posterior part of the optic tectum, and in some neuromasts of the posterior lateral line (G), PXR in small diencephalic and telencephalic nuclei as well as in adenohypophysis (H), PNR in epiphysis, ventral part of retina, and some neurons of posterior diencephalon (I), TLL in diencephalon and mesencephalon with more labeling in ventral diencephalon, anterior tegmentum, and optic tectum (J). COUPTFα-A is expressed in the ventral part of the diencephalon, in forebrain ventricular zone, in tegmentum, and hindbrain (K), COUPTFβ displays a similar expression with additional expression in the dorsal half of the diencephalon (L). EAR2-B expression is restricted to dorsoventral stripes in tegmentum, in hindbrain of rhombencephalon, and in spinal cord (M), COUPTFα-B displays an expression in ventral diencephalon, telencephalon ventricular zone, anterior tegmentum, pretectum, and hindbrain (N). ERβ-A is expressed in a small nucleus in the anterior ventral part of the diencephalon (O), while ERRα is expressed in all brain subdivisions except for the forebrain ventricular zone, tegmentum, and dorsal rhombencephalon (P). ERRβ displays a complex expression with a nucleus in ventral telencephalon, nuclei in diencephalon and tegmentum, and an expression in hindbrain (Q), ERRγ has a very similar expression except for the ventral telencephalic nucleus (R). NURR1 is expressed in part of the telencephalon, in a nucleus in anterior diencephalon, in posterior diencephalon and anterior tegmentum, and in the ventral anterior part of rhombencephalon (S). NOR1 is expressed weakly in ventral telencephalon, tegmentum, and hindbrain and strongly in the habenula (T), SF1-A, LRH1, and SF1-B are expressed strongly in the ventral diencephalon (U–W). RXRα-B is expressed at a low basal level in all brain territories with a much stronger intensity in the ventral part of the optic tectum (X). Embryos are in lateral view, anterior to the left, and are 36 hpf except for (A–D, O, W, and X), where they are at 48 hpf. More extensive anatomical descriptions of these expression patterns are presented in Figure S3 and anatomical details are available at ZFIN (http://zfin.org).

We then analyzed in detail the expression pattern of NR genes that are spatially restricted during embryogenesis. Strikingly, we observed that many of them are expressed in the retina and in the CNS (e.g., spinal cord and/or brain), even if for each receptor, the expression is restricted to a part of these organs (Figure 2B). Figure 3 presents a selection of the expression patterns we detected in the brain, stressing the diversity of expression of NR genes in the CNS. At the mid-somitogenesis (MS) stage, more than 55% of spatially restricted NRs are expressed in the brain, and this proportion increases up to 71% at 48 hpf. The same picture holds for the retina (from 29% at MS stage up to 59% at 48 hpf). In addition, all genes expressed in the retina, except for TRβ, are also expressed in the brain and/or in the anterior spinal cord.

To test whether this high percentage of genes expressed in CNS and retina could be specific to NRs, we analyzed a set of 1,900 genes with spatially restricted expression patterns available in the ZFIN database. We found 40% to 54% of these genes expressed in the CNS between 24 and 48hpf, whereas for NR genes, this percentage rose to 71%. Eleven percent to 37% of genes were expressed in the retina, whereas 30% to 59% NR genes were expressed in the same organ (Figure 2C). Therefore, even if many genes are indeed expressed in CNS and retina, NR genes tend to be expressed more often than expected in these organs.

In contrast, some organs or tissues express very few NR genes in a restricted manner. This is the case for the lens, blood, somites, and heart, even if these organs express the NRs that show a ubiquitous expression pattern. Phenotypic analyses of mouse knockouts, as well as studies on the implication of NRs in human diseases, have suggested a major role for NRs in the control of homeostasis, and specifically in lipid metabolism, including cholesterol and steroid metabolism (see [2] for a review). These processes occur in organs such as liver, intestine, pancreas, and adipose tissue, all of which are endodermal derivatives, as well as in the adrenal gland, which is derived from neural crest cells. Looking at NR expression in these organs, we found, at various stages, VDR-B, EAR2-B, and FF1C expressed in the intestinal bulb, whereas FXRα, ERβ-A, and LRH1 were found in the liver. In addition, three genes, PPARβ-A, PXR and HNF4α, were detected in both organs. Therefore, we are confident that we did not miss expression of NR genes in endodermal tissues before 5 d of development. The case of PXR, which in mammals is restricted to endodermal derivatives, nicely illustrates this point. In zebrafish, we found this gene expressed at 24 hpf in the pituitary and at 36 hpf with a complex pattern in the telencephalon and diencephalon (see Figure S3). At 48 hpf, expression remains in the CNS but is also found at a relatively low level in intestine and liver. This demonstrates the power of whole-mount in situ screens in revealing heretofore unsuspected expression patterns. Recently, two analyses of genes of the NR2E, RAR, and RXR groups also revealed extensive expression in retina and CNS, globally supporting our findings [30,31].

Another well-known target of NRs in mammals is the sexual organs. Sex determination is a complex and late event in zebrafish and sexual organs are not yet differentiated at the stages examined by whole-mount in situ hybridization. We thus cannot discuss the eventual implication of NRs genes in differentiation of sexual organs in this species and this may account for the lack of expression of AR, PR, and ERα that we noticed in our study. However, at the studied stages, primordial germ cells are present in the embryo and migrate along the body axis, but we did not detect specific NR expression (e.g., GCNFs) in these cells.

### Differential Expression of NR Genes in the Retina

**Figure 4:**
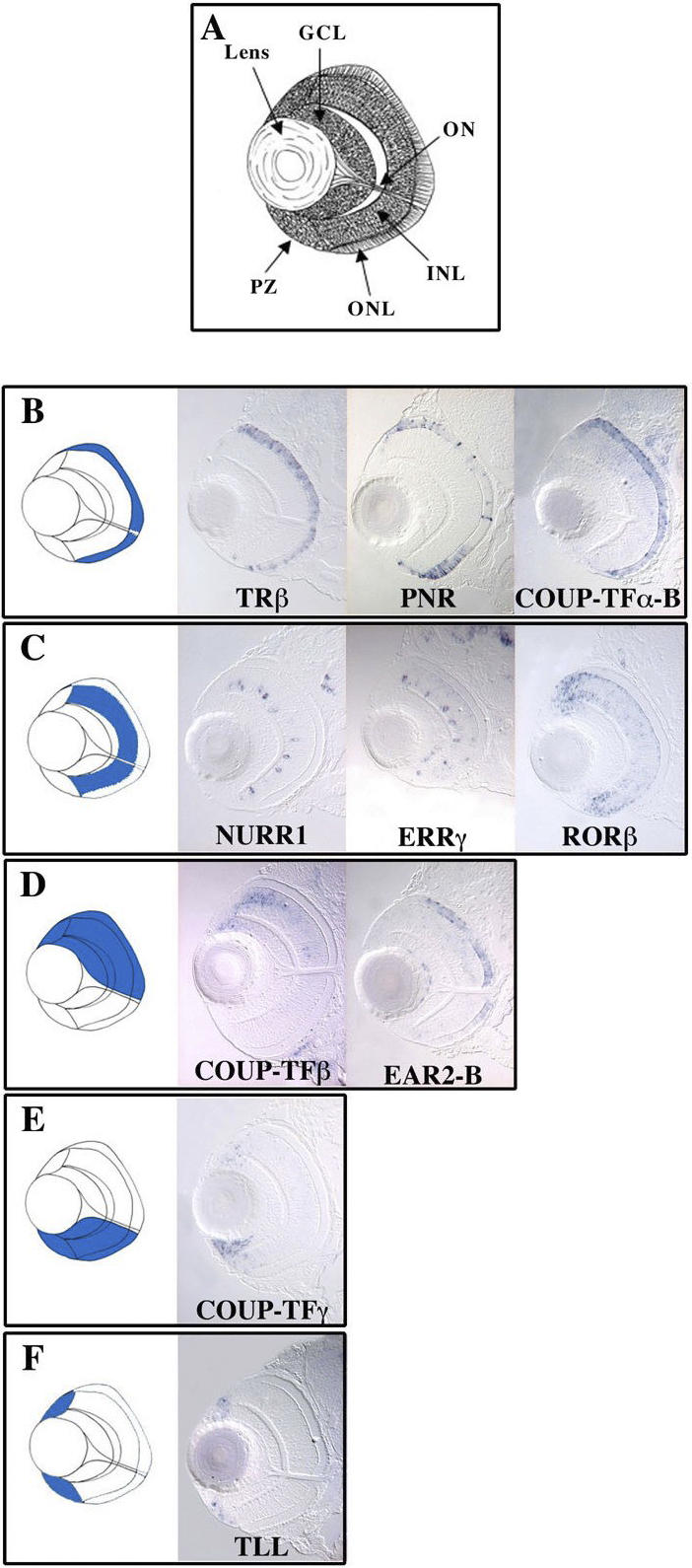
Expression of NR Genes in Retina at 72 hpf (A) Schematic of a zebrafish eye at 72 hpf showing the characteristic multilayered structure. GCL, ganglion cell layer; INL, inner nuclear layer; ONL, outer nuclear layer; ON, optic nerve; PZ, proliferative zone. (B–F; left panels) Schematic showing in blue the various types of expression patterns found for NR genes in retinas at 72 hpf after whole-mount *in situ* hybridization and section. (B) Genes expressed in the ONL. (C) Genes expressed in the INL. (D) Genes expressed in the dorsal part of the retina. (E) Genes showing expression in the ventral part of the retina. (F) Genes showing expression in the proliferative zone of the retina.

Because we found frequent and complex restricted expression in the developing retina, we performed high-resolution analysis at 72 hpf, when the retina is already well differentiated. We then analyzed systematically the expression of the 25 NR genes expressed in the retina at this stage (Figures 4 and S4). At 72 hpf, the retina is divided into three main layers: the outer nuclear layer (ONL) containing cell bodies of photoreceptors, the inner nuclear layer (INL) which contains four classes of interneurons (amacrine, bipolar, horizontal and interplexiform) as well as Müller glia, and finally the ganglion cell layer (GCL), which contains ganglion cells.

By examining the retinal expression of these 25 genes, we observed a large diversity of patterns (Figure 4). TRβ, PNR, COUP-TFα-B are only expressed in the ONL (Figure 4B). RORβ, NURR1 and ERRγ are found only in the INL (Figure 4C), whereas no NRs are expressed only in the GCL. COUP-TFβ and EAR2-B are expressed in an asymmetric manner in the dorsal part of the INL and the ONL, respectively (Figure 4D). COUP-TFγ shows expression in the ventral part of these two layers (Figure 4E). TLL expression is not restricted to a specific layer of the retina, but is associated with cell proliferation (Figure 4F) [31]. Finally, the remaining 15 NRs are expressed in more than one layer and often ubiquitously. All these data highlight a diversity of NR gene expression in the retina suggesting that these genes may be implicated in a wide variety of processes.

The fact that the retina expresses a large proportion of the members of the NR superfamily has not been noticed in other vertebrates. This may be due to the fact that no global spatiotemporal expression pattern study of this superfamily has been performed with whole-mount in situ hybridization in mammals or *Xenopus*, or that there are specific differences between mammals and zebrafish concerning NR gene expression in the retina. We thus specifically verified if the genes that are expressed in the zebrafish retina are implicated in retinal development in other vertebrates. By an extensive survey of the literature, we found that among the ten genes that express in specific cell layers or cell types in the retina, four (TRβ, PNR, RORβ, and TLL) are known to be important for retina development in mammals. Indeed, retinal phenotypes in knockout mice and mutations in human diseases have been associated with these genes [33–35]. In addition, expression in the retina has been observed in other vertebrates for five more genes: NURR1 [36–38], ERRγ [39], COUP-TFγ, COUP-TFβ, and EAR2 [40–42]. Finally only one of these genes, COUP-TFα-B is expressed in zebrafish retina, while its mammalian counterpart is not [43]. We noticed that some genes ubiquitously expressed in the zebrafish retina (Rev-erb and ROR) have also been described as expressed in the mammalian retina [44,45]. Taken together, our data strongly suggest that some receptors have a conserved role in vertebrate retinal development and that the importance of this organ for the study of NR biological functions has been largely overlooked. This nicely illustrates the power of large expression screens, such as the one we performed here, in unraveling potential functions of NRs in specific organs.

### Expression of Coregulators

In striking contrast with NR genes, most of the CoA/CoR we studied show ubiquitous expression (CBP-A, CBP-B, P300-A, P300-B, BRG1, PCAF, NCoA6, Baf 53, SRC1, SRC2, NCoA4, Baf 60, N-CoR, Alien, Sin3A, HDAC1, HDAC3, and TIF1α) or do not display embryonic expression that could be detected by whole-mount in situ hybridization (TRAP220, MYST-HAT2, TRIP13, and ARA54) (see Figure S3). In fact, only 30% of the coregulators (SRC3, RIP140-A, RIP140-B, PGC1, TRIP7, TIF1γ, Cited3, CARM1, SMRT, and HDAC4) show a spatially restricted expression pattern, suggesting that tissue specificity of hormone action is conferred more by the receptors than by their coregulators. Apart from TIF1γ, which is expressed in ventral hematopoietic mesoderm [46], all other spatially restricted coregulator genes are expressed in the CNS, stressing again the importance of NR signaling in this organ.

Among the ten spatially restricted coregulators, we found expression territories that do not correlate with expression of spatially restricted NRs. For example, HDAC4 is expressed in trigeminal ganglia and PGC1 and RIP140-A are expressed in several cranial ganglia, whereas RIP140-B is specifically expressed in the habenula. Some of the coregulators, namely HDAC4, Cited3, CARM1, SMRT, and RIP140-B, also show restricted expression in the retina. It should be noted that TRIP7 is expressed in the lens, where only EAR2-B is expressed in a restricted manner. We also observe expression of SMRT at 5 dpf in the thymus, where we did not find any expression of spatially restricted NR genes. These data support in vivo the notion that coregulators mediate the action of transcription factors other than NRs.

### Identification of Overlapping Expression Patterns

Our systematic analysis revealed extensive similarities of expression patterns between NRs and their coregulators. For example, in the p160 family, which contains three members (SRC1, SCR2, and SRC3), SRC3 shows a restricted expression that is reminiscent of that of RXRs and RARs (Figure S5) [30]. This gene is mainly expressed in anterior spinal cord, posterior branchial arches, and tail bud, suggesting possible RAR/RXR interactions with SRC3 in these territories.

**Figure 5:**
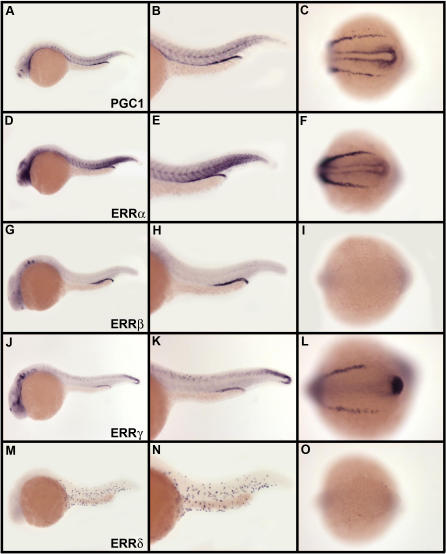
Overlapping Domains of Expression between PGC1 and ERRs (A–O) Expression of PGC1 in slow muscle fibers, posterior pronephric ducts, mucous cells, epiphysis, and part of the telencephalon and diencephalon (A–C) overlaps extensively with the expression of ERRs. ERRα is coexpressed with PGC1 in slow muscle fibers, posterior pronephric ducts, telencephalon, and mucous cells (D–F). ERRβ is coexpressed with PGC1 in epiphysis and posterior pronephric ducts (G–I), ERRγ in epiphysis, part of the tegmentum, and posterior pronephric ducts and ERRδ in mucous cells. Embryos are at 24 hpf in lateral view anterior on the left except for (C, F, I, L, and O), which are shown at the 14-somite stage. Posterior part of the embryo is presented in dorsal view, anterior to the left. More extensive anatomical descriptions of these expression patterns are presented in Figure S3 and anatomical details are available at ZFIN (http://zfin.org).

PGC1 is another coactivator showing a striking correlation of expression with certain NR genes. This gene was identified by its direct interaction with PPARγ and was later shown to be important for other NRs, including ERRα, TRs, and RXRs (for a review, see [47,48]). In zebrafish, PGC1 shows a very specific expression pattern in adaxial cells, pronephric ducts, and mucous cells during somitogenesis, and in the epiphysis, olfactory bulb, diencephalic nuclei, hindbrain, heart, pronephric ducts, mucous cells, and slow muscle fibers at 24 hpf. Overall, this expression pattern overlaps extensively with those of the ERR genes (Figure 5). During somitogenesis stages, ERRα is expressed in adaxial cells, pronephric ducts, and mucous cells, ERRβ and ERRγ are expressed in pronephric ducts, while ERRβ/γ is expressed in mucous cells. At 24 hpf, PGC1 expression overlaps with that of ERRα in pronephric ducts, in slow muscle fibers, of ERRβ in pronephric ducts, epiphysis and in diencephalic nucleus, of ERRγ in epiphysis and diencephalic nucleus and of ERRβ/γ in the mucous cells. In the mouse, no complete embryonic expression pattern of PGC1 has been reported, but complex expression in adult brain was observed in rat [49]. In mouse, PGC1 is preferentially expressed in slow muscle fibers, a situation that we also found in zebrafish [50]. This is consistent with the notion of specific needs for PGC1 in mediating transcriptional activity of ERRs during embryogenesis and with reports highlighting the functional importance of the PGC1/ERR hub [51].

In addition, we identified two other groups of genes (Rev-erb/ROR and COUP-TF) sharing extensive similarity of expression suggestive of common functions. Nine of the ten Rev-erb/ROR genes are expressed in retina, optic tectum, hindbrain, and/or epiphysis. We also found that the expression patterns of three coregulators, RIP140-B, SMRT, and HDAC4, largely overlap with those of Rev-erb/ROR. These expression data strongly suggest that in vivo these genes are regulated in a similar way. In accordance with this notion, we recently observed that Rev-erbα expression is under the control of Rev-erbs and RORs both in vitro and in vivo [52,53]. These expression patterns are fully consistent with the important role played by these genes in the generation and control of circadian rhythm [54–56]. Interestingly, SMRT has been shown to interact with Rev-erbs in mammalian cells [57]. Taken together, these observations suggest that the roles played by RIP140-B, SMRT, and HDAC4 in circadian rhythm should be more carefully examined in the future. Similarly, among the six members of the COUP-TF group, COUP-TFα-A, COUP-TFα-B, COUP-TFβ, COUP-TFγ, and EAR2-B are expressed in a similar and complex expression pattern in the CNS (Figure S6). Once again, this is congruent with the known role of these genes in nervous system development in zebrafish and more generally in vertebrates.

### Hierarchical Clustering of NR and Coregulator Expression

**Figure 6:**
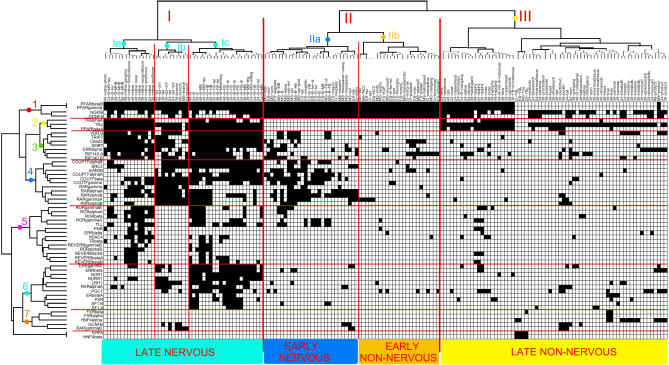
Clustering of NR and Coregulator Expression Patterns during Zebrafish Development A hierarchical clustering procedure was performed to compare the expression profiles of regionally expressed NR genes (Table S2) and the patterns of anatomical structures. The correspondence between the resulting classifications reveals the existence of clusters of genes with hierarchically discriminated expression in time and space during development: early versus late (II/I-III), nervous versus non-nervous (I/III or IIa/IIb), and optical versus spinal versus brain (Ia/Ib/Ic). Abbreviations used for anatomical structures are defined in Table S5.

We performed hierarchical clustering of regionalized NR and coregulator genes and the anatomical structures expressing them using a binary matrix that quantifies expression pattern divergence between genes (Tables S2 and S5; Figure 6). This clustering analysis revealed the existence of a higher-order network relating NR genes, their coregulators, and development according to space and time. The anatomical structures expressing NRs and coregulators reveal a clear organization into three clusters (Figure 6): expression in nervous system at late stages (I), early embryonic expression (II), and late expression in non-nervous system structures (III). Cluster I can be further subdivided: retina and optic tectum (Ia), spinal cord (Ib), and brain structures (Ic). Similarly, cluster II can be divided into an early nervous system (IIa) and an early non-nervous system organs (IIb) subcluster. These results suggest that during development, NR genes and their coregulators can be categorized depending on their timing of expression (early/late) and their expression in nervous or non-nervous tissues.

NR and coregulator genes are split into seven clusters (shown on the vertical axis of Figure 6) that follow the previously discussed organ clustering. The genes that we defined above as coexpressed at several developmental stages are clustered within this hierarchy. SRC3 is found in cluster 4 with RARα-A, RARα-B, RARγ-A, RXRα-B, and RXRγ, since they are expressed early (organ subcluster IIa) and late (organ subcluster Ib) in the spinal cord, a situation illustrated in Figure S5. Similarly, PGC1 belongs to cluster 6 as ERRβ and ERRγ. Several members of the COUP-TF family (COUP-TFα-A, COUPTFα-B, COUP-TFβ, COUP-TFγ, and EAR2B) are grouped in clusters 3 and 8, and the ten Rev-erb and ROR genes are found together in cluster 5, since they are expressed late in the retina and in the brain. Furthermore, these genes are never expressed in the spinal cord, a situation explaining their inclusion in cluster 5.

Therefore, this clustering reveals an underlying hierarchy of NR and coregulator genes and suggests that several transcriptional networks are differentially deployed in a spatiotemporal manner during zebrafish development.

### Evolution of Expression and Function of Duplicated Genes

Our expression dataset gives us the opportunity to analyze the evolution of NR gene expression after duplication. We found in zebrafish 19 pairs of genes specifically duplicated in actinopterygians that account for the increased number of NR genes when compared to tetrapods.

According to the Duplication–Degeneration–Complementation (DDC) model [58], duplicated genes have three main fates: in the majority of cases, one of the copies is lost (64% for zebrafish NR genes), in some cases both duplicated genes are subfunctionalized (i.e., they share the function of their nonduplicated ancestor), and in other cases one of the copies undergoes neofunctionalization (i.e., it acquires a new function), while the other retains the function of the ancestor gene. Sub- or neofunctionalization can occur at the level of the expression patterns of the duplicated genes or at the level of their protein coding sequence.

Taking into account that we have no expression data from a basal actinopterygian fish that was not subjected to the genome duplication, expression divergence after duplication can only be inferred by comparison with other vertebrates. Of the 19 duplicated couples that we have studied, we found four cases indicative of neofunctionalization at the level of their expression patterns (RARγ, RORα, RORγ, and GCNF). GCNF provides a clear example of such a case: GCNF-A has an expression pattern that is reminiscent of *Xenopus* and mouse GCNF [59,60], whereas GCNF-B expression is very divergent, with expression observed in head, lateral line neuromasts, and branchial arches. Therefore, it seems that GCNF-A has kept the ancestral expression pattern, whereas GCNF-B has acquired a new one.

The acquisition of a new function can be achieved by fixing advantageous mutations within one of the duplicated genes. The neofunctionalized gene will then evolve under positive selection, significantly faster than the other gene in the pair, which will retain the ancestral role and thus evolve under purifying selection (elimination of deleterious mutations). Asymmetric evolution between gene duplicates may thus be interpreted as a sign of neofunctionalization [61,62]. We compared the protein sequences of the 19 NR gene pairs to the protein sequence of a nonduplicated outgroup (Homo sapiens) and found that the ratios of the evolution rates of the duplicated proteins varied from 1.01 (i.e., similar rates) to 6.1. Because the outgroup is very distant, only strong differences in the evolution rate can be detected and evaluated as statistically significant, making our results conservative. We found a significant acceleration of the protein evolution rate (i.e., a ratio significantly different from 1), relative to the nonduplicated sequence of the outgroup, for eight out of the 19 gene pairs (*p*-values < 0.01 in seven out of the eight cases and a *p*-value = 0.03 in the remaining one). An alternative explanation for the asymmetry in the evolution rates would be the genomic context, as proposed by Zhang and Kishino [63,64]. When two copies have different recombination rates, the copy in the low recombination context accumulates deleterious substitutions because of Hill–Robertson effects (degeneration) and thus will evolve faster than the copy in the high recombination context. We have controlled for this effect by estimating, when possible, the recombination rates of the two genes in each pair (Table S4). The recombination rates were estimated by comparing genetic and physical maps of the zebrafish genome (A. Popa, personal communication). In three out of the eight cases of asymmetrical evolution rates between duplicates, this estimation was not possible at least for one of the genes. Out of the five remaining pairs, only one presented a difference in the recombination rates of the duplicates compatible with the asymmetry in their evolution rates (SHP-A/SHP-B), which suggests that the vast majority of the asymmetrically evolving pairs truly evolved through the neofunctionalization model.

We then looked further into this asymmetric evolution of the duplicates by evaluating their expression pattern divergence. Doing this in a quantitative manner allowed us to investigate if there was any correlation between sequence evolution and the evolution of the expression patterns after duplication. The divergence of the expression patterns of the duplicates varied from 0 (same expression pattern found for both genes, e.g., RXRβ, an almost ubiquitously expressed pair detected in 162 out of 165 organs considered in the analysis or PPARα, a nondetected pair) to 1 (almost completely different expression patterns of the two genes; e.g., SHP-A is detected in only four of the 165 organs and SHP-B is not detected, see Table S2). We computed the sequence divergence between duplicates by calculating the ratio between nonsynonymous to synonymous substitutions (Ka/Ks) between the coding sequences. The Ka/Ks ratio can only be calculated for 17 of the 19 pairs of genes because in two cases (SHP and RORγ) the Ks was saturated. Because all the gene duplicates are from the same duplication event (fish-specific genome duplication), differences in Ks values reflect different mutation rates within the genome. By dividing Ka by Ks we corrected for the influence of these mutation rate differences in the evolution of the coding sequence.

**Figure 7:**
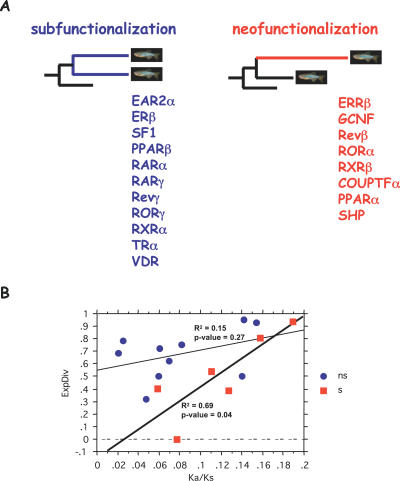
Relative Rates of Protein Evolution, Coding Sequence Divergence, and Expression Pattern Divergence of the Fish-Specific NR Duplicates (A) Phylogenetic view of the relative evolutionary rates of the zebrafish duplicates. On the left side, in blue, NR pairs with similar evolution rates (ratio not significantly different from 1). On the right side, in red, NR pairs where one of the duplicates evolved significantly faster than the other, which suggests neofunctionalization. (B) Relation between expression pattern divergence (calculated as explained in the Materials and Methods section) and coding sequence divergence (Ka/Ks ratio) for the pairs with similar evolution rates (blue circles, ns, the same as in (A) except for RORγ, for which Ks is too saturated to be calculated) and for the pairs with an acceleration of the evolutionary rate of one duplicate (red squares, s, the same as in (A) except for PPARα, a nonexpressed pair, and SHP, for which Ks is too saturated to be calculated). s, significant protein sequence acceleration; ns, nonsignificant difference in protein evolution rates (calculated as explained in Materials and Methods). Regression lines are plotted and Pearson correlation coefficients and *p*-values are indicated. A positive significant correlation is observed for the pairs with a putative “neofunctionalized” duplicate. No significant correlation is detected for the pairs with similar evolution rates of the duplicates.

Strikingly, we observed a significant positive correlation (Pearson correlation factor *R* ^2^ = 0.69 and *p*-value = 0.04) between the expression divergence and the sequence divergence of the duplicates belonging to the pairs (six) where a neofunctionalization is suggested by the asymmetrical evolutionary rates of the proteins (Figure 7B). This means that the divergence of the coding sequence was accompanied by a divergence of the regulatory sequences. No significant correlation between the expression divergence and the sequence divergence was found for the pairs (11) with similar evolutionary rates (a positive but nonsignificant correlation may be observed in Figure 7B).

Taken together, our results show that for duplicated NR genes, neofunctionalization occurred in almost half of the cases, both at the protein and RNA expression patterns.

## Discussion

### Extensive Spatiotemporal Analysis of NR Gene Expression

Several systematic analyses of the NR superfamily at the gene expression level have recently been reported. Sullivan and Thummel [23] have conducted a northern blot analysis of all 21 Drosophila melanogaster NRs from egg to adulthood. A systematic quantitative PCR analysis of expression of 49 NR genes in 39 adult tissues and at several circadian times has been reported in the mouse [21,22]. These studies revealed NR gene coordinated transcriptional programs in developmental and physiological pathways. Analyzing transcript expression at the tissue level with quantitative PCR or northern blots has the advantage of providing a quantitative measure of transcript abundance. Coupled with hierarchical clustering of the data, this allowed the division of the NR regulatory network in the mouse into two main processes: reproduction, development, and growth on the one hand, and nutrient uptake, metabolism, and excretion on the other. Our analysis of embryonic and larval expression patterns, studied by whole-mount in situ hybridization, allows a direct visualization of the spatiotemporal dynamics of the NR superfamily during development. Our study thus nicely complements these previous global analyses by providing, with unprecedented details, a complete dataset of the embryonic territories where NR-mediated regulation is likely to be deployed.

Our data also allow the definition at the global scale of groups of genes expressed in similar locations at several developmental stages and thus highlight the potential transcriptional hierarchies of NRs and coregulators that occur during development. Clustering of the tissues expressing NR and coregulator genes into three main groups according to developmental timing and nature (neural/nonneural) of the tissue supports the notion that NR regulation is used differently during embryonic development. There is no extensive overlap between the seven clusters we defined and those found by Bookhout and colleagues [21]. This suggests that the underlying logic of NR deployment during embryonic development in zebrafish and in the adult mouse is different. Nevertheless, one should keep in mind that the two datasets are different (qualitative versus quantitative data and embryonic versus adult stages) and are thus difficult to compare. The detection of groups of coexpressed genes suggests that some crossregulation might occur between NR genes and/or their coregulators. The ERR-PGC1 and RAR-RXR-SRC3 groups provide good examples of these potential hubs. Future comparison of the expression patterns reported here with those issued from large-scale gene expression analyses will undoubtedly provide relevant information on NR-regulated networks that control embryonic development.

### Implication for Human Diseases

Our exhaustive expression screen reveals that many NRs known to be tightly linked to the control of metabolism in adults are expressed during embryogenesis (e.g., PXR, HNF4α, RXRs, COUP-TFs, and ERRs as well as several coactivators such as PGC1, CITED3, and RIP140).

It is important to stress that most of the expression patterns we describe here are conserved in vertebrates. Given that the methods used to determine expression during development differ from one model organism to another (e.g., tissue sections in mouse, whole-mount in situ in zebrafish, and *Xenopus*), and that only a minority of these NR genes have been studied in several organisms, an exhaustive global comparative analysis of the expression patterns is not yet feasible. Nevertheless, of 26 genes for which data are available, we found 22 cases of complete (TRα-A, TRα-B, PPARβ-A, PPARβ-B, VDR, HNF4α, RXRβ-A, RXRβ-B, TLL, NURR1, SF1-A, SF1-B, LRH1, and GCNF-A) or partial (TRβ, RARα-A, RARα-B, RARγ-A, RARγ-B, RXRα-A, RXRα-B, and COUP-TFβ) conservation of expression, whereas in only four cases (PPARα-A, PPARα-B, PXR, and RXRγ) we found very different expression patterns between zebrafish and other vertebrates. Therefore, we are confident that most of the data we generated will be transferable to mammals and will thus be relevant for the study of human diseases.

Both epidemiological and clinical evidence suggests that prenatal factors play a role in the origin of the metabolic syndrome and its components: hypertension, insulin resistance, obesity, and dyslipidemia (reviewed in [65]). Experimental studies demonstrate that an adverse embryonic or fetal environment can induce structural and functional abnormalities in pancreatic islet cells and can lead to permanent changes in insulin sensitivity [66]. Thus, any developmental perturbation that would affect NR expression and/or the production of NR ligands may be transferred to the NR gene regulatory hierarchy and may impact embryonic development and later on adult physiology and metabolism. Indeed, it is easy to induce insulin resistance and symptoms of the metabolic syndrome by manipulating maternal nutrition (an event that could easily affect NR ligand production) or by exposing the mother to synthetic glucocorticoids [67–69]. Therefore, relating the embryonic expression of NRs, including classical pharmacological targets like TR, RAR, RXR, and PXR, to specific developmental processes will help to better understand the mechanisms of the development of metabolic syndrome. Our data provide a unique basis from which to begin such an analysis.

Our expression analysis can also be used to identify roles of certain NR or coregulator genes in specific human diseases. For example, since an unexpected number of them are expressed in retina, it could be fruitful to search for their implication in the development of retinal diseases. There are still a large number of mapped but unidentified Mendelian human retinal diseases, some of which match to the chromosomal location of the NR genes, which we found expressed in the retina. For example, we found both RXRα and Rev-erbα in the retina and both have a chromosomal location in humans (17q) that corresponds to the one detected for a specific retina disease, CORD4 (Cone Rod Dystrophy 4) [70].

In sum, this expression screen, performed on a species that resembles humans on the level of organization and physiology and on a protein superfamily that can easily be targeted by drugs, will provide important new information for the identification of interesting targets for drug discovery.

### Analysis of NR Gene Duplication

The importance of neofunctionalization following gene duplication has been continuously discussed in the literature since Ohno proposed that it was the main mechanism allowing phenotypic diversity [71]. There is no doubt now that subfunctionalization plays an equally or even more important role in the functional evolution of gene pairs [58,72]. In contrast, the relative contribution of both mechanisms for functional diversification between gene duplicates is still an open question. Different factors must be taken into account when analyzing gene evolution after duplication, including population characteristics of the species studied [73]. Asymmetric evolutionary rates of duplicates, which may be interpreted as a sign of neofunctionalization [61–64], have been shown to affect 10% to 56% of duplicated genes analyzed in various species from yeast to fish [62]. In teleost fish, differences in evolution rates were found in 37% of the duplicated genes analyzed [74,75]. Here, our analysis revealed that 42% of the 19 NR gene pairs analyzed evolved at different rates (when compared with an orthologous single copy outgroup). Furthermore, the retention of gene duplicates among the NR family (36%) is also higher than the one estimated for the whole genome after the fish whole-genome duplication (15% [74]). This is consistent with a higher gene retention after duplication and the presence of neofunctionalization, both of which have been reported in regulatory/development-implicated gene families [74,76–78] (e.g., NRs) compared with other functional classes of genes.

Finally, we also observed a significant positive correlation between coding sequence divergence and expression pattern divergence for the asymmetrical evolving gene pairs. Coupled evolution between coding and regulatory sequences was previously found for single-copy genes, between orthologs of D. melanogaster and D. yakuba [79] and of C. elegans and C. briggsae [80]. In our case, this parallel evolution between coding and regulatory sequences suggests that neofunctionalization affected both the protein function and the expression pattern of the gene. For instance, the evolution rate of GCNF-B is more than two times that of GCNF-A, suggesting that GCNF-B evolved under positive selection, thus acquiring a new function. This is consistent with the divergence of GCNF-B expression patterns suggestive of neofunctionalization: as is the case for the protein sequence, it seems that GCNF-A has kept the ancestral expression pattern, whereas GCNF-B has acquired a new one. It can be hypothesized that following expression divergence of a pair of duplicated genes, the gene that is expressed in novel embryonic territories will accumulate mutations in its coding region more rapidly, because the cognate protein will be exposed to a novel set of interaction partners.

### Metabolism and Development

One of the striking results of our screen is the widespread expression of NR genes in the nervous system: at 36 hpf, 70% of the spatially restricted NRs are expressed in the CNS, whereas 40% of them are expressed in the retina. This represents an underestimation, because ubiquitously expressed NR genes may also play an important role in these organs. Indeed, the expression of the zebrafish HDAC1 gene is widespread in the embryo at all stages of development, whereas this gene plays an important role in the anterior CNS by maintaining neurogenesis [81]. The developmental role played by these genes is perhaps not connected to their adult function in regulating metabolism, but it has to be emphasized that many other observations focus on an unanticipated link between the control of metabolism and nervous system development. In fact, several large-scale expression screens have revealed expression of metabolic enzymes, cholesterol and fatty acid transport proteins, and hormonal receptors in embryos, even during early embryogenesis. In zebrafish, the brain-type fatty acid binding proteins FABP7a and FABP7b, which intracellularly bind to docosahexaneoic acid (DHA), an RXR ligand [82], are distributed in the early developing CNS, retina, pharynx, and swim bladder [83]. Similarly, a fatty acid hydroxylase (FA2H) is expressed in enveloping layer, pronephric ducts, nose, pharynx, liver, and gut during embryonic development [84]. In a recent genome-scale analysis of genes expressed during mouse retina development, prominent expression of metabolic enzymes has been observed in specific cell types, such as the Müller glia [40]. The reasons for such a widespread spatiotemporal control of metabolic genes may be linked to a variable metabolic demand of developing organs or cell compartments related to differential proliferation or differentiation. Alternatively, metabolic proteins could play a specific developmental role. In the case of NR genes, we have at present no specific indication that, for example, the restricted expression of PXR in specific areas of the zebrafish CNS is linked to its detoxification function in adult liver. Another possibility is that metabolic enzymes may be implicated in the production or delivery of signaling molecules. This is of course the case for the CYP26, retinaldehyde dehydrogenases, CRBP, and CRABP, the molecules implicated in retinoid metabolism and transport in vertebrate embryos. Clearly, the evidence that continues to accumulate from various experimental model systems suggests that metabolism should no longer be disconnected from the study of embryonic development.

## Materials and Methods

### Isolation of NR and coregulator partial cDNAs.

Given the unknown expression patterns of most of NR genes in zebrafish, we used total RNA extracted from various adult tissues (muscle, gills, liver, etc.) as well as from embryos at different developmental stages. RNA was extracted from frozen tissues using TRIZOL reagent (Life Technologies). The RNA samples were treated with RQ1 deoxyribonuclease, extracted using phenol/chloroform/isoamylic alcohol (25:24:1) and chloroform/isoamylic alcohol (24:1), and finally precipitated with ethanol.

Degenerate or specific primers were designed using an alignment of all published nucleotide sequences for homologs from other vertebrate species according to previously described methods [85] or using available sequences. Many of the primers are degenerate and were used in a touchdown PCR assay [85]. PCR products were cloned into the PCR2.1-TOPO vector (Invitrogen) and subcloned in pBSK+ or pBKS+ to allow synthesis of sense and antisense probes. A list of studied genes and their sequence accession numbers is given in Table S1.

### Phylogenetic analysis.

Predicted amino acid sequences were aligned automatically using ClustalW [86] with manual correction in Seaview [87]. Phylogenetic reconstruction was done using amino acid alignments of the longest sequences found for each gene. Only complete sites (no gap) were used. To separate orthologs and paralogs for each sequence, trees were constructed for each group (see Figure S1) with the Phylo_win program [87] using the neighbor-joining method [88] with Poisson-corrected distances on amino acids. Reliability of nodes was estimated by 1,000 bootstrap replicates [89]. Alignments of amino acids were also used to calculate the level of sequence similarities with other vertebrate sequences.

### Whole-mount in situ hybridization.

Whole-mount in situ hybridization was performed as previously described [25]. Several stages were used: gastrula (G), early somitogenesis (ES, 3–6 somites); mid-somitogenesis (MS, 14–18 somites); and 24, 36, and 48 hpf [28]. For several genes, expression was also studied at 5 d post-fertilization. Sense and antisense RNA probes for each gene tested were prepared from partial cDNA. Probes were made against internal coding regions for most NRs, allowing detection of the different 5′ and 3′ isoforms.

### Expression data analysis.

After in situ hybridization, embryos were mounted on slides in 100% glycerol. Pictures were taken with a Leica M420 Macroscope or with a microscope (Leica DM RA2) with differential interference contrast using a digital camera (Coolsnap CCD, Roper Scientific). Digital pictures were saved as TIFF files, then adjusted for contrast, brightness, and color balance using Adobe Photoshop software and stored as such or after conversion to JPEG format to reduce the file size.

To analyze retinal expression in more detail, embryos previously hybridized with a specific probe were postfixed overnight at 4 °C in 4% paraformaldehyde, 3% glutaraldehyde, and phosphate buffer 0.1 M pH 7.4; dehydrated in graded ethanol and propylene oxide; embedded in a mix of araldite and epon; and sectioned (3.5 μm) on a microtome using standard techniques.

The expression patterns were further coded in a binary matrix to quantify their divergence (see Table S2). In this table, all organs in which at least one gene is expressed, are listed (a total of 165 organs for the whole set of developmental stages), and the presence or absence of each gene transcript in each organ is indicated respectively by a “1” or a “0.” All the organs or anatomical structures were labeled with “1” for ubiquitously expressed genes, whereas all organs were marked with “0” for nonexpressed genes.

Starting from this matrix, expression divergence between the duplicates was calculated as the number of gene expression differences (i.e., the number of organs where only one gene in the pair is detected) over the total number of organs where at least one of the genes in the pair is expressed. This means that the same number of differences will give a stronger divergence if the genes concerned have a restricted expression pattern (i.e., if the pair is expressed in only a few organs) than if they are broadly expressed.

Hierarchical clustering analysis was performed using the binary matrix (101 genes versus 166 anatomical structures; Table S2). We excluded 13 genes for which no expression was detected in the 166 organs, and 31 genes ubiquitously expressed in all structures (except in the yolk syncytial layer). Thus, only genes with regionalized expression (detected here in a number of organs between 1 and 41) were included in the analysis. We have verified that the inclusion of ubiquitous and undetected genes in the analysis does not modify the overall conclusions of the hierarchical analysis. Similarities between the expression patterns of the 57 genes and also between the patterns of anatomical structures were computed as Jaccard's coefficient, which is classically employed for species presence–absence data in ecology [90]. Jaccard's coefficient is an asymmetrical binary coefficient, which does not take into account the case of absence/absence in the degree of similarity between two binary patterns. It is suitable in the framework of expression data, because the presence (i.e., the detection) of a gene in an organ is more informative in terms of expression or not than its absence due to the existence of detection thresholds. Distances between the expression patterns of genes and between the patterns of organs were calculated as *d* = sqrt(1 − *s*), with *s* being the similarity coefficient. Dendrograms were built using the two sets of distances (genes and organs) by hierarchical clustering following the Ward's method. We performed all analyses with the R software (http://www.R-project.org) using the package ade4 [91] to compute distances between expression patterns.

### Sequence analysis.

The protein sequences of each pair of actinopterygian-specific paralogs were aligned with the orthologous nonduplicated protein sequence of the outgroup using ClustalX [86]. We used the closest appropriate outgroup (having diverged before the actinopterygian genome duplication) being completely sequenced (H. sapiens). We used RRTree [92] on these protein alignments to make relative rate tests and thus evaluate differences in protein evolution rates of the duplicates. Nucleotide alignments of the corresponding coding sequences were obtained based on the protein alignments. We used Gestimator (analysis-0.6.6 by K. Thornton) to compute the Ka/Ks ratios for each pair of duplicates with Comeron's method [93].

## Supporting Information

Figure S1 Phylogenetic Trees of the NRs and Their Coregulators The trees were calculated using the neighbor-joining method with Poisson-corrected distances on amino acids. Sequences have been treated group by group, according to the official nomenclature of NRs. For coregulators, paralogous sequences have been treated collectively. The zebrafish sequences are in red. Reliability of the nodes was estimated by 1,000 bootstrap replicates. The boostrap values are indicated for the relevant branch only when they are above 50%.
(171 KB PDF)

Figure S2 Schematic Representation of NR Coregulators Whose Expression Pattern Was Determined in This Study These proteins are indicated in purple and blue for coactivators and corepressors, respectively. NRs are represented bound to their response element in the promoter region of a target gene. The coregulators can be associated in three main types of complexes, namely the SWI/SNF complex, which remodels chromatin structure; the CBP/P300-PCAF complex, which possesses histone deacetylase activity; and the TRAP/DRIP/SMCC complex, also called the mediator complex, which interacts with the basal transcription machinery. In addition, the figure depicts the NCoR/SMRT corepressor complex, which harbors histone deacetylase activity. The other coregulators implicated in mediating NR activity that are not part of one of these complexes but have also been incorporated in this study are presented at the bottom left of the figure.
(223 KB JPG)

Figure S3 Spatiotemporal Expression Pattern of All Members of the NR superfamily (70 Genes) and of Their Main Coregulators (31 Genes) Expression patterns of these 101 genes are described on each panel by a full annotation of the anatomical structures expressing these genes at the different developmental stages. Annotation in red points to unlabeled parts of organs. Except when mentioned, all embryos are presented in standard view with the anterior pointing to the left, except at gastrula stage, where the anterior part (animal pole) points to the top. Captions can be found in Text S1.
(304.4 MB PDF)

Figure S4 Detailed Analysis of the 26 NRs Expressed in the Retina Captions in Text S1
(130.3 MB PDF)

Figure S5 Extensive Overlapping Domains of Expression between RAR, RXR, and SRC3 RAR, RXR, and SRC3 display obvious extensive similiarities of expression patterns at different developmental stages in posterior hindbrain, anterior spinal chord, and in the tail bud region, suggesting a functional link between the coactivator SRC3 and the RXR-RARs. Embryos are in lateral view, anterior to the left. For A, C, E, G, I, K, M, and O, embryos are at the 15-somite stage, while for B, D, F, H, J, L, N, and P, they are at 24 hpf.
(246 KB JPG)

Figure S6 Extensive Overlapping Expression between Members of the COUPTF Subfamily Members of the COUPTF family display extensive overlapping expression patterns in the CNS. Of note, the two EAR2 genes (NR2F6-A and NR2F6-B) are distant members of the COUP-TF group (NR2F) and are even often called COUP-TFγ in mammals [21–22]. To avoid confusion, we kept their original name. In particular COUPTFα-A, COUPTFβ, and COUPTFα-B belong to the same synexpression group characterized by expression in ventral diencephalon, forebrain ventricular zone, anterior tegmentum, and hindbrain (A, C, and E at 36 hpf and B, D, and F at the middle of the somitogenesis stage). COUPTFγ overlaps with this synexpression group in the hindbrain and displays extensive coexpression (H) with EAR2 in the middle of the somitogenesis stage (J) and in the hindbrain and anterior spinal chord at 36 hpf (G and I). Embryos are in lateral view, anterior to the left. The complete anatomical descriptions of these expression patterns are presented in Figure S3 and Text S1.
(237 KB JPG)

Figure S7 Expression of RXRγB (A) Embryo at the gastrula stage in lateral view. RXRγB is not expressed at this stage. (B–D) Embryo at the 2-somite stage in lateral view (B), dorsal view (D), and optical cross section at the level of the presumptive anterior spinal cord. Expression starts in presumptive anterior spinal cord and paraxial mesoderm. A transient expression is also observed in Kupffer's vesicle (D). (E–H) Embryo at the 5-somite stage in lateral view (E), dorsal view (H), and in optical cross section at the level of the presumptive anterior spinal cord (F) and of the cephalic region (G). (I–K) Embryo in the middle of somitogenesis in lateral view (I), in dorsal view (J), and in optical cross section (K). Expression is observed in the anterior spinal cord as a ventral-to-dorsal gradient with almost no expression in the dorsal spinal cord. (l) Embryo at 24 hpf in lateral view. (M) Embryo at 36 hpf in lateral view. (N–P) Embryo at 48 hpf in lateral view (N), oblique view (O), and dorsal view (P). In addition to the expression in the anterior spinal cord, RXRγB is also expressed in liver and in the photoreceptor cell layer of the retina.
(252 KB JPG)

Table S1 Nomenclature, Name, and Genbank Accession Number for Each NR or Coregulator Gene for Which We Studied the Expression Pattern during Zebrafish Embryogenesis (18 KB PDF)

Table S2 Organs or Embryonic Territories Expressing NR and Coregulator Genes All the organs or embryonic territories in which at least one gene was expressed at a given developmental stage are listed. A 0/1 code was used to describe the expression of each gene. For ubiquitously expressed genes, all the organs or anatomical structures were associated with 1, whereas for genes whose expression could not be detected in any organ we indicated a 0.
(108 KB PDF)

Table S3 Type of Expression Observed at Each Developmental Stage for Each Studied Gene At each stage a given gene could be in three categories: ubiquitous expression, restricted expression pattern, or not expressed.
(35 KB DOC)

Table S4 Relative Rates of Protein Evolution, Coding Sequence Divergence, and Expression Pattern Divergence of the Fish-Specific NR Duplicates “gene1” and “gene2” indicate the members of the pair. The corresponding group inside the NR family is also indicated. Acceleration corresponds to the ratio between the protein evolution rates, calculated by RRTree (see Materials and Methods for details); the *p*-value of the relative evolution rate is shown for each pair. Ka and Ks were calculated by Gestimator based on the cds sequence alignment of the duplicates. 999 indicates that Ks is too saturated to be calculated. Expression divergence corresponds to the number of differences found between the expression patterns of the two genes divided by the total number of organs/tissues where gene pair expression is detected.
(24 KB PDF)

Table S5 Abbreviations Used in Figure 6
(38 KB PDF)

Text S1 Click here for additional data file. Detailed Description of Expression Patterns Shown in Figures S3 and S4
(86 KB DOC)

## Abbreviations

CNS: central nervous system
hpf: hours post-fertilization
INL: inner nuclear layer
MS: mid-somitogenesis
NR: nuclear receptor
ONL: outer nuclear layer
RT-PCR: reverse transcriptase PCR

## Note Added in Proof

Isolation of a new RXR duplicate, RXRγ-B (NR2B3-B), was published while this article was at the proofs stage. Reference: Wasman JS, Yelon D (2007) Comparison of the expression patterns of newly identified zebrafish retinoic acid and retinoid X receptors. Dev Dyn 236: 587–595.

The total number of NR genes present in the zebrafish genome is thus 71. As for all the other NR genes studied, we performed whole-mount in situ hybridization. The panel caption and link are below.
