# Supplementary material for: Unexpected Novel Relational Links Uncovered by Extensive Developmental Profiling of Nuclear Receptor Expression
Source: PLoS Genet. 2007 Nov 9;3(11):e188. doi: 10.1371/journal.pgen.0030188 (PMC2065881; doi:10.1371/journal.pgen.0030188)
Supplement: Supplementary file 1 [file pgen.0030188.sg001.pdf]

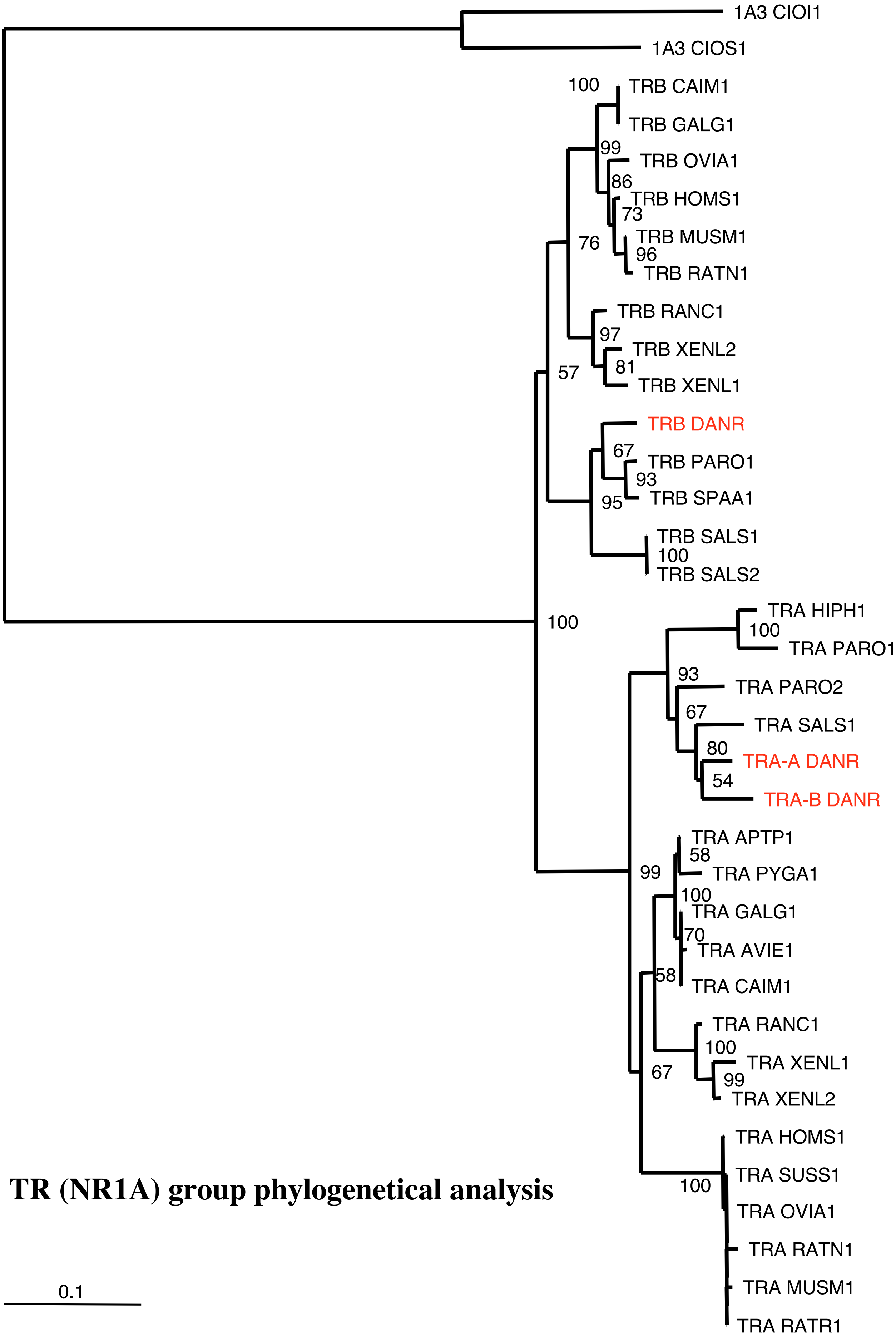

TR (NR1A) group phylogenetical analysis

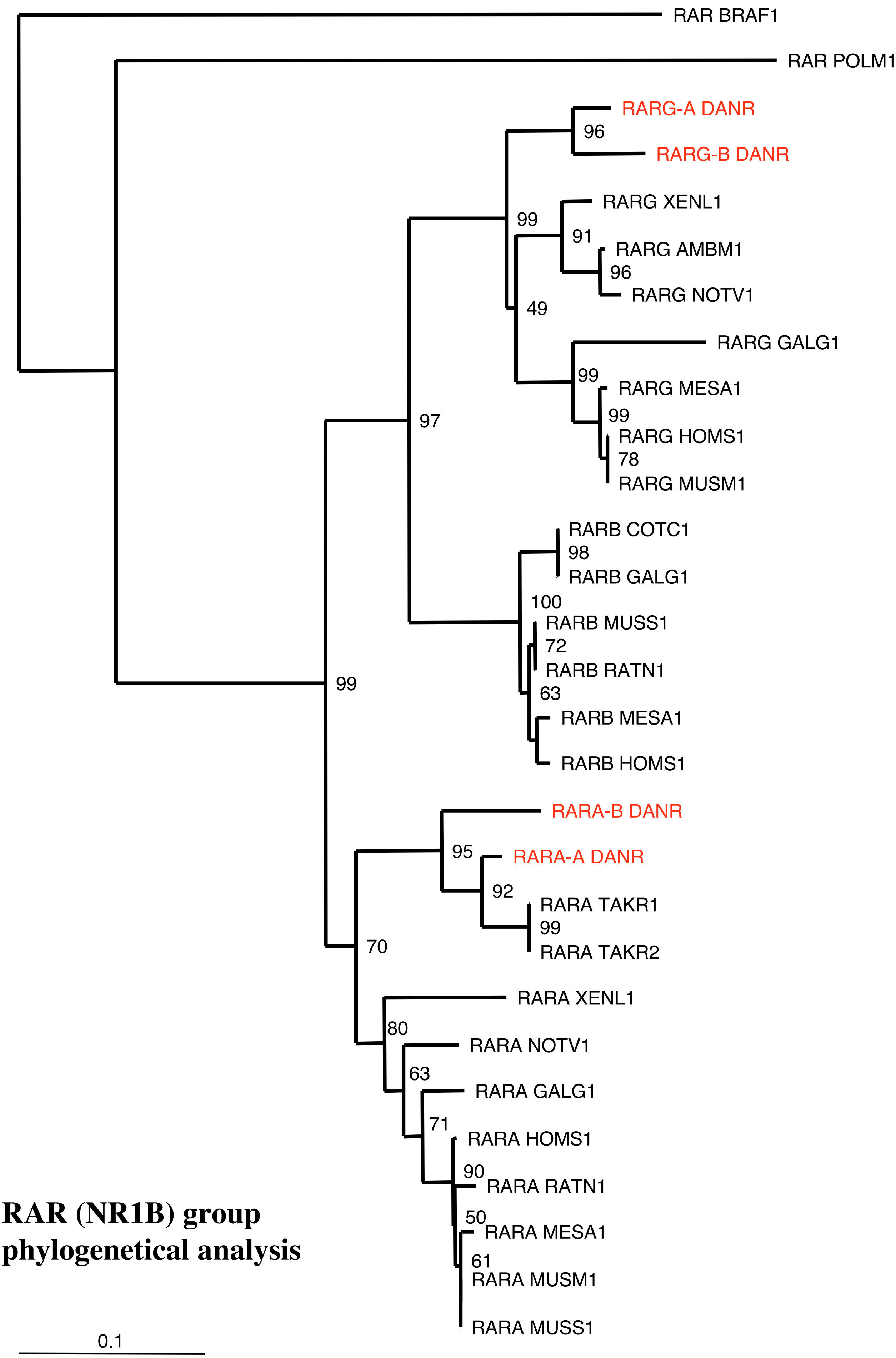

RAR (NR1B) group phylogenetical analysis

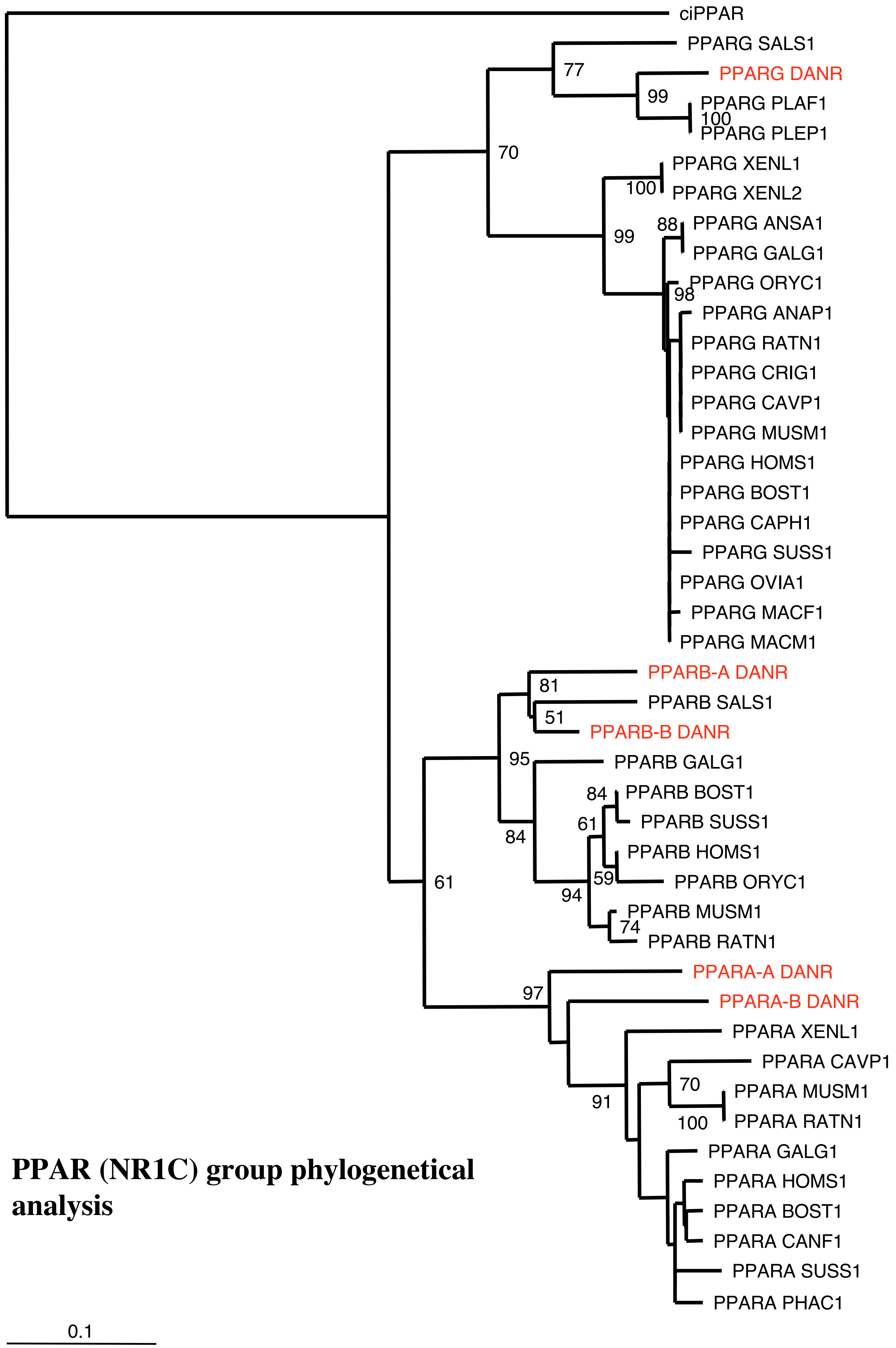

PPAR (NR1C) group phylogenetical analysis

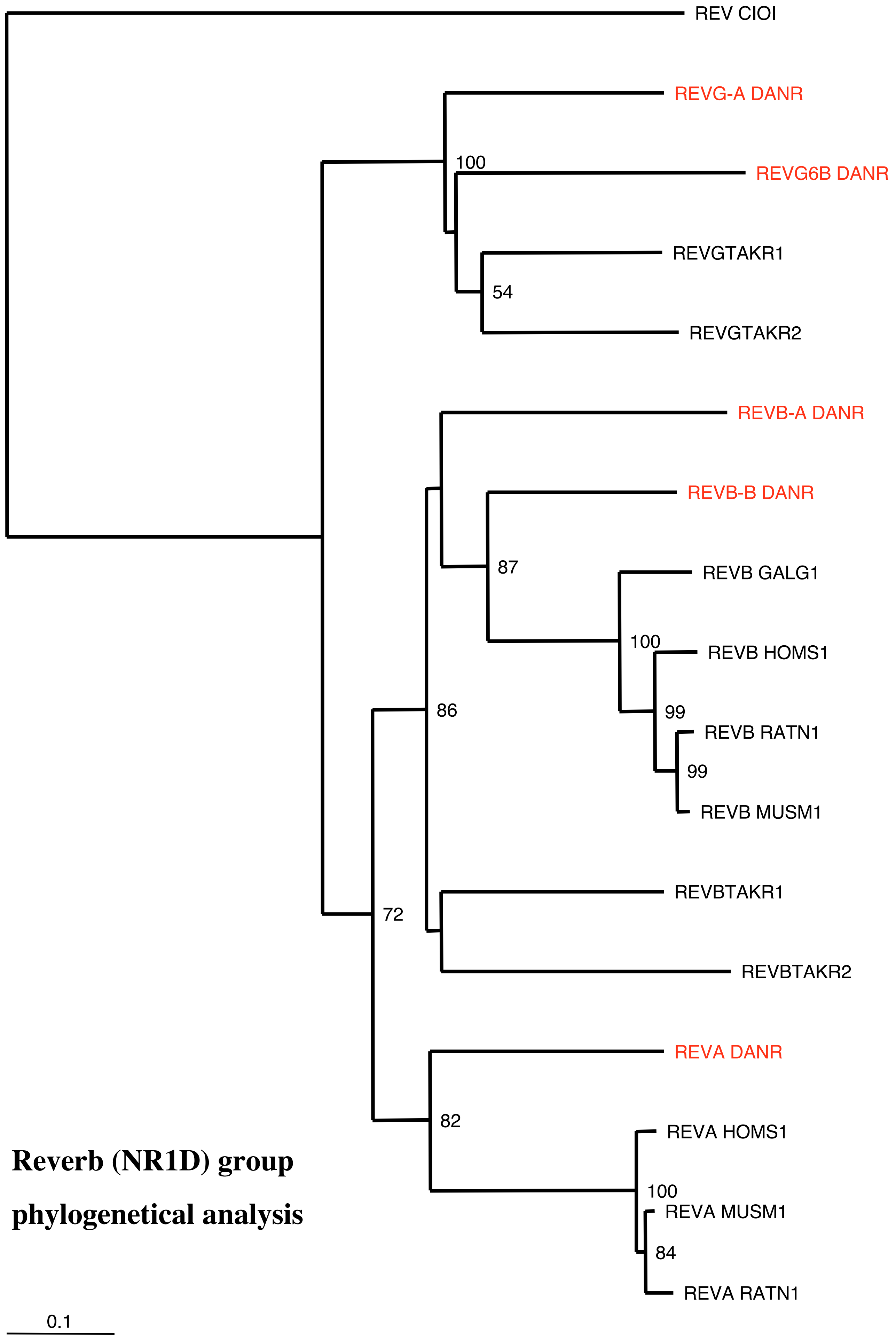

Reverb (NR1D) group

phylogenetical analysis

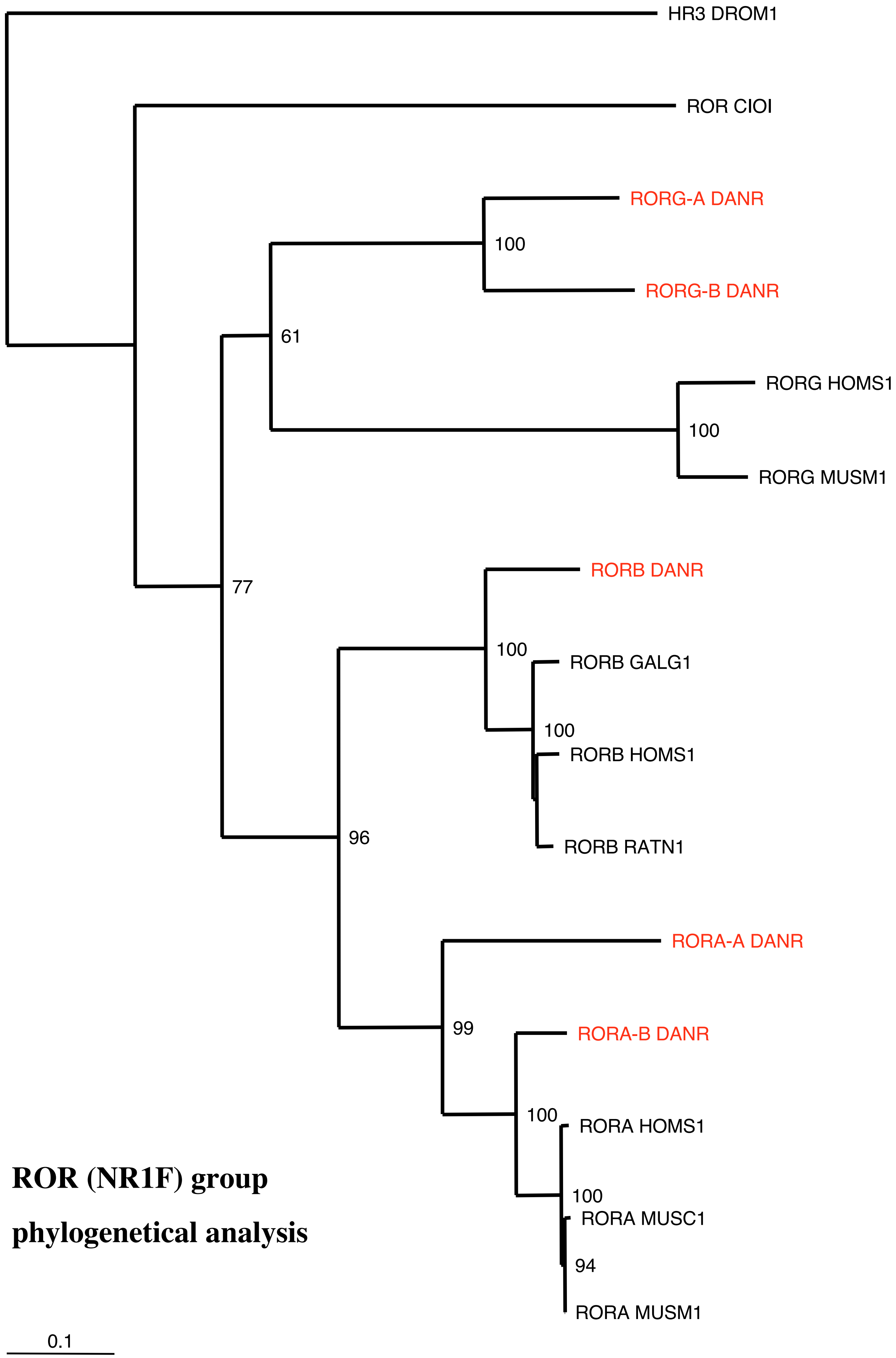

ROR (NR1F) group

phylogenetical analysis

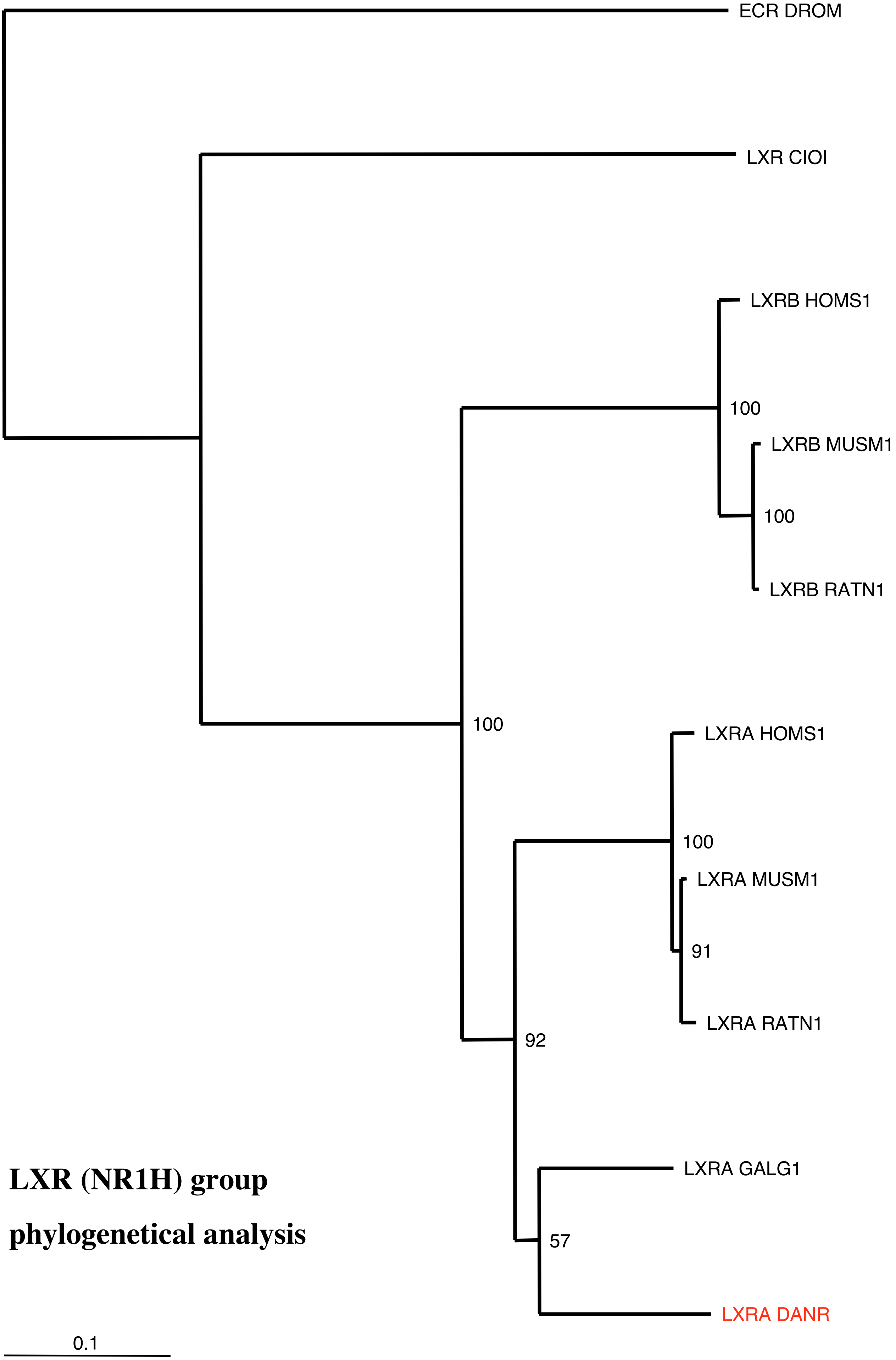

LXR (NR1H) group

phylogenetical analysis

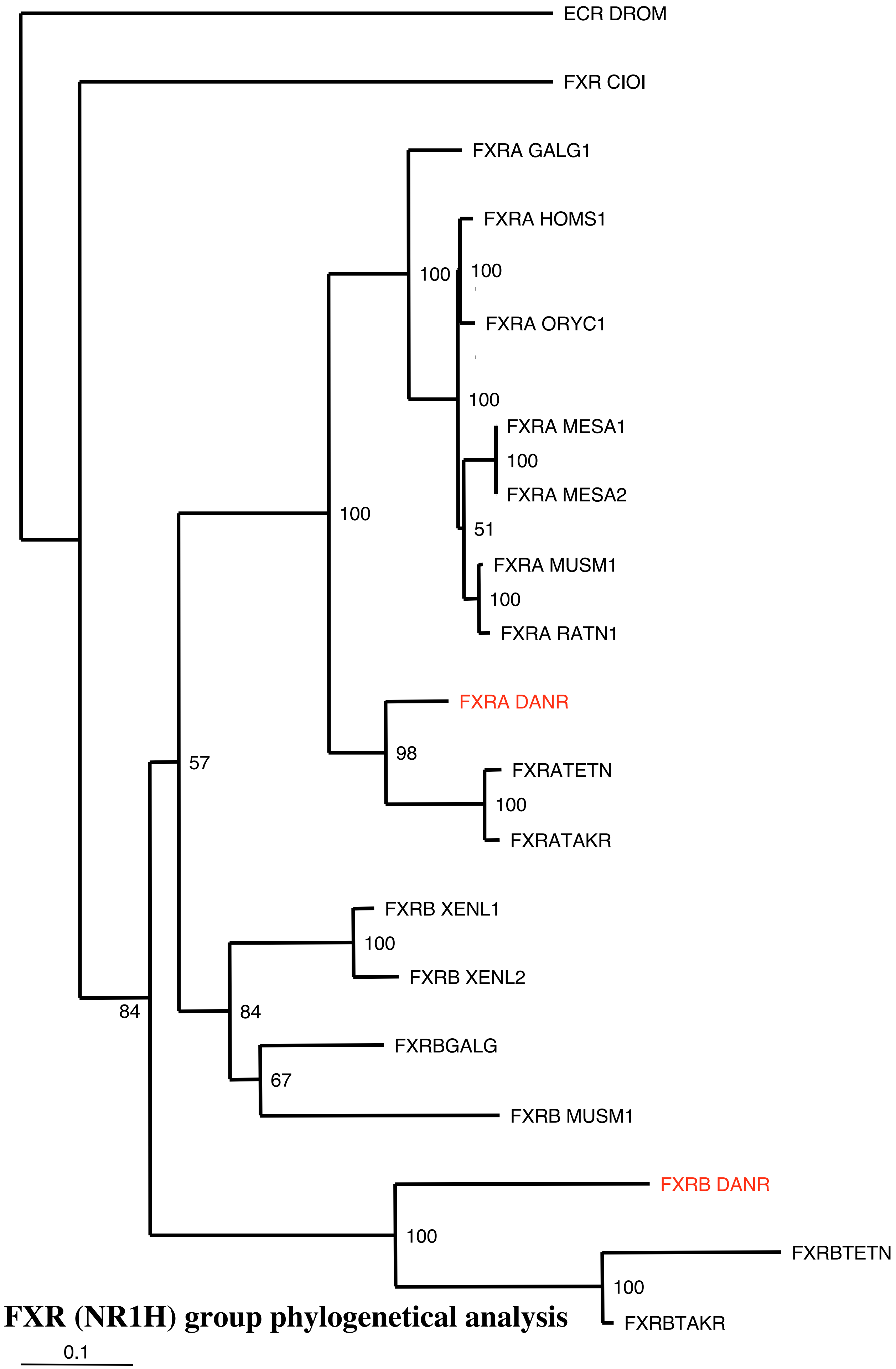

FXR (NR1H) group phylogenetical analysis

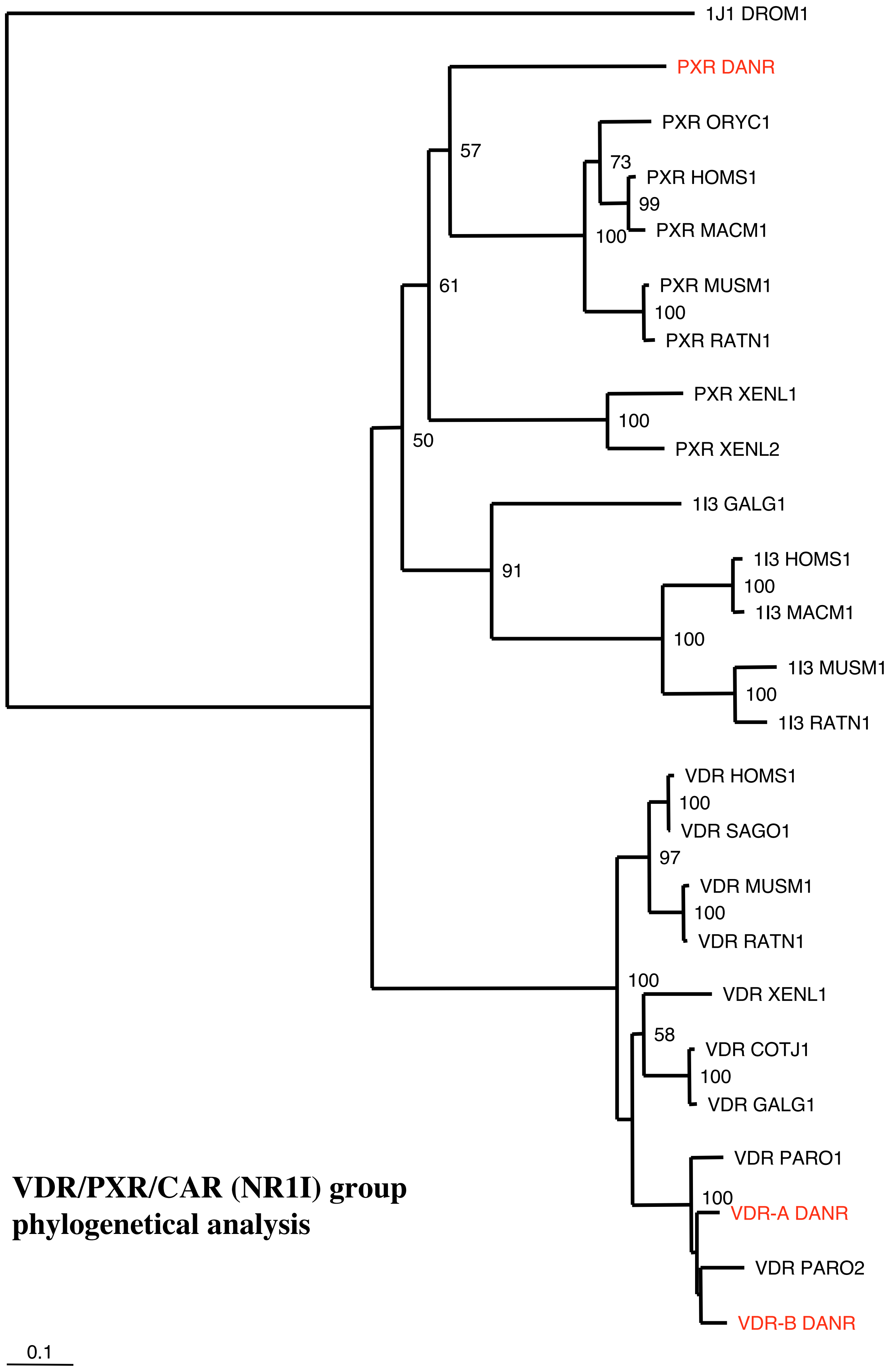
1J1 DROM1
PXR DANR
PXR ORYC1
PXR HOMS1
PXR MACM1
PXR MUSM1
PXR RATN1
PXR XENL1
PXR XENL2
1I3 GALG1
1I3 HOMS1
1I3 MACM1
1I3 MUSM1
1I3 RATN1
VDR HOMS1
VDR SAGO1
VDR MUSM1
VDR RATN1
VDR XENL1
VDR COTJ1
VDR GALG1
VDR PARO1
VDR-A DANR
VDR PARO2
VDR-B DANR
57
73
99
100
61
100
100
50
91
100
100
100
100
97
100
100
58
100
100
0.1
VDR/PXR/CAR (NR1I) group
phylogenetical analysis

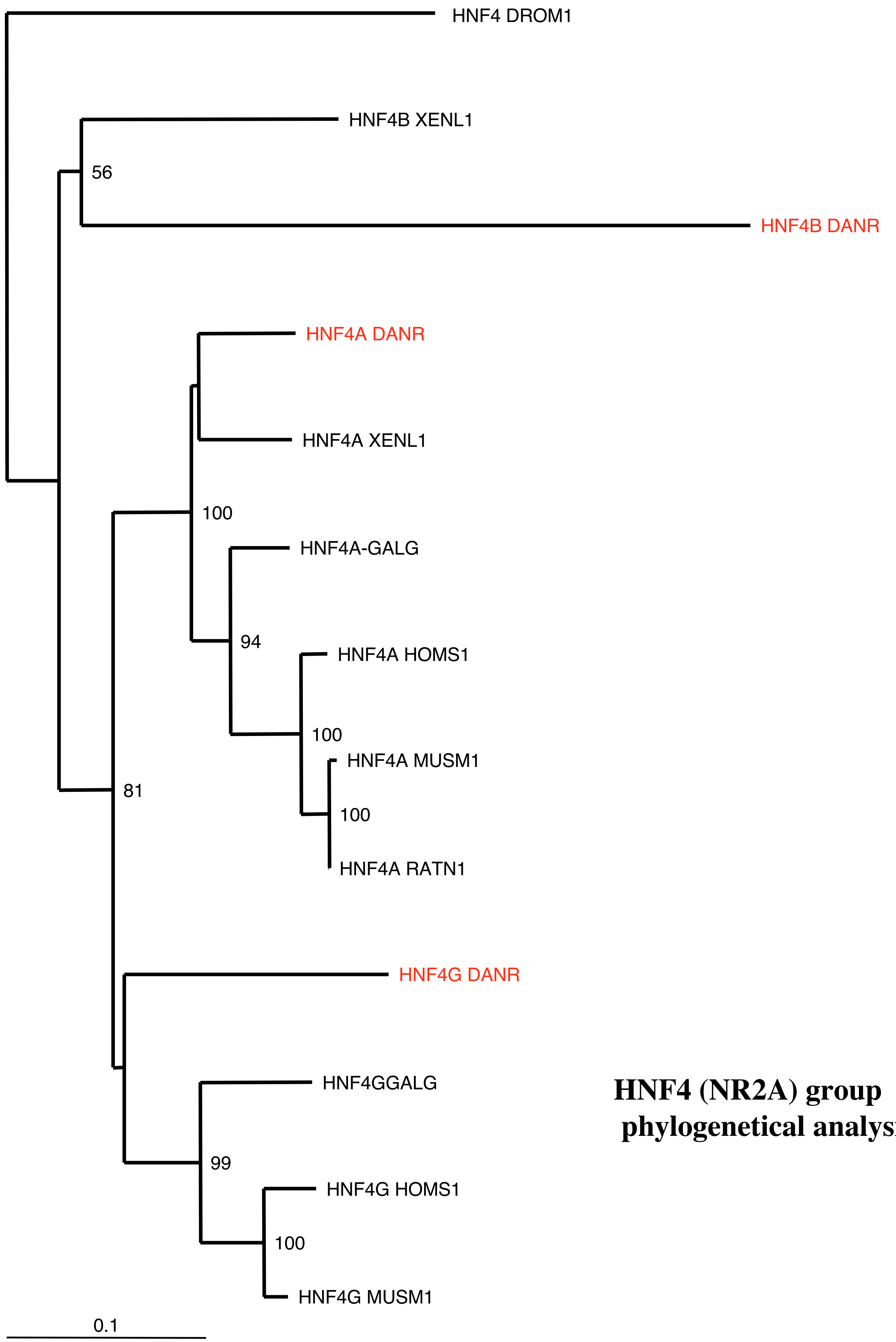

HNF4 (NR2A) group phylogenetical analysis

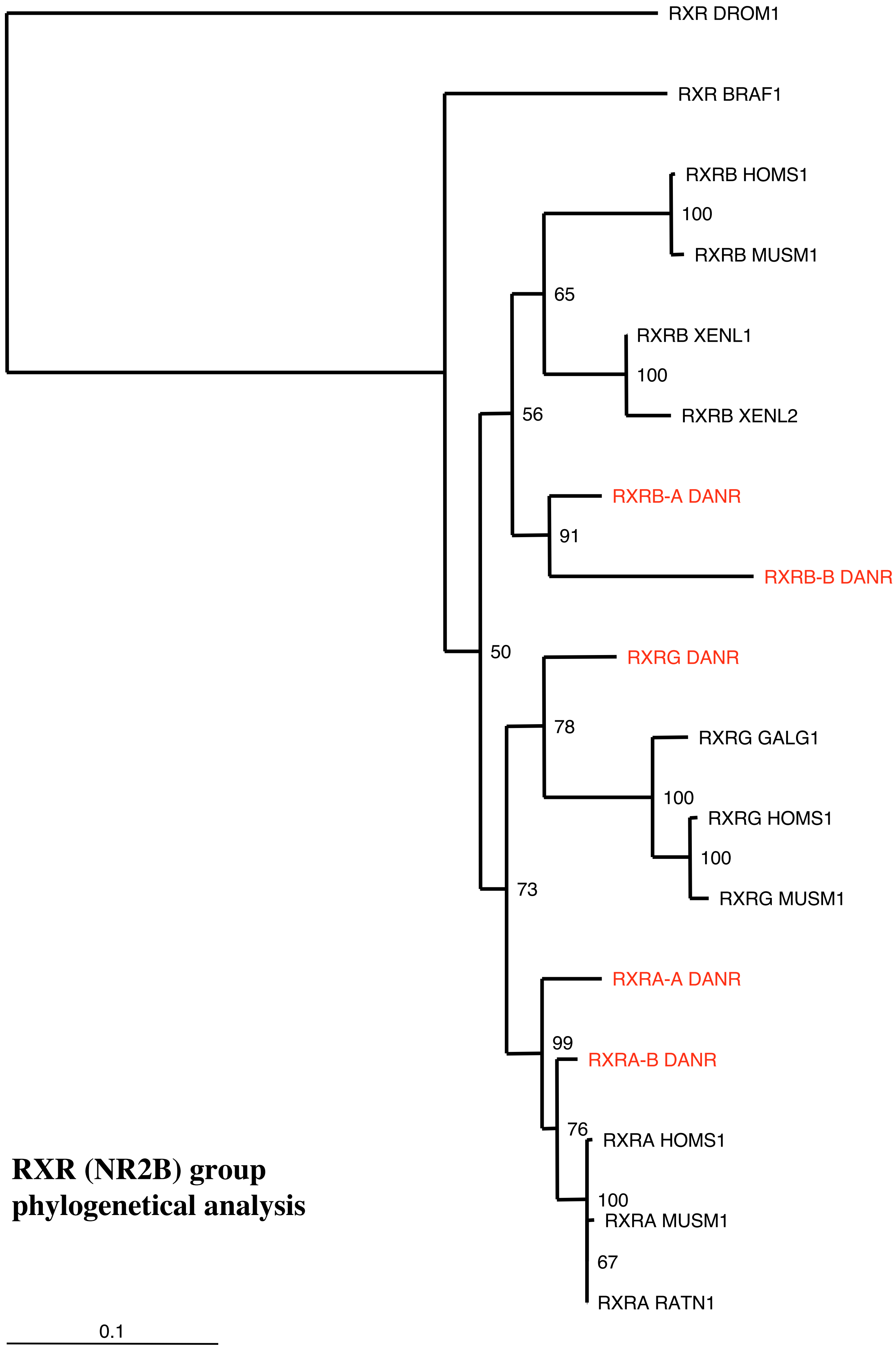
RXR DROM1
RXR BRAF1
RXRB HOMS1
100
RXRB MUSM1
65
RXRB XENL1
100
RXRB XENL2
56
RXRB-A DANR
91
RXRB-B DANR
50
RXRG DANR
78
RXRG GALG1
100
RXRG HOMS1
100
RXRG MUSM1
73
RXRA-A DANR
99
RXRA-B DANR
76
RXRA HOMS1
100
RXRA MUSM1
67
RXRA RATN1
RXR (NR2B) group
phylogenetical analysis
0.1

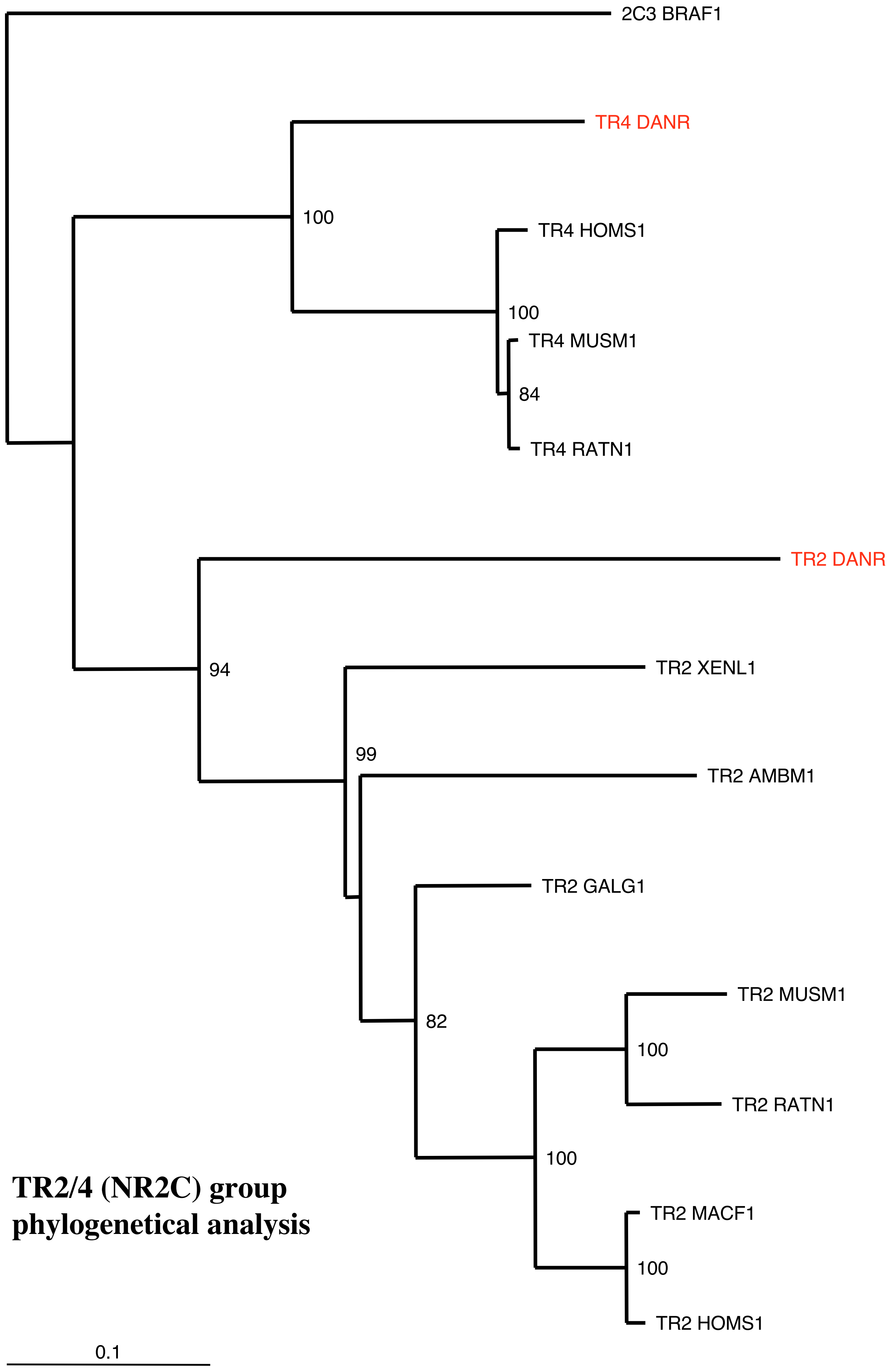

TR2/4 (NR2C) group phylogenetical analysis

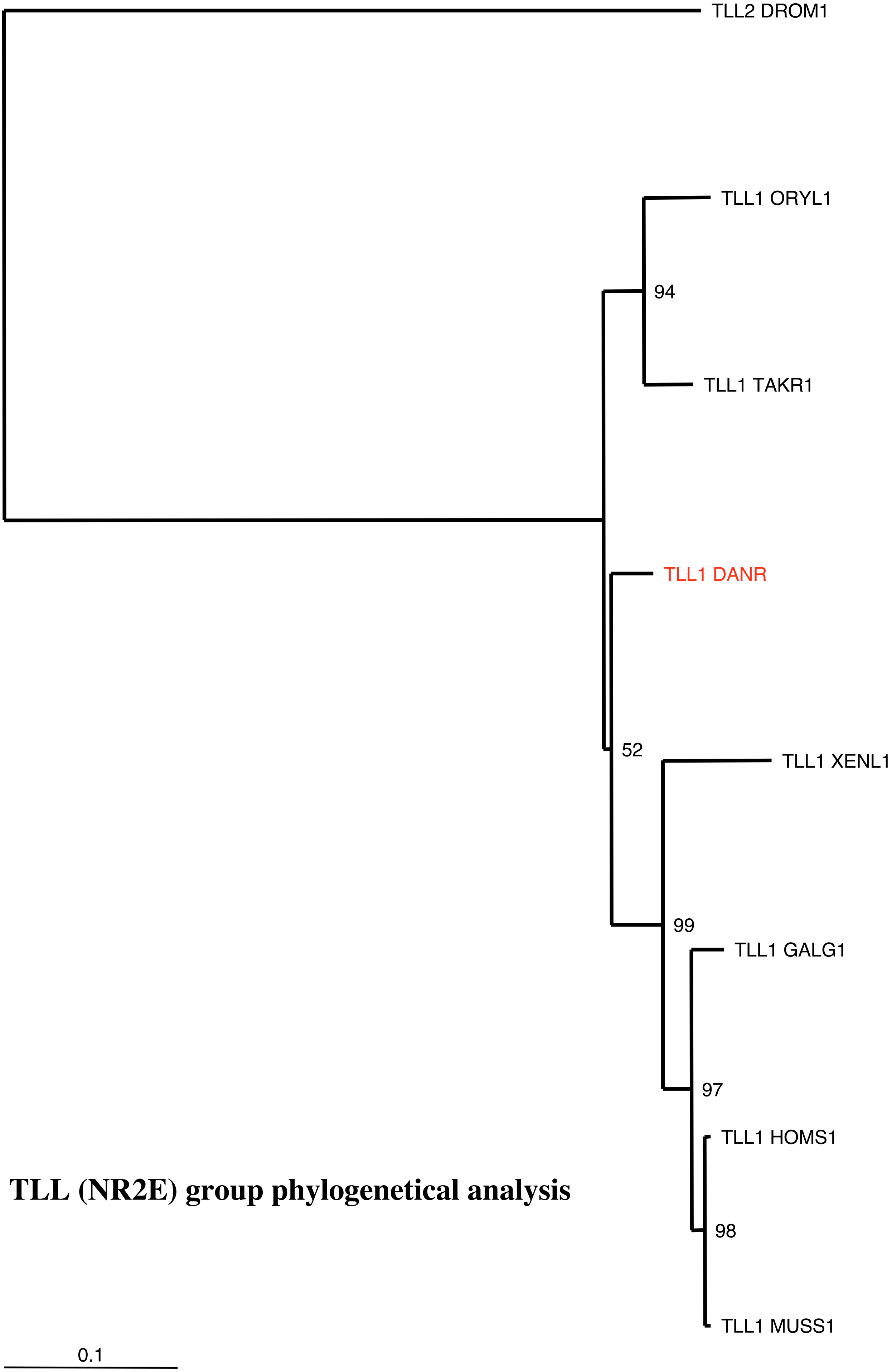

TLL (NR2E) group phylogenetical analysis

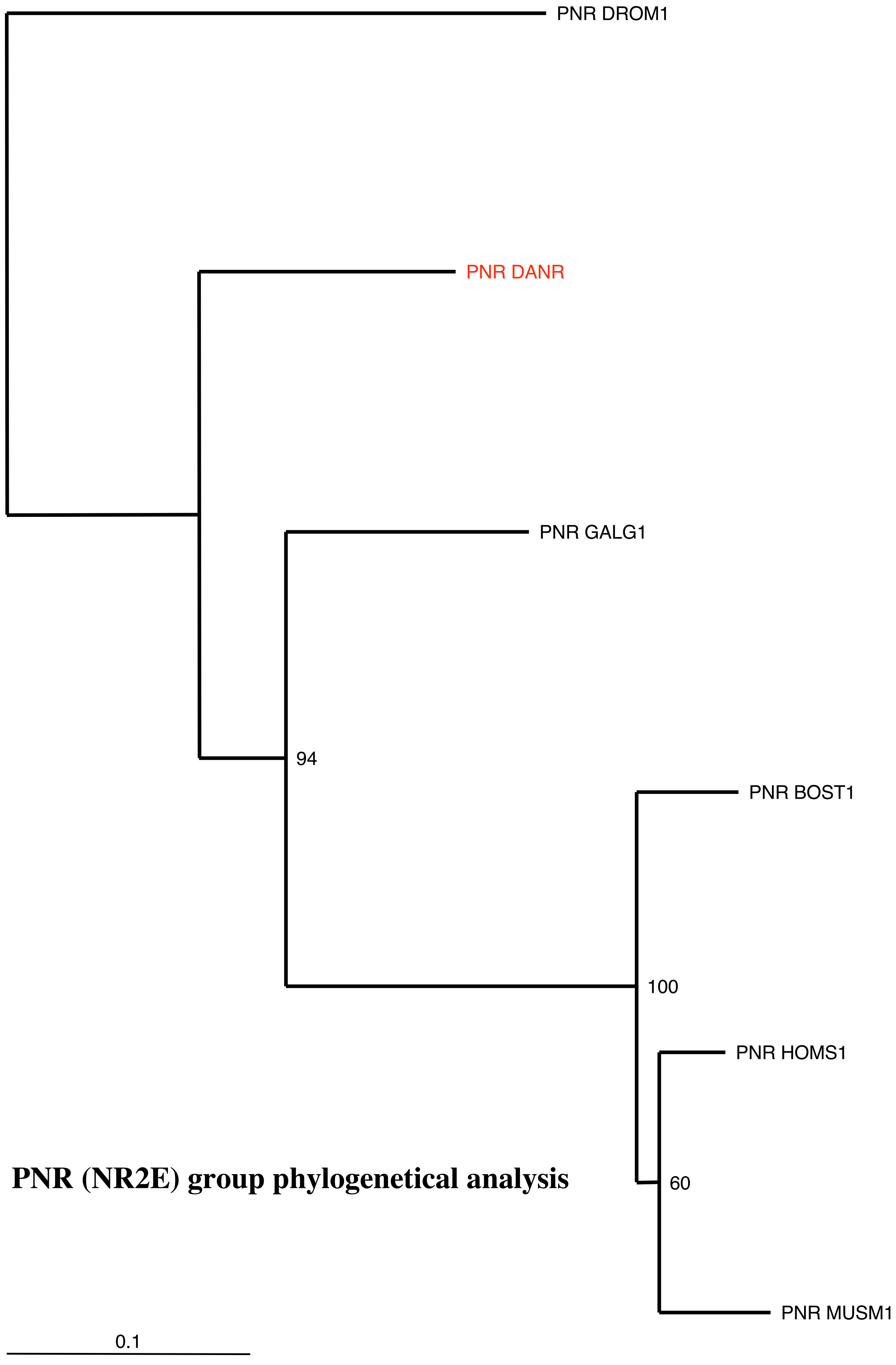

PNR (NR2E) group phylogenetical analysis

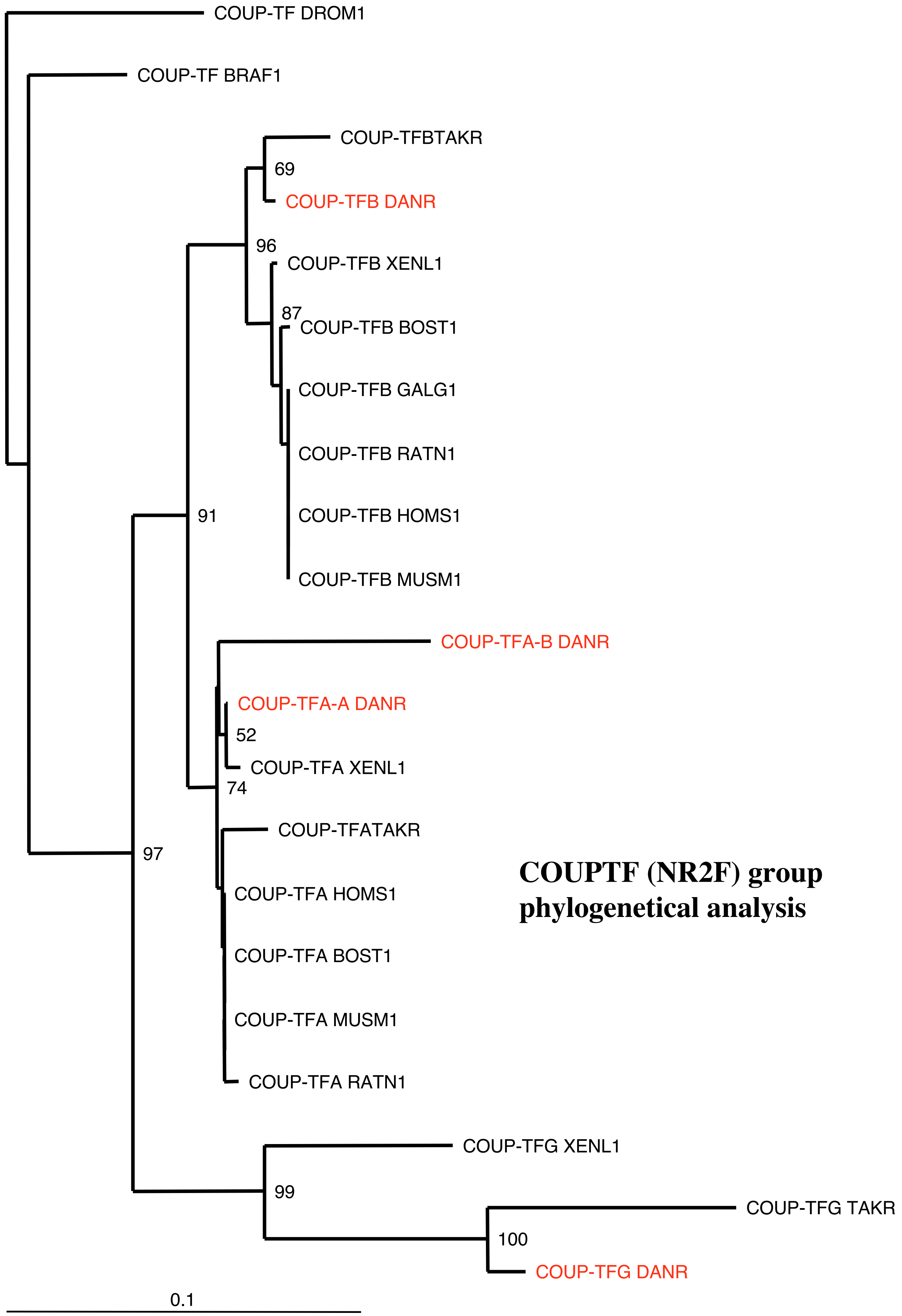

COUPTF (NR2F) group phylogenetical analysis

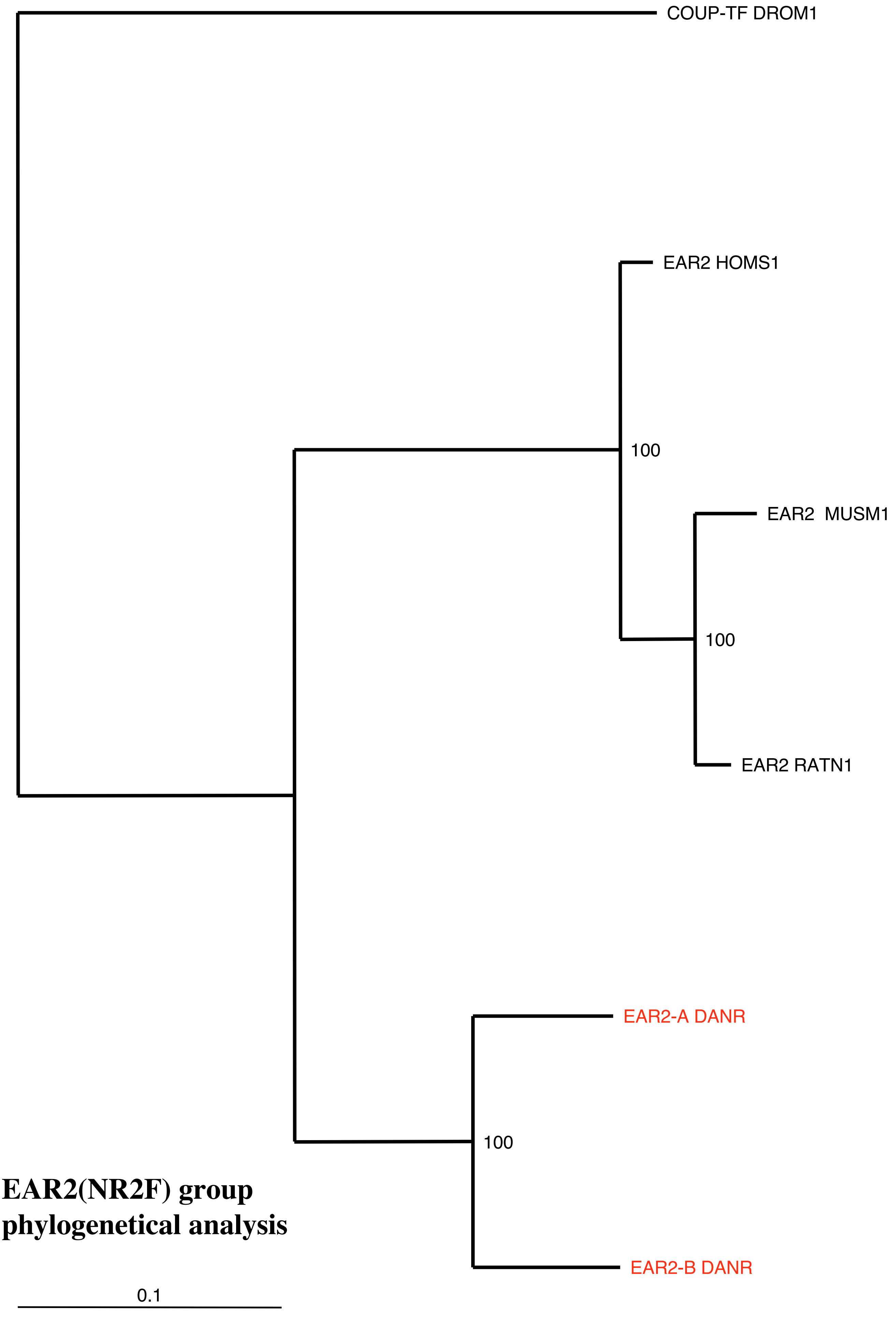

EAR2(NR2F) group phylogenetical analysis

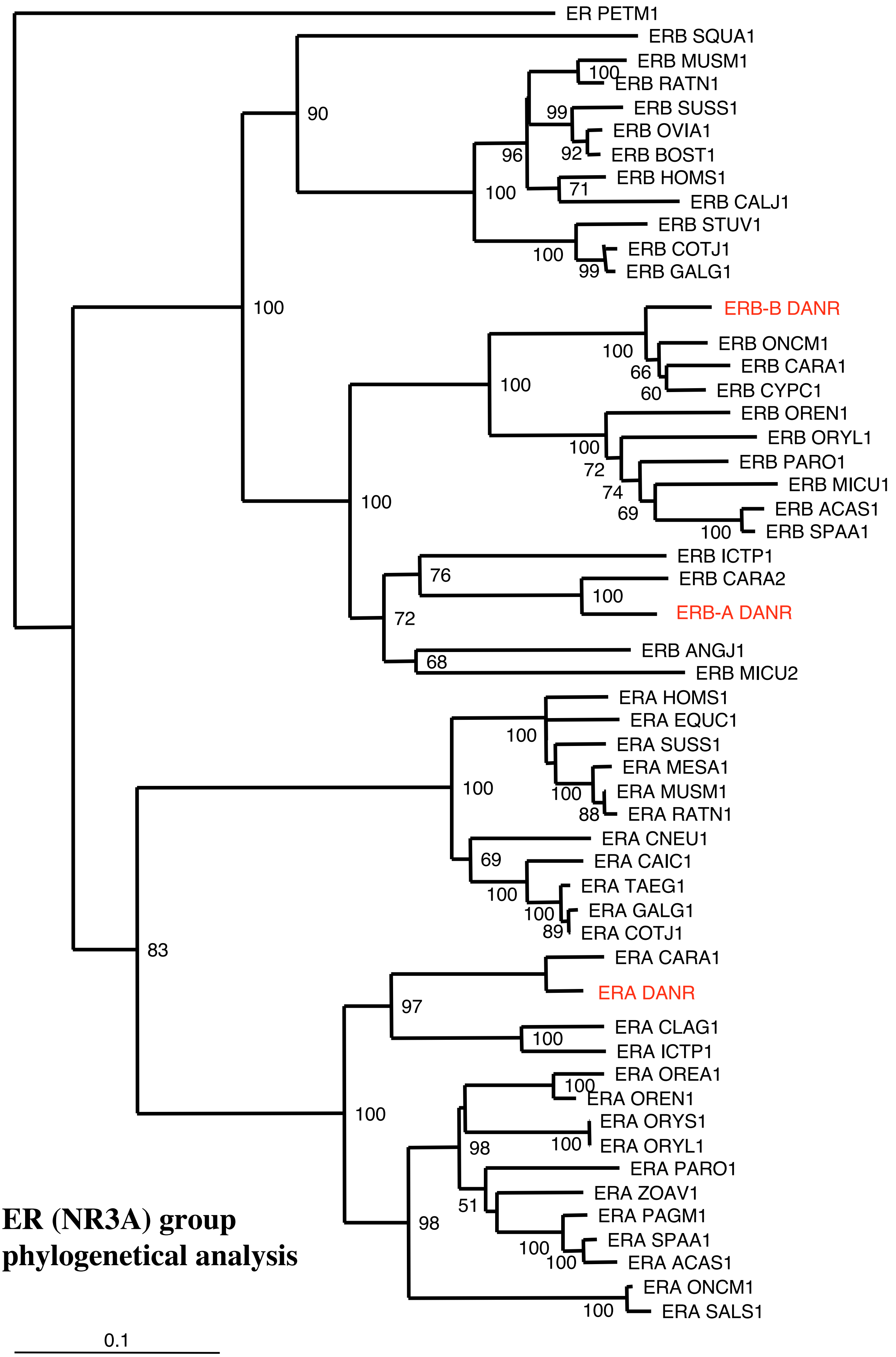

ER (NR3A) group phylogenetical analysis

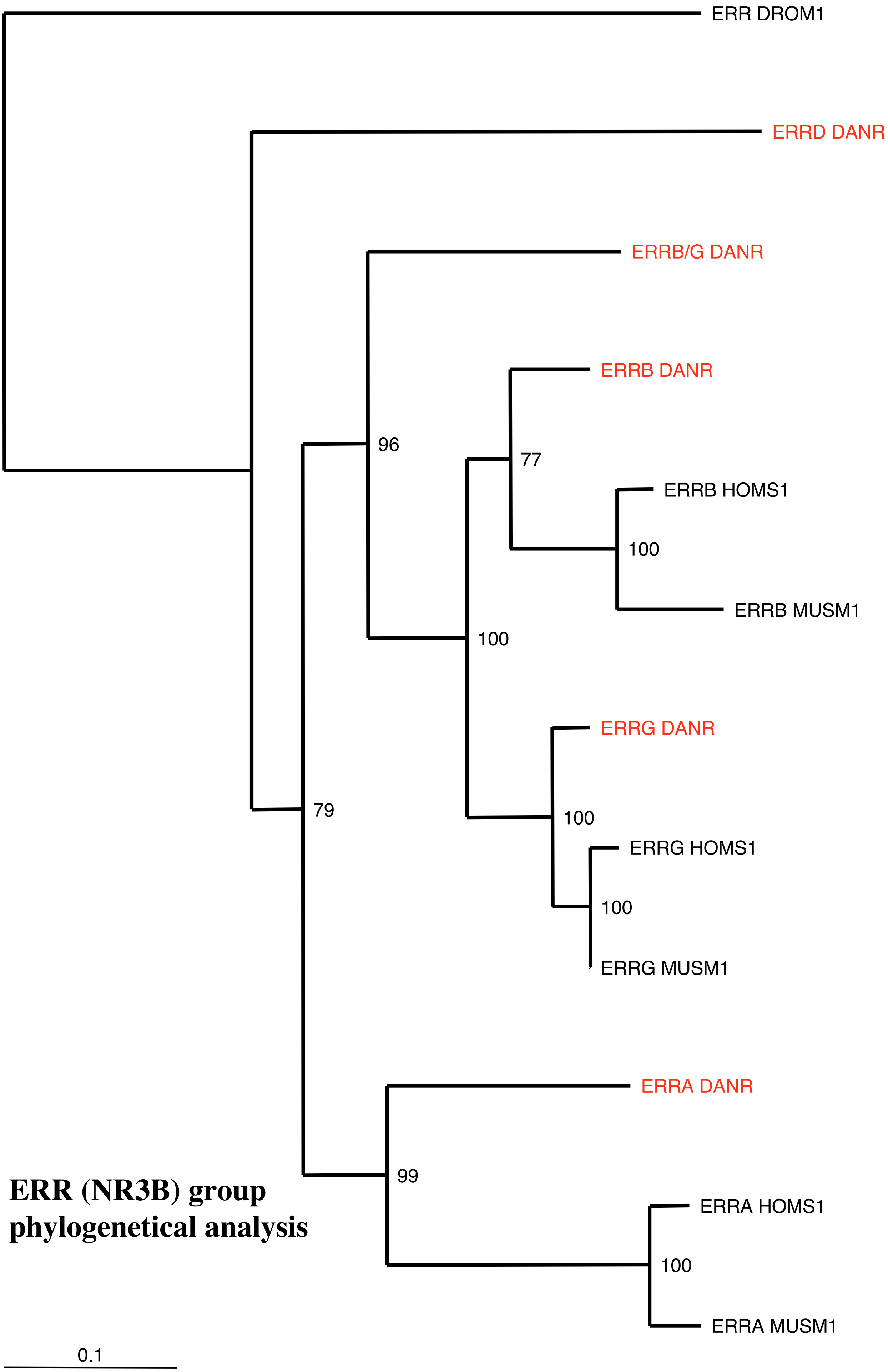

ERR (NR3B) group phylogenetical analysis

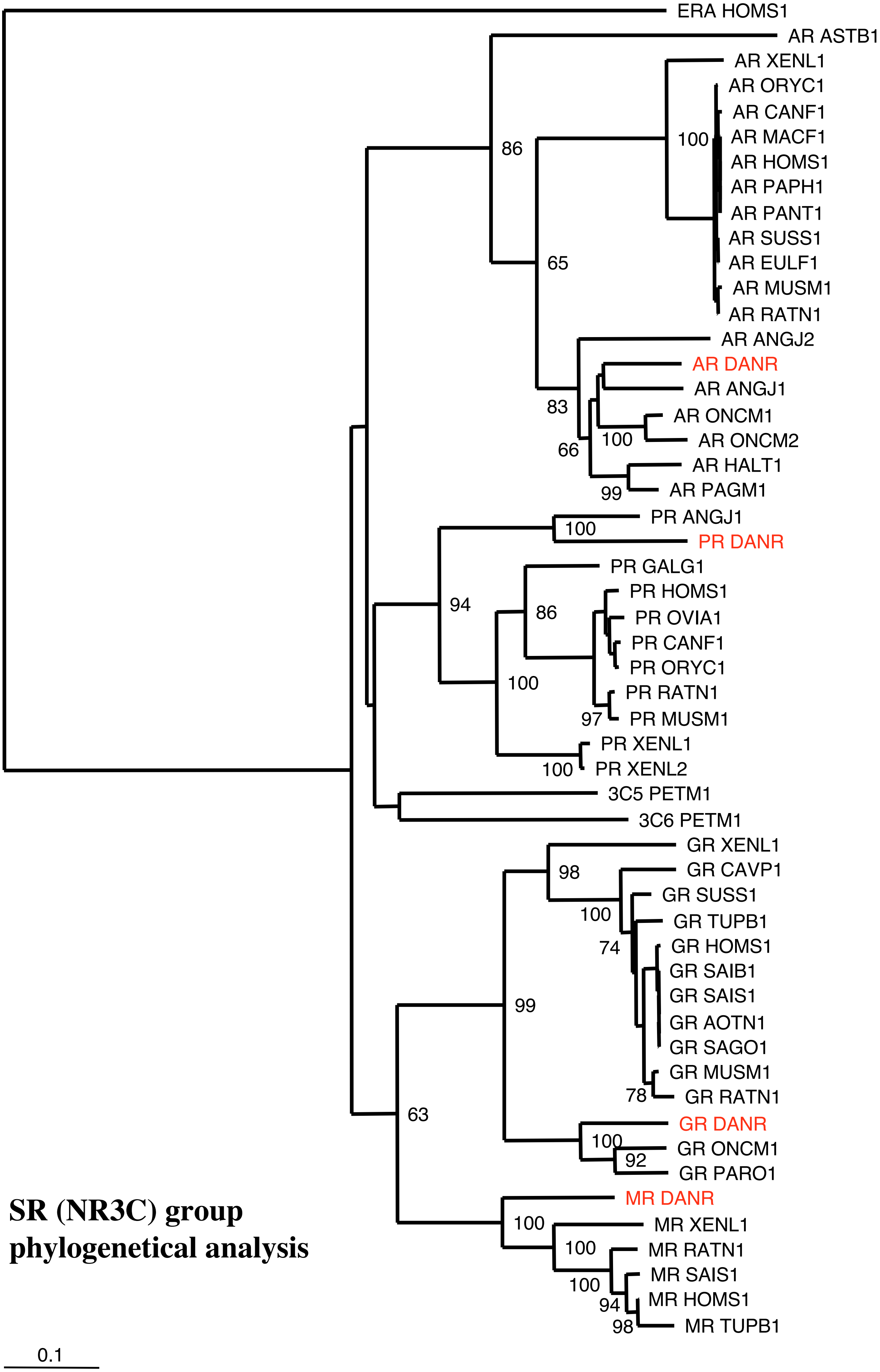

SR (NR3C) group phylogenetical analysis

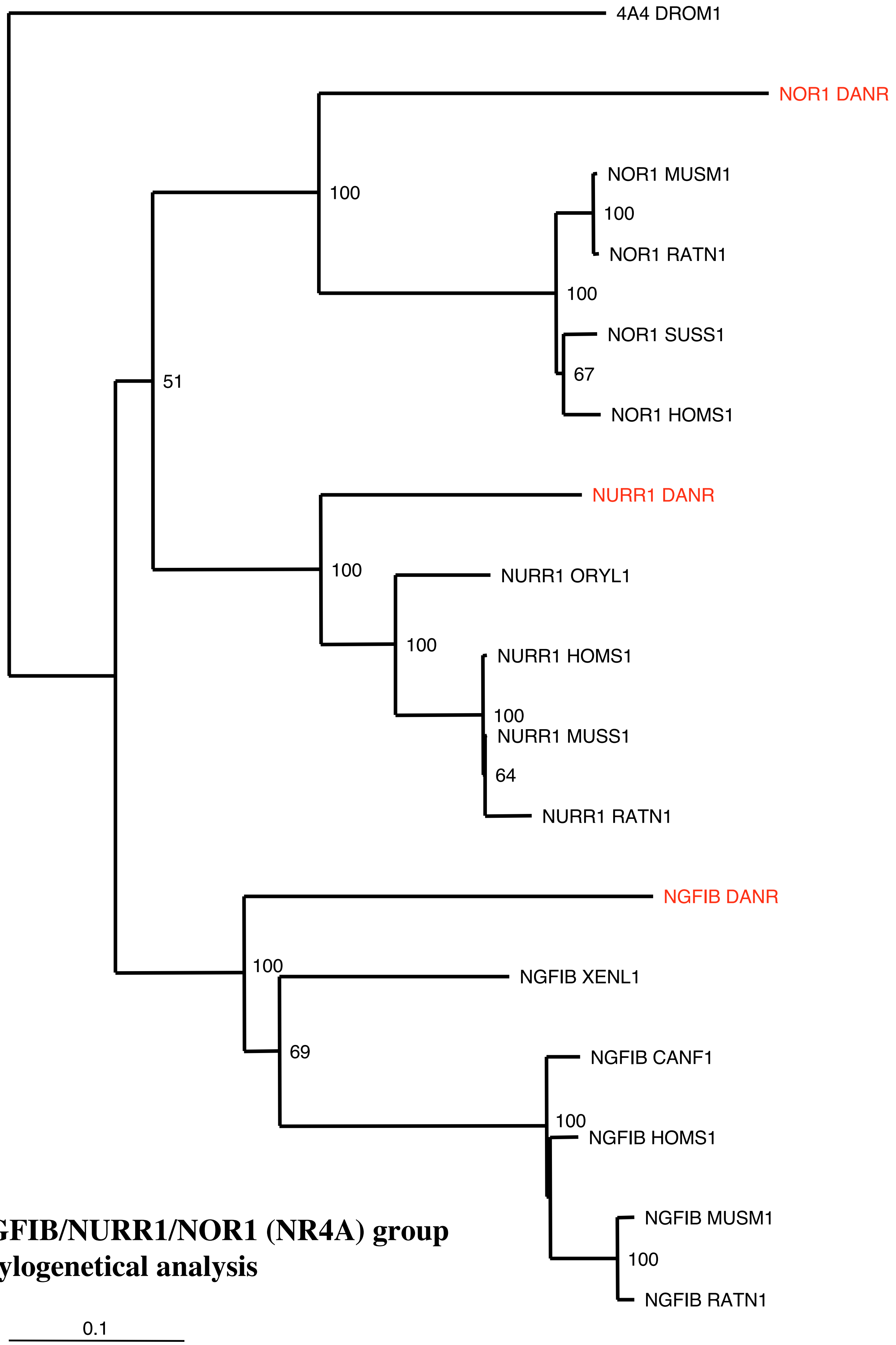

NGFIB/NURR1/NOR1 (NR4A) group phylogenetical analysis

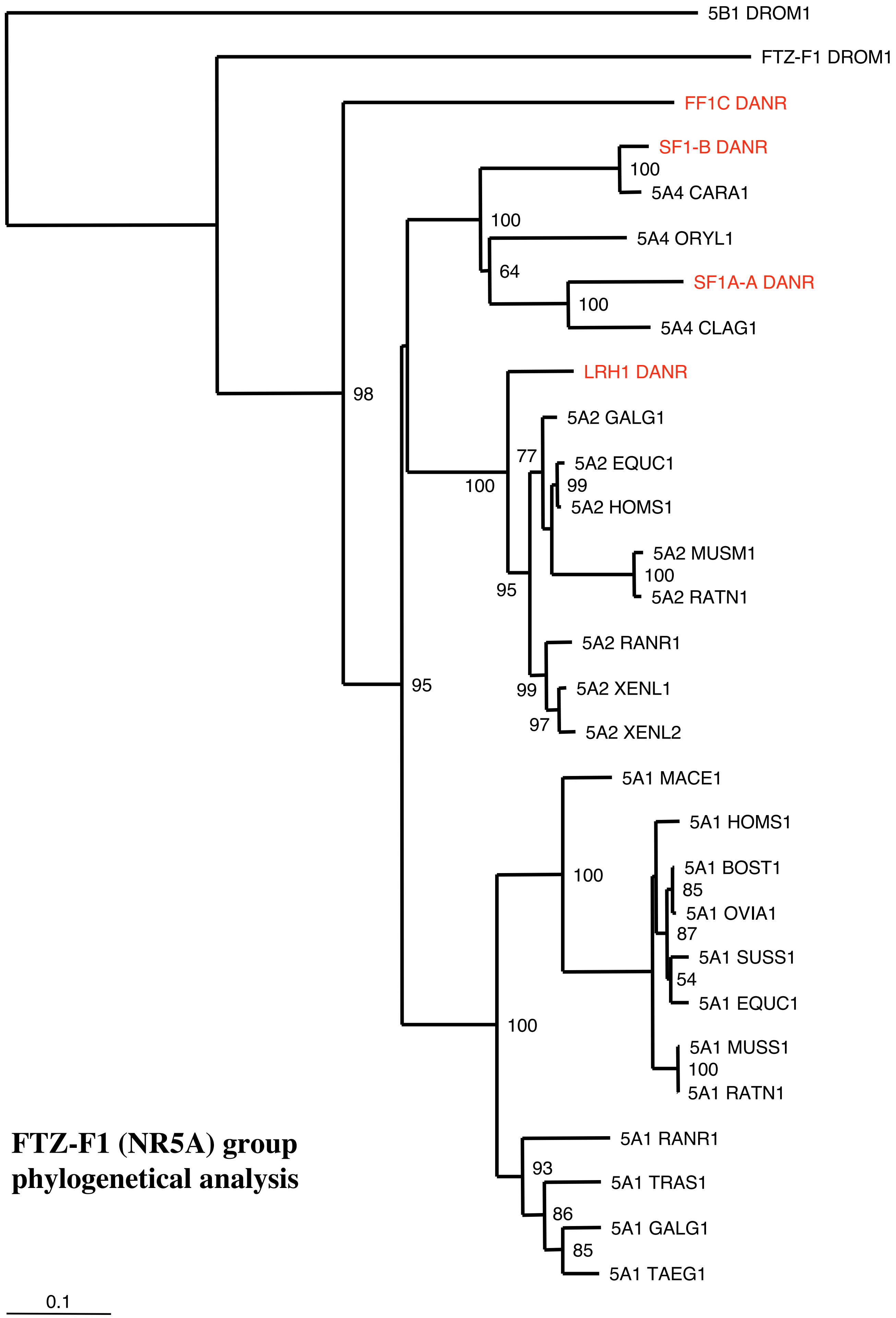

FTZ-F1 (NR5A) group phylogenetical analysis

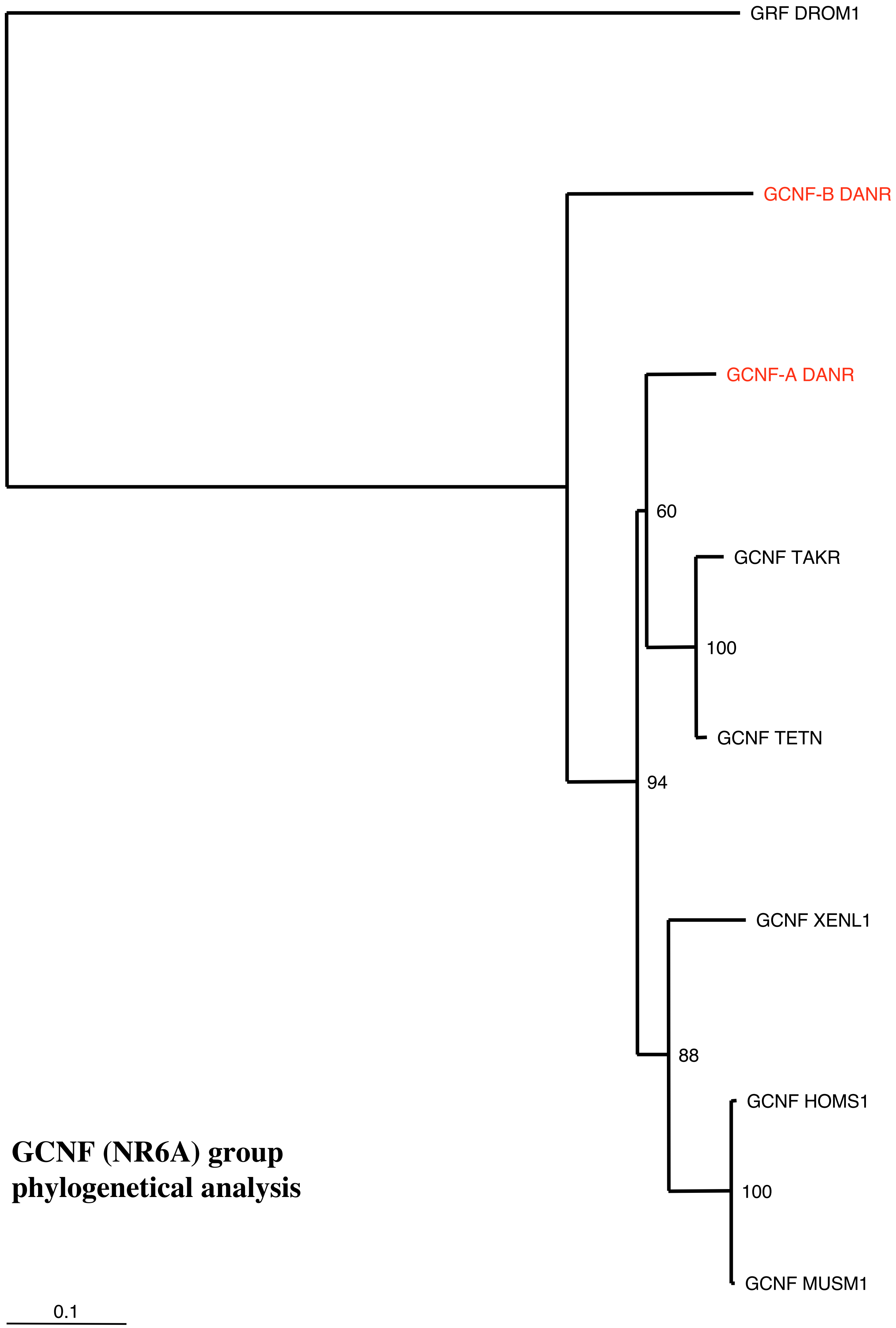

GCNF (NR6A) group phylogenetical analysis

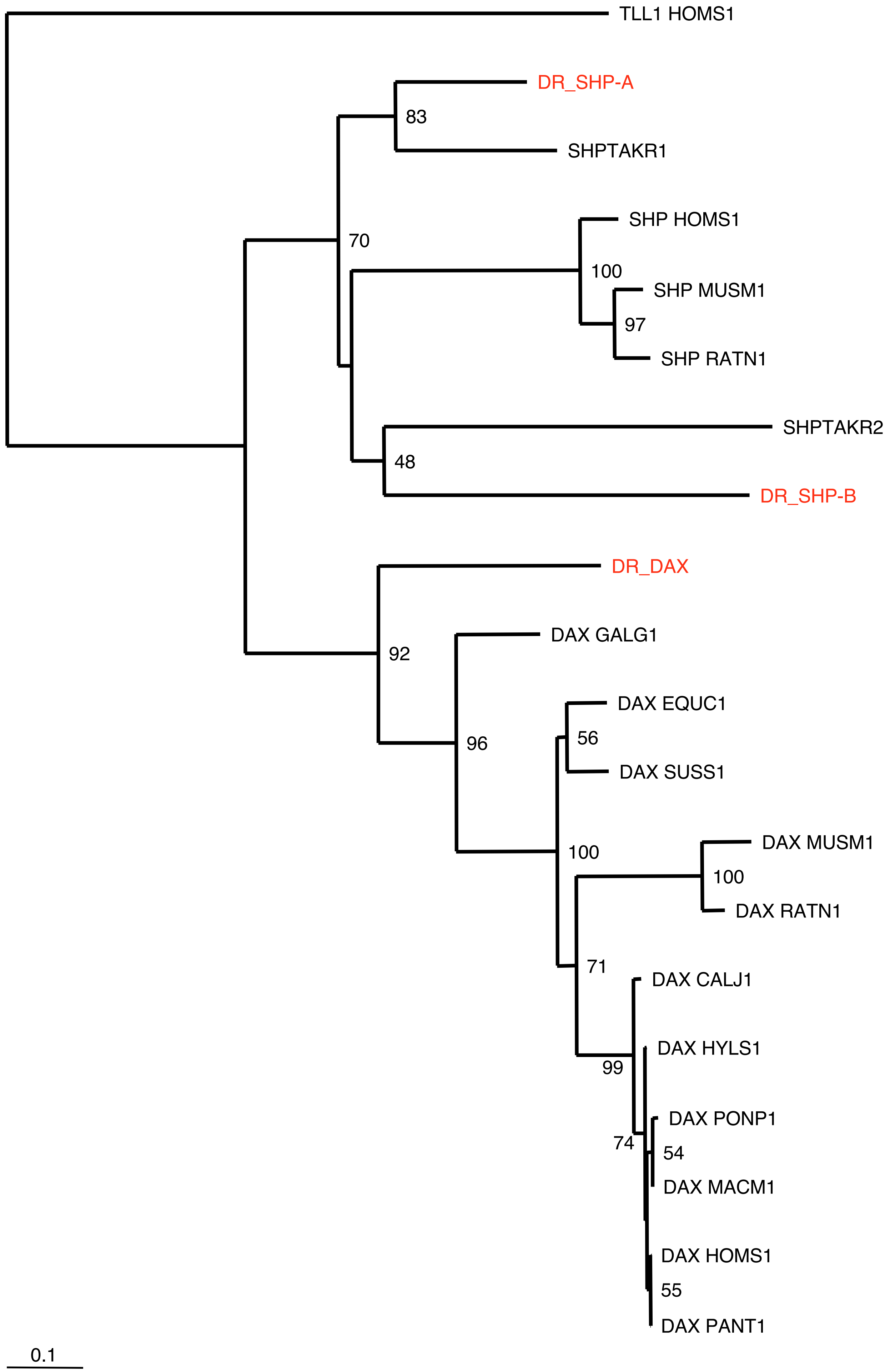

TLL1 HOMS1
DR_SHP-A
83
SHPTAKR1
SHP HOMS1
70
100
SHP MUSM1
97
SHP RATN1
SHPTAKR2
48
DR_SHP-B
DR_DAX
DAX GALG1
92
DAX EQUC1
96
56
DAX SUSS1
DAX MUSM1
100
100
DAX RATN1
71
DAX CALJ1
DAX HYLS1
99
DAX PONP1
74
54
DAX MACM1
DAX HOMS1
55
DAX PANT1
0.1

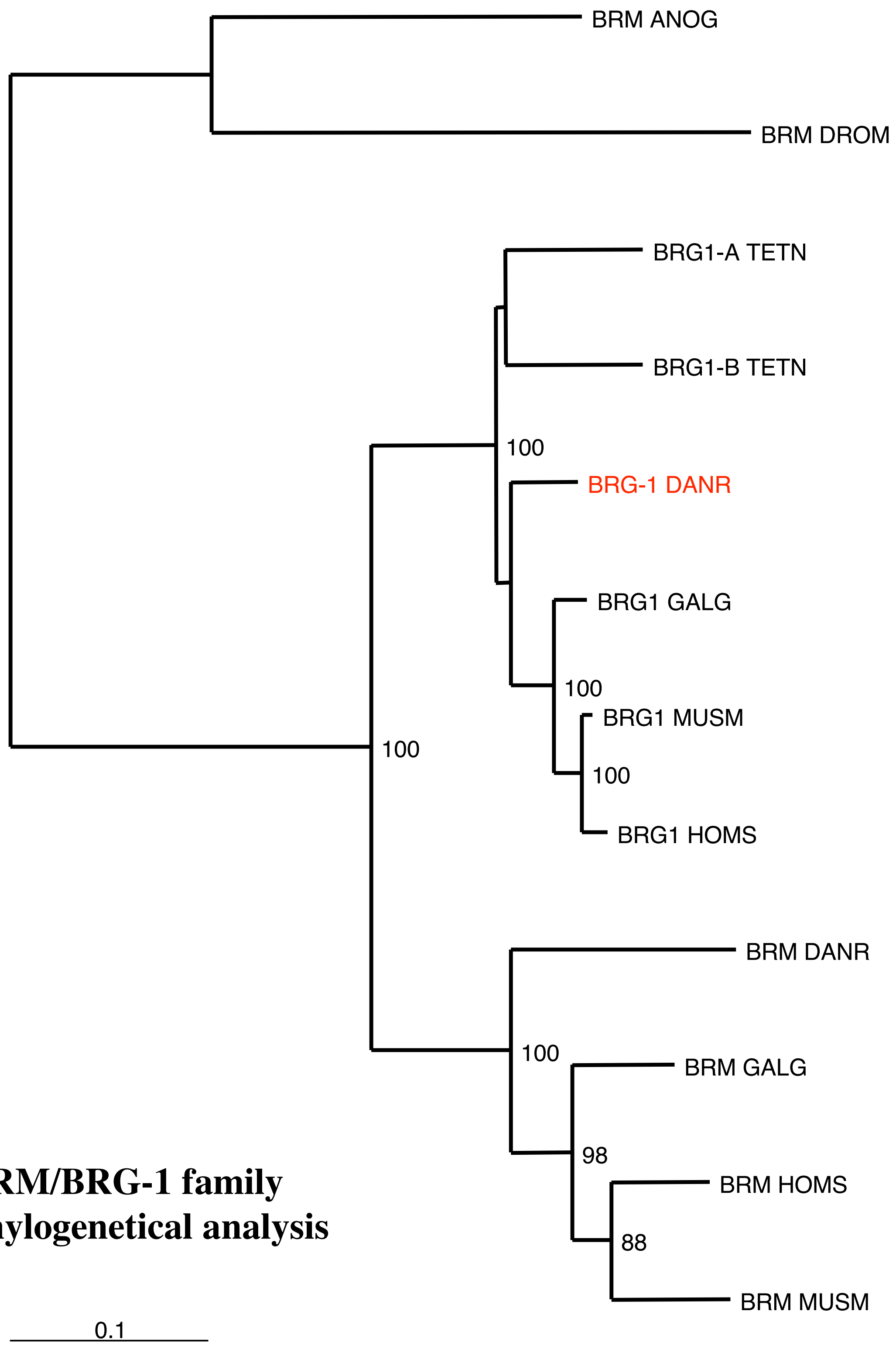

BRM/BRG-1 family phylogenetical analysis

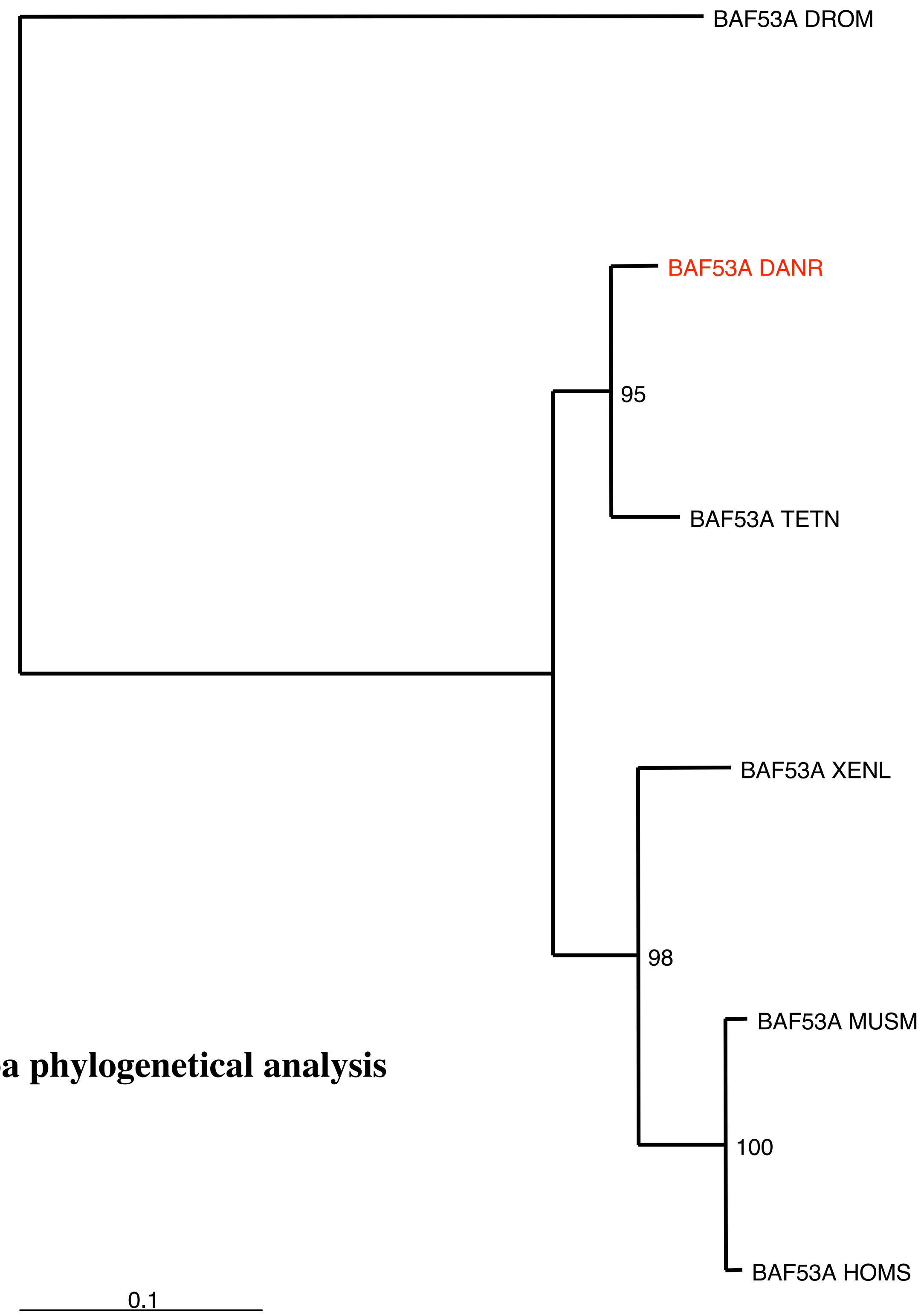

Baf53a phylogenetical analysis

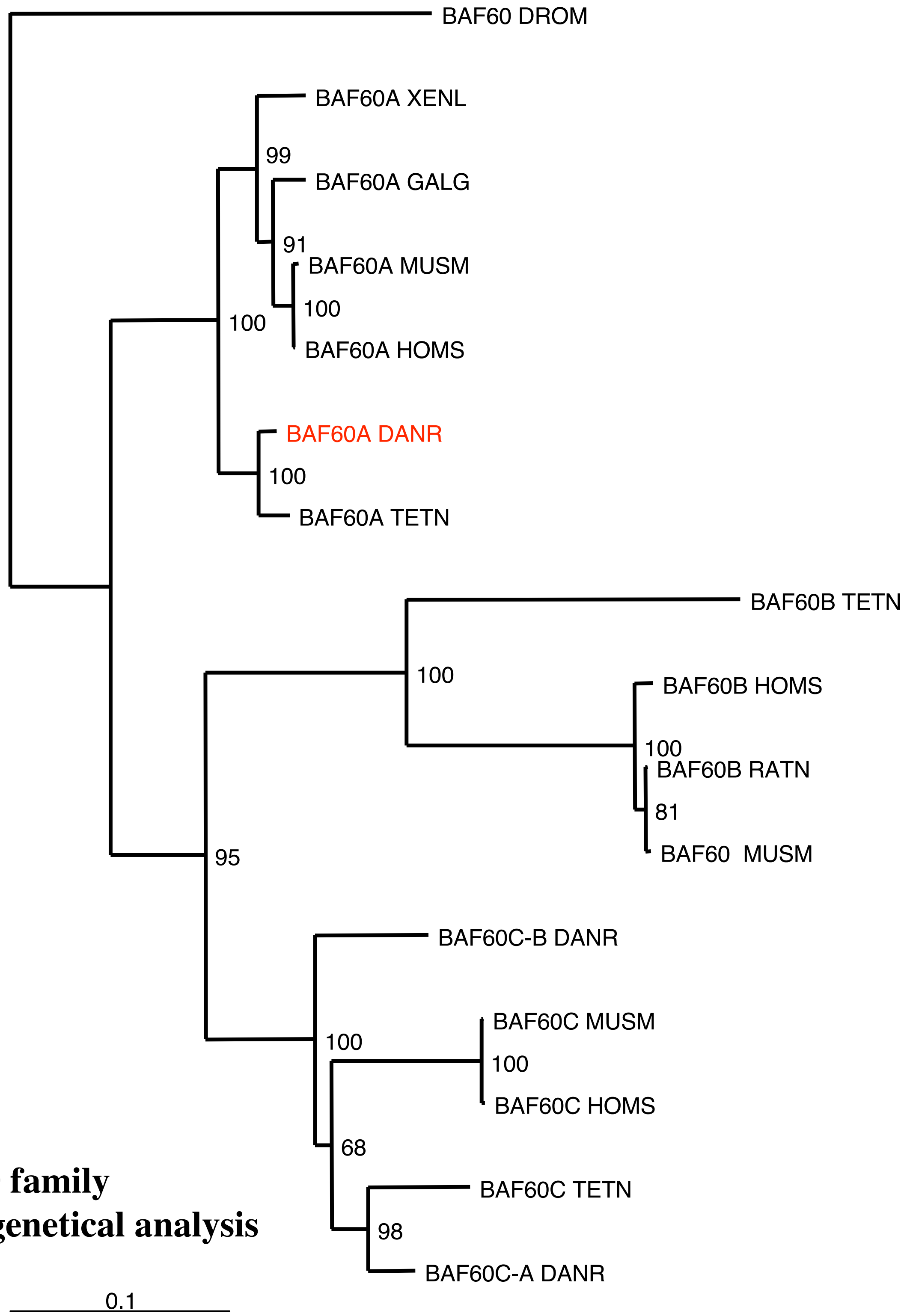

Baf 60 family phylogenetical analysis

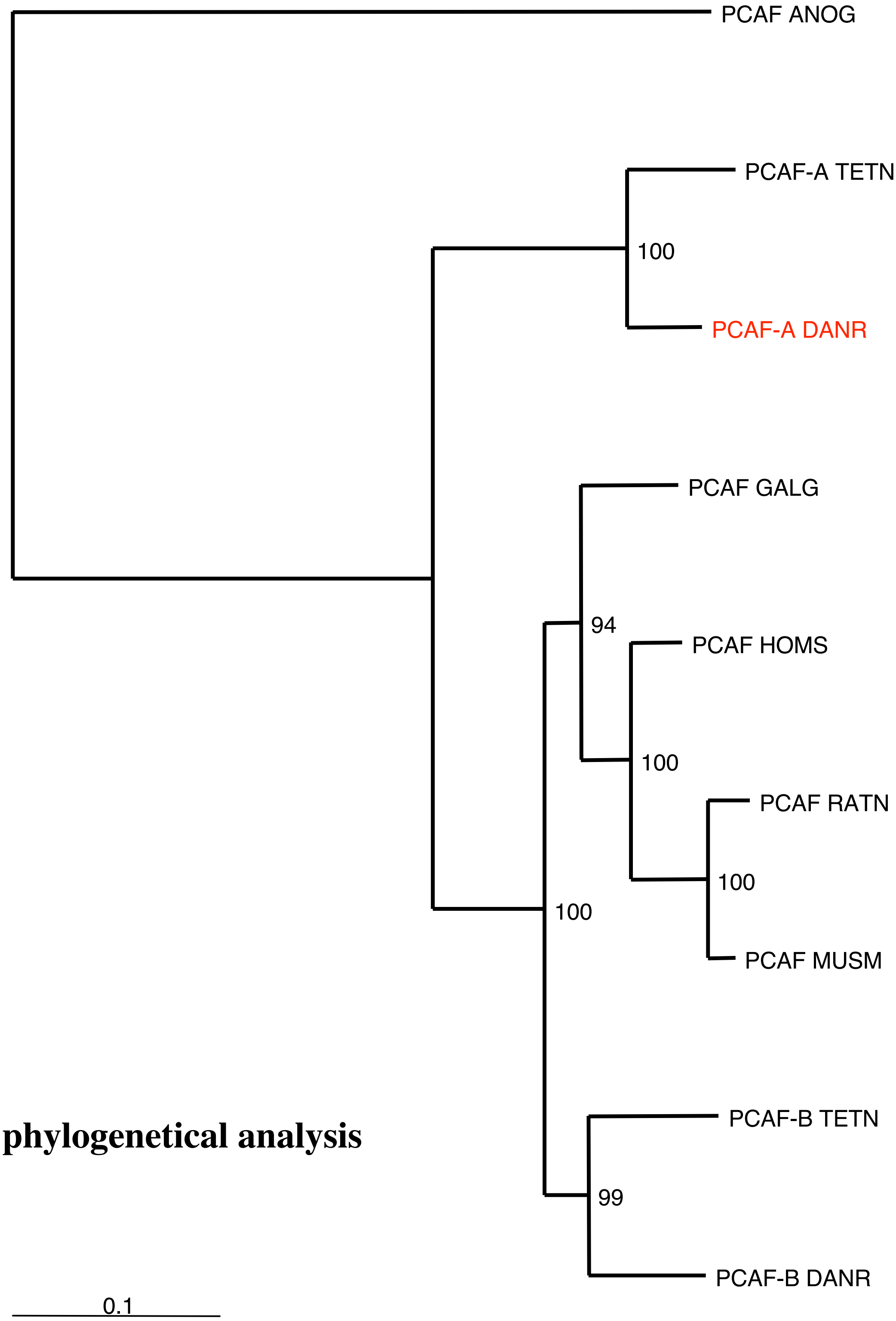

PCAF phylogenetical analysis

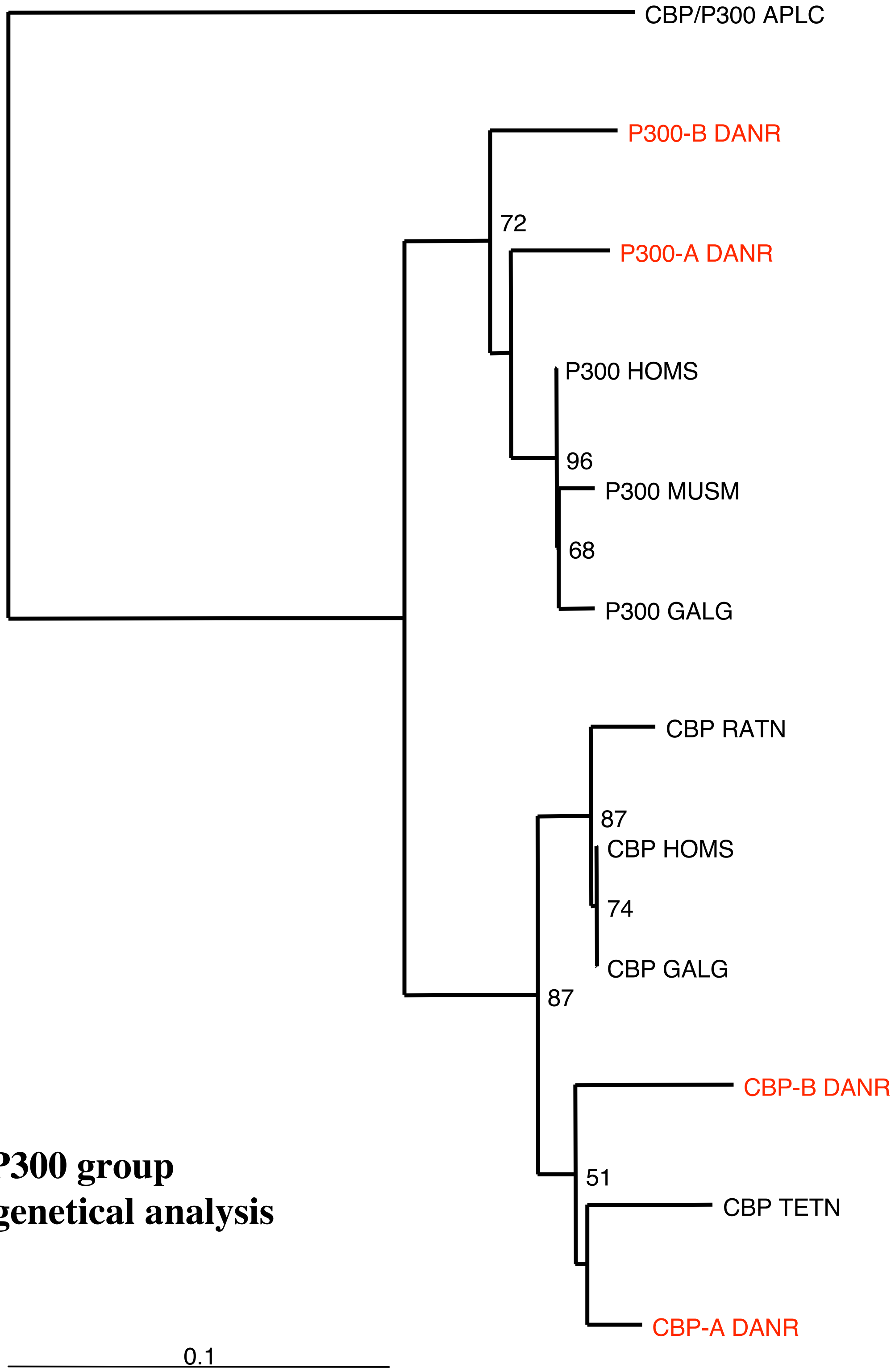

CBP/P300 group phylogenetical analysis

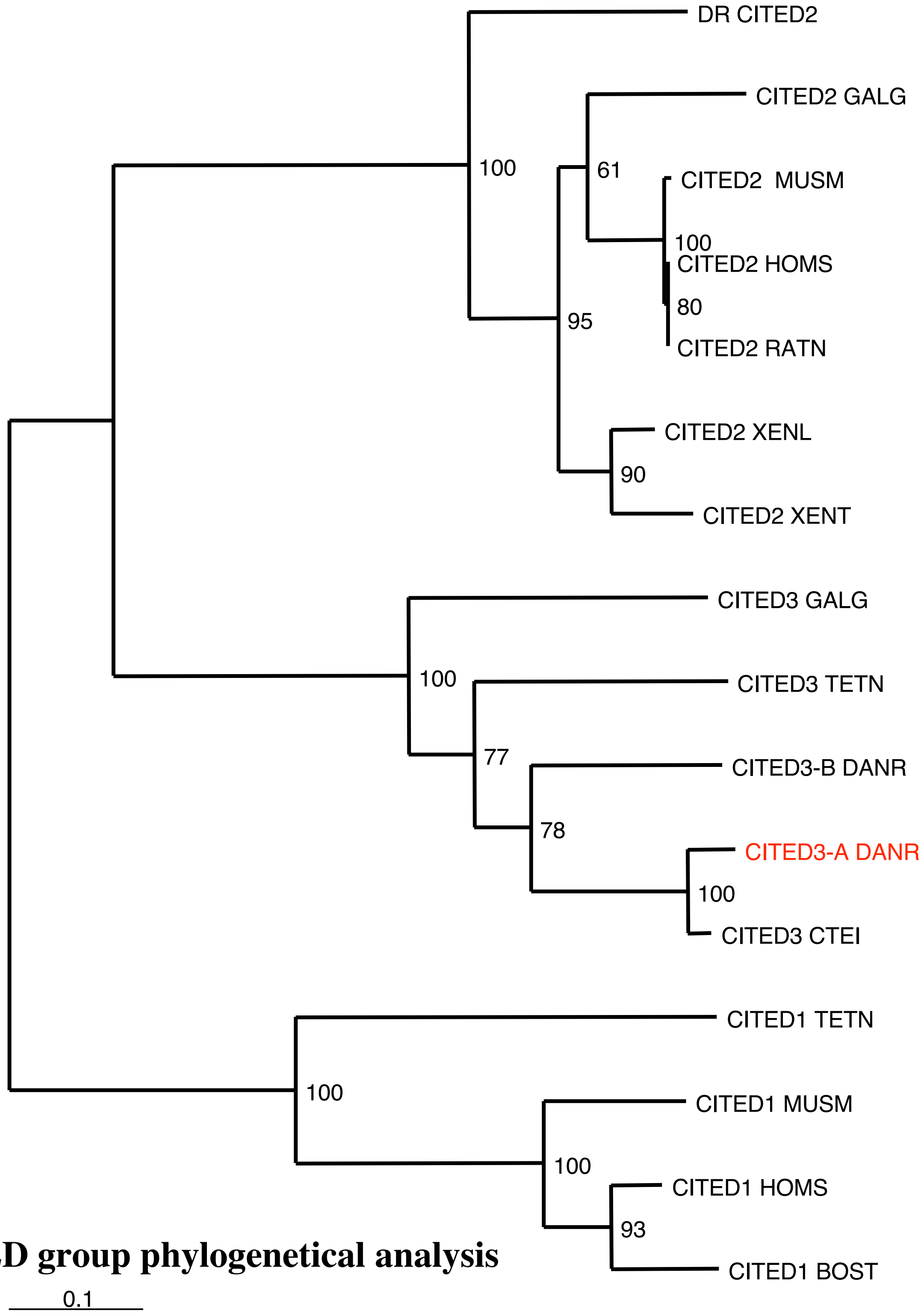

CITED group phylogenetical analysis

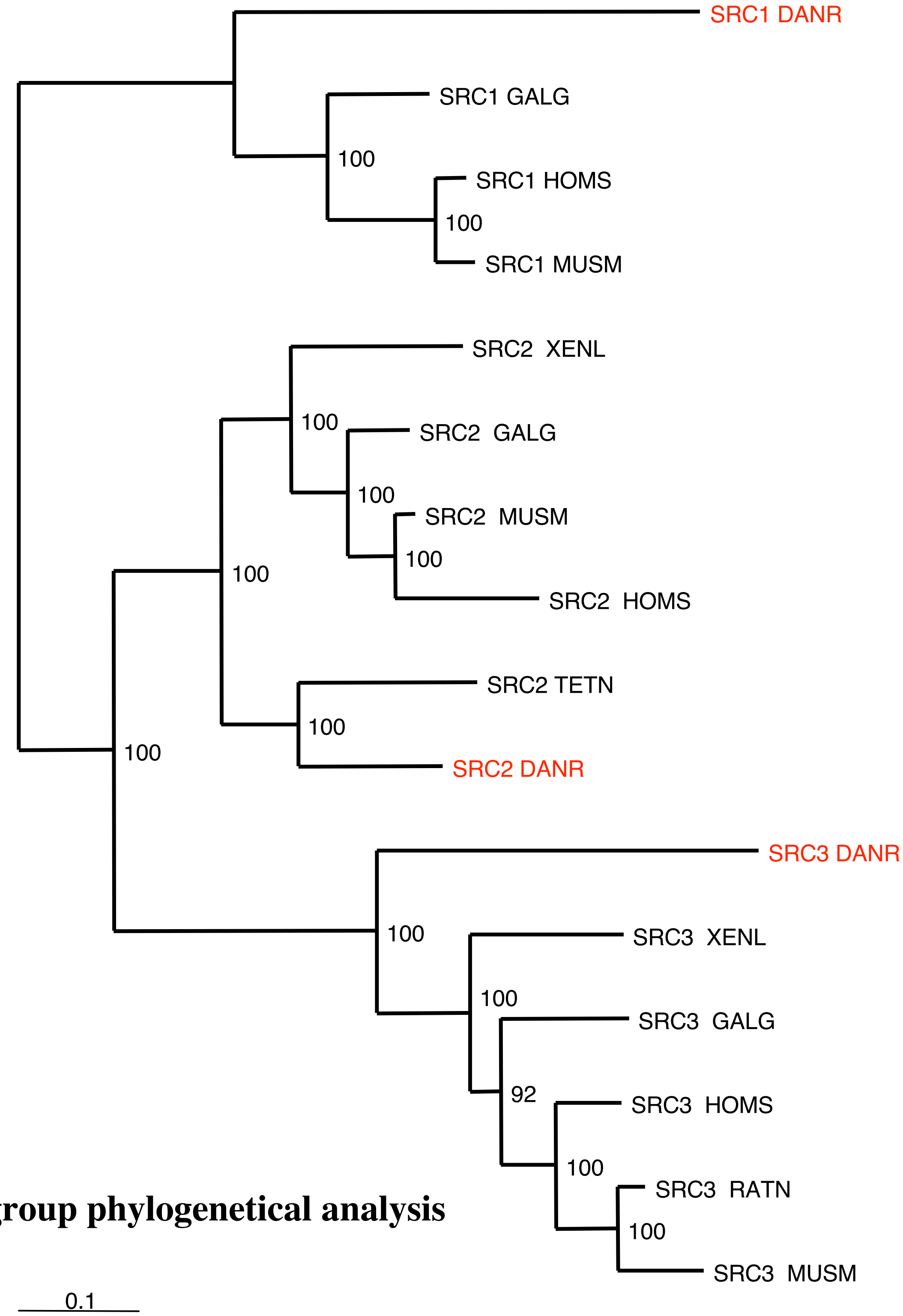

SRC group phylogenetical analysis

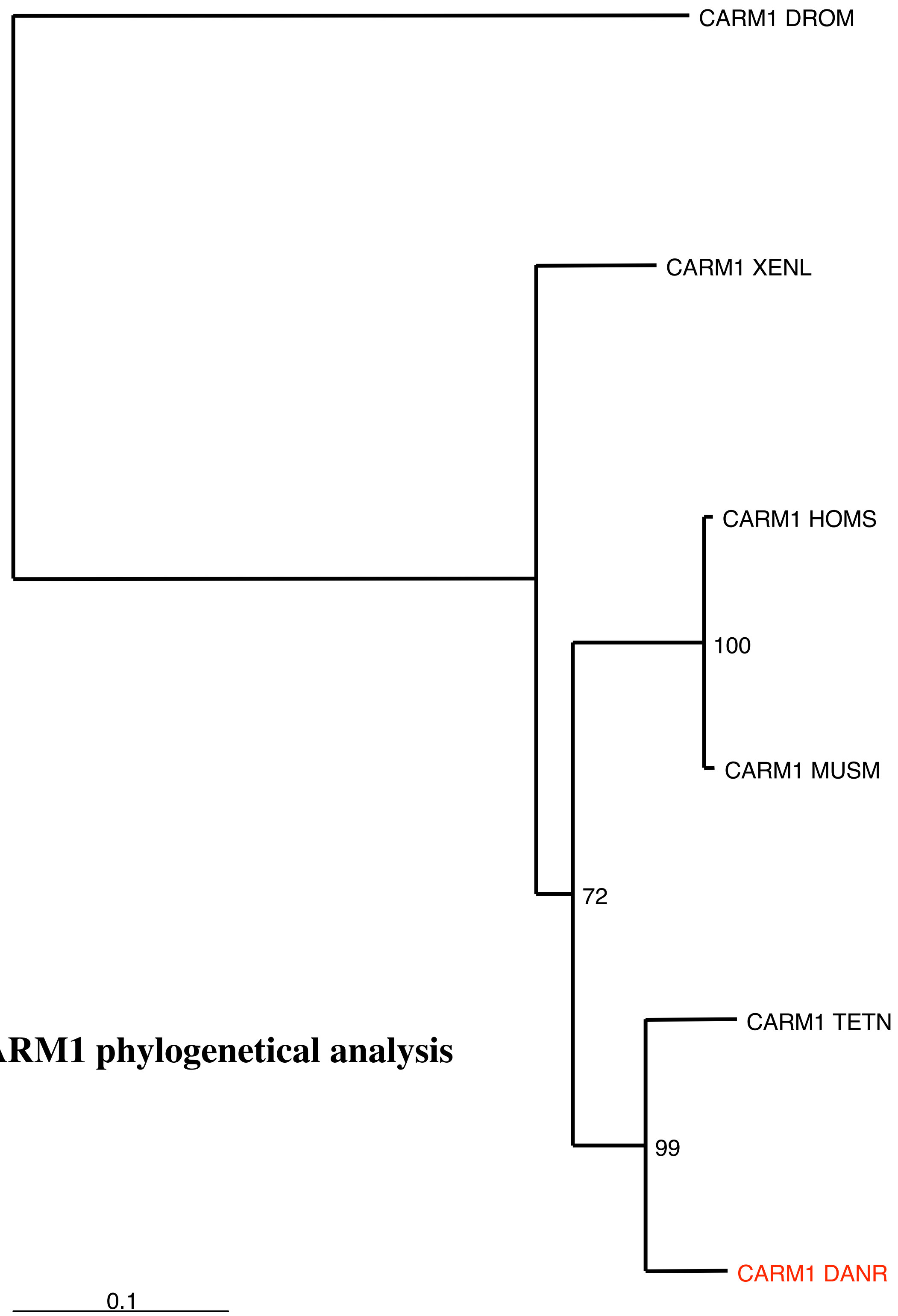

CARM1 phylogenetical analysis

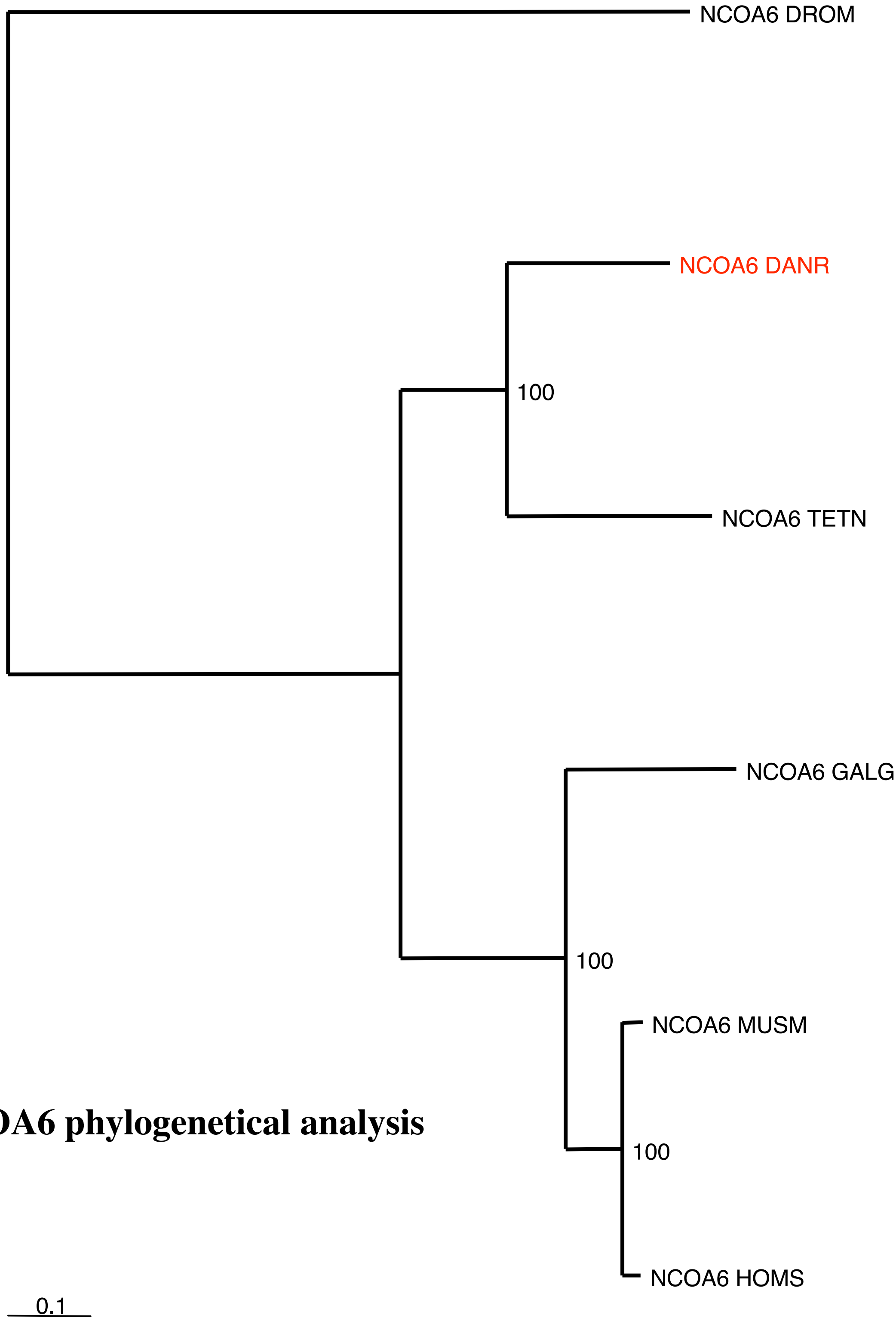

NCOA6 phylogenetical analysis

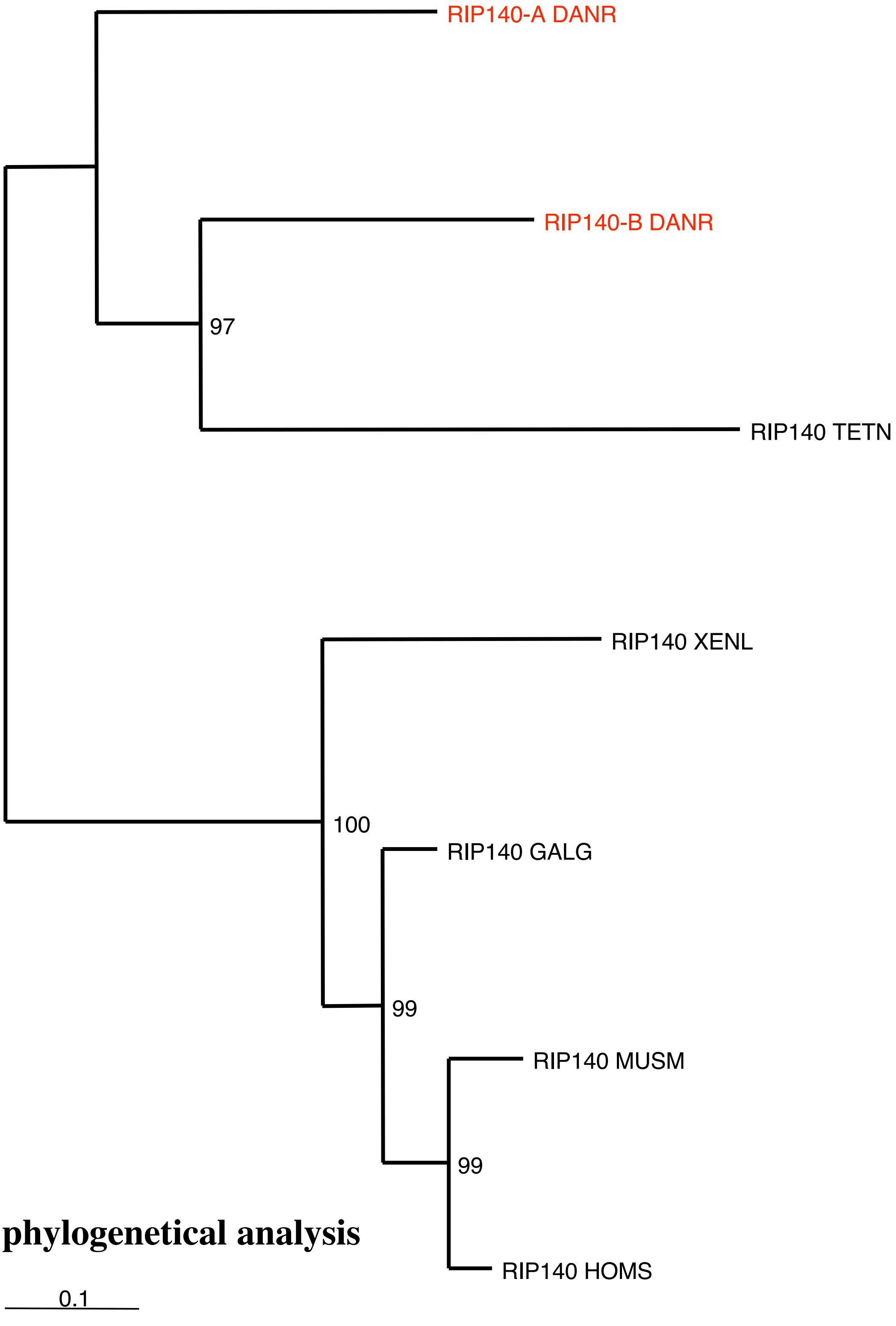

RIP140 phylogenetical analysis

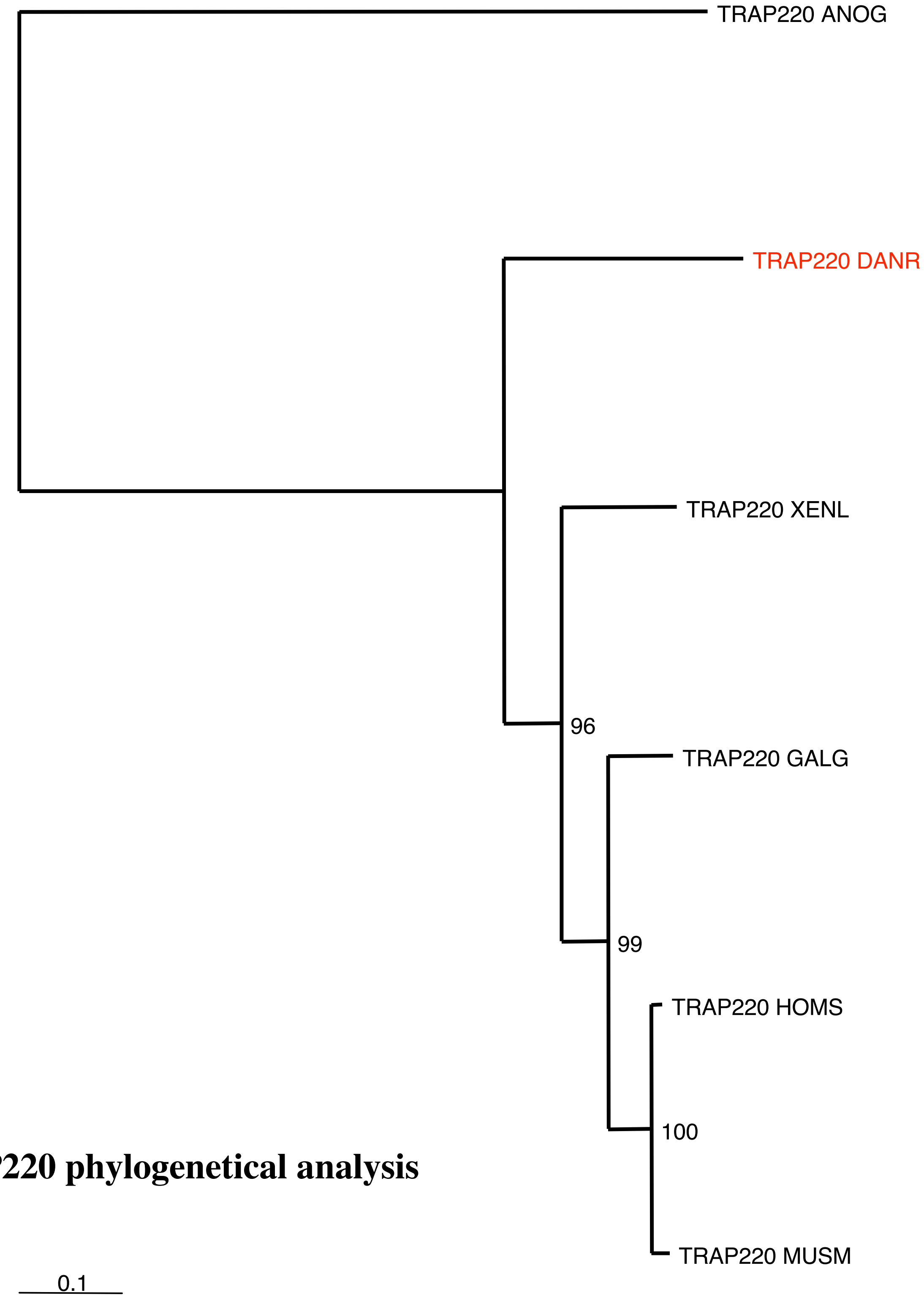

TRAP220 phylogenetical analysis

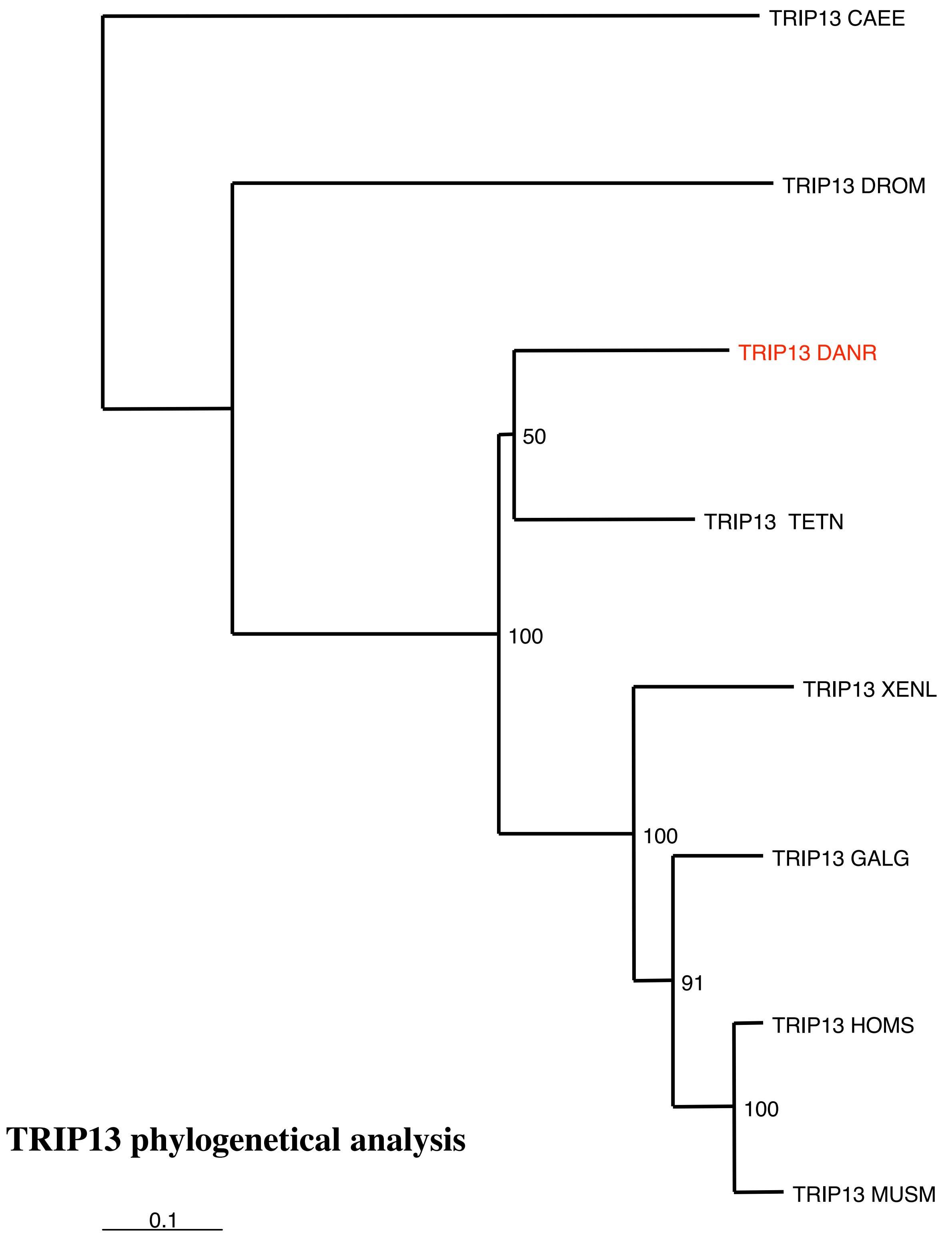

TRIP13 phylogenetical analysis

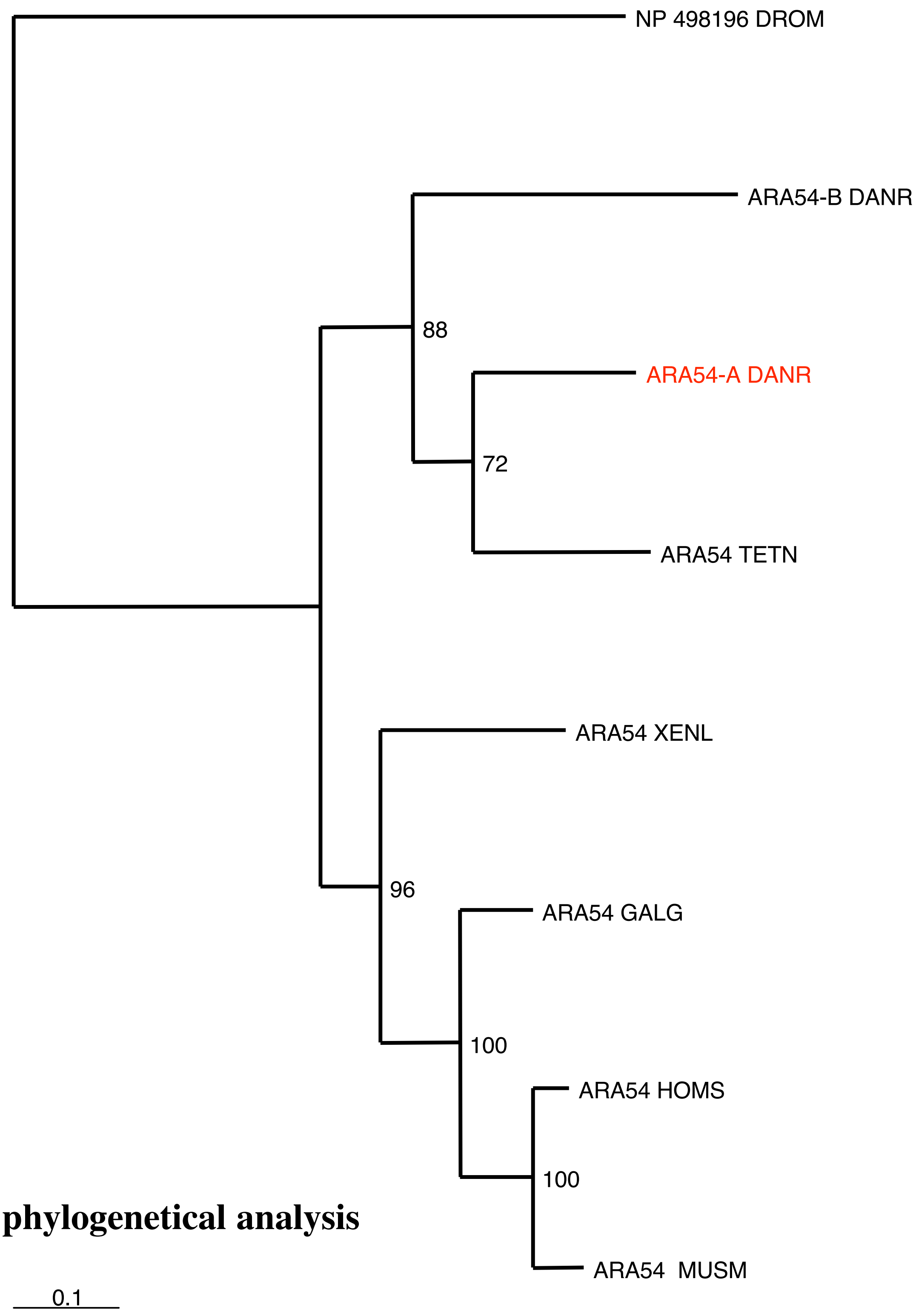

ARA54 phylogenetical analysis

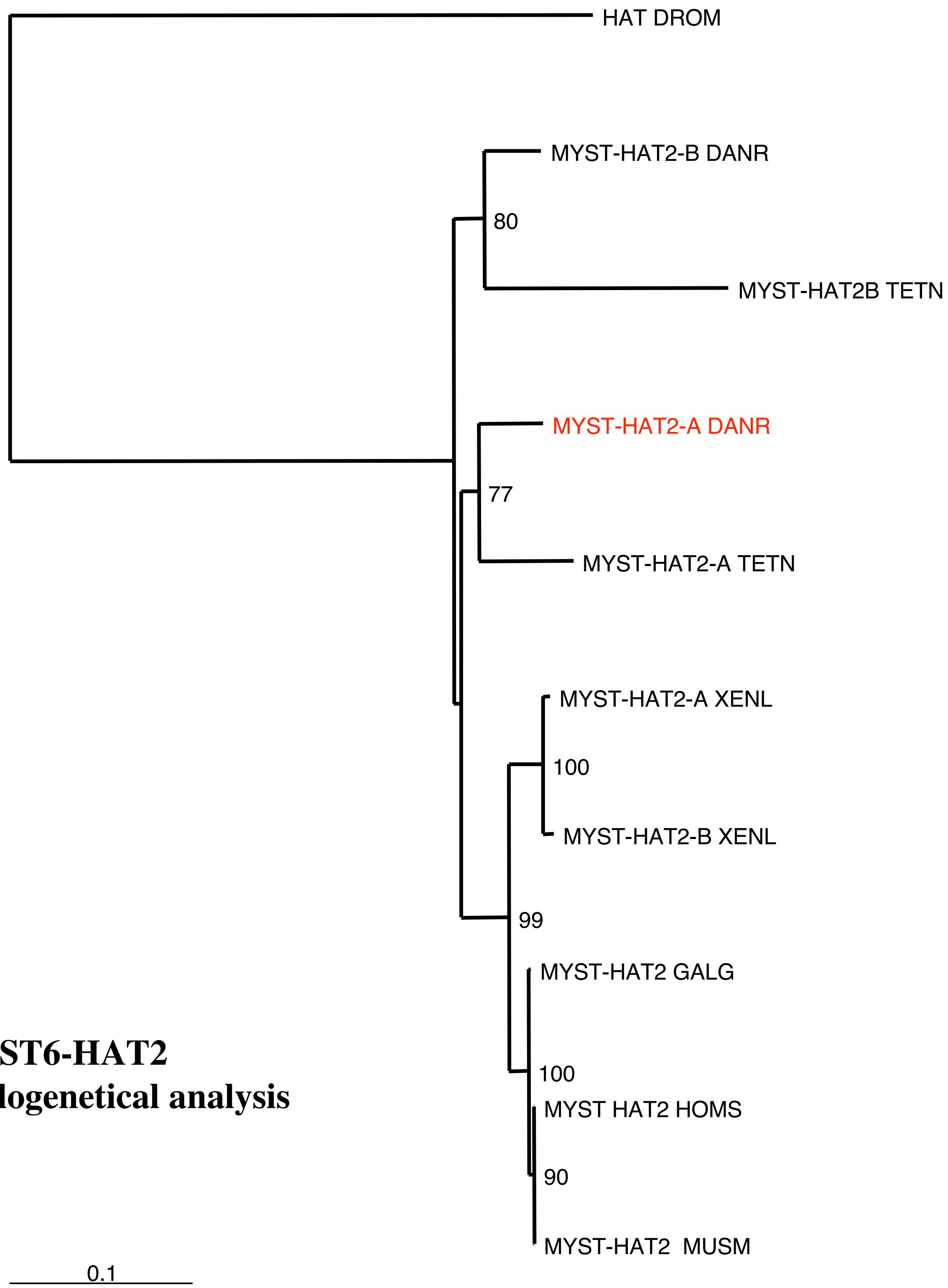

MYST6-HAT2
phylogenetical analysis

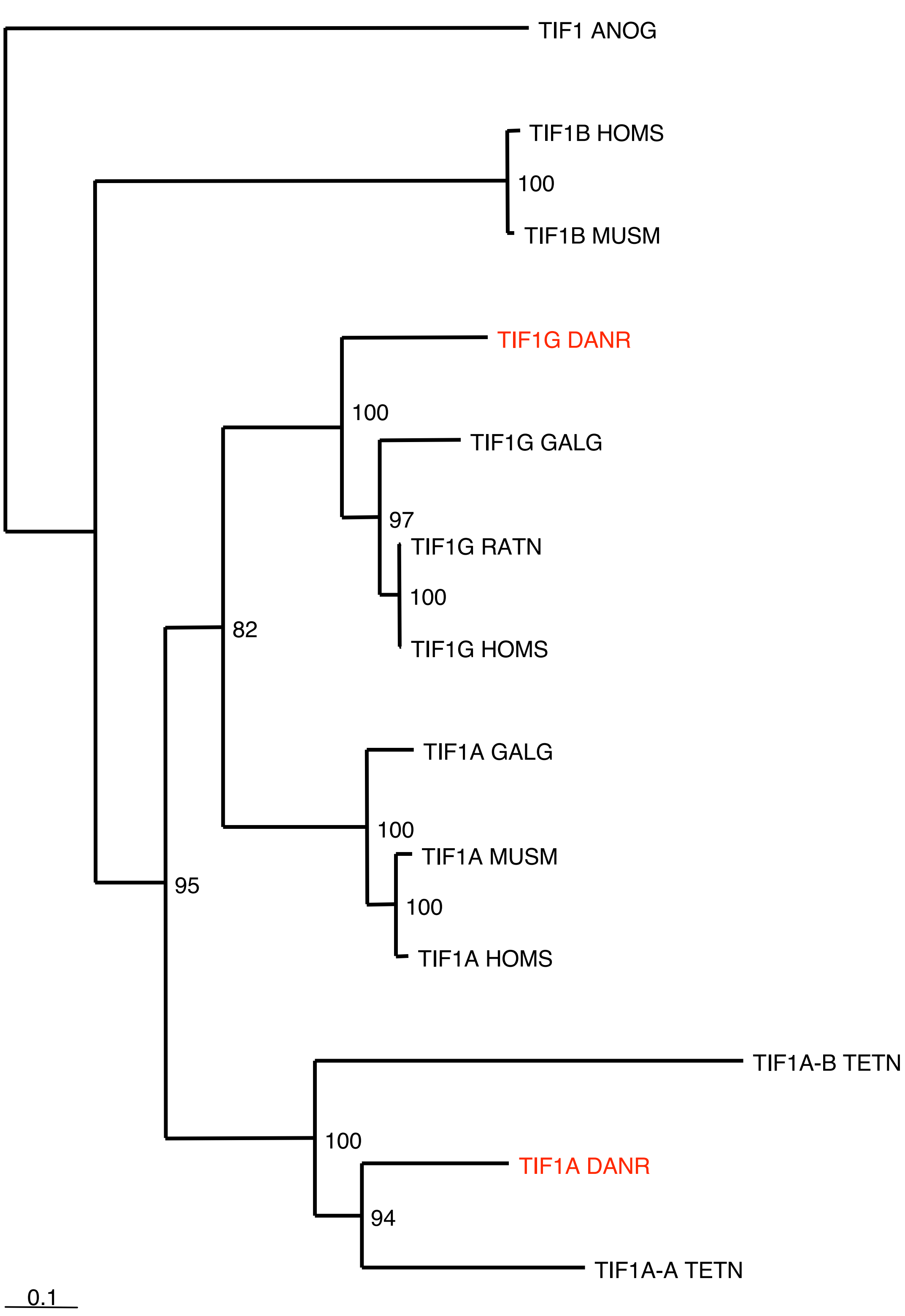

TIF1 group phylogenetical analysis

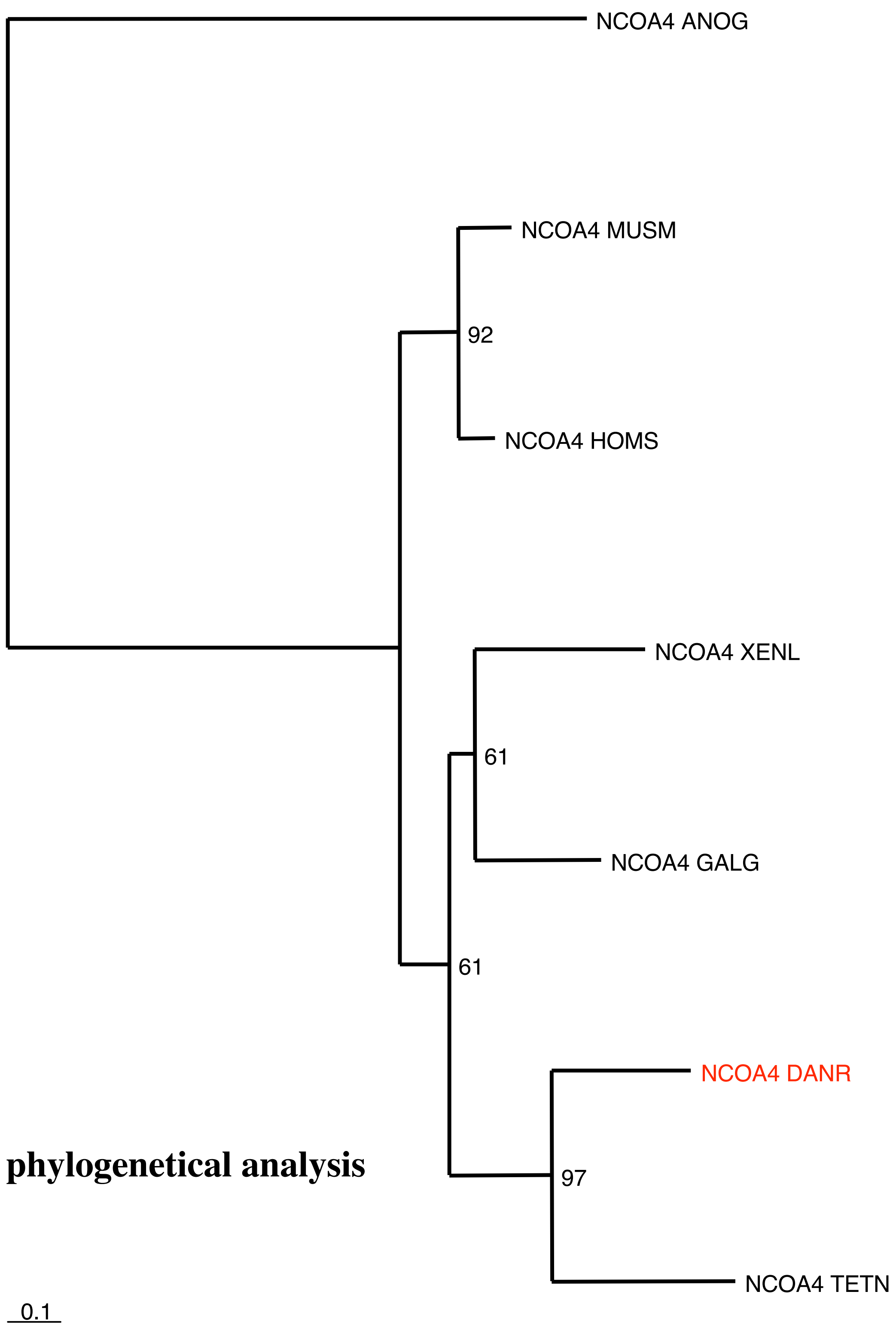

NCOA4 phylogenetical analysis

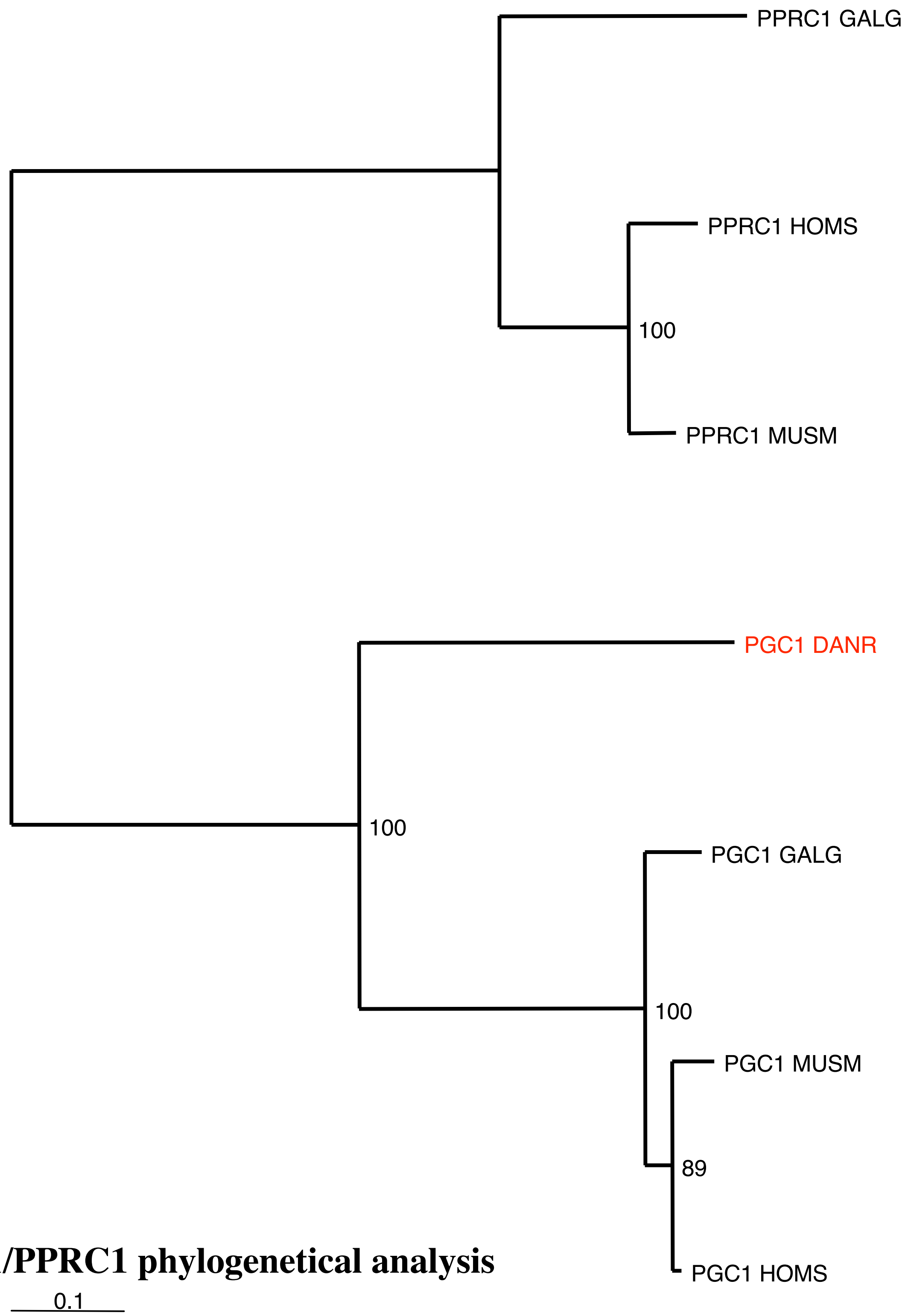

PGC1/PPRC1 phylogenetical analysis

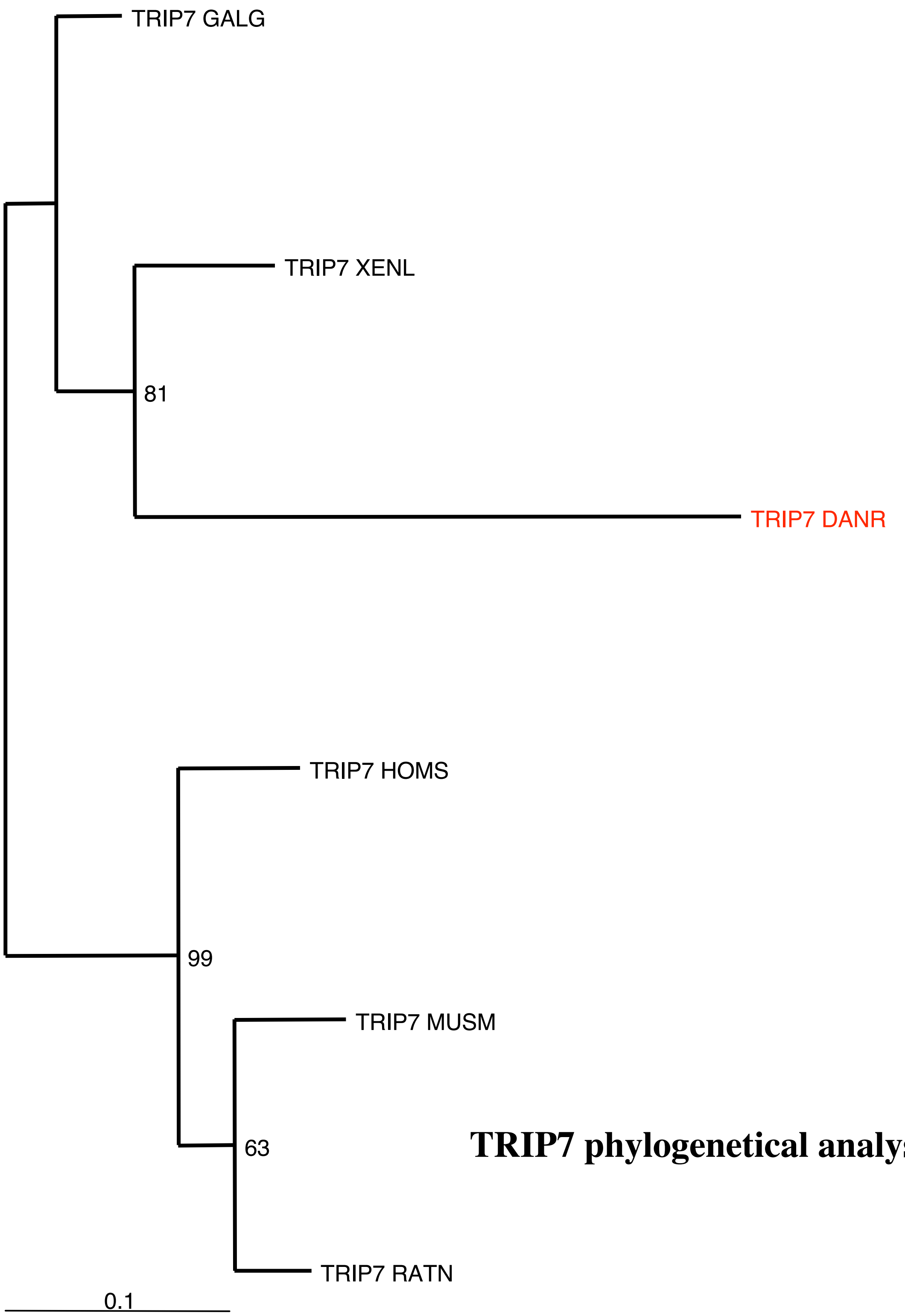

TRIP7 phylogenetical analysis

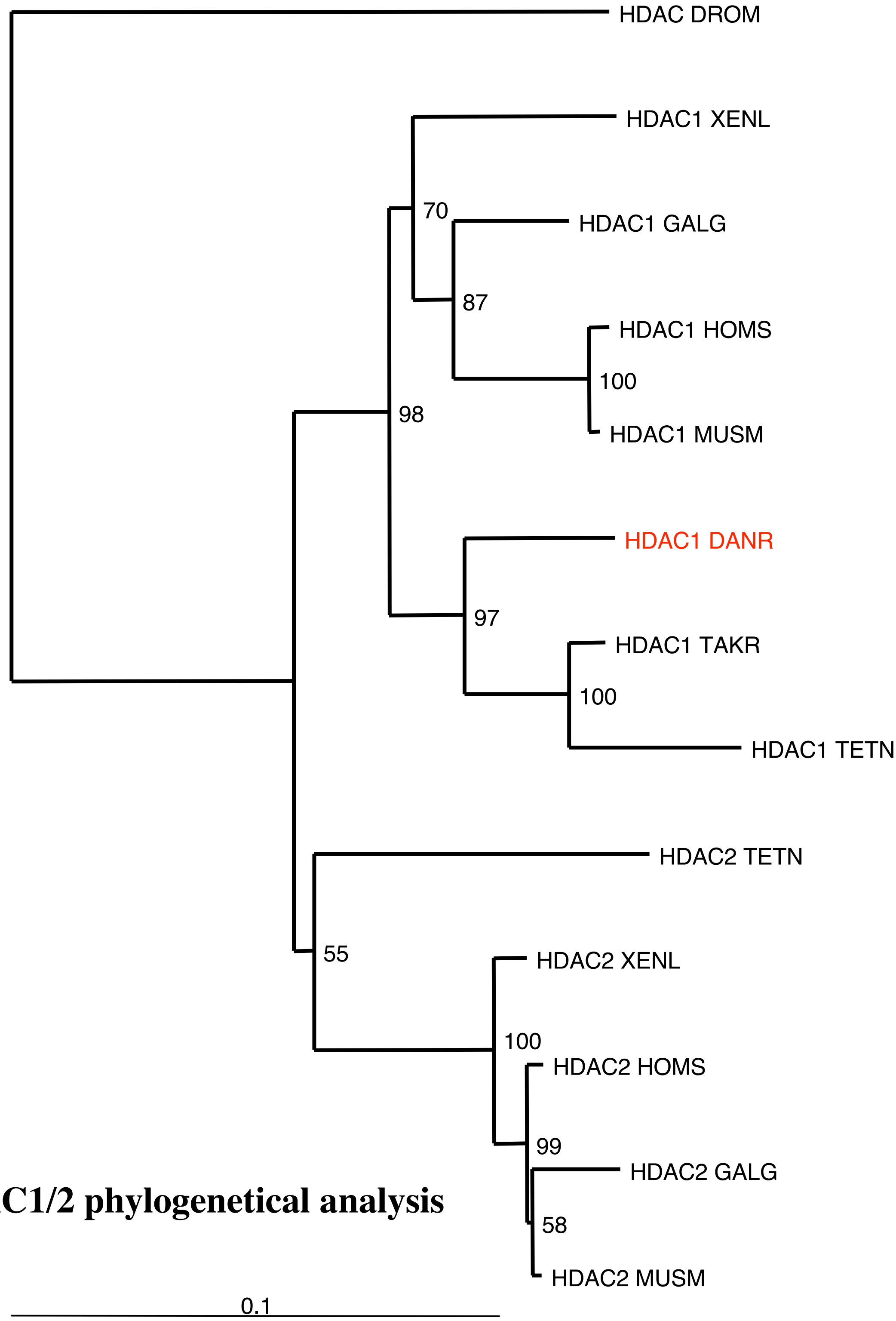

HDAC1/2 phylogenetical analysis

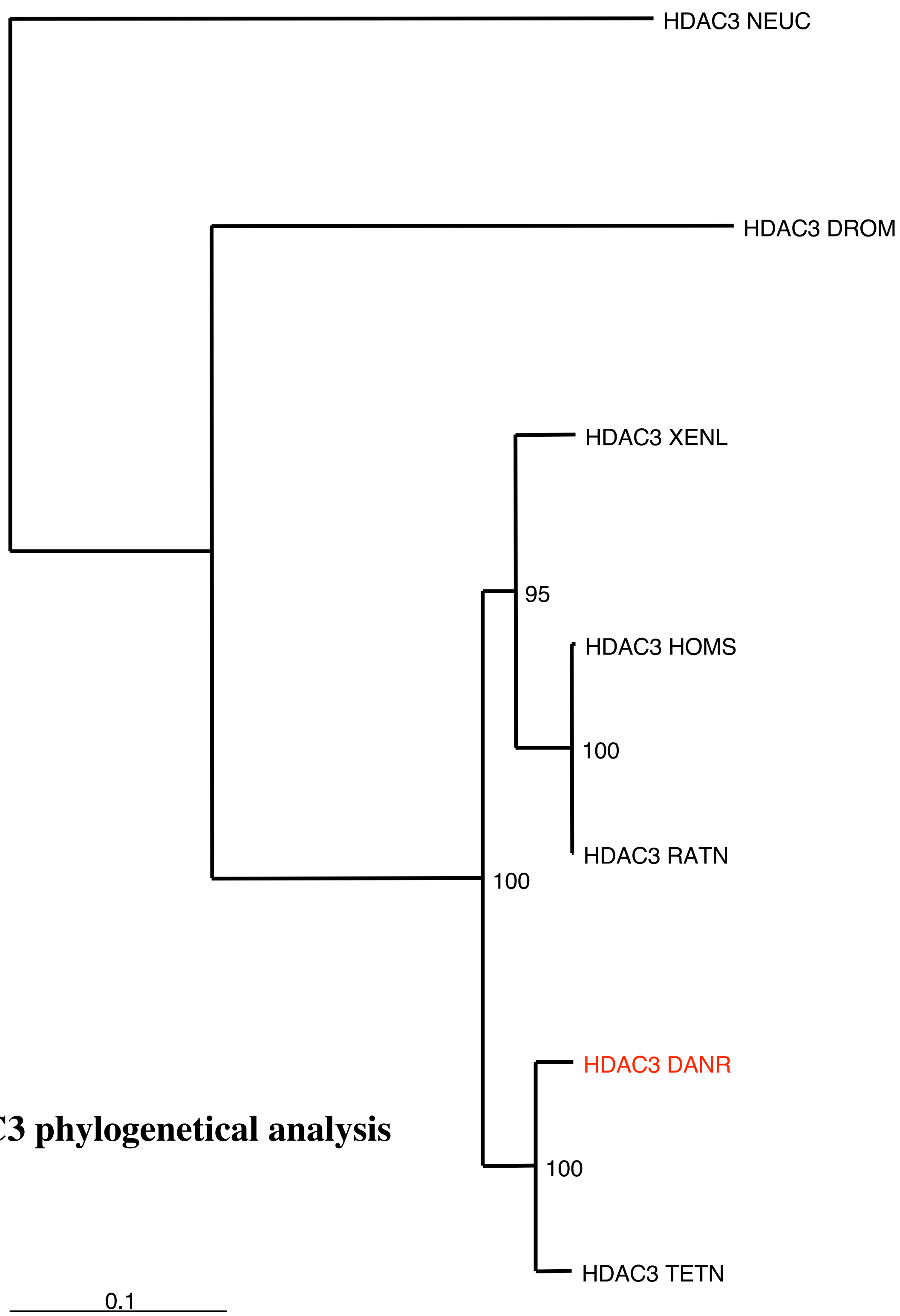

HDAC3 phylogenetical analysis

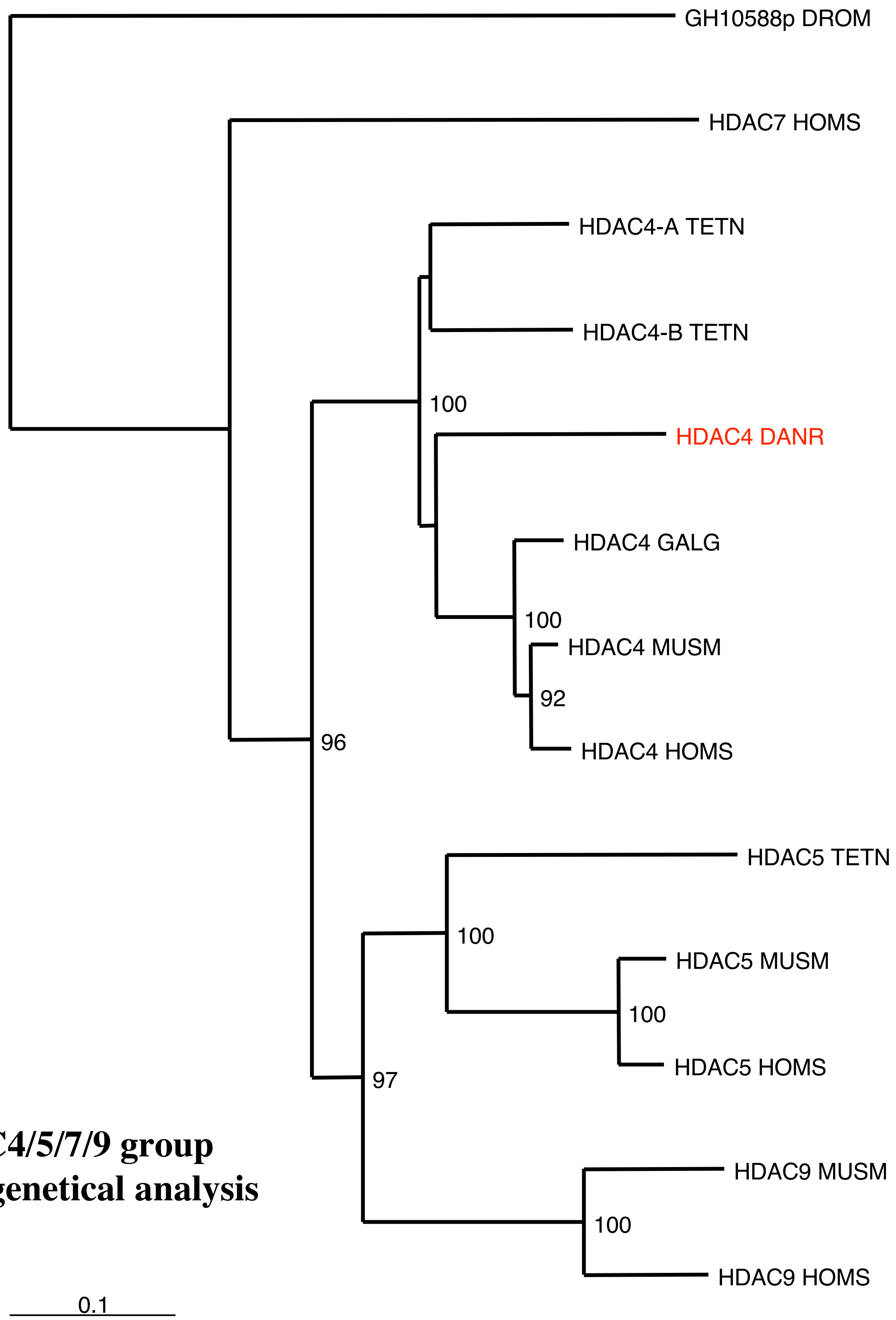

HDAC4/5/7/9 group phylogenetical analysis

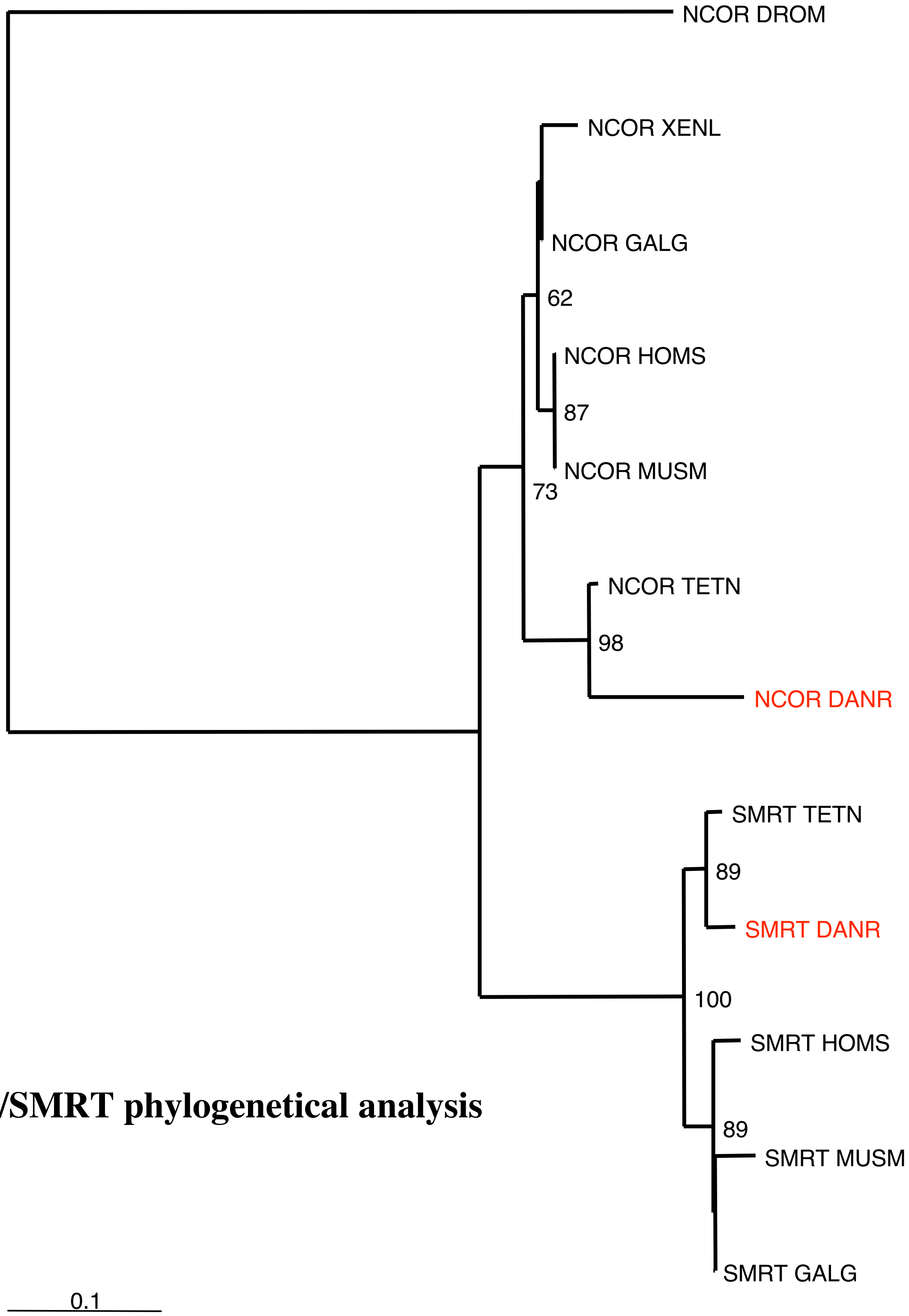

NCOR/SMRT phylogenetical analysis

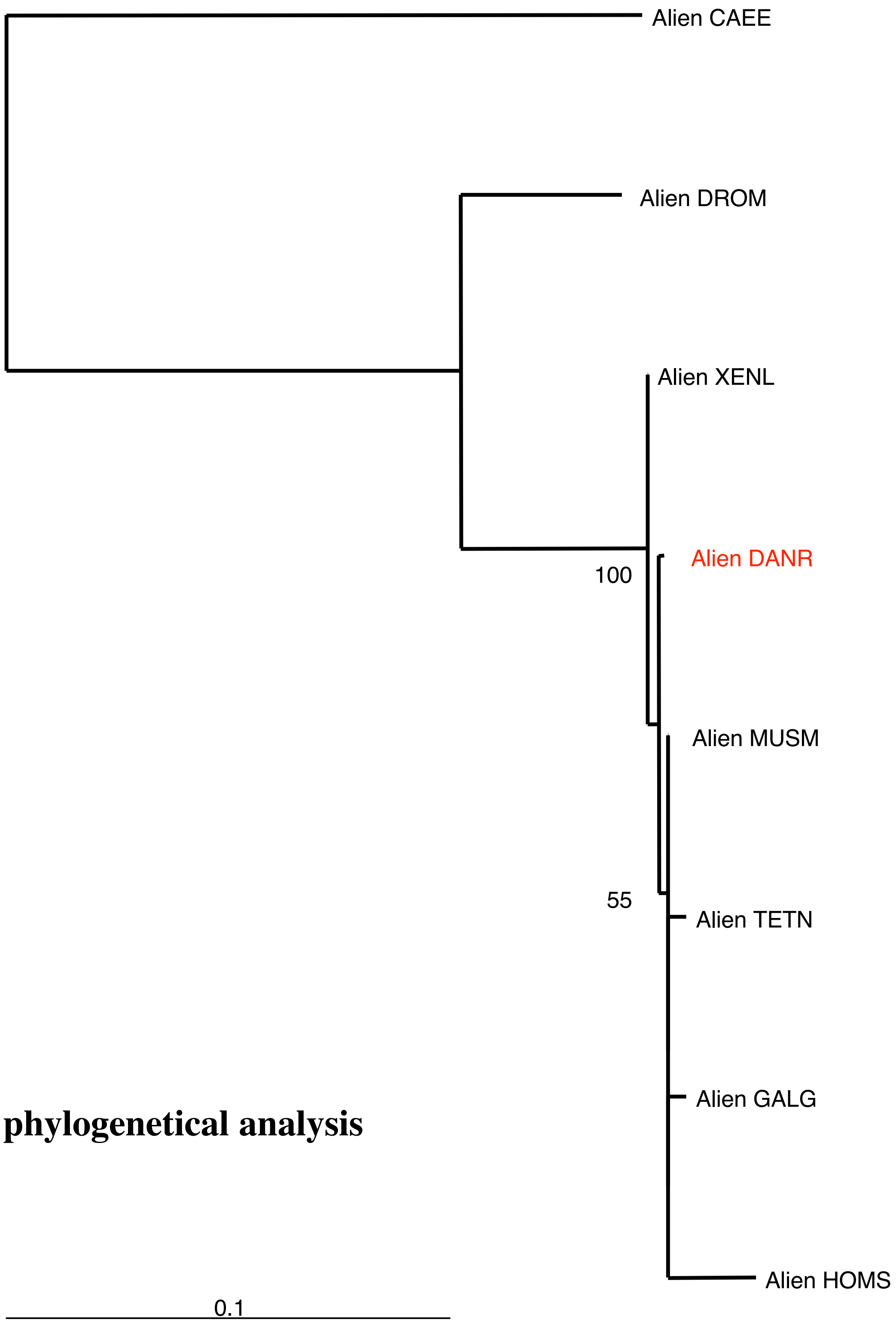

Alien phylogenetical analysis
