# Supplementary material for: Unexpected Novel Relational Links Uncovered by Extensive Developmental Profiling of Nuclear Receptor Expression
Source: PLoS Genet. 2007 Nov 9;3(11):e188. doi: 10.1371/journal.pgen.0030188 (PMC2065881; doi:10.1371/journal.pgen.0030188)
Supplement: Supplementary file 2 [file pgen.0030188.sg003.pdf]

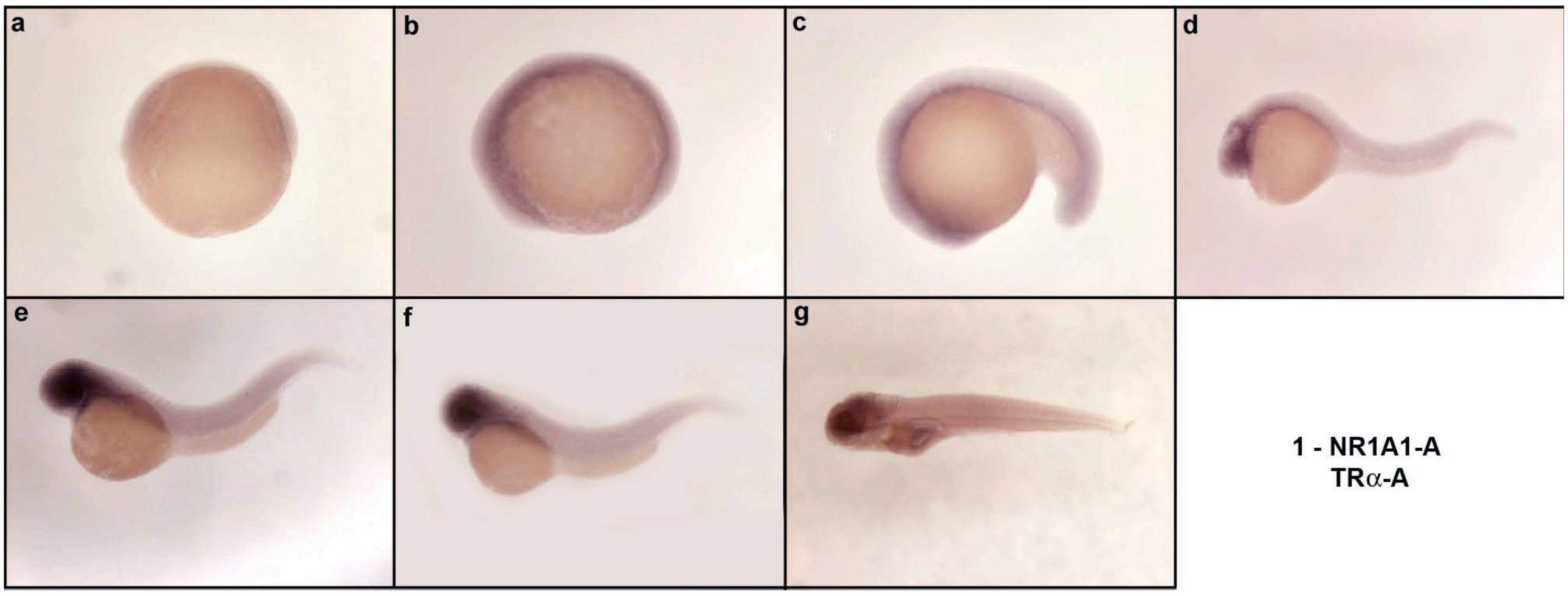

1 - NR1A1-A
TRα-A

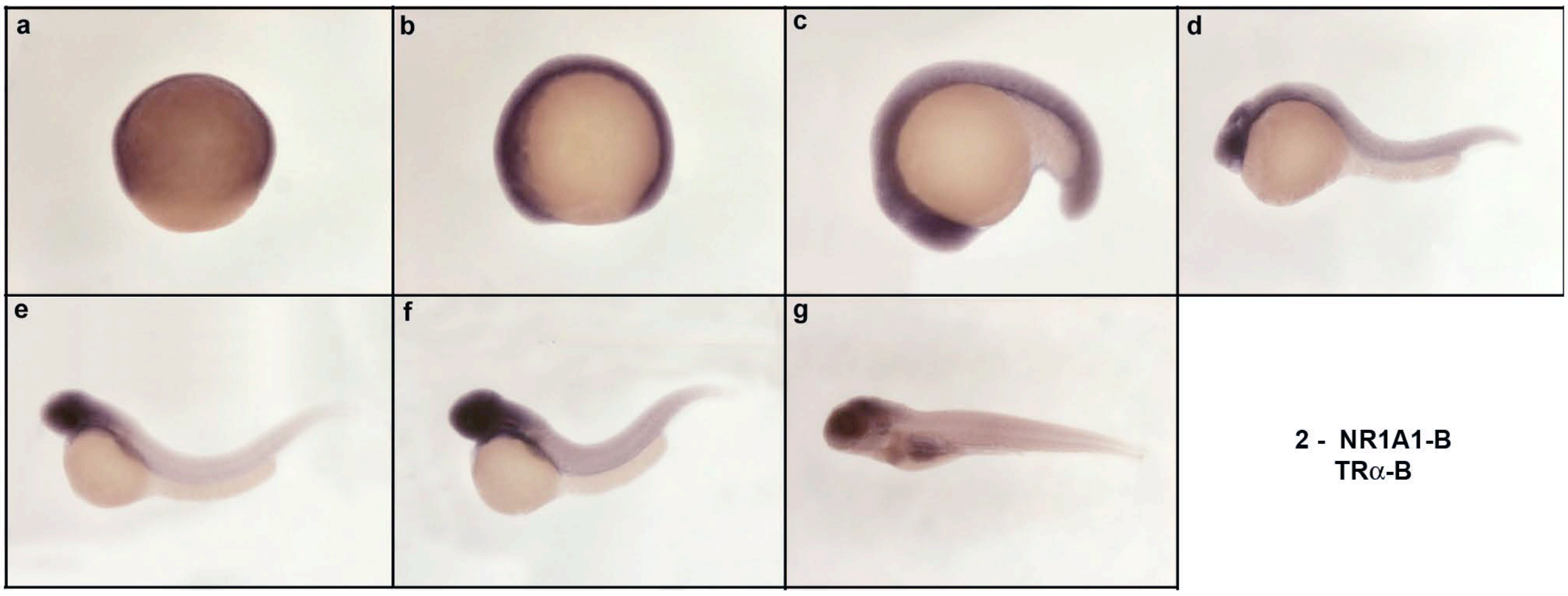

2 - NR1A1-B
TRα-B

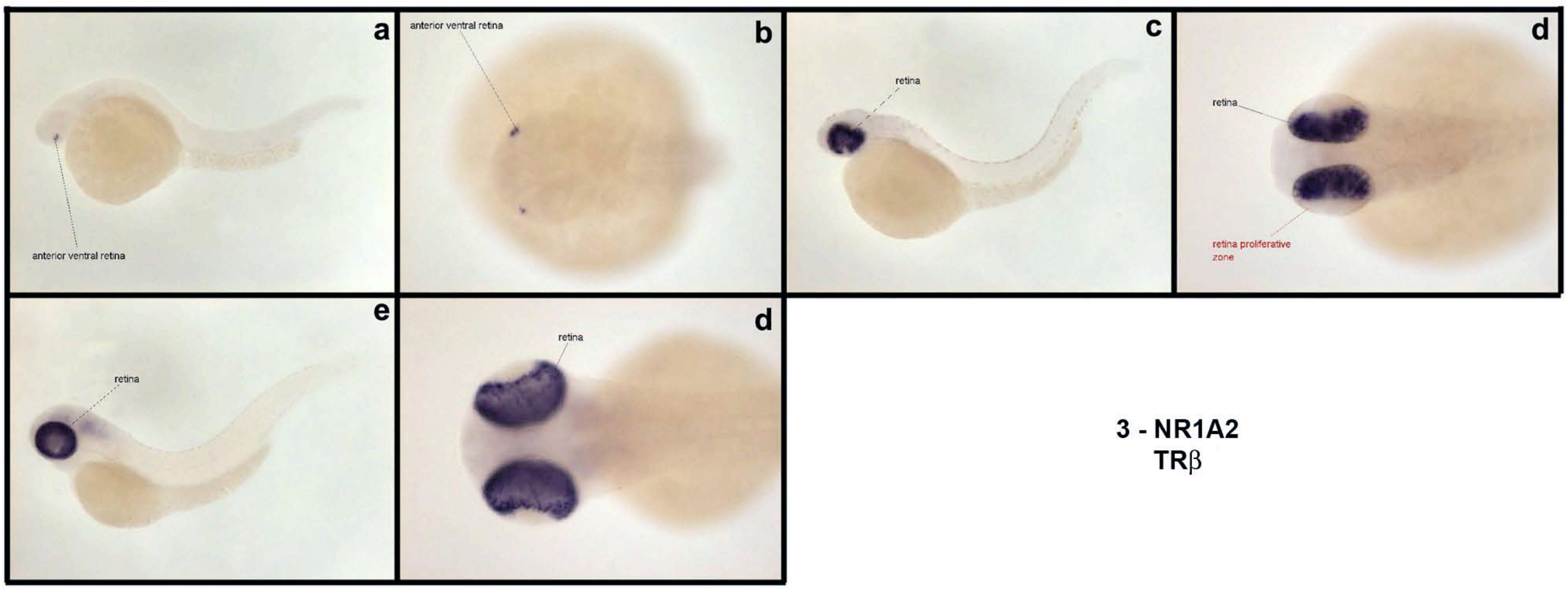
a
anterior ventral retina
b
anterior ventral retina
c
retina
d
retina
retina proliferative zone
e
retina
d
retina

3 - NR1A2
TRβ

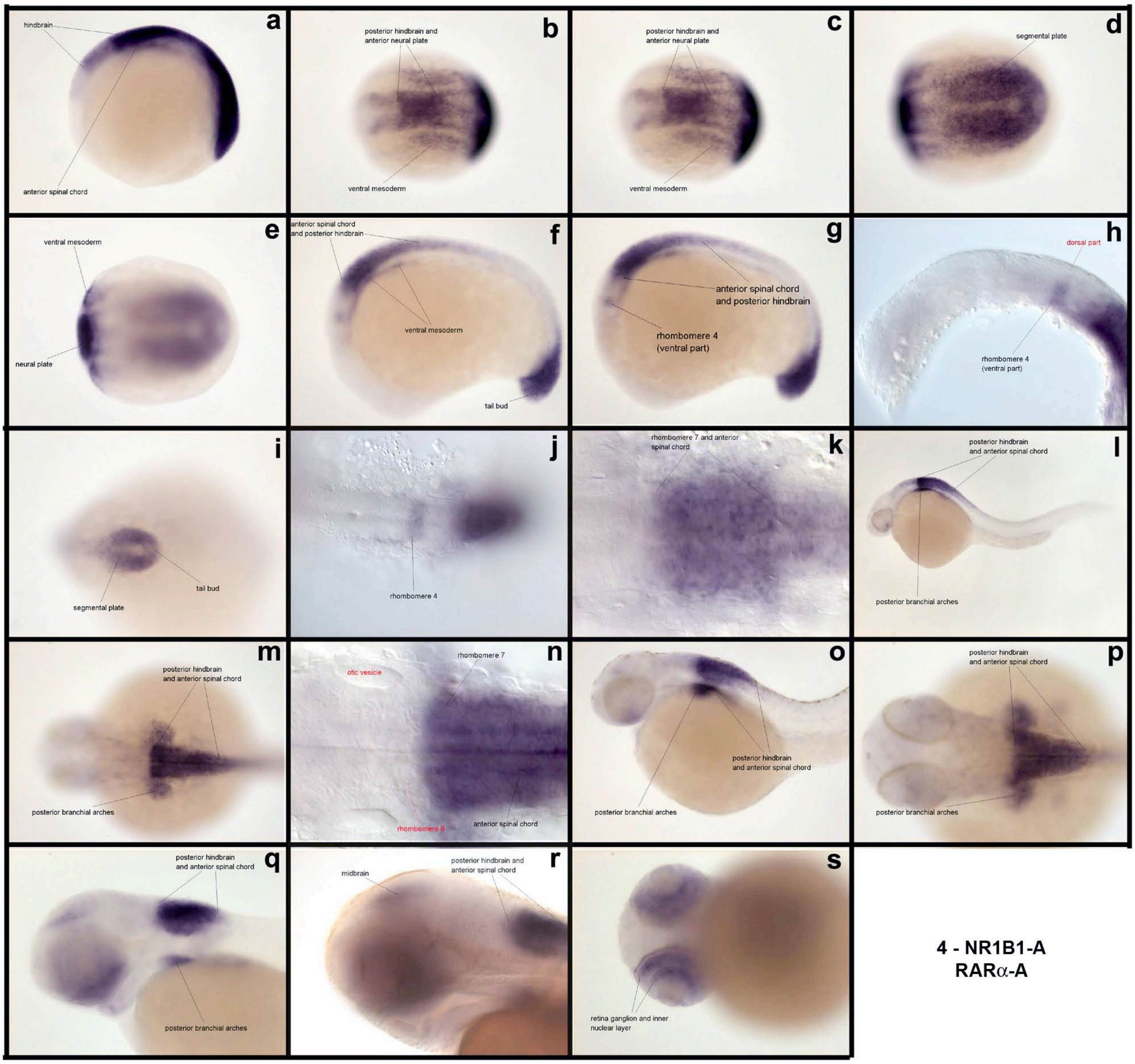

4 - NR1B1-A
RARα-A

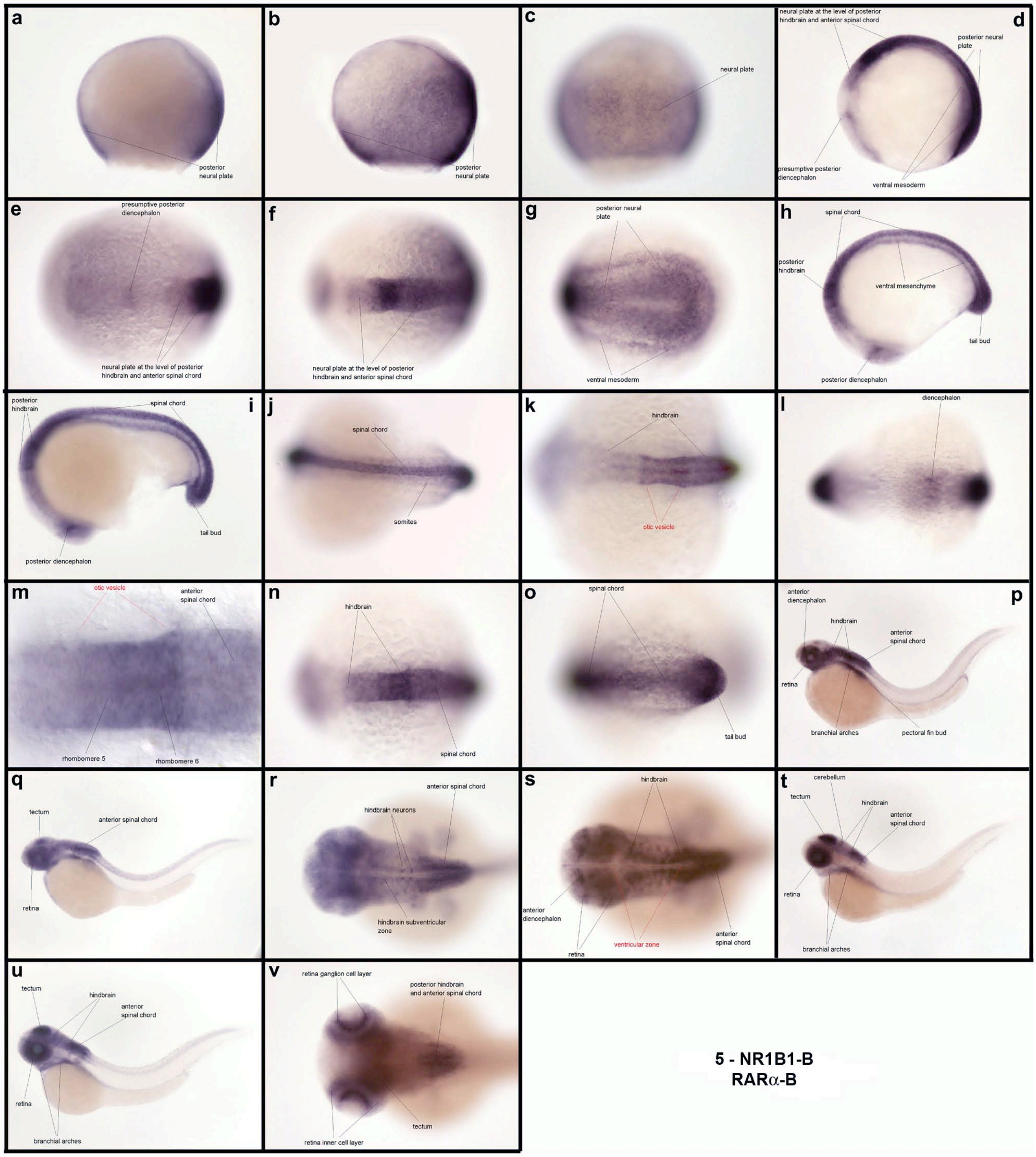

5 - NR1B1-B
RARα-B

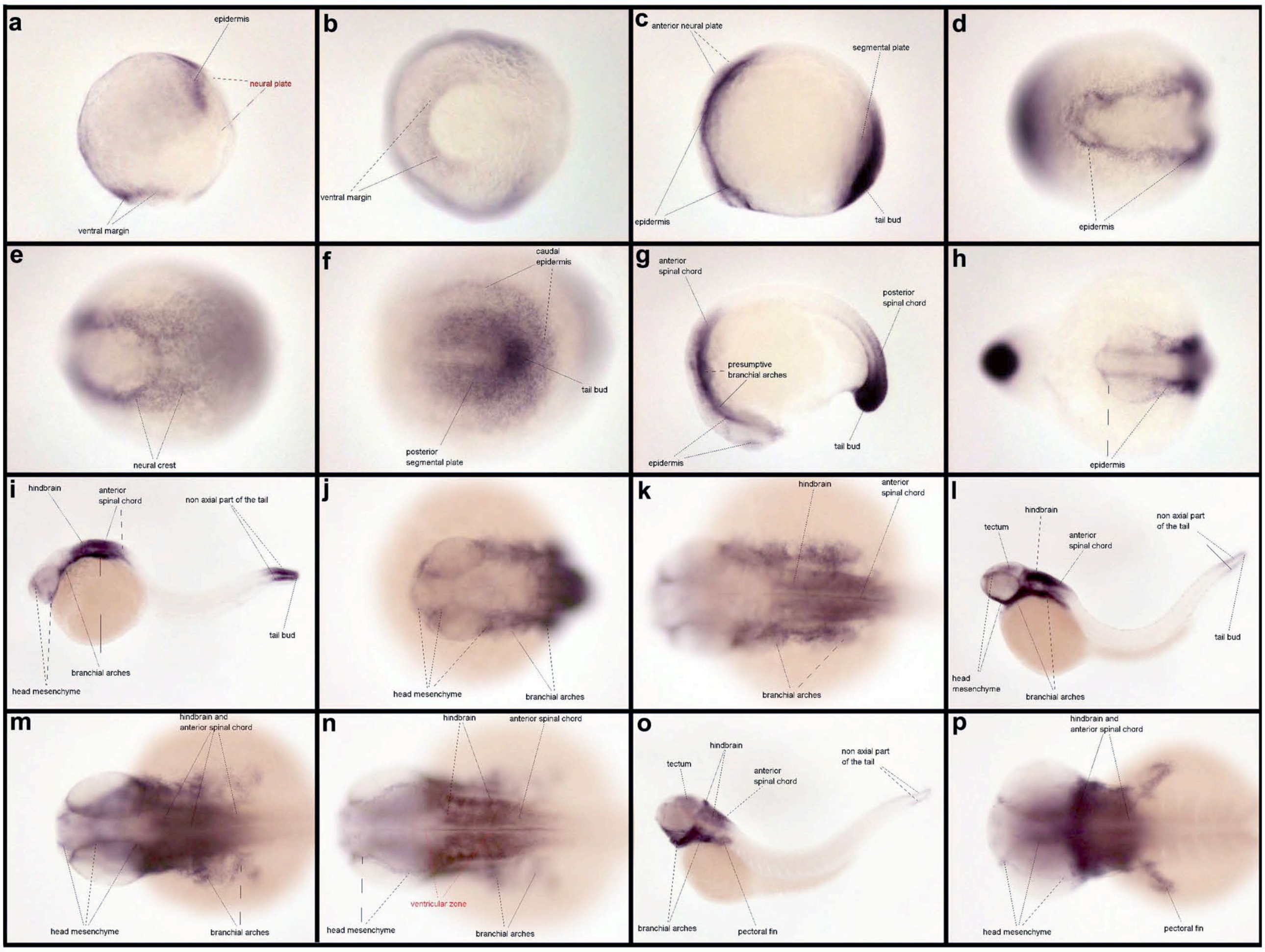

6 - NR1B3-A
RARγ-A

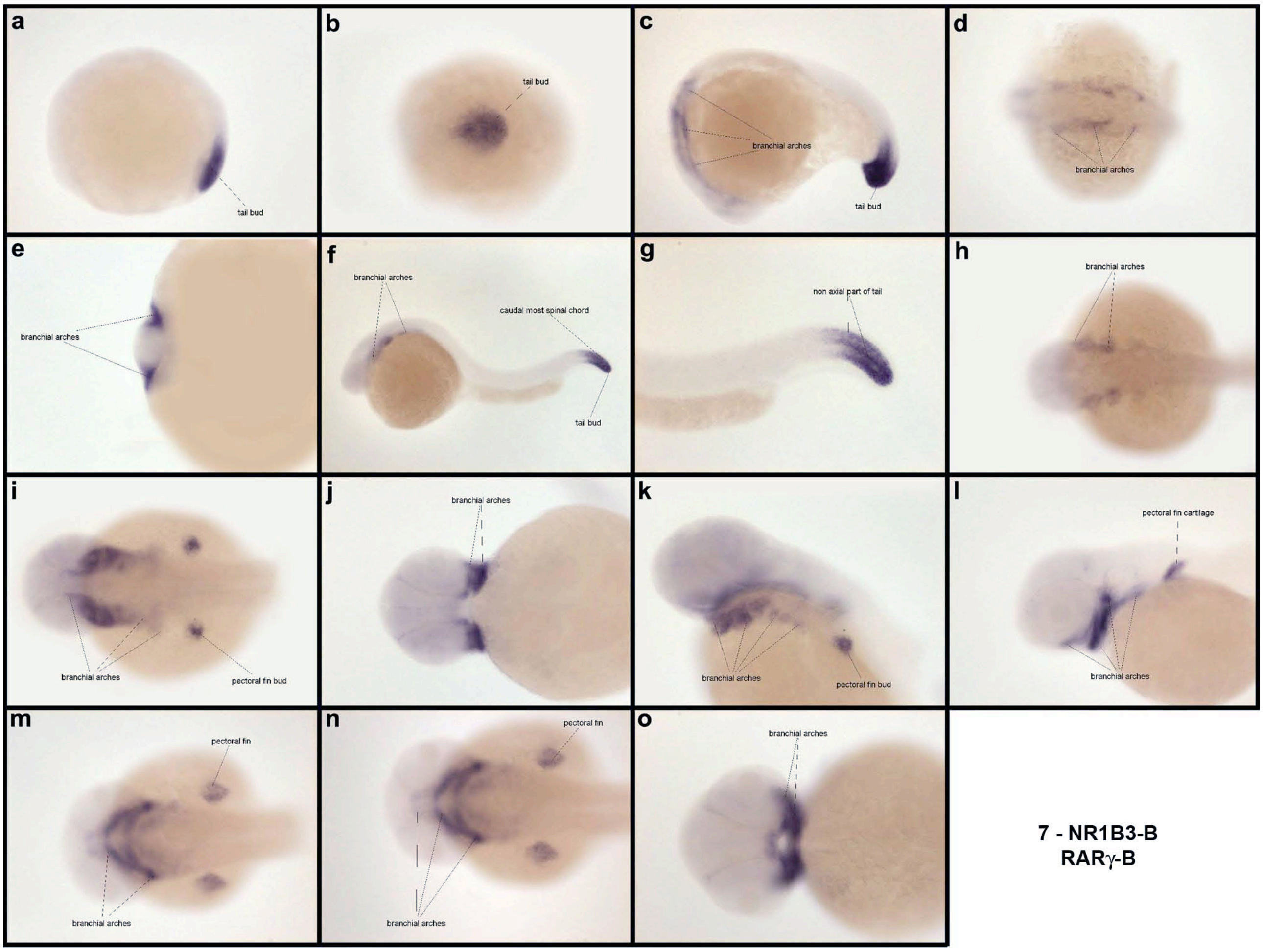

7 - NR1B3-B
RARγ-B

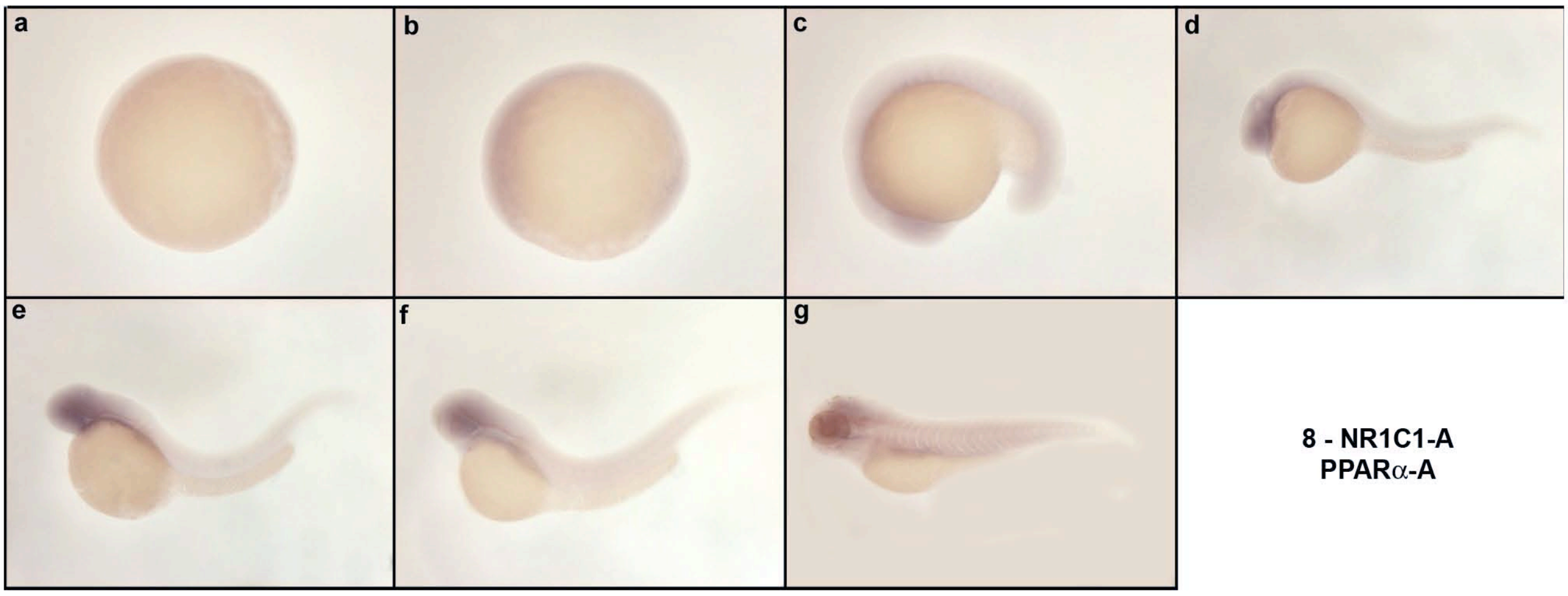

8 - NR1C1-A
PPARα-A

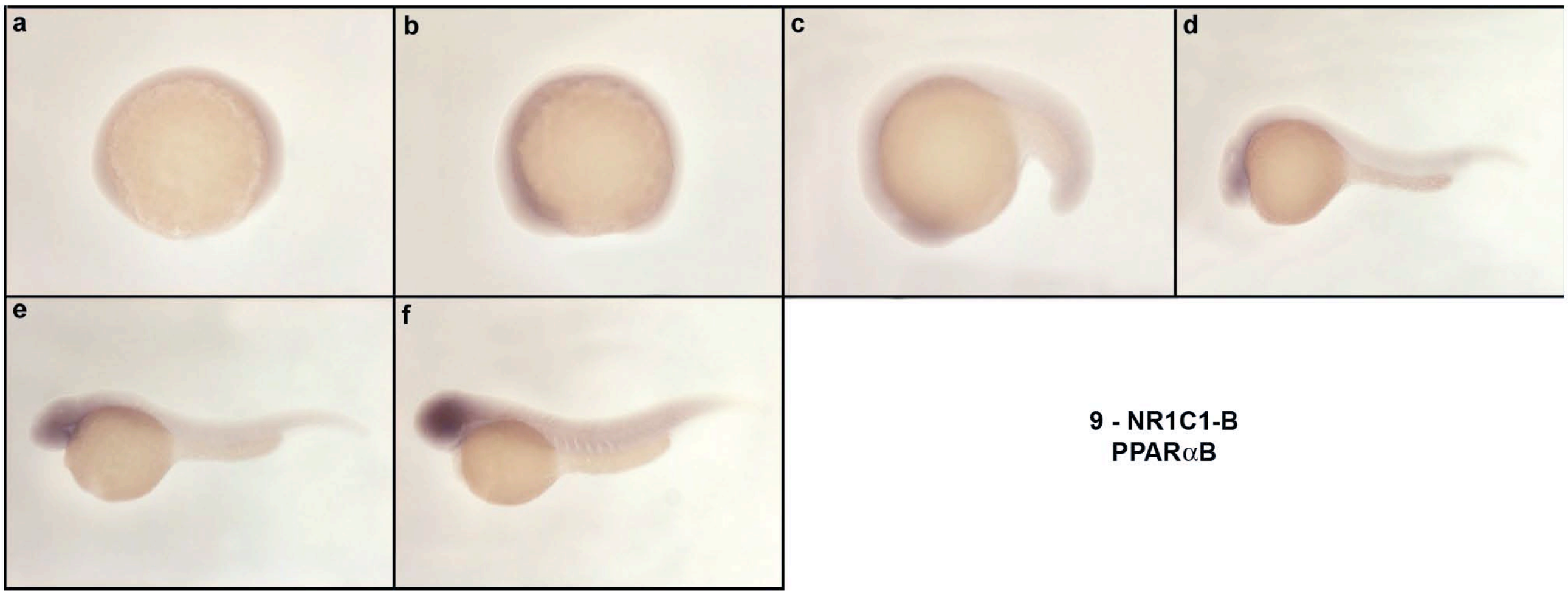

9 - NR1C1-B
PPARαB

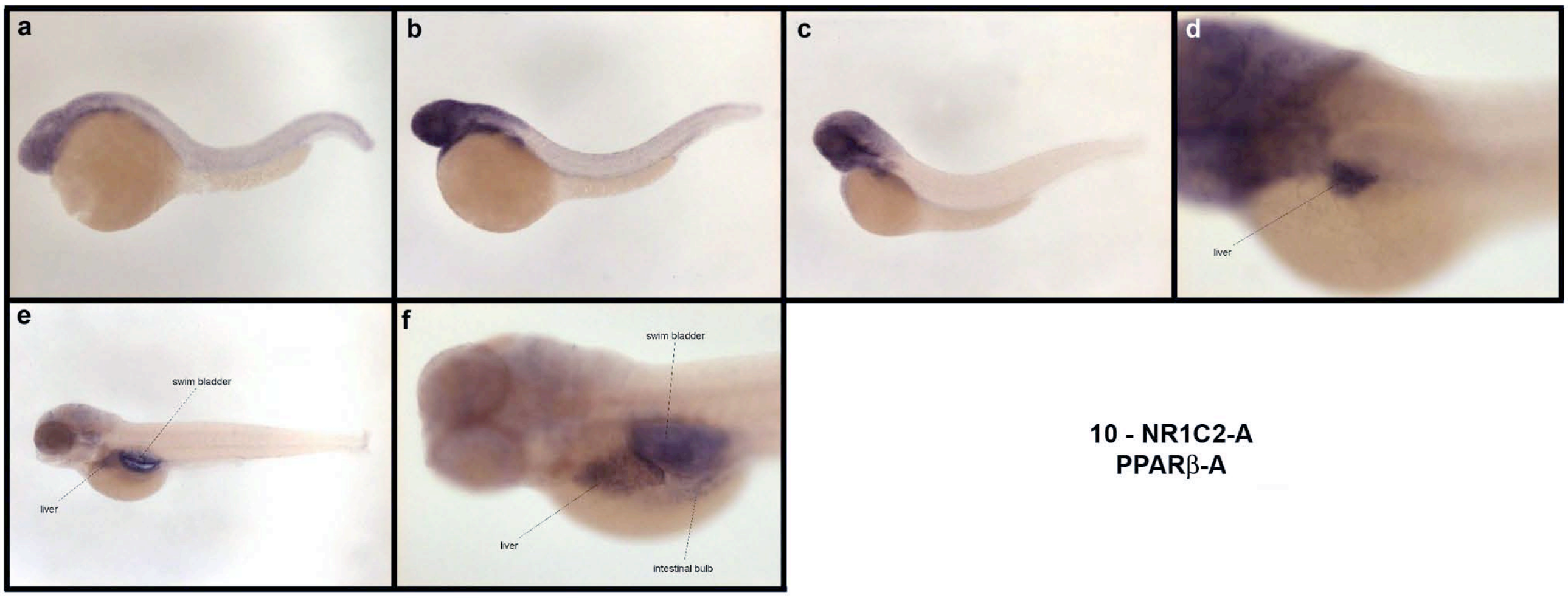

10 - NR1C2-A
PPARβ-A

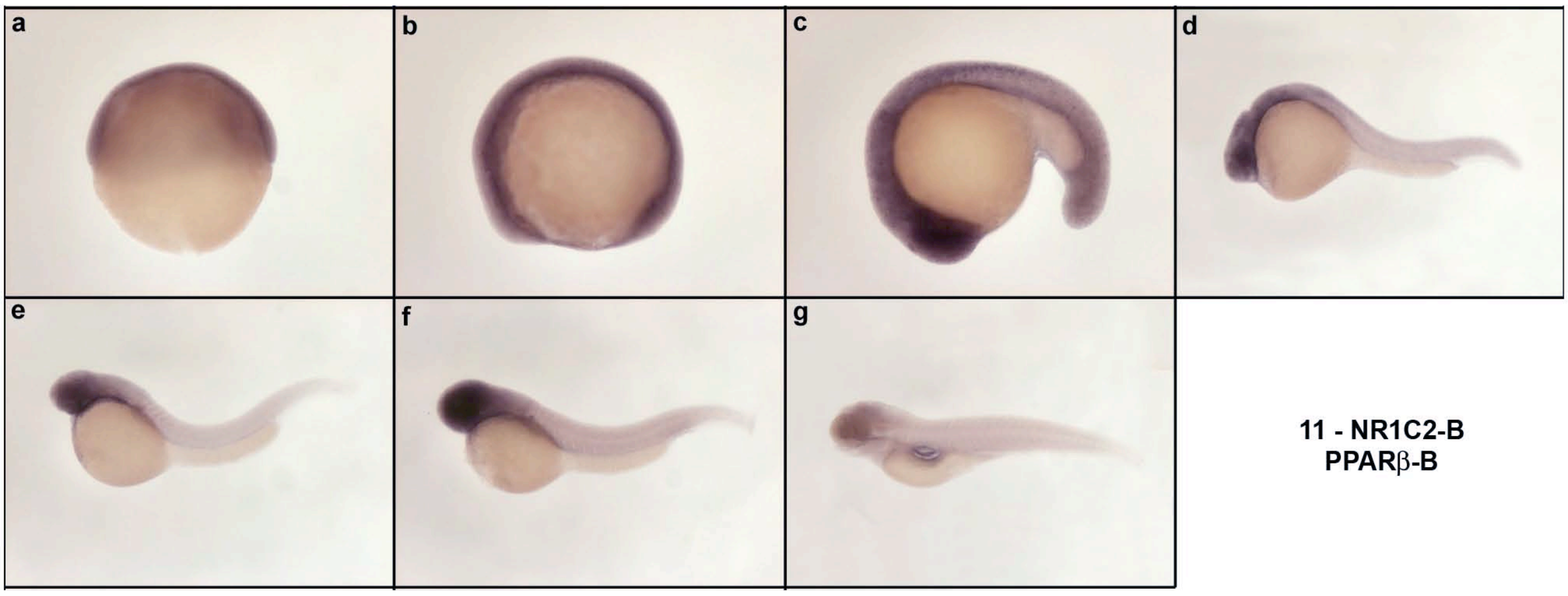

11 - NR1C2-B
PPARβ-B

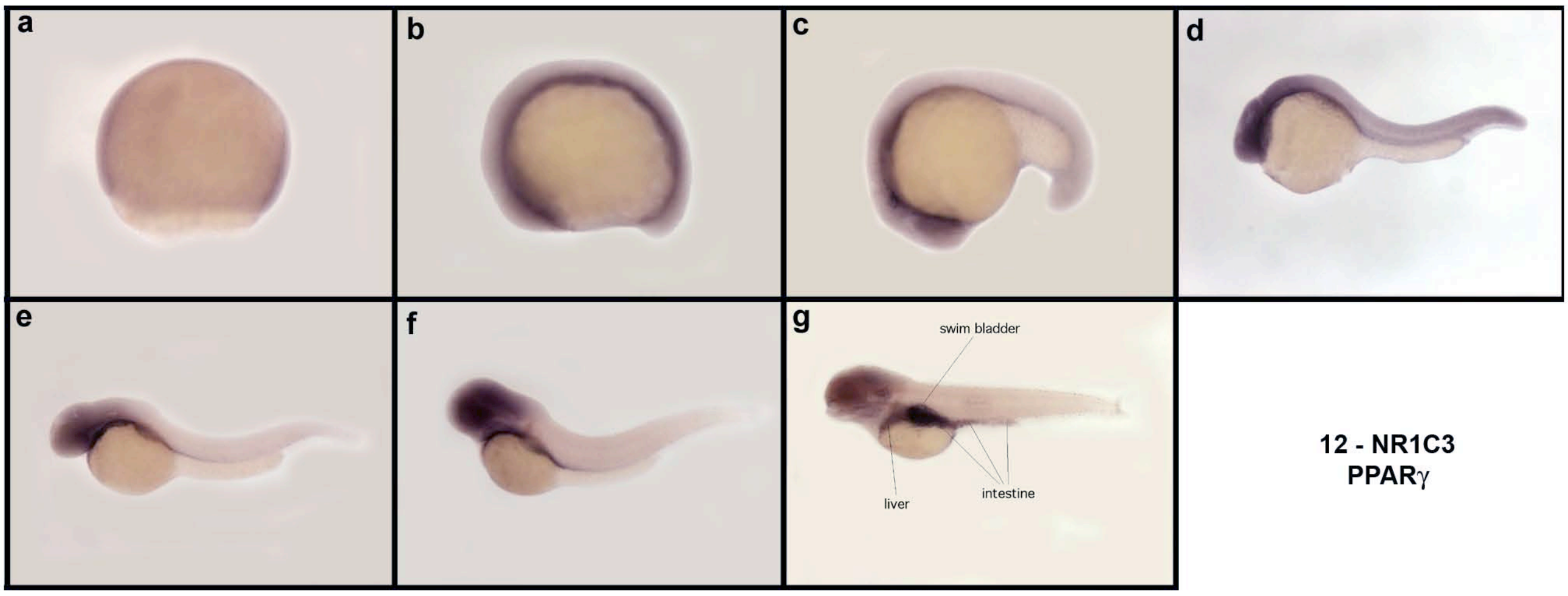

12 - NR1C3
PPARγ

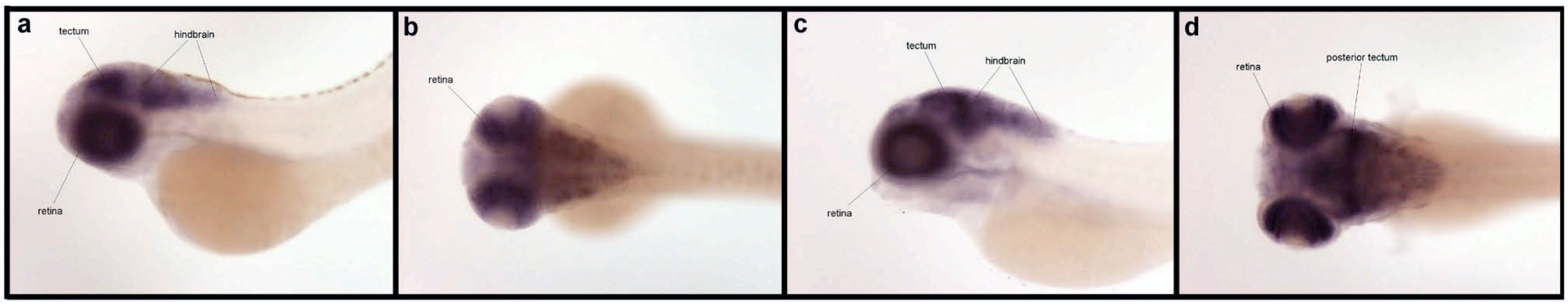

13 - NR1D1
Reverbα

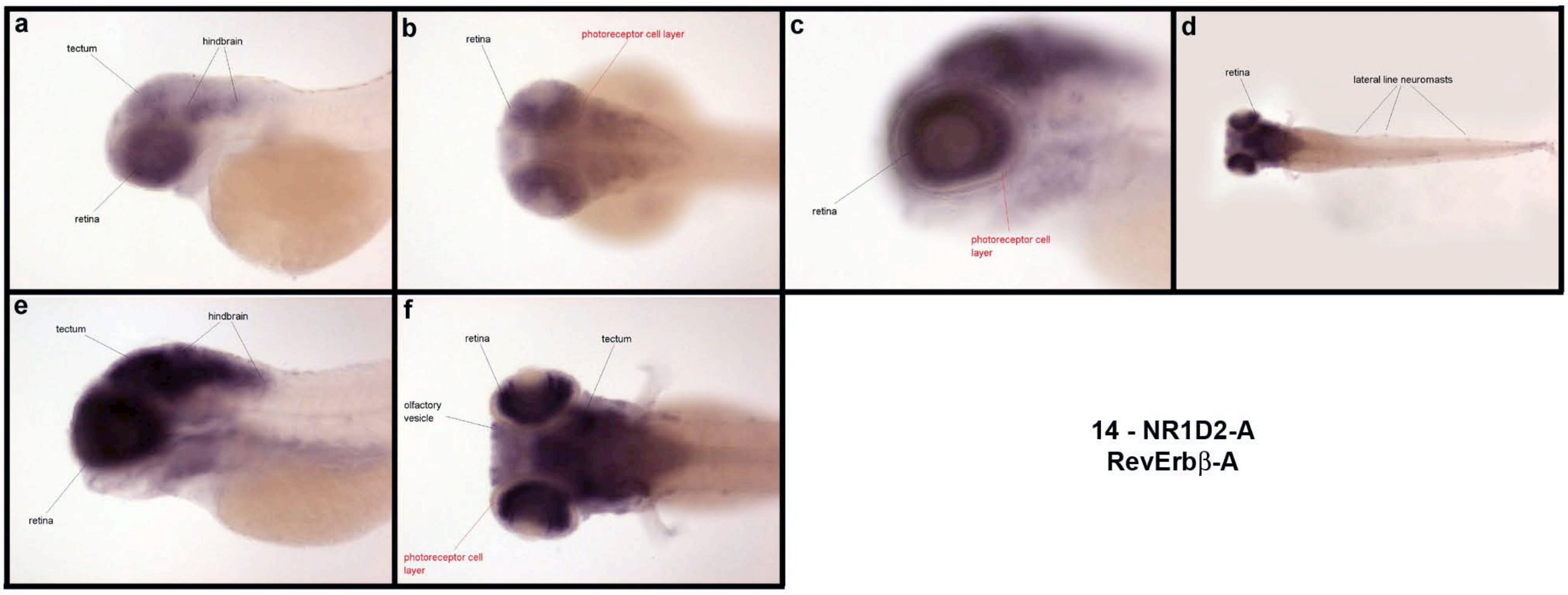

14 - NR1D2-A
RevErbβ-A

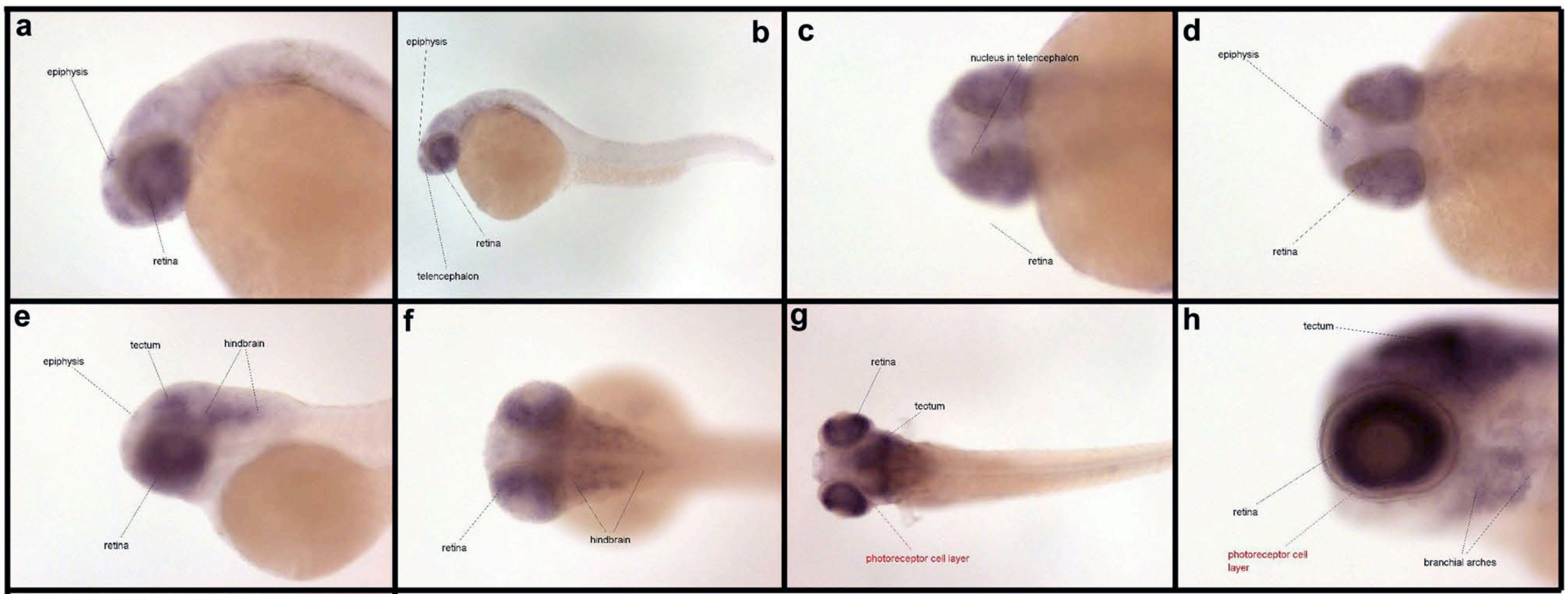

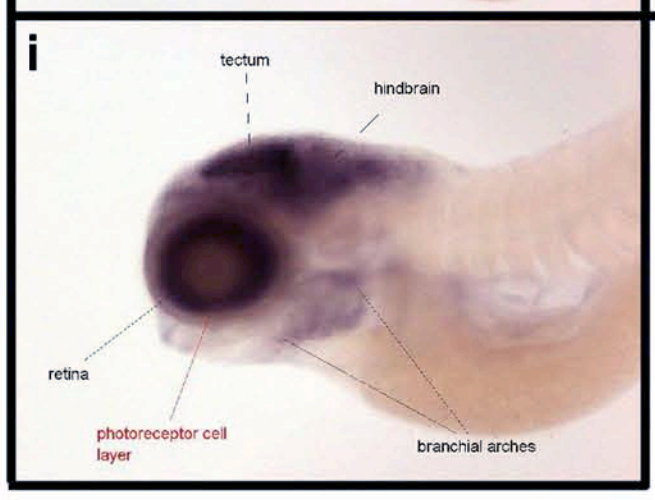

15 - NR1D2-B
Reverbβ-B

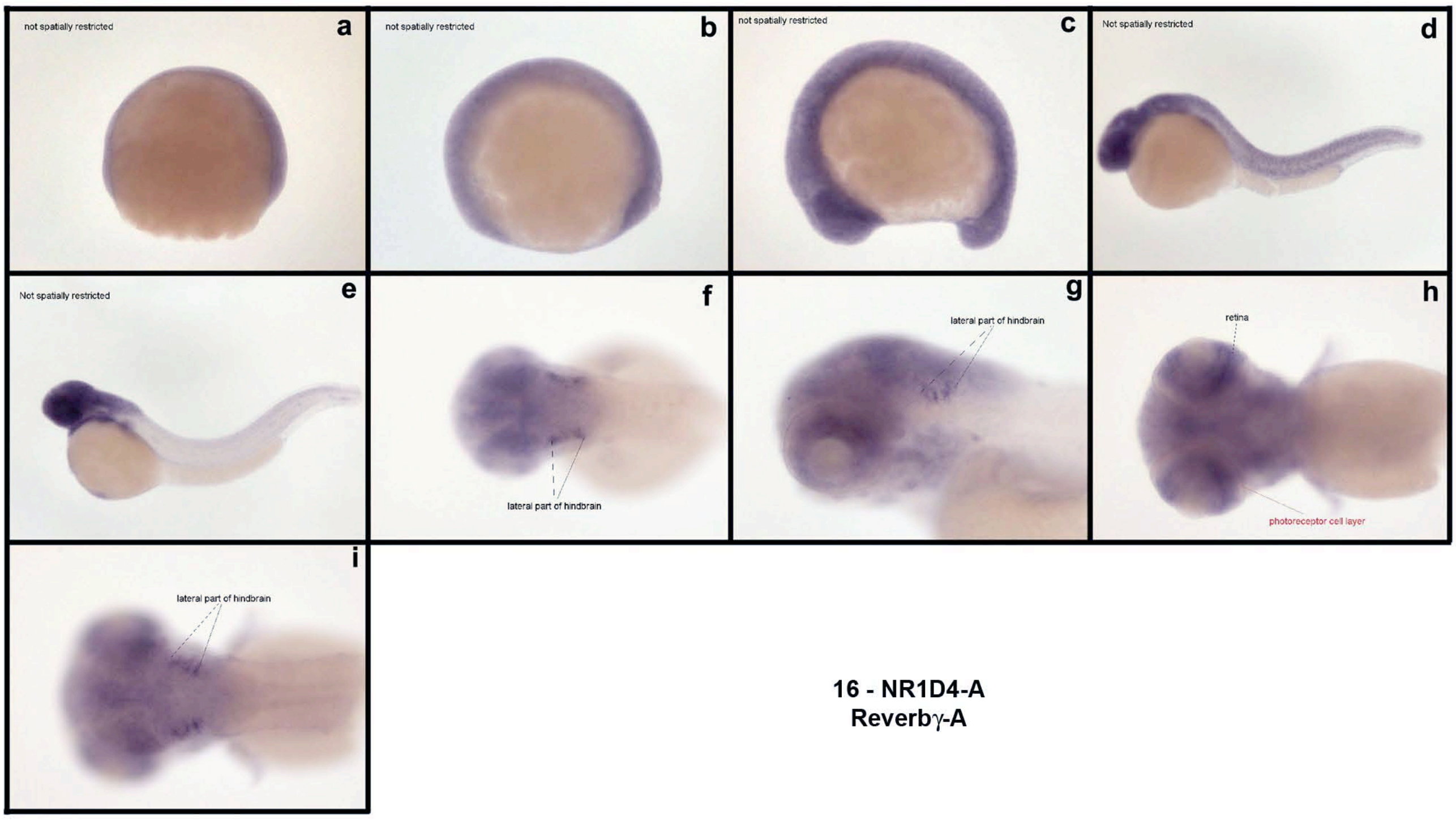

16 - NR1D4-A
Reverbγ-A

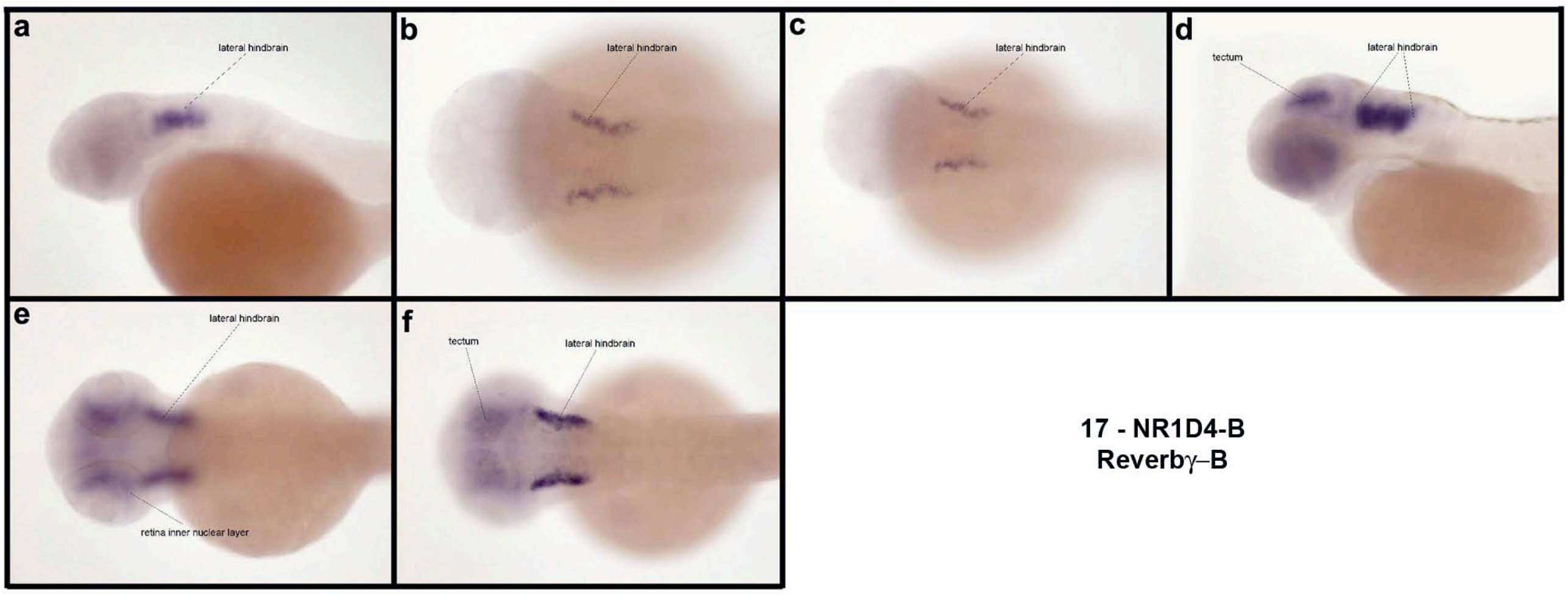

17 - NR1D4-B
Reverbγ–B

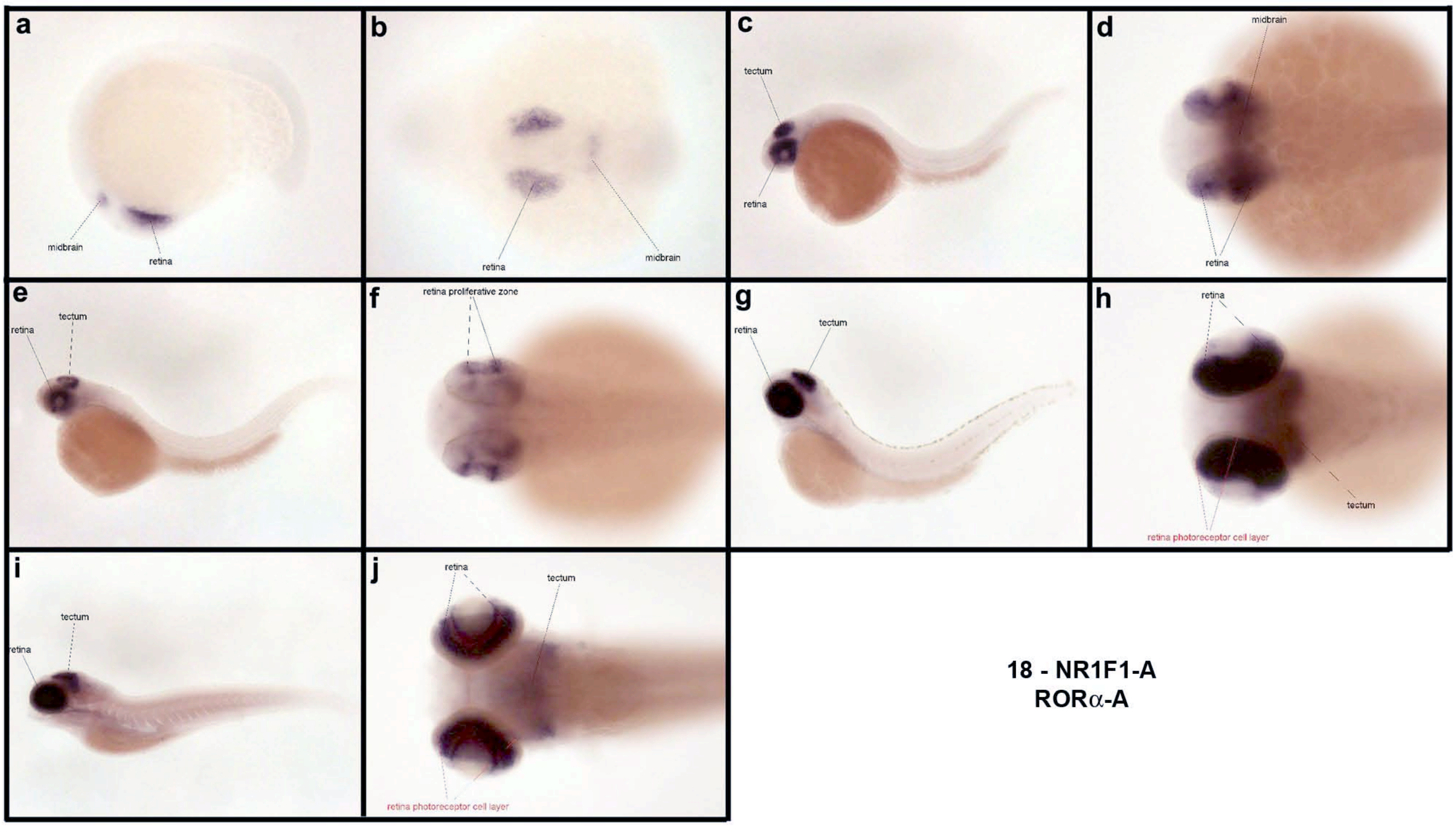

18 - NR1F1-A
RORα-A

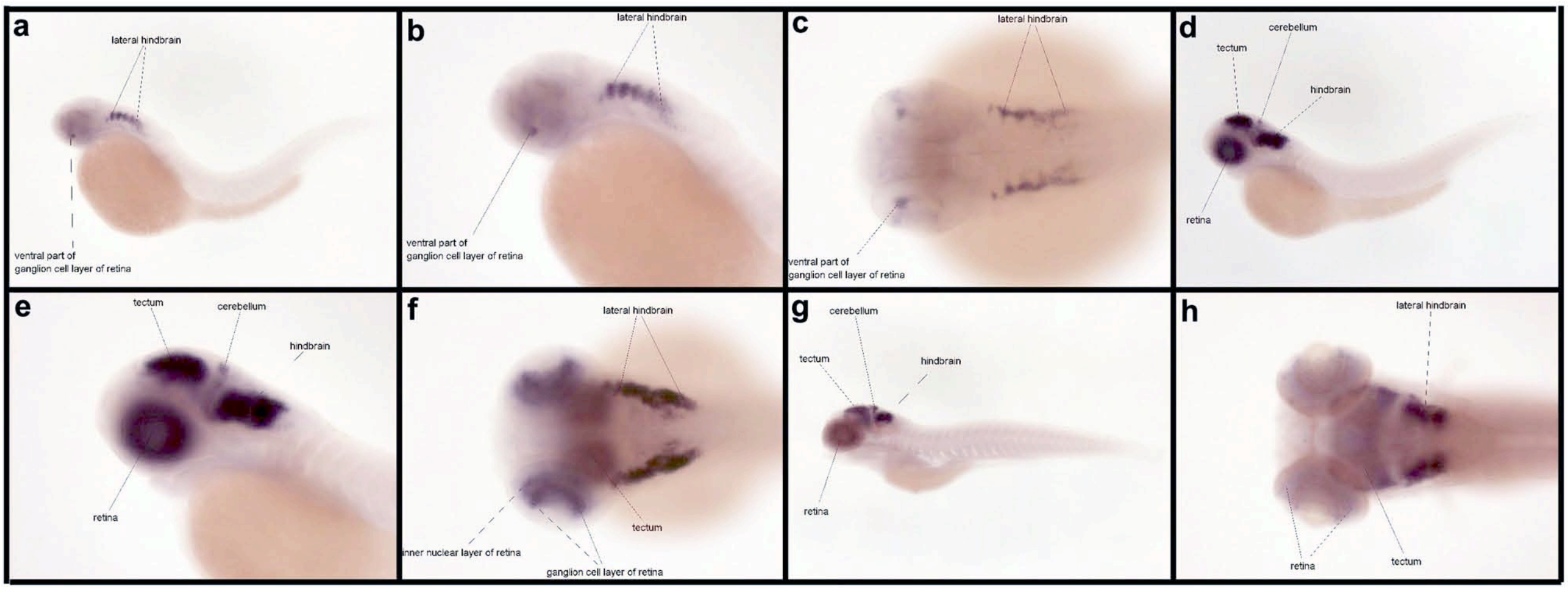

19 - NR1F1-B
RORα-B

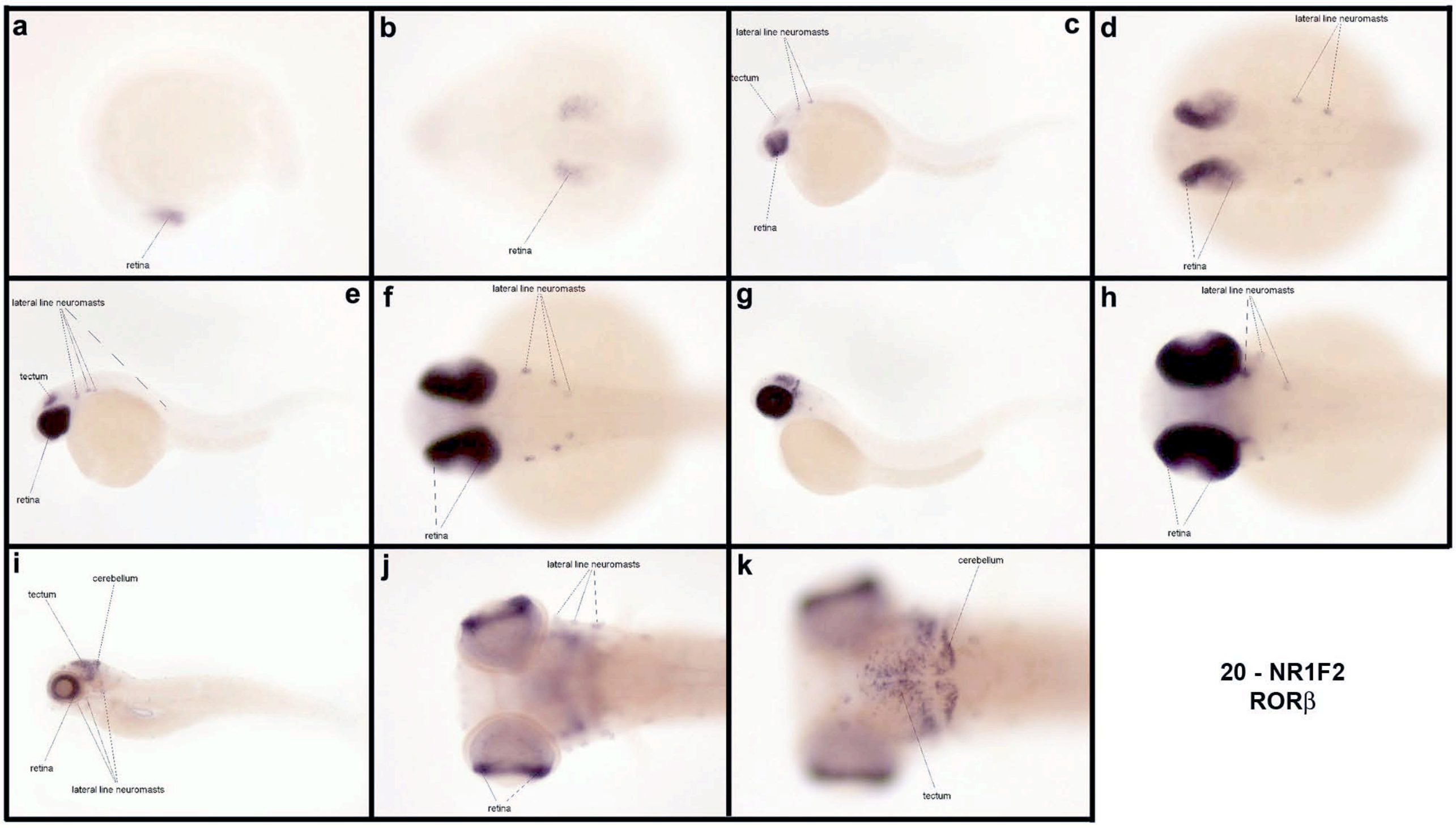

20 - NR1F2
RORβ

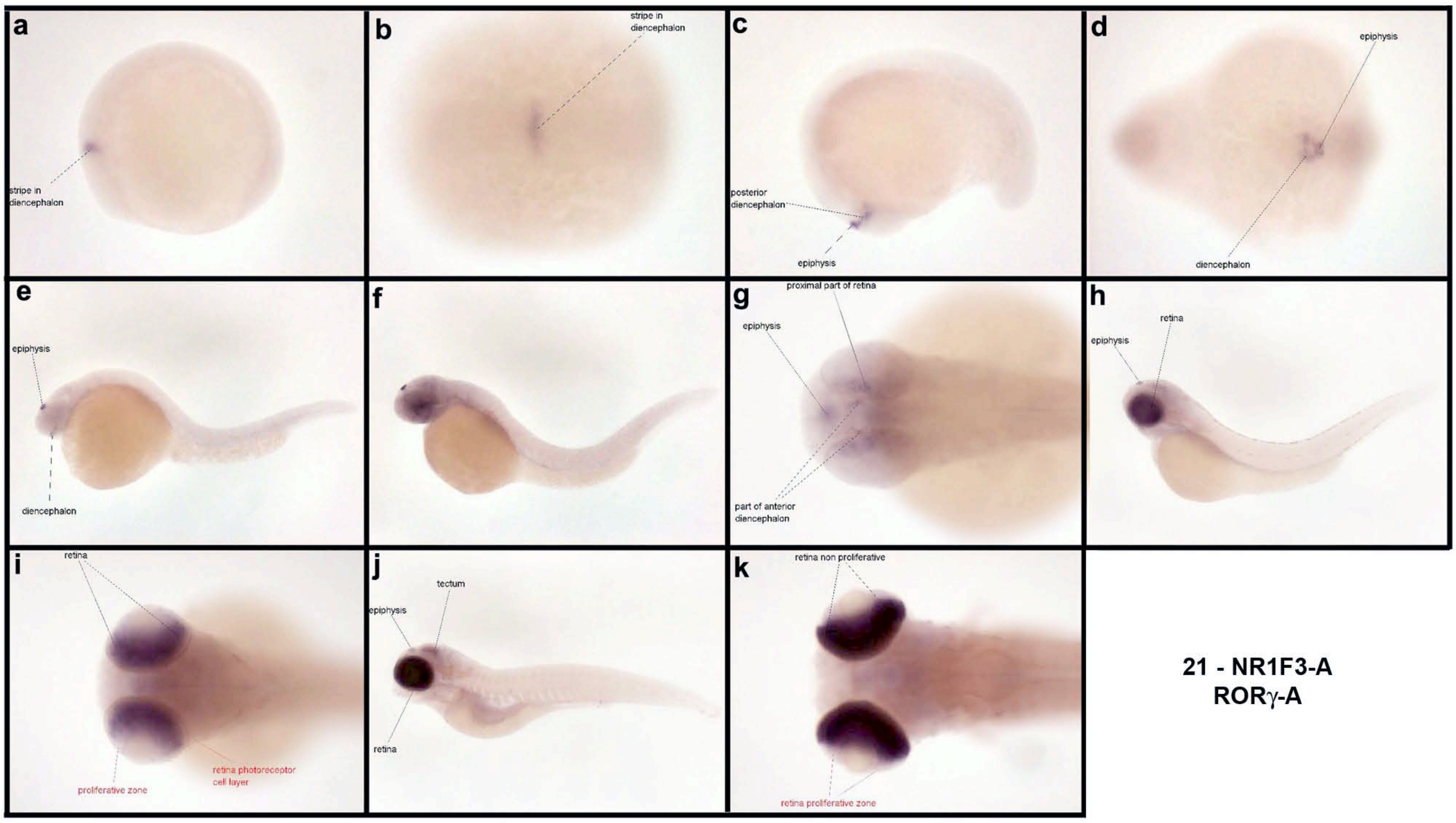

21 - NR1F3-A
RORγ-A

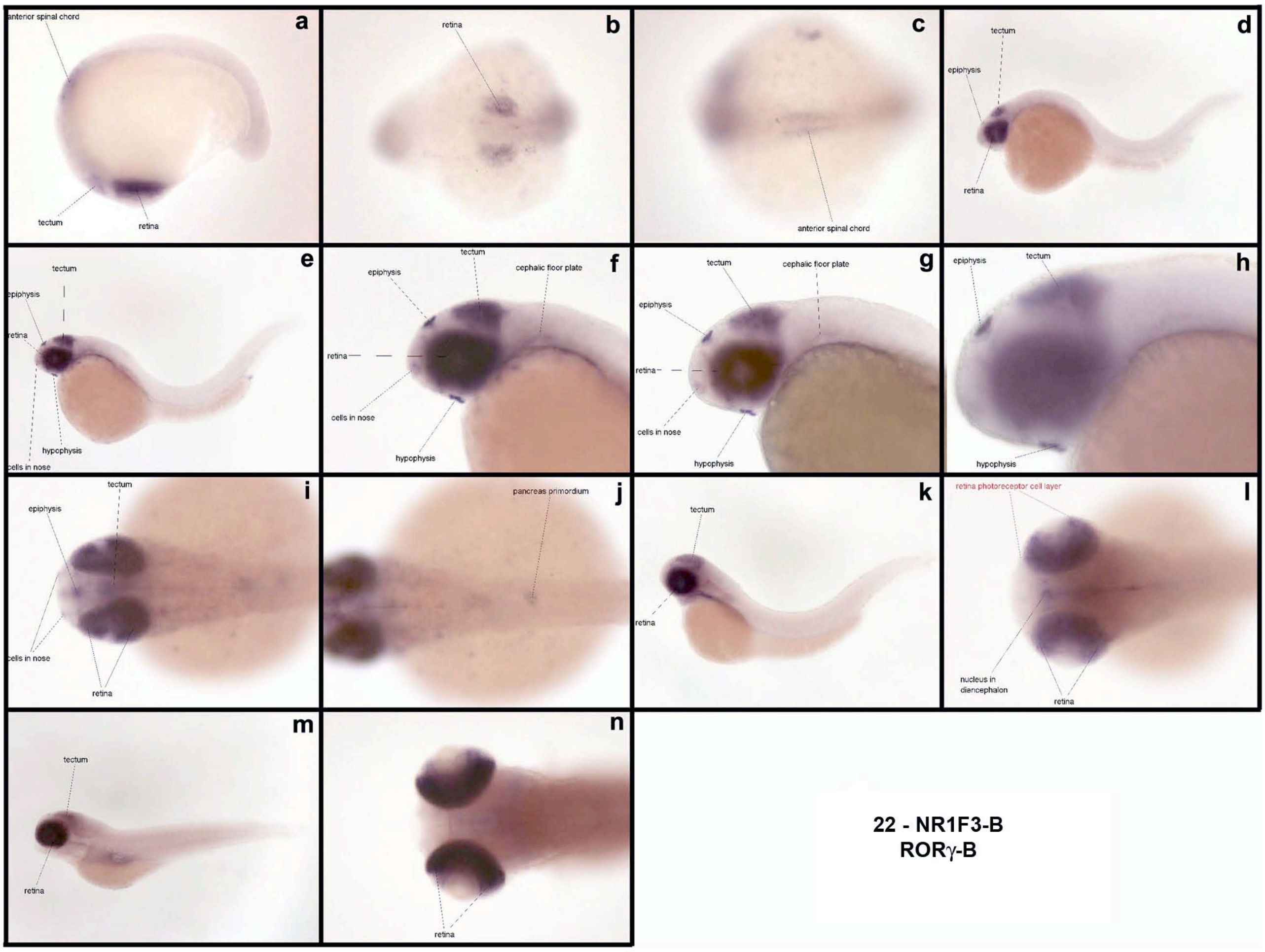

22 - NR1F3-B
RORγ-B

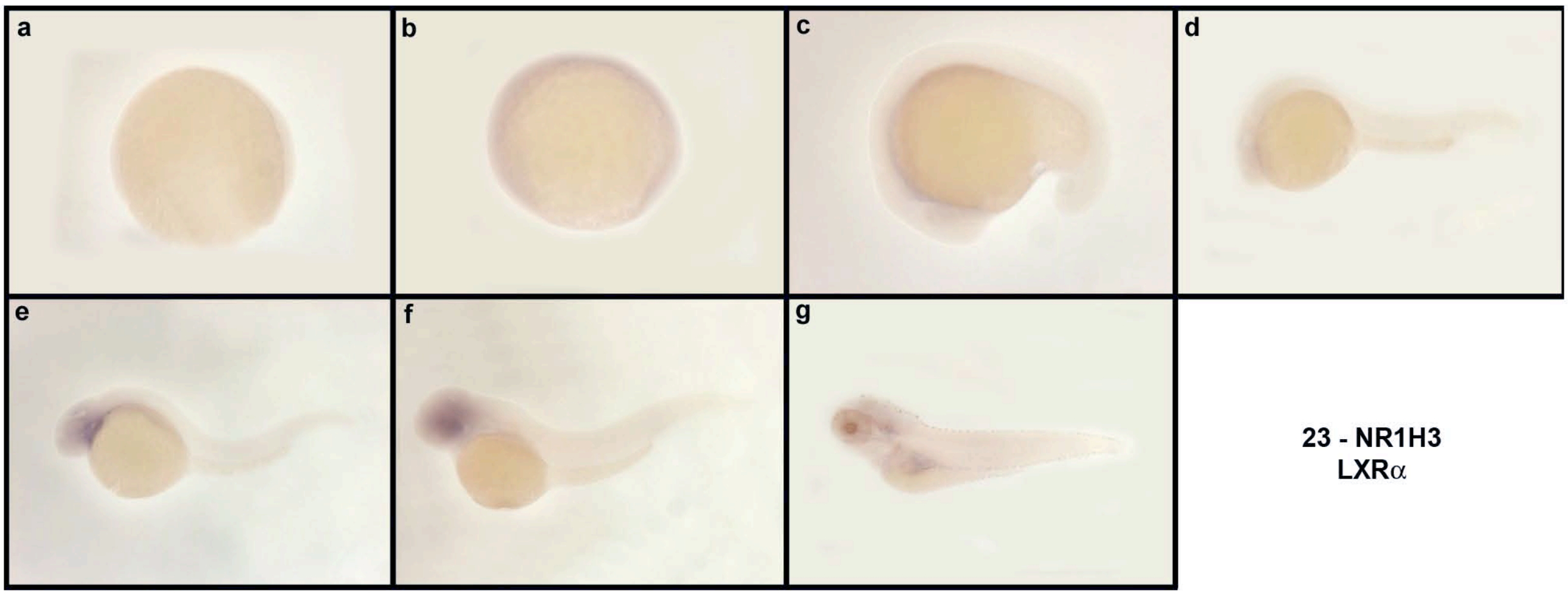

23 - NR1H3
LXRα

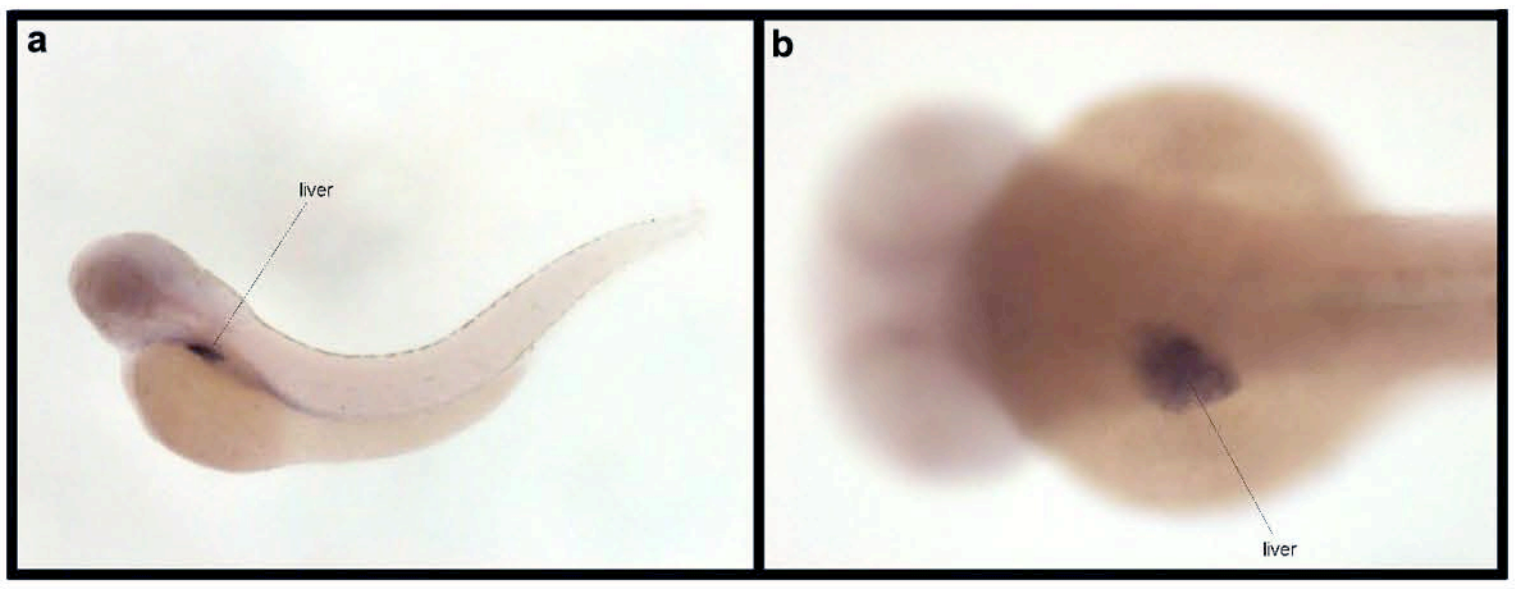

24 - NR1H4
FXRα

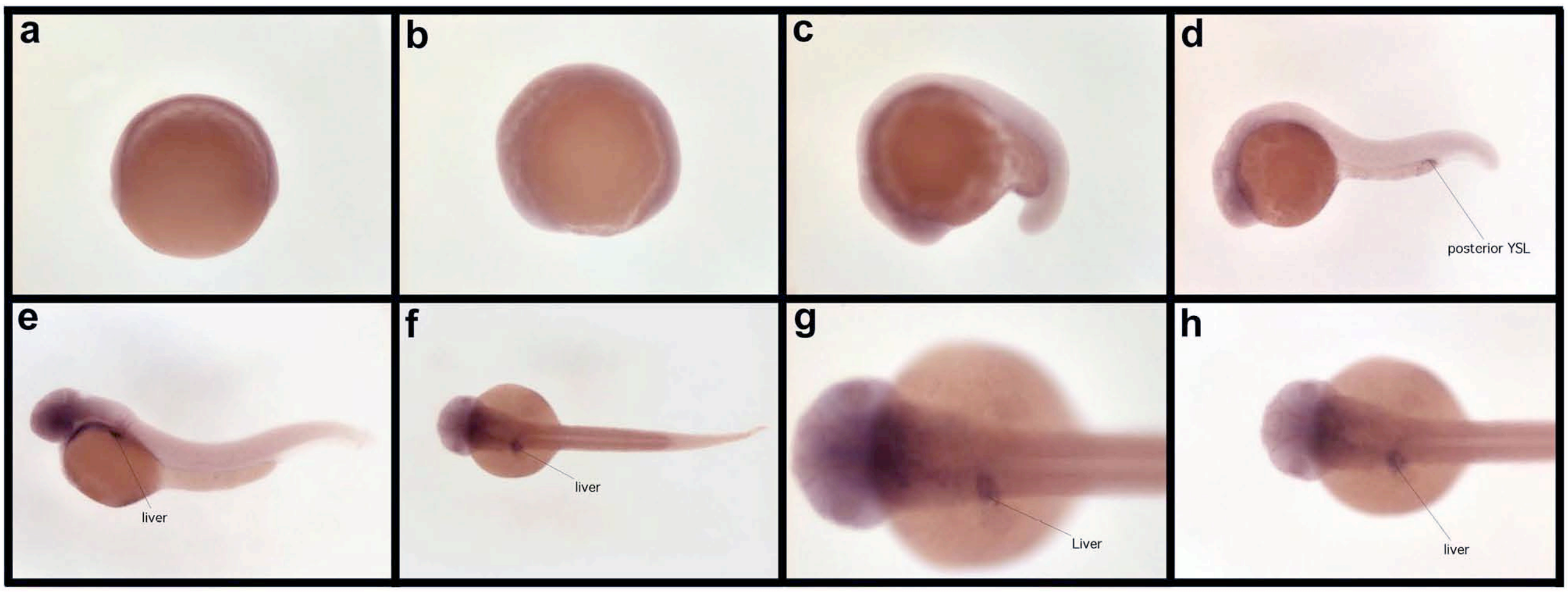

25 - NR1H5
FXRβ

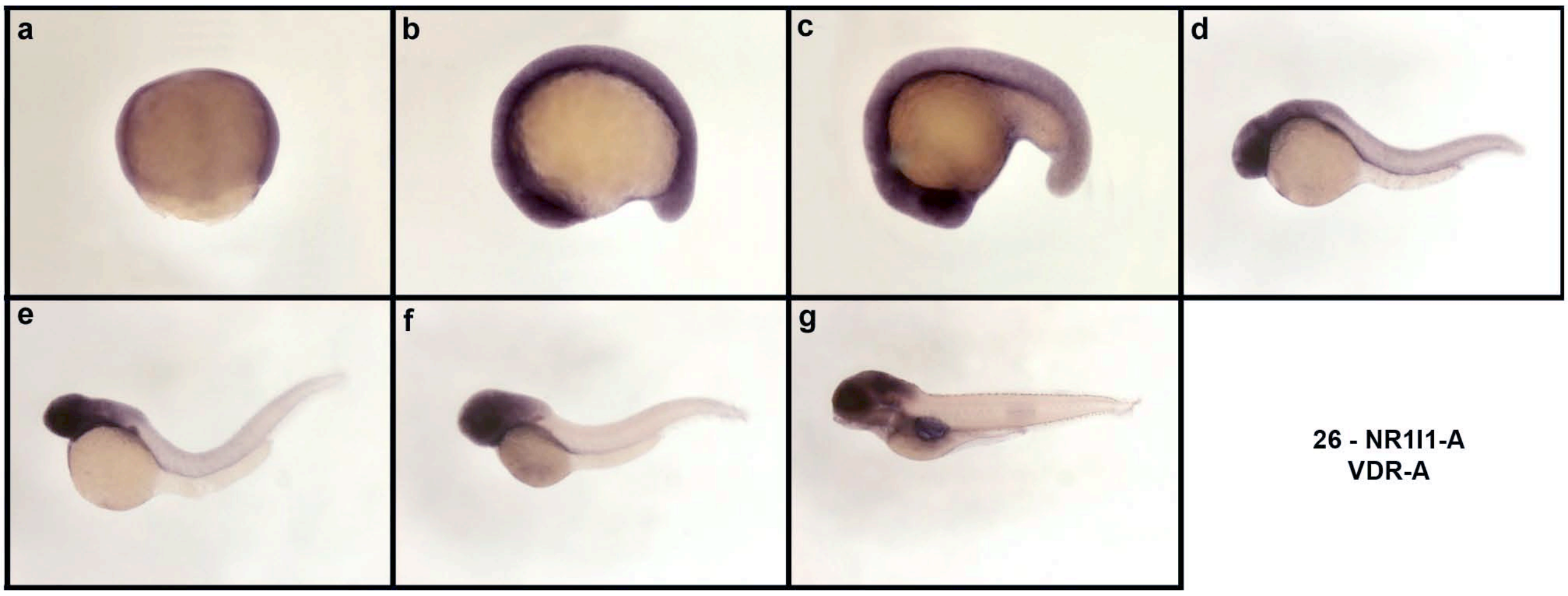

26 - NR1I1-A
VDR-A

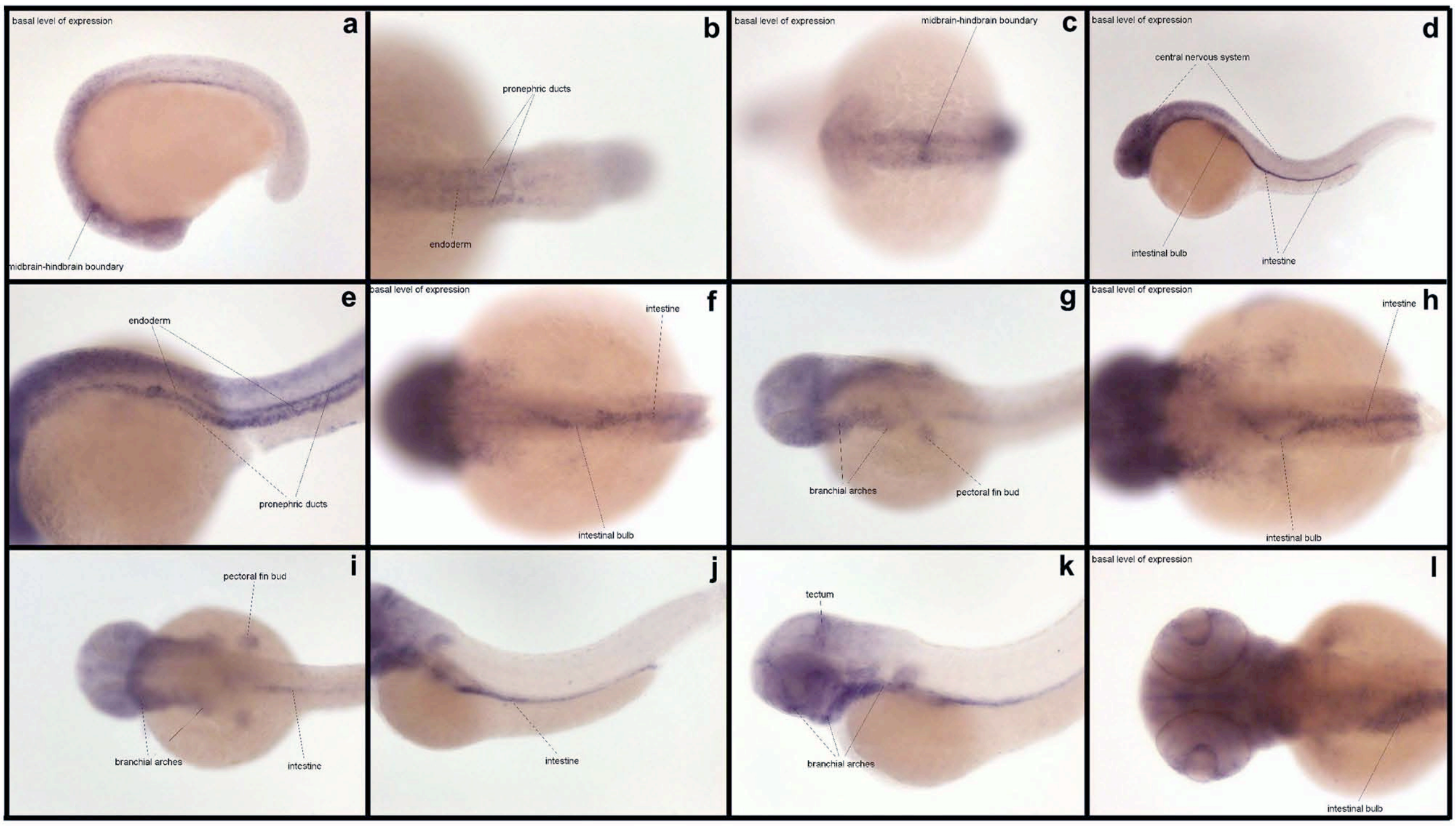

27 - NR1I1-B
VDR-B

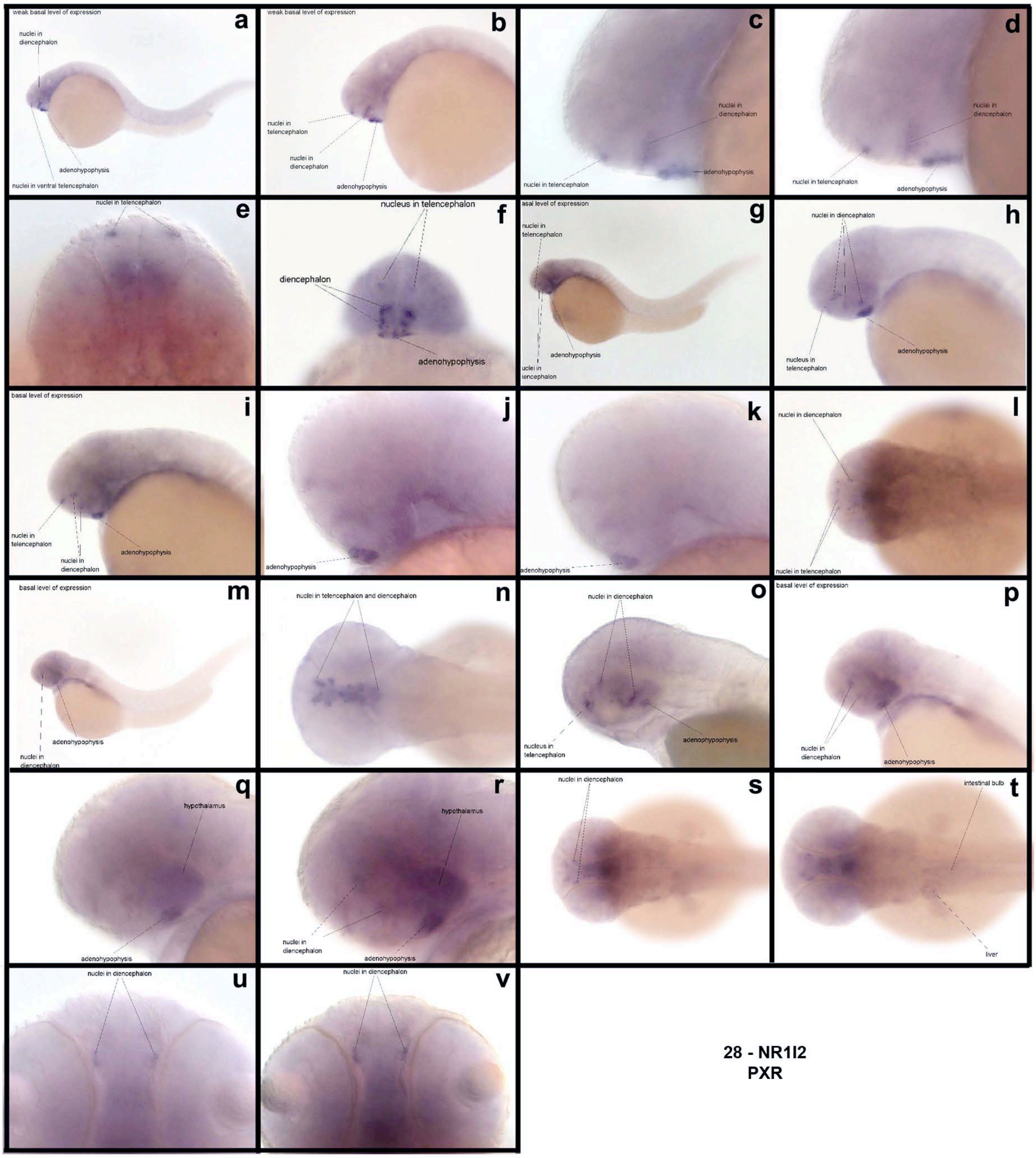
a
weak basal level of expression
nuclei in diencephalon
adenohypophysis
nuclei in ventral telencephalon
b
weak basal level of expression
nuclei in telencephalon
nuclei in diencephalon
adenohypophysis
c
nuclei in diencephalon
nuclei in telencephalon
adenohypophysis
d
nuclei in diencephalon
nuclei in telencephalon
adenohypophysis
e
nuclei in telencephalon
f
nucleus in telencephalon
diencephalon
adenohypophysis
g
asal level of expression
nuclei in telencephalon
adenohypophysis
uclei in iencephalon
h
nuclei in diencephalon
nucleus in telencephalon
adenohypophysis
i
basal level of expression
nuclei in telencephalon
nuclei in diencephalon
adenohypophysis
j
adenohypophysis
k
adenohypophysis
l
nuclei in diencephalon
nuclei in telencephalon
m
basal level of expression
adenohypophysis
nuclei in diencephalon
n
nuclei in telencephalon and diencephalon
o
nuclei in diencephalon
nucleus in telencephalon
adenohypophysis
p
basal level of expression
nuclei in diencephalon
adenohypophysis
q
hypothalamus
adenohypophysis
r
hypothalamus
nuclei in diencephalon
adenohypophysis
s
nuclei in diencephalon
t
intestinal bulb
liver
u
nuclei in diencephalon
v
nuclei in diencephalon

28 - NR1I2
PXR

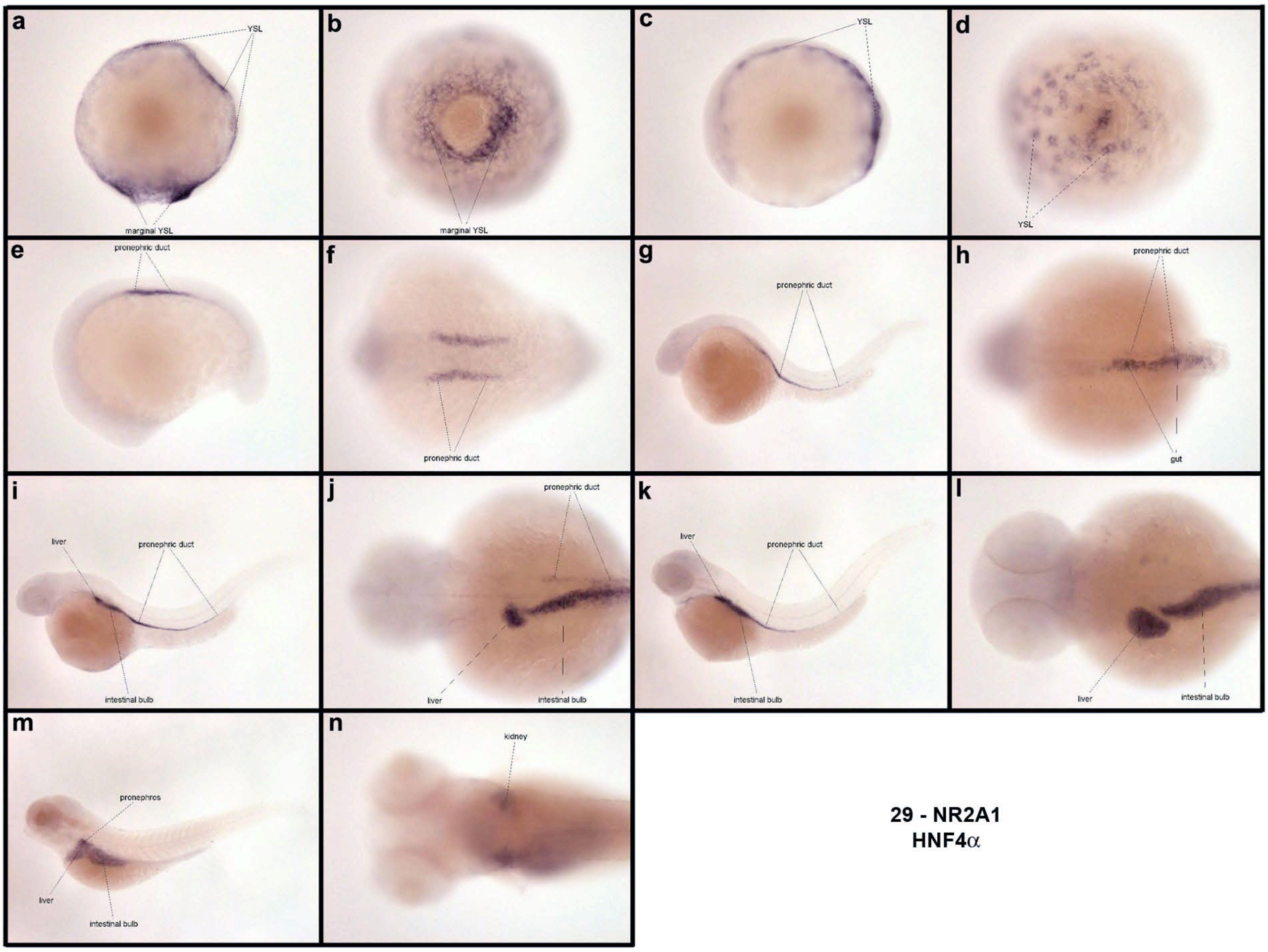

29 - NR2A1
HNF4α

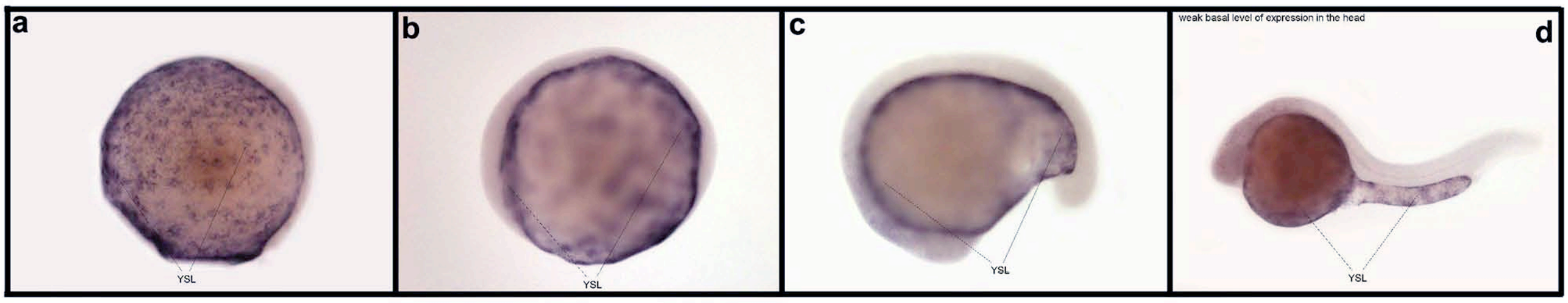

30 - NR2A3
HNF4β

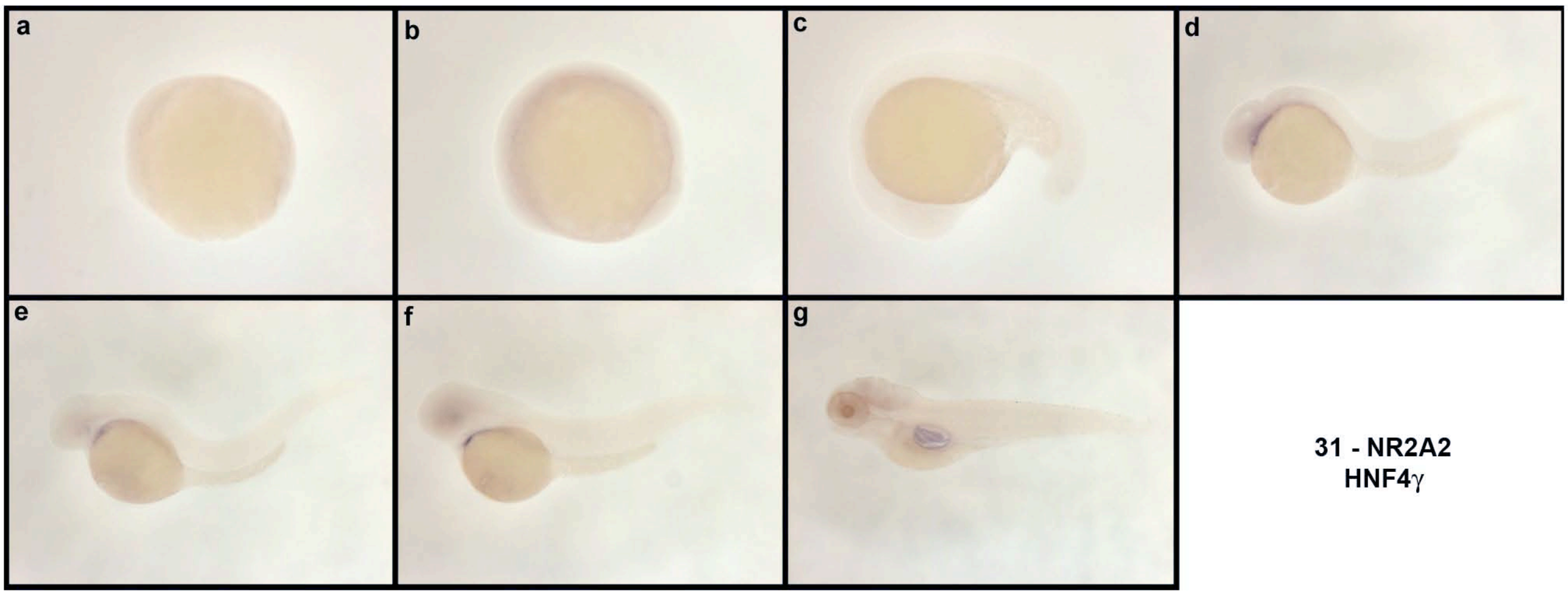

31 - NR2A2
HNF4γ

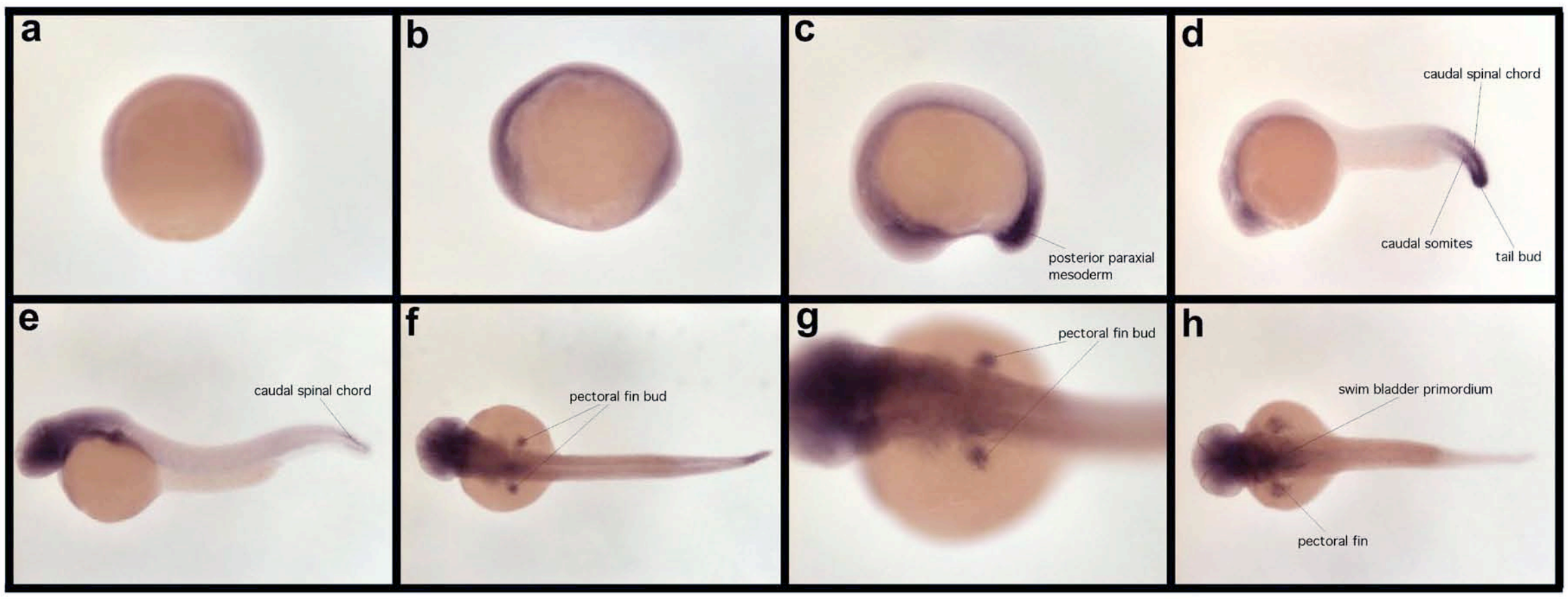

32 - NR2B1-A
RXRα-A

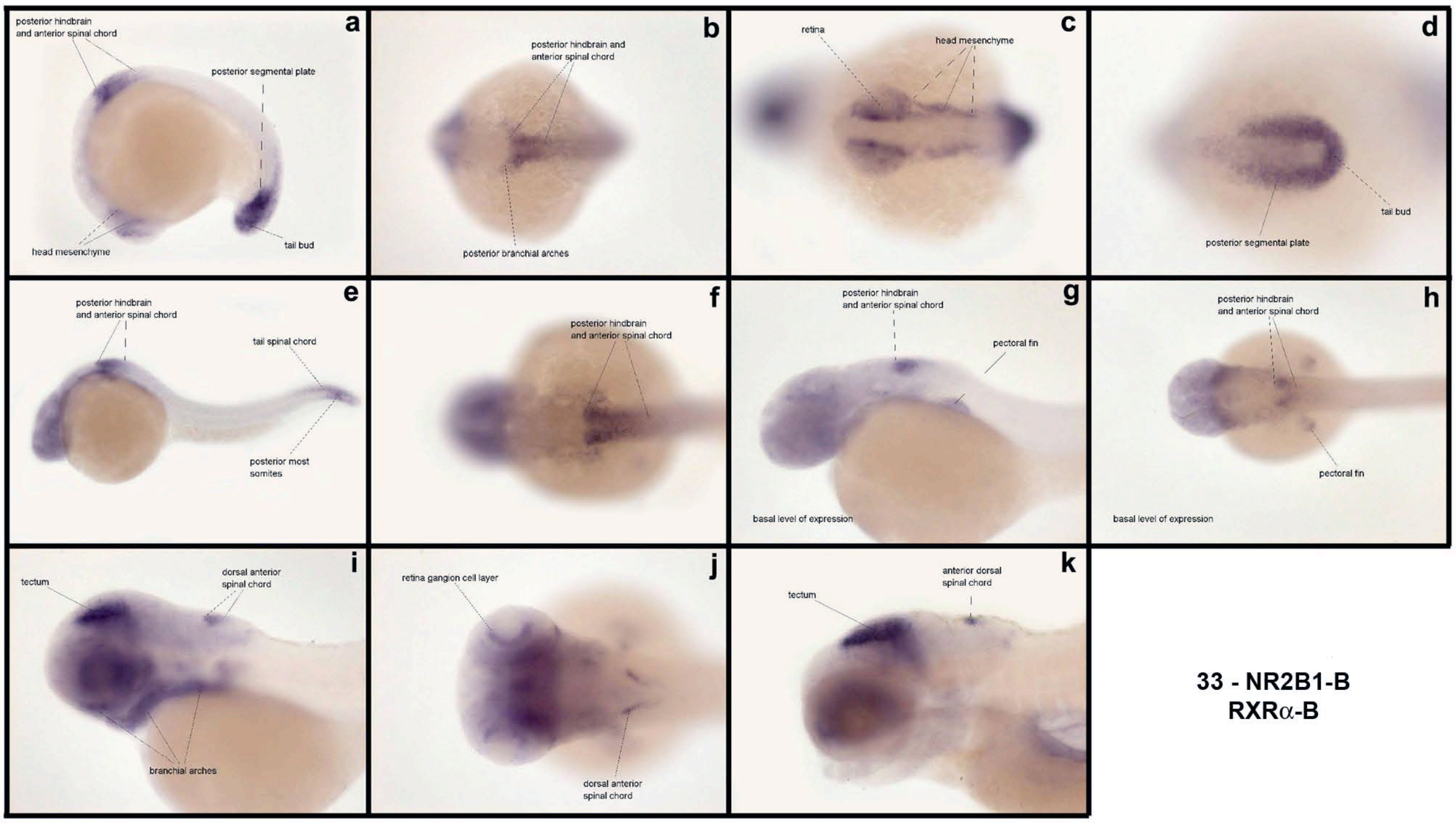

33 - NR2B1-B
RXRα-B

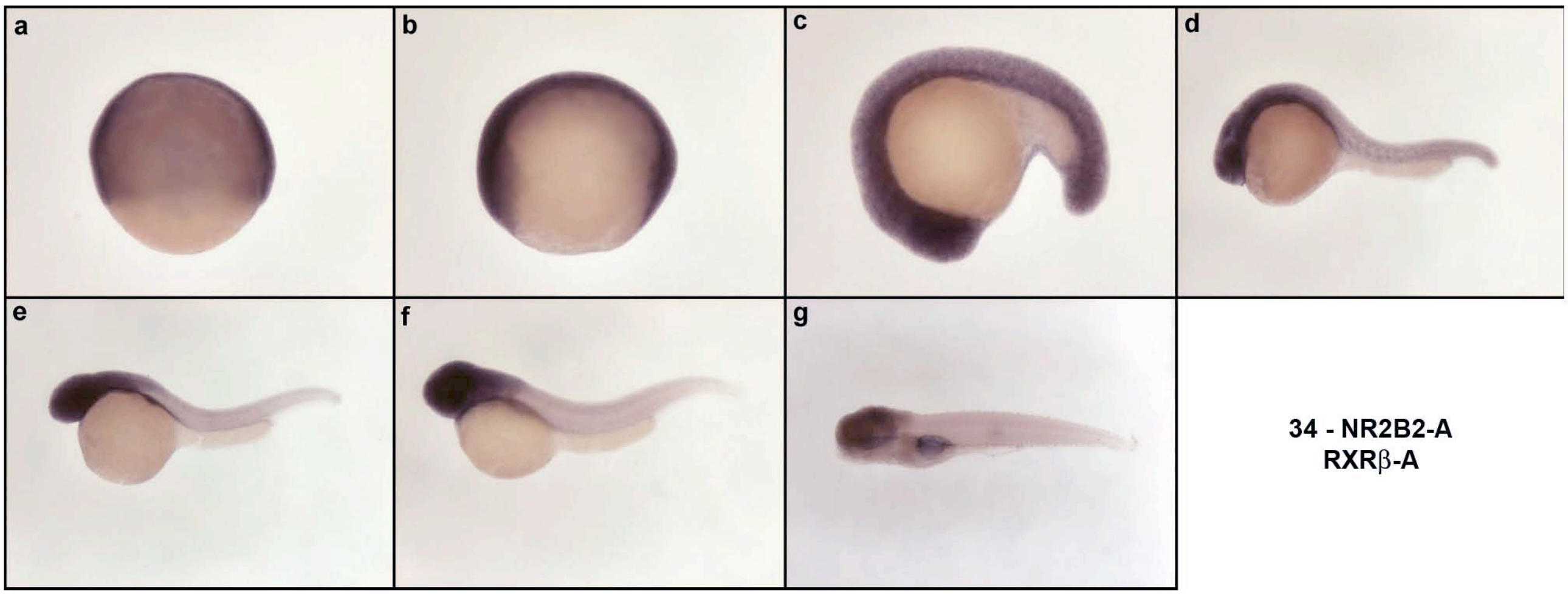

34 - NR2B2-A
RXRβ-A

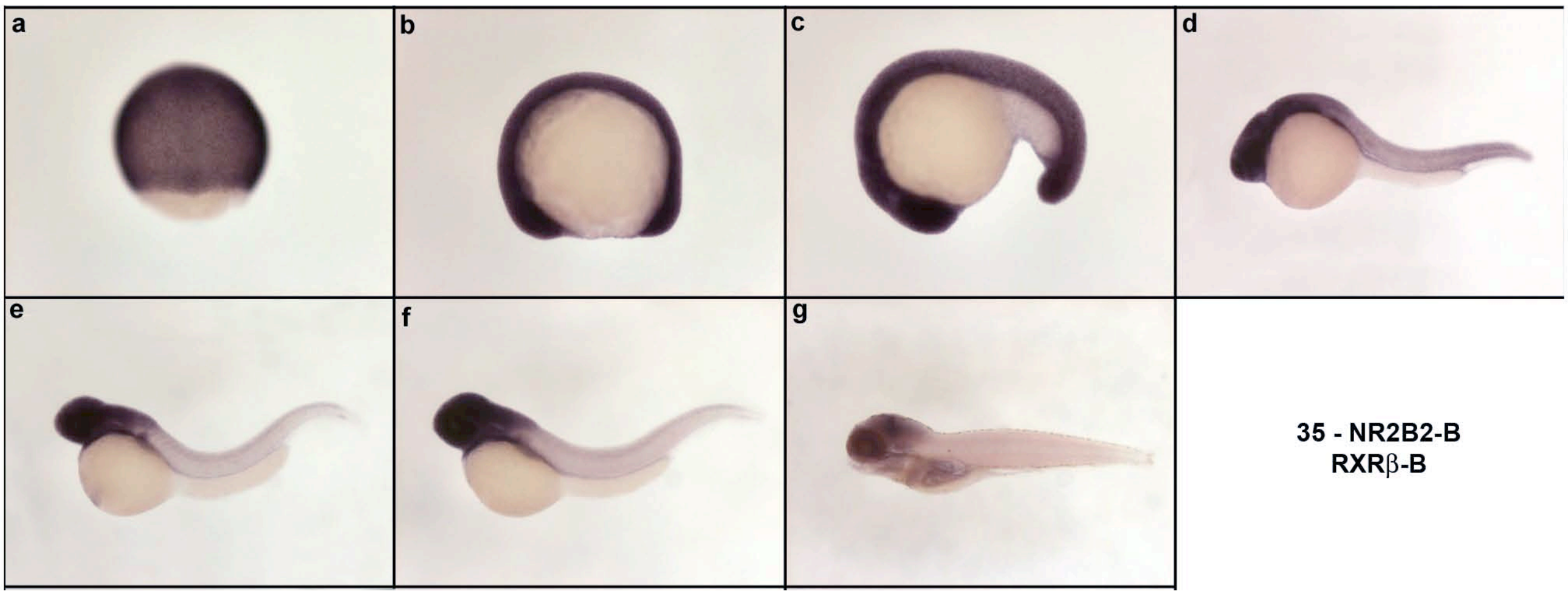

35 - NR2B2-B
RXRβ-B

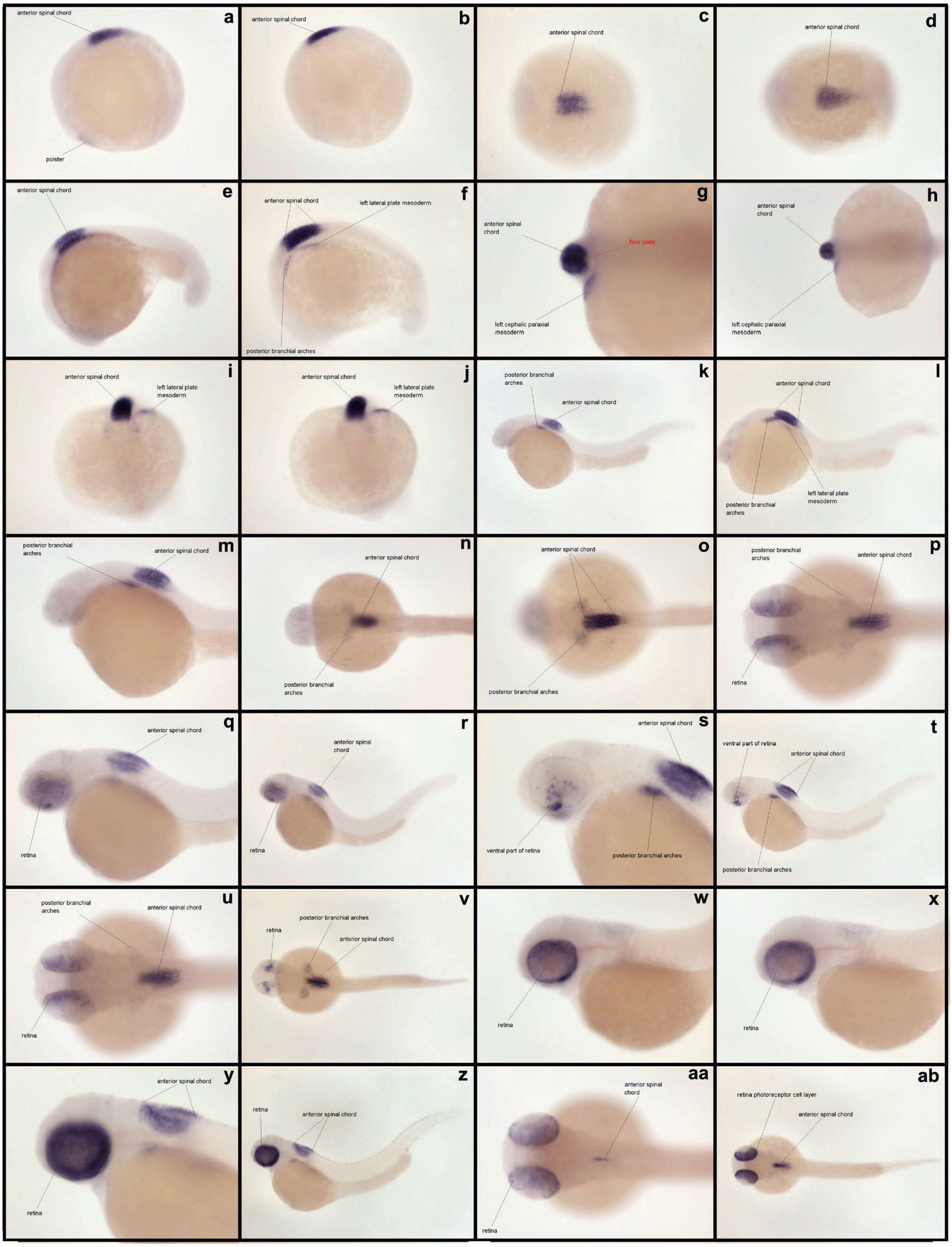

36 - NR2B3
RXRγ

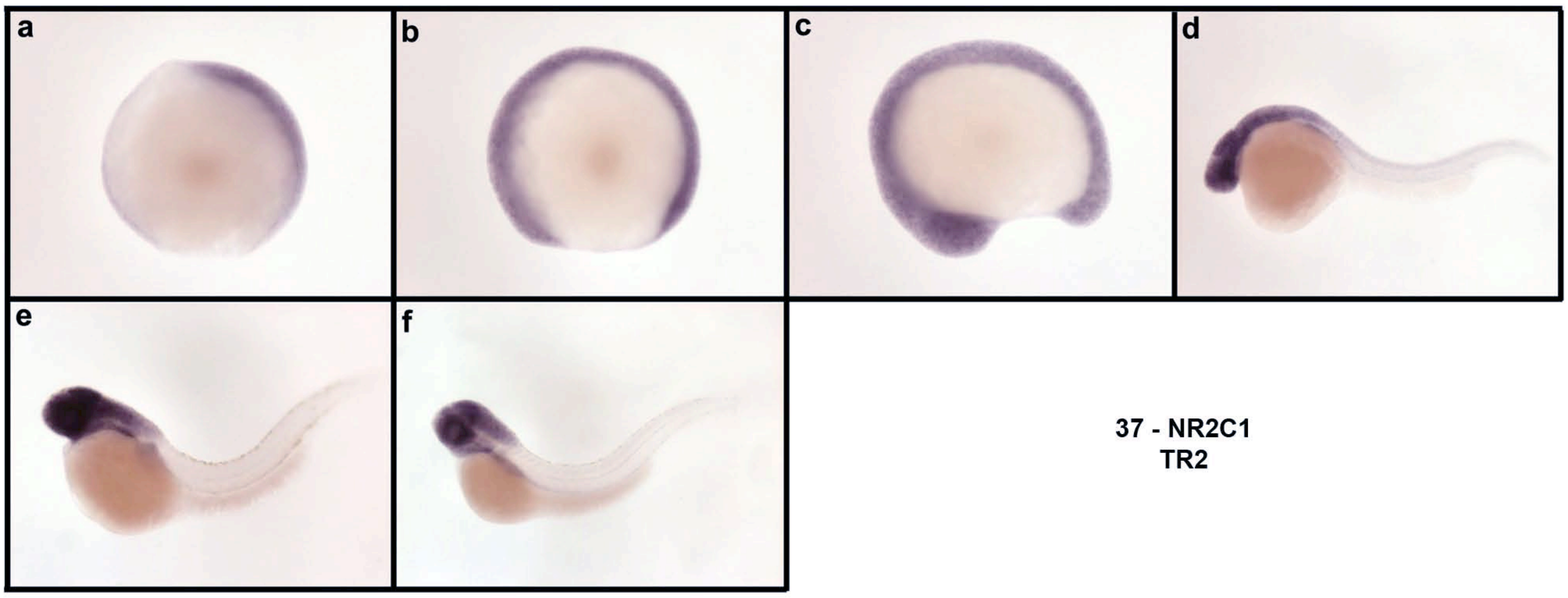

37 - NR2C1
TR2

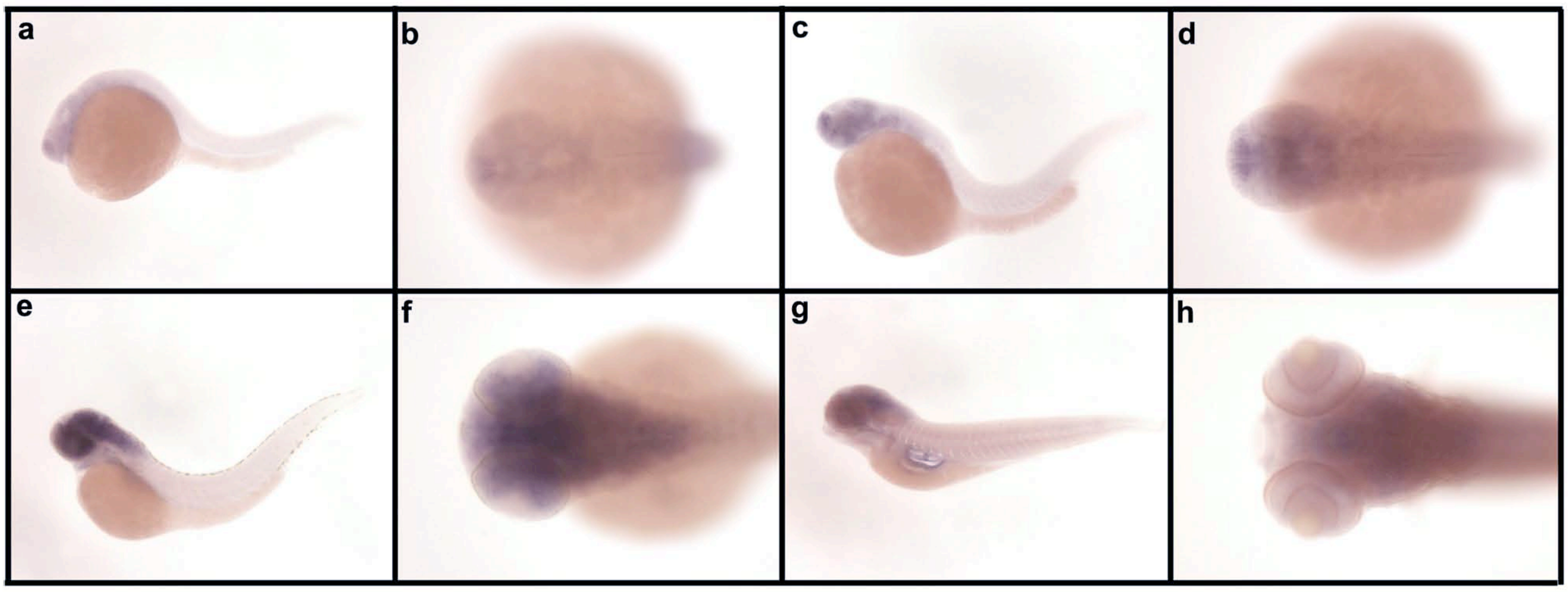

38 - NR2C2
TR4

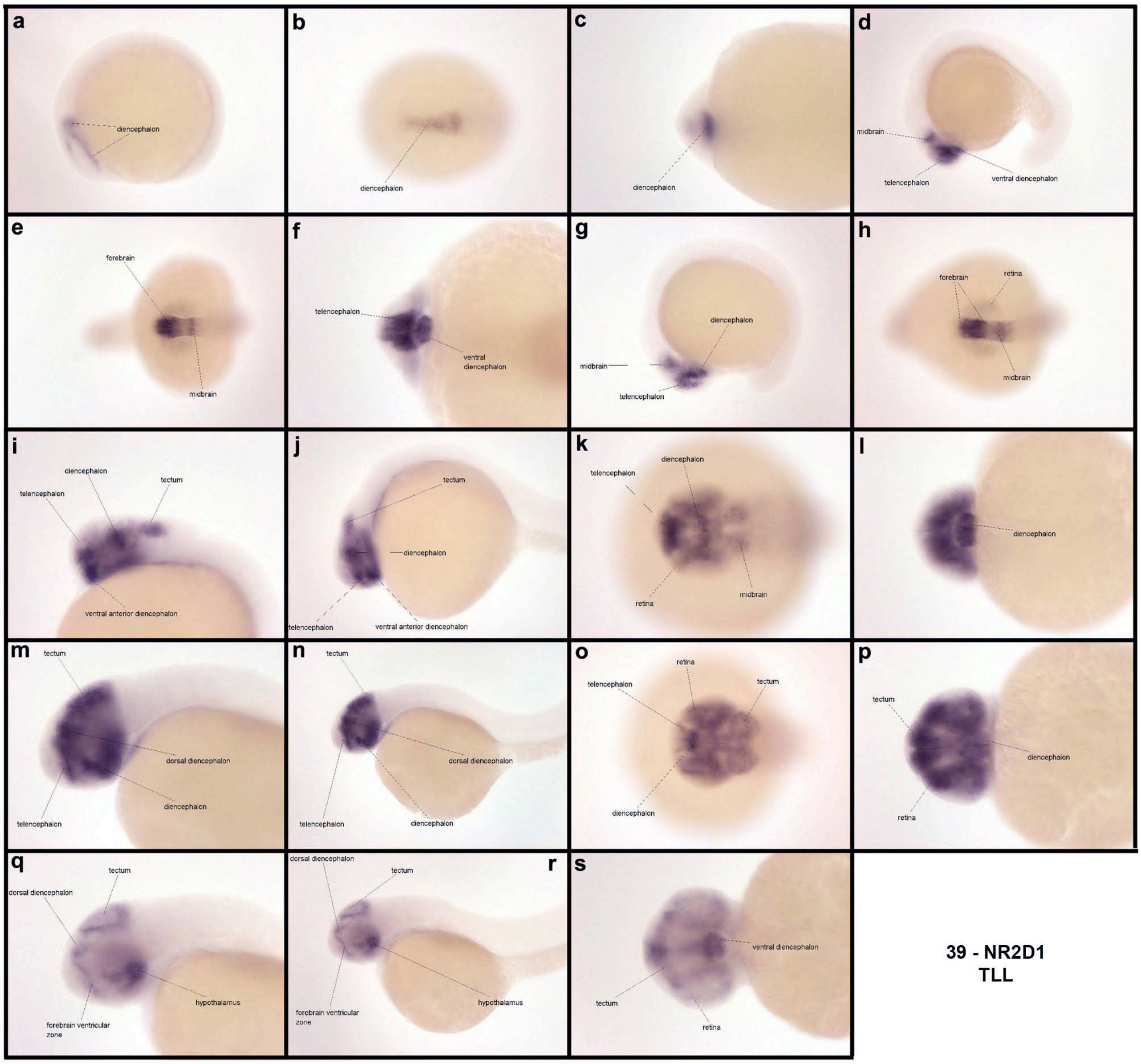

a
diencephalon
b
diencephalon
c
diencephalon
d
midbrain
telencephalon
ventral diencephalon
e
forebrain
midbrain
f
telencephalon
ventral diencephalon
g
diencephalon
midbrain
telencephalon
h
forebrain
retina
midbrain
i
diencephalon
tectum
telencephalon
ventral anterior diencephalon
j
tectum
diencephalon
telencephalon
ventral anterior diencephalon
k
diencephalon
telencephalon
retina
midbrain
l
diencephalon
m
tectum
dorsal diencephalon
diencephalon
telencephalon
n
tectum
dorsal diencephalon
telencephalon
diencephalon
o
retina
telencephalon
tectum
diencephalon
p
tectum
diencephalon
retina
q
tectum
dorsal diencephalon
hypothalamus
forebrain ventricular zone
r
dorsal diencephalon
tectum
hypothalamus
forebrain ventricular zone
s
ventral diencephalon
tectum
retina

39 - NR2D1
TLL

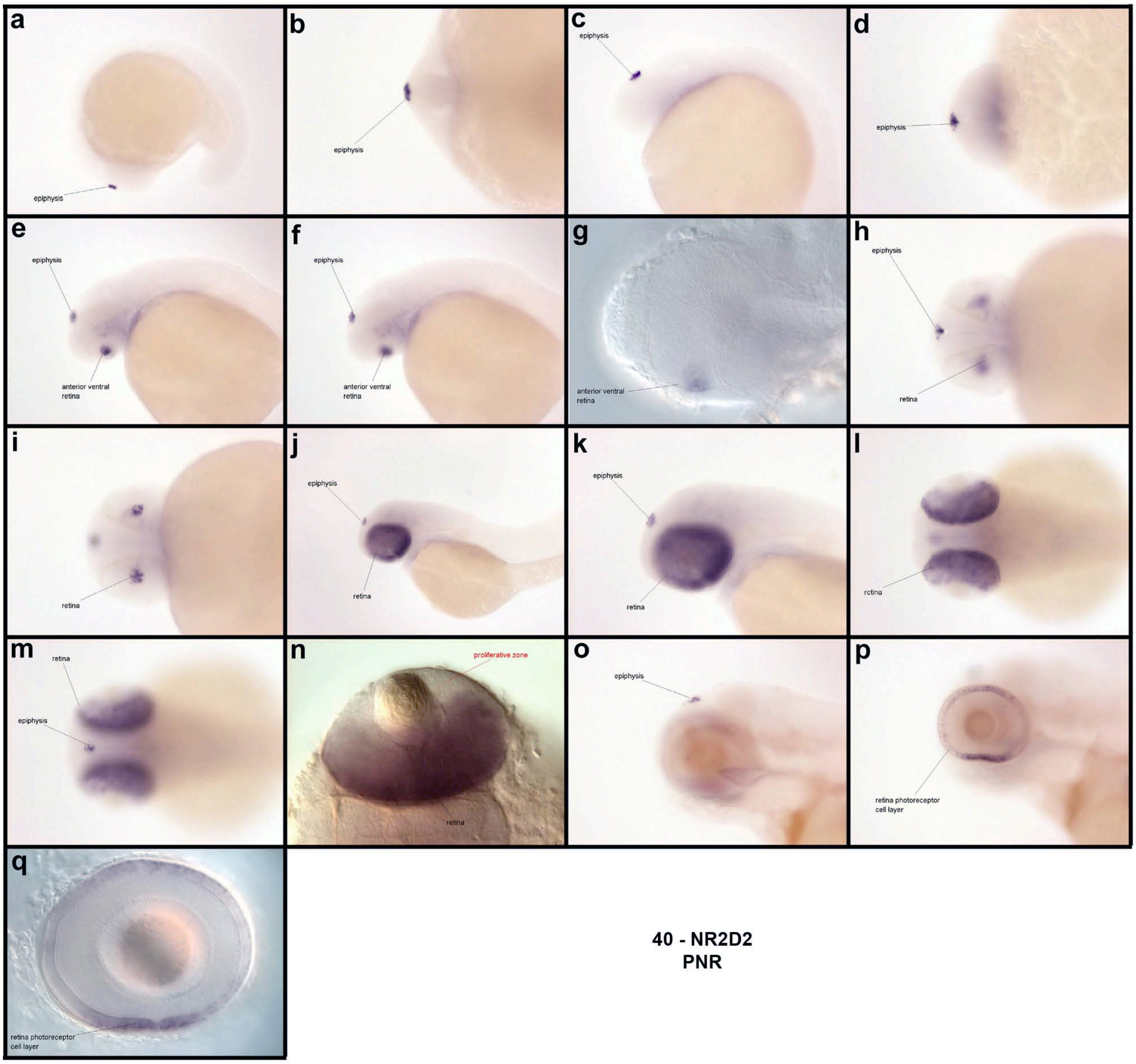
a
epiphysis
b
epiphysis
c
epiphysis
d
epiphysis
e
epiphysis
anterior ventral retina
f
epiphysis
anterior ventral retina
g
anterior ventral retina
h
epiphysis
retina
i
retina
j
epiphysis
retina
k
epiphysis
retina
l
retina
m
retina
epiphysis
n
proliferative zone
retina
o
epiphysis
p
retina photoreceptor cell layer
q
retina photoreceptor cell layer

40 - NR2D2
PNR

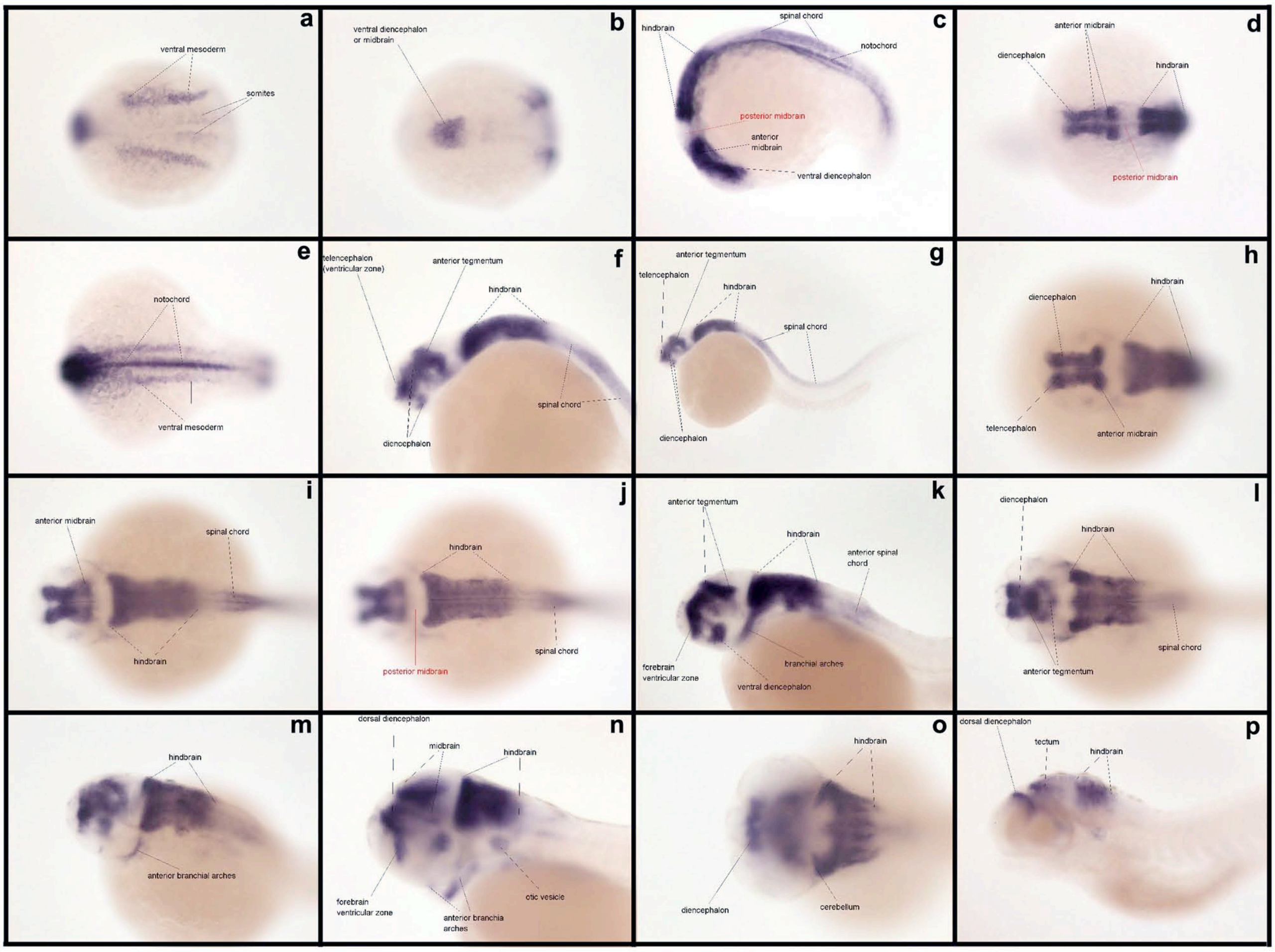

41 - NR2F1-A
COUPTFα-A

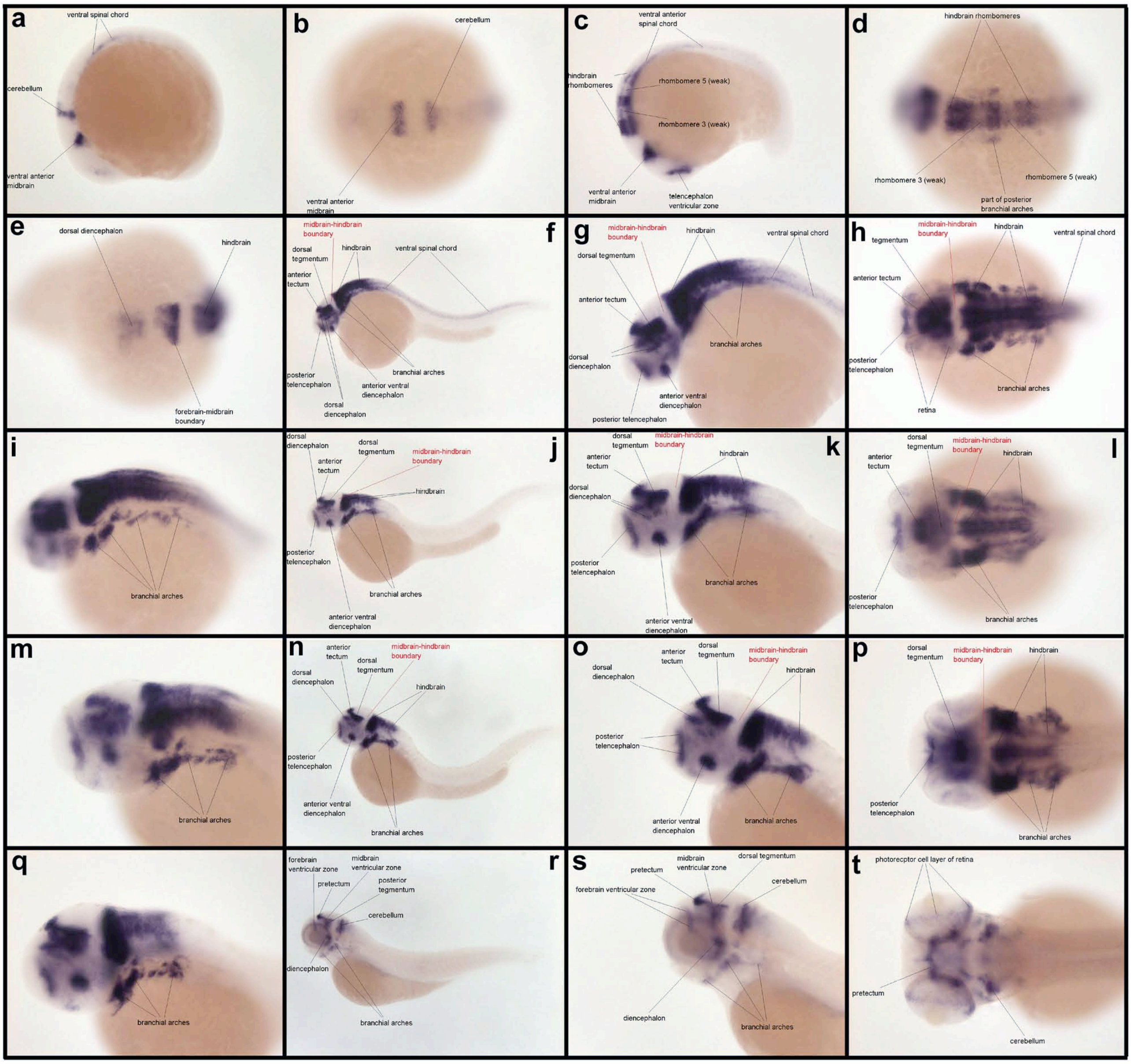

42 - NR2F1-B
COUPTFα-B

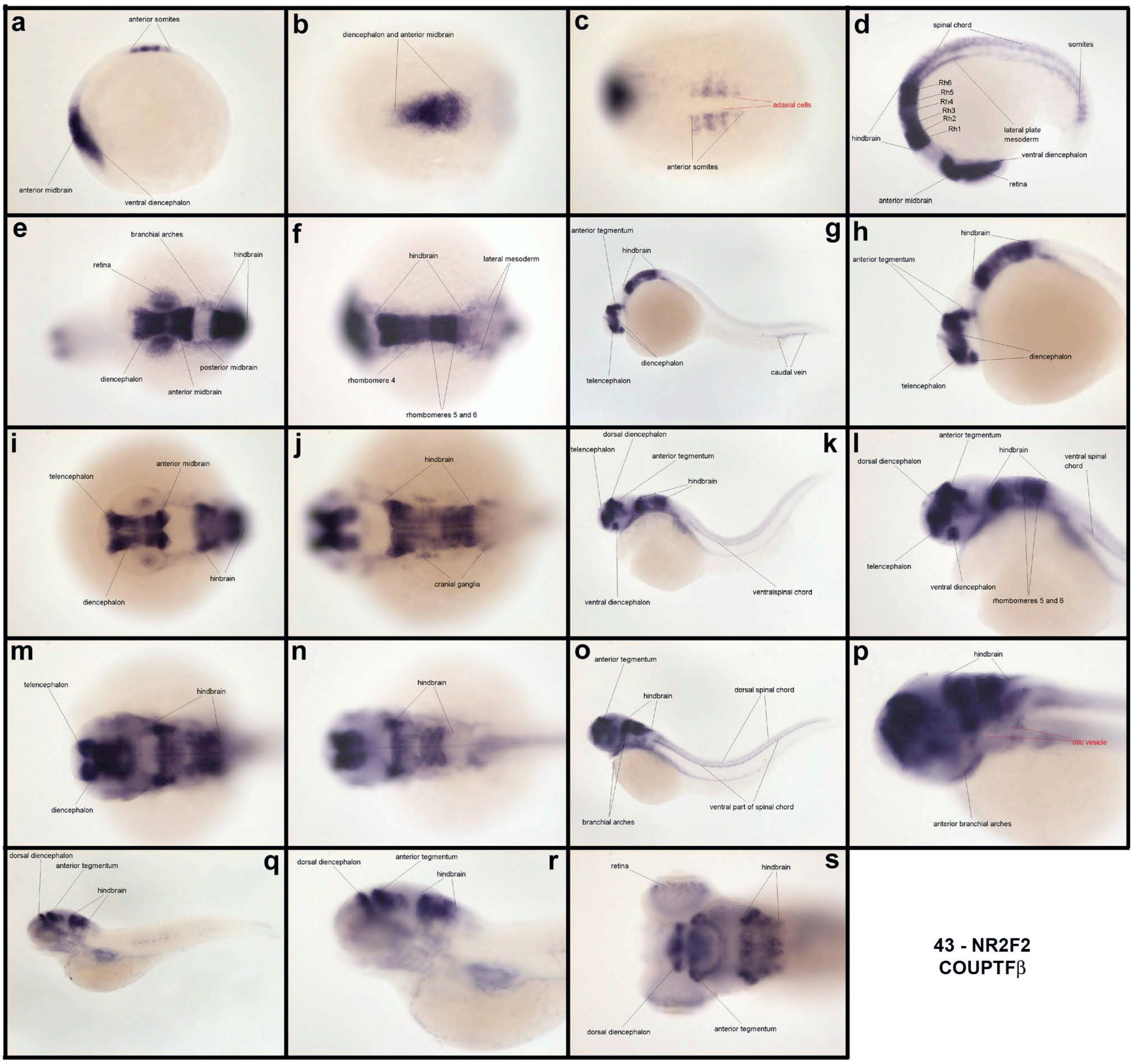

43 - NR2F2
COUPTFβ

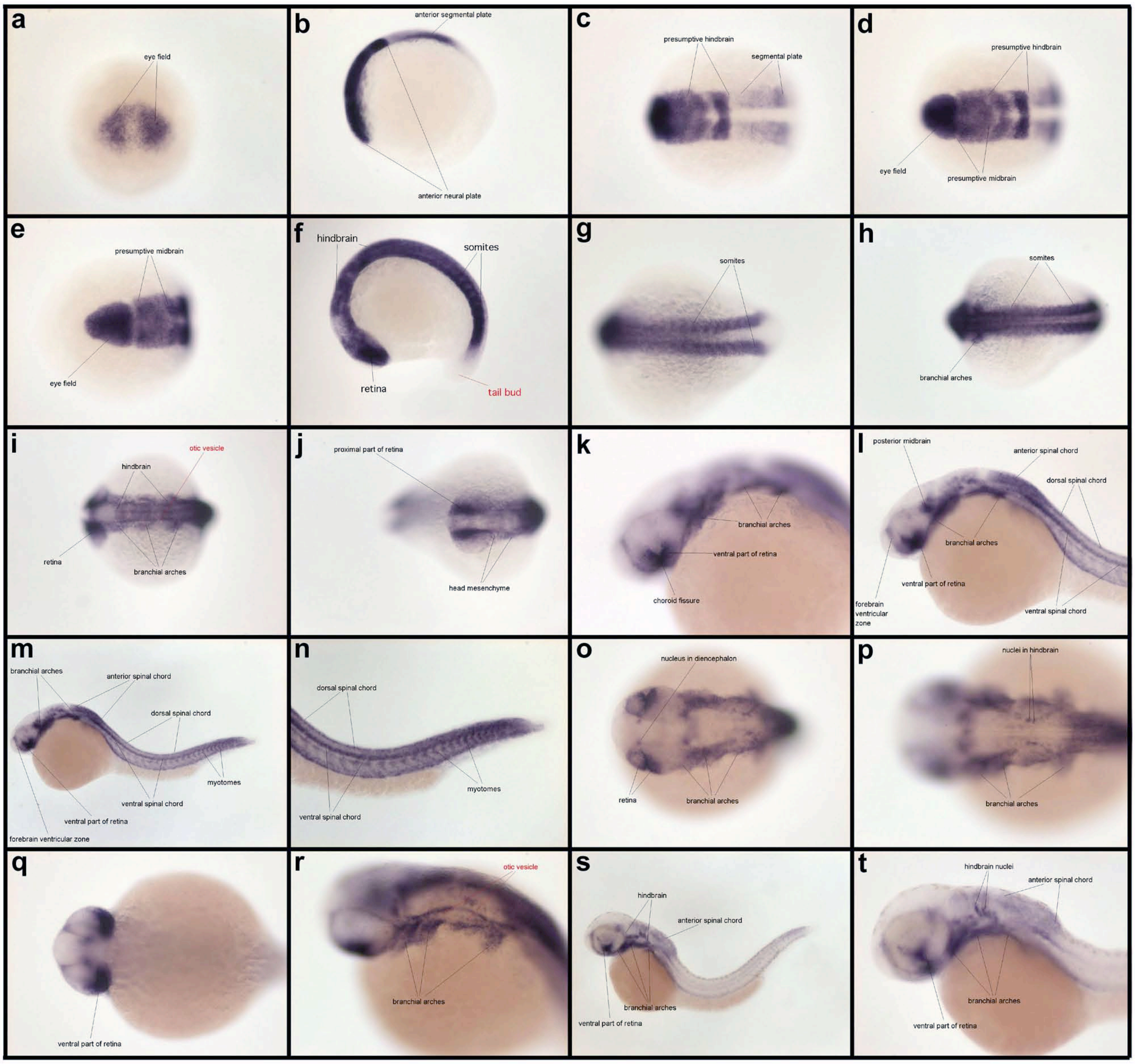

44 - NR2F5
COUPTFγ

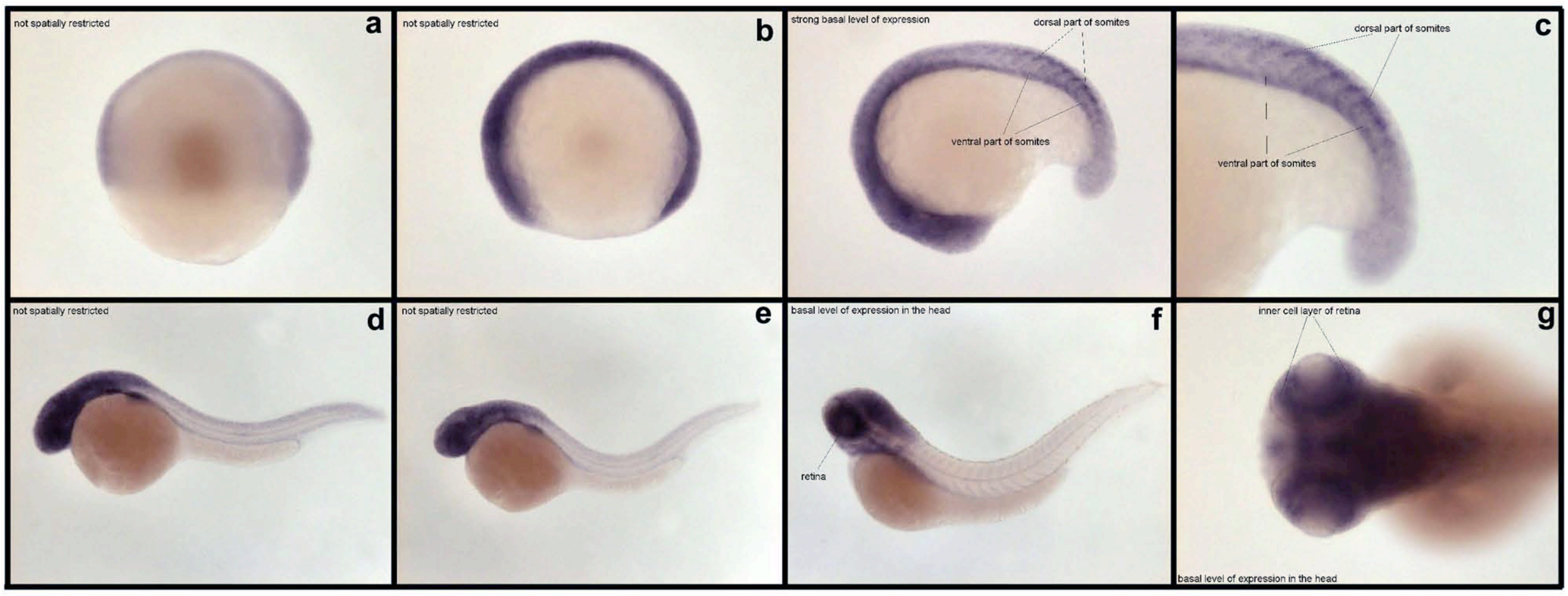

45 - NR2F6-A
EAR2-A

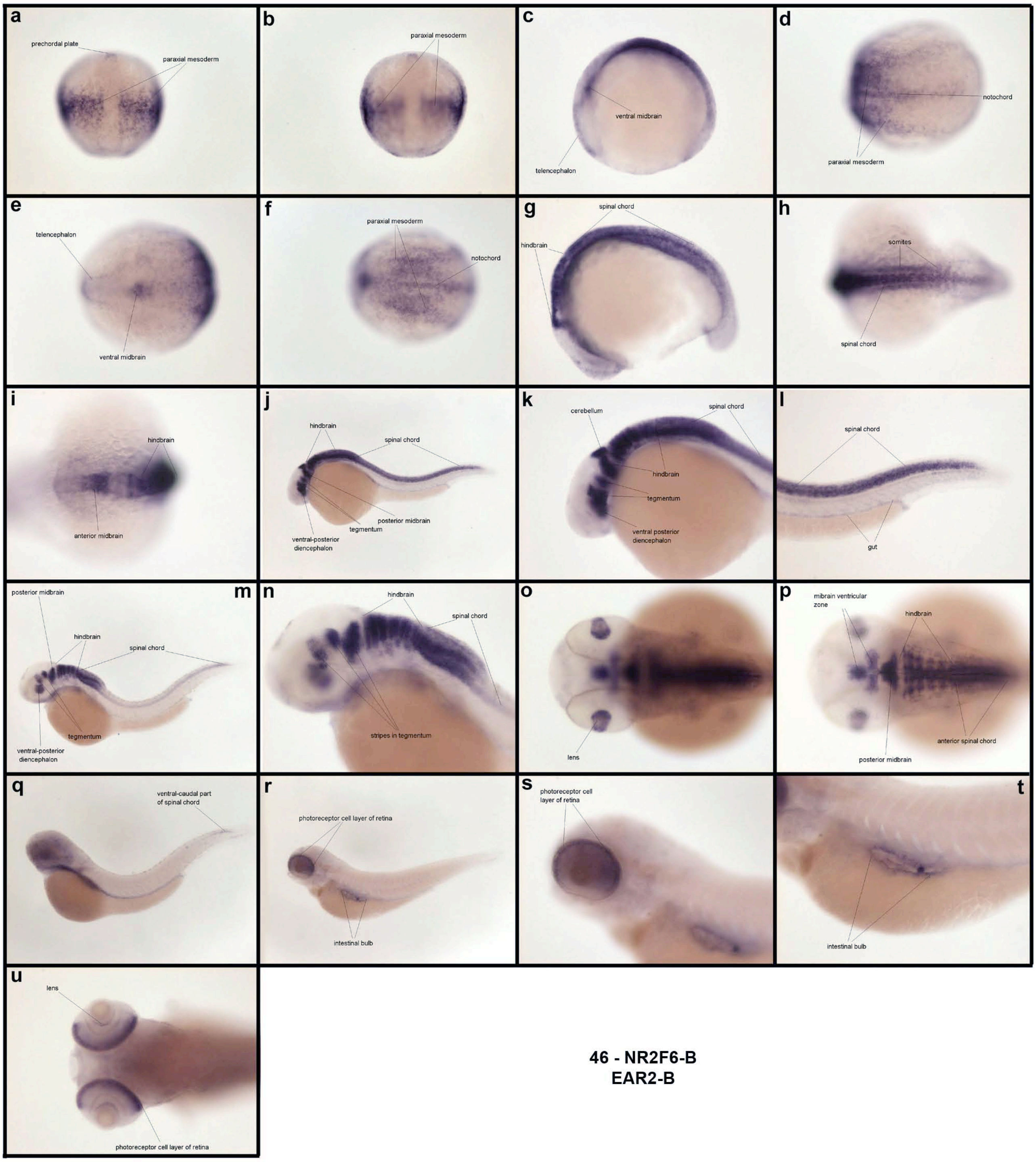

46 - NR2F6-B
EAR2-B

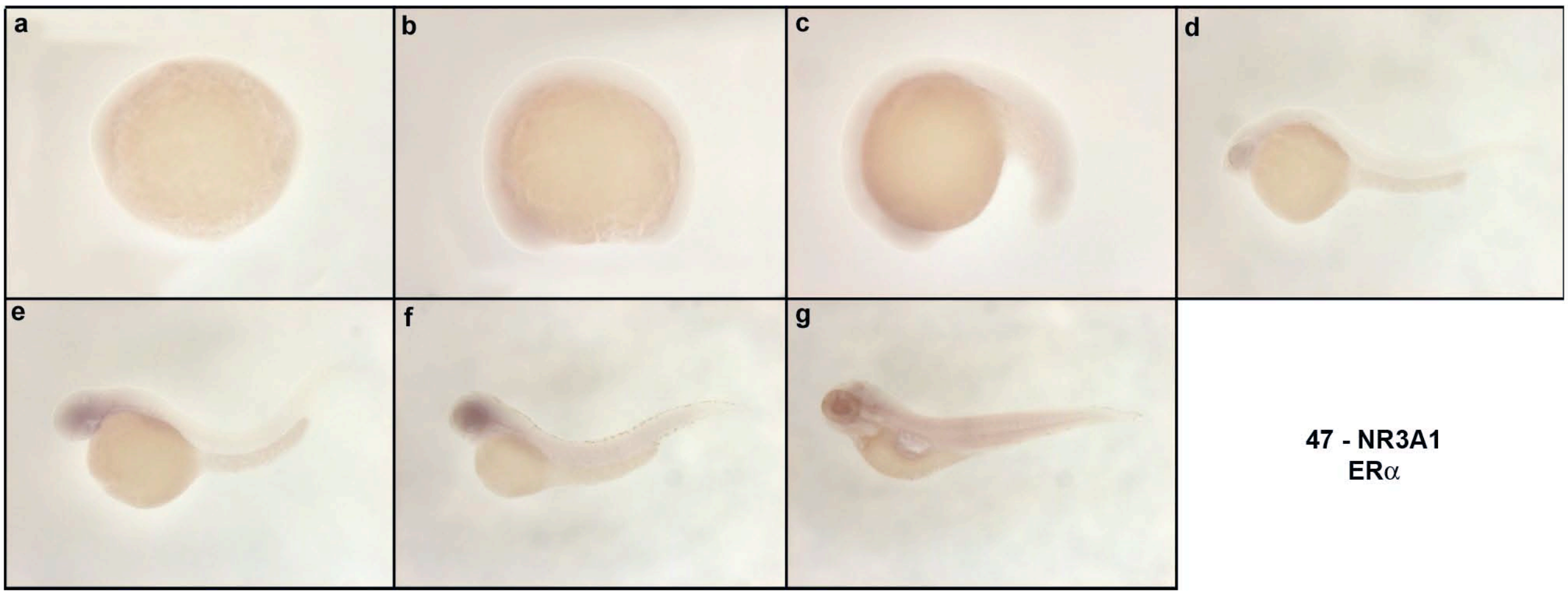

47 - NR3A1
ERα

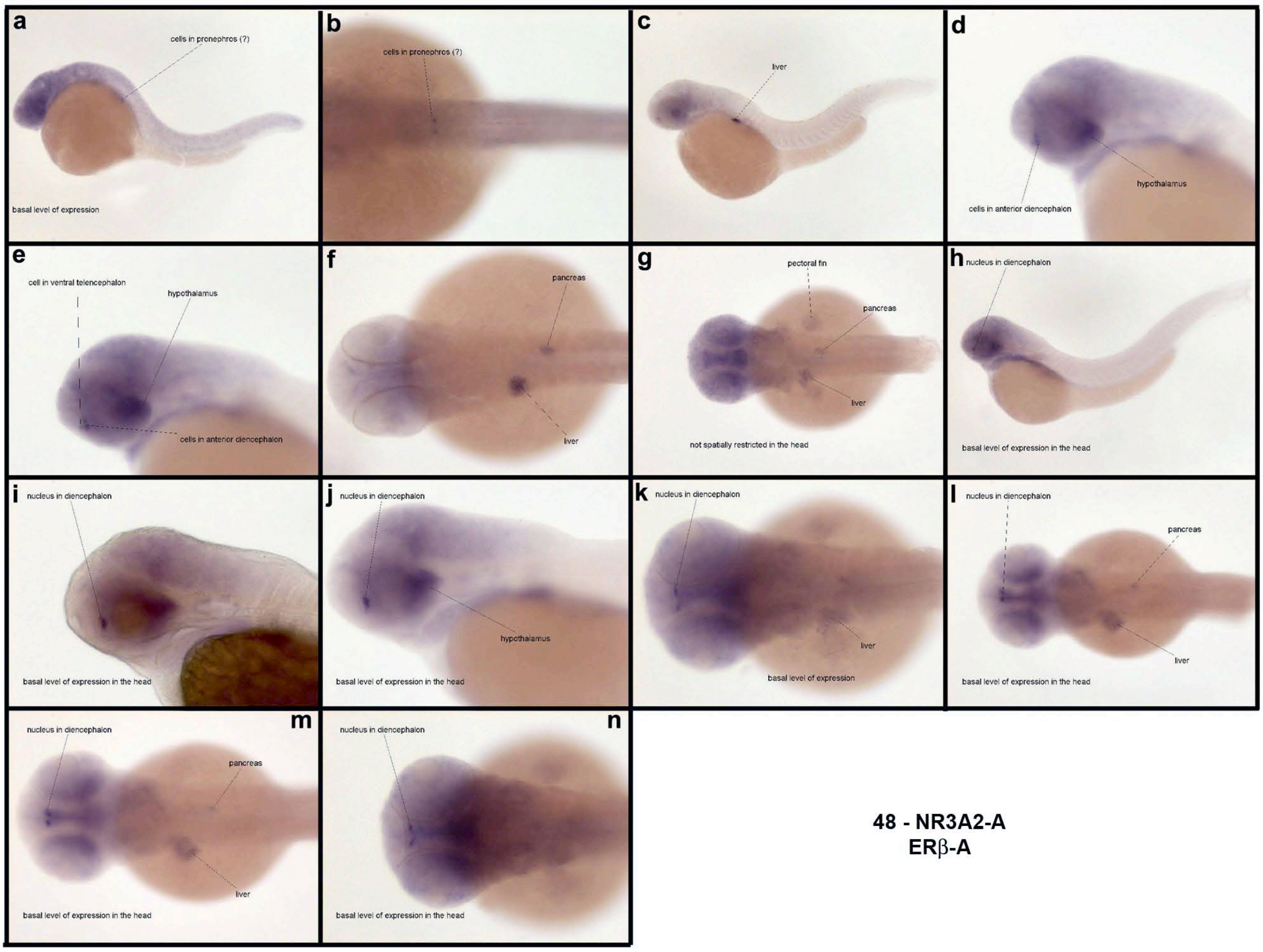

48 - NR3A2-A
ERβ-A

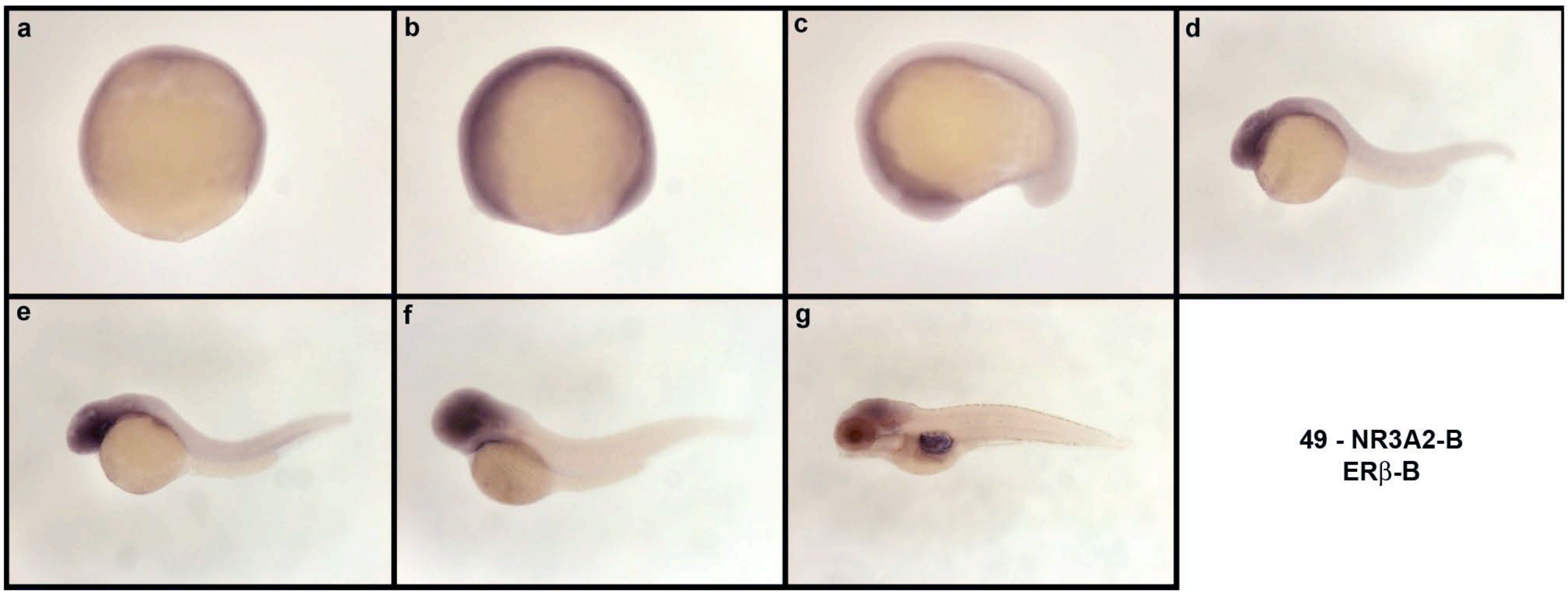

49 - NR3A2-B
ERβ-B

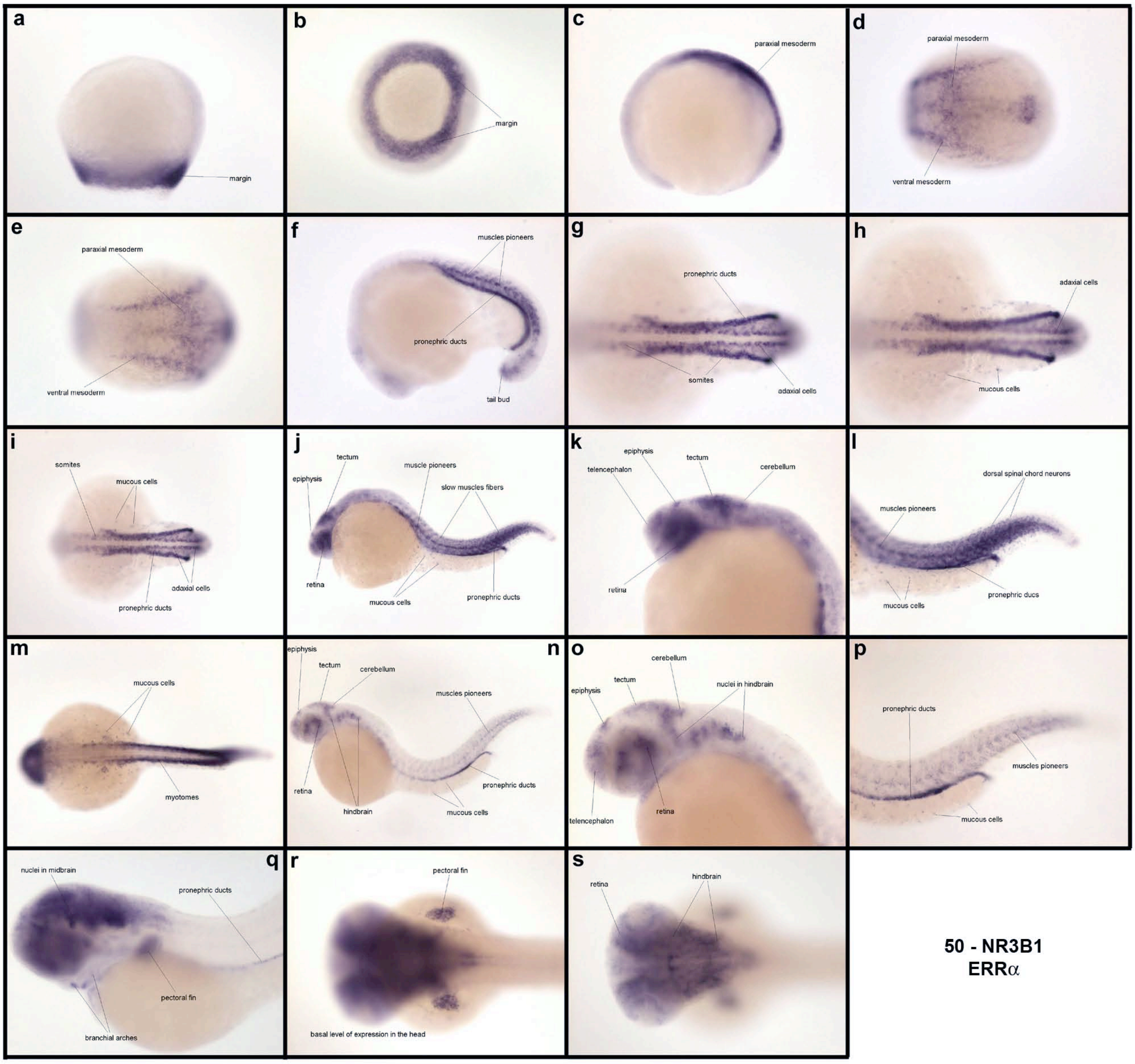

50 - NR3B1
ERRα

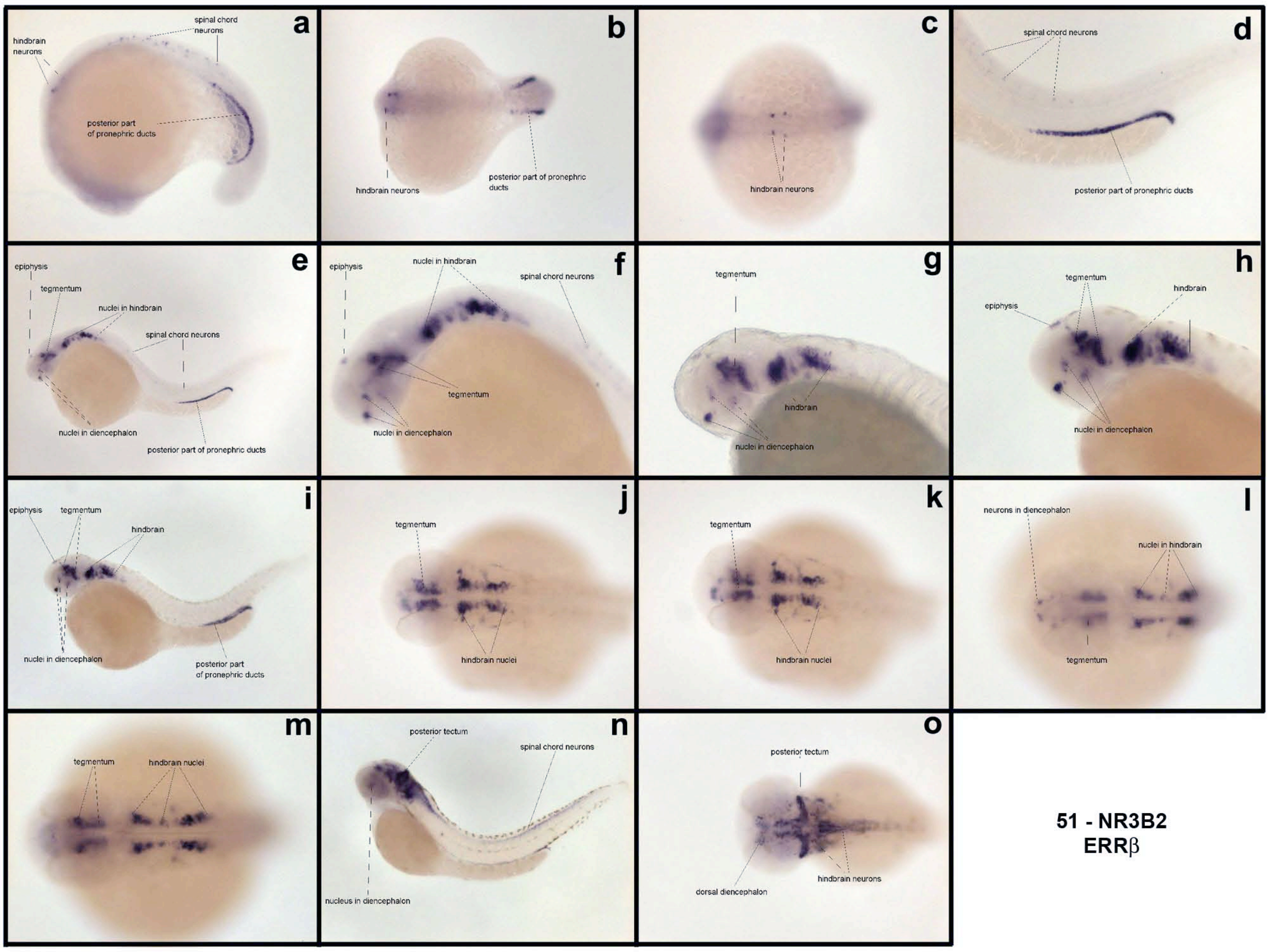

51 - NR3B2
ERRβ

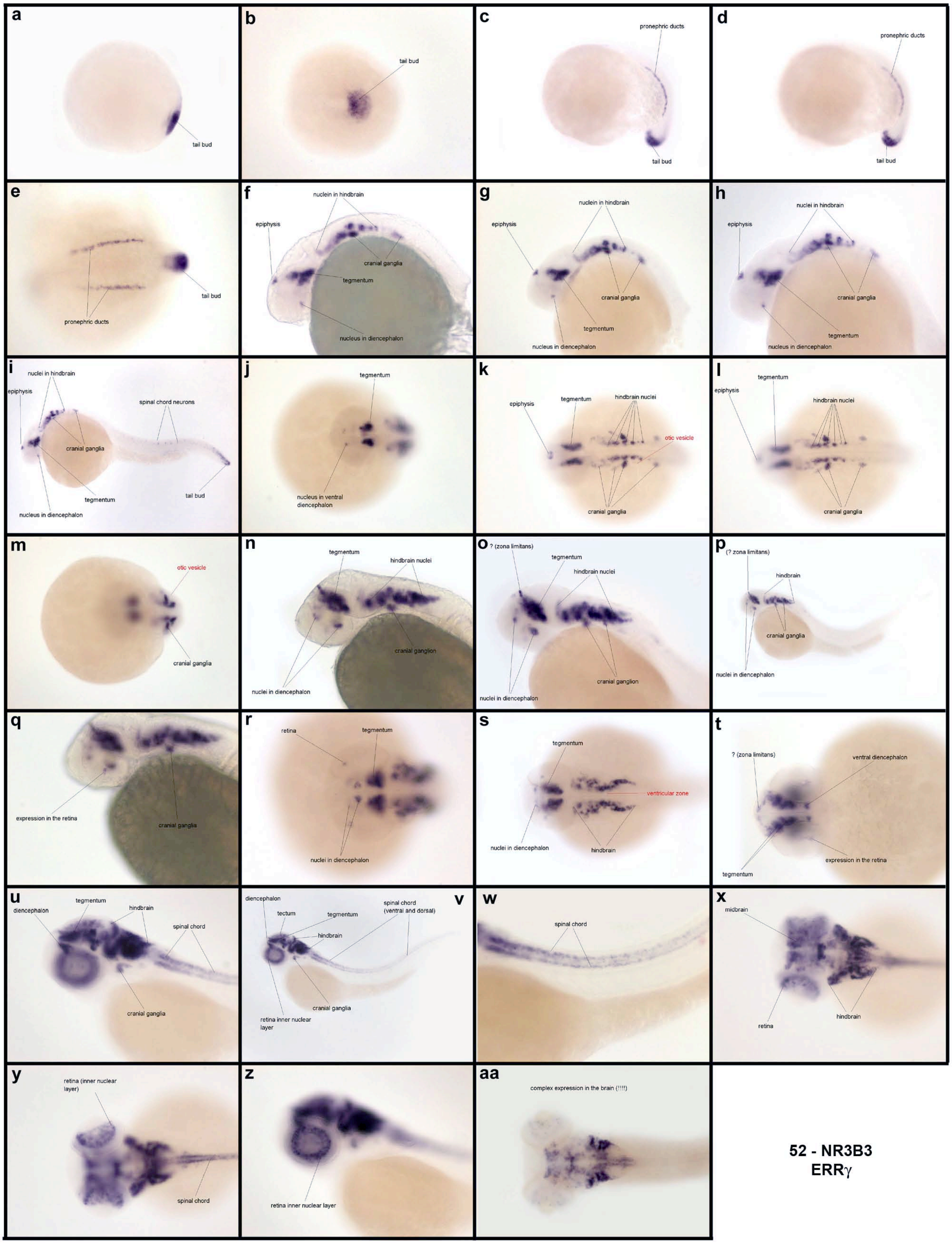

52 - NR3B3
ERRγ

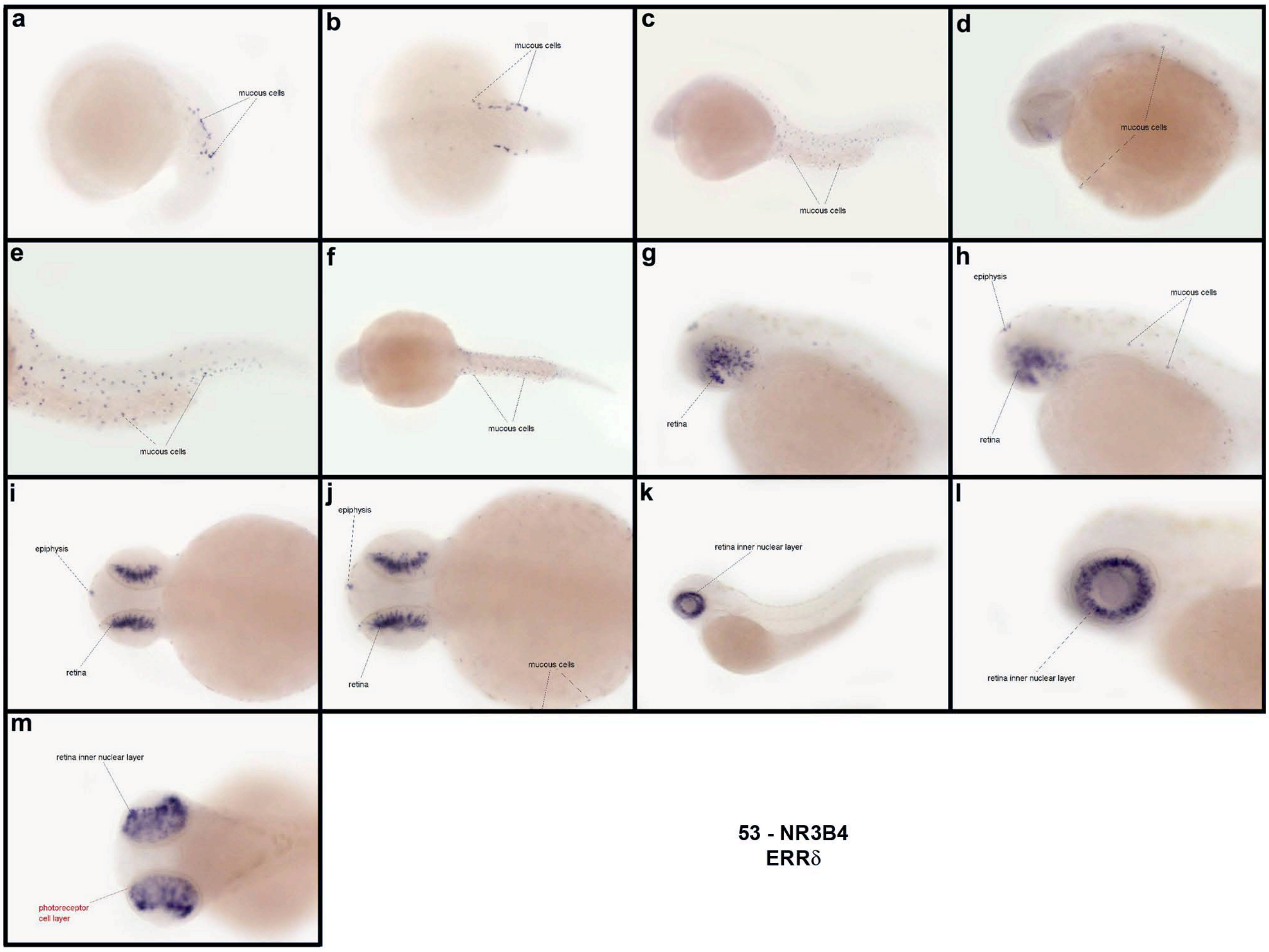

53 - NR3B4
ERRδ

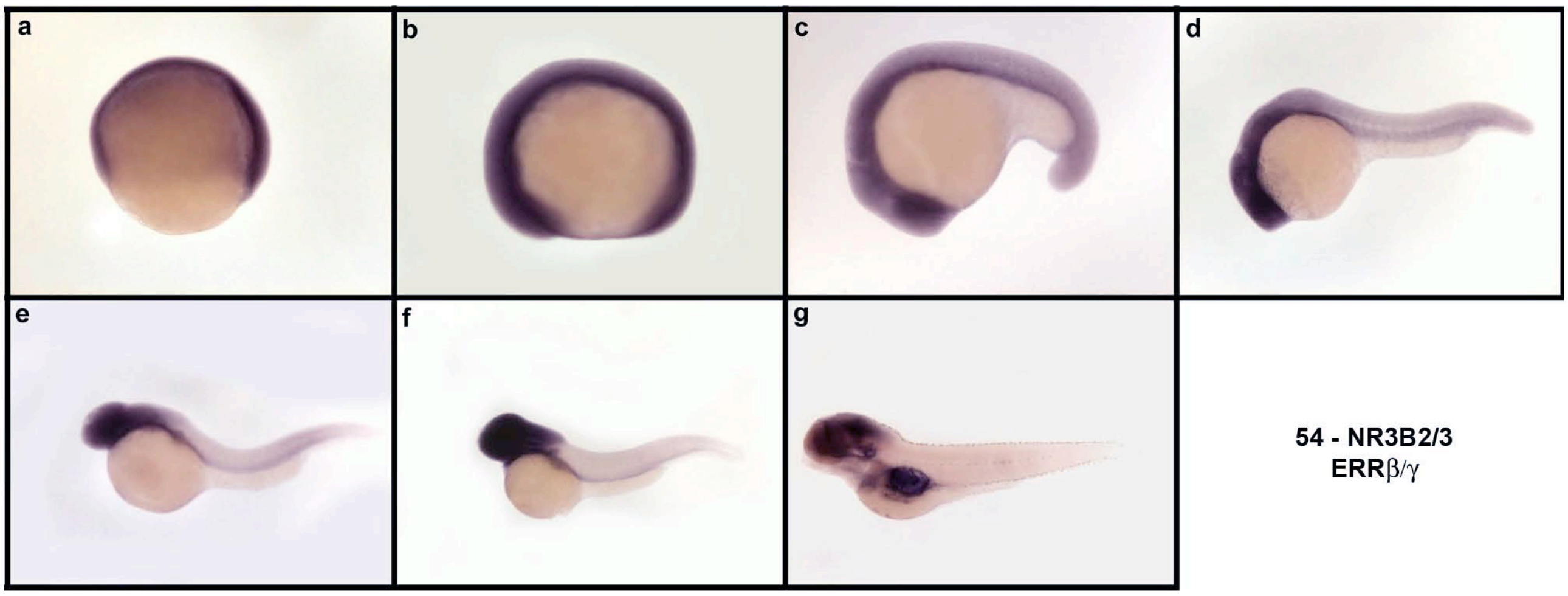

54 - NR3B2/3
ERRβ/γ

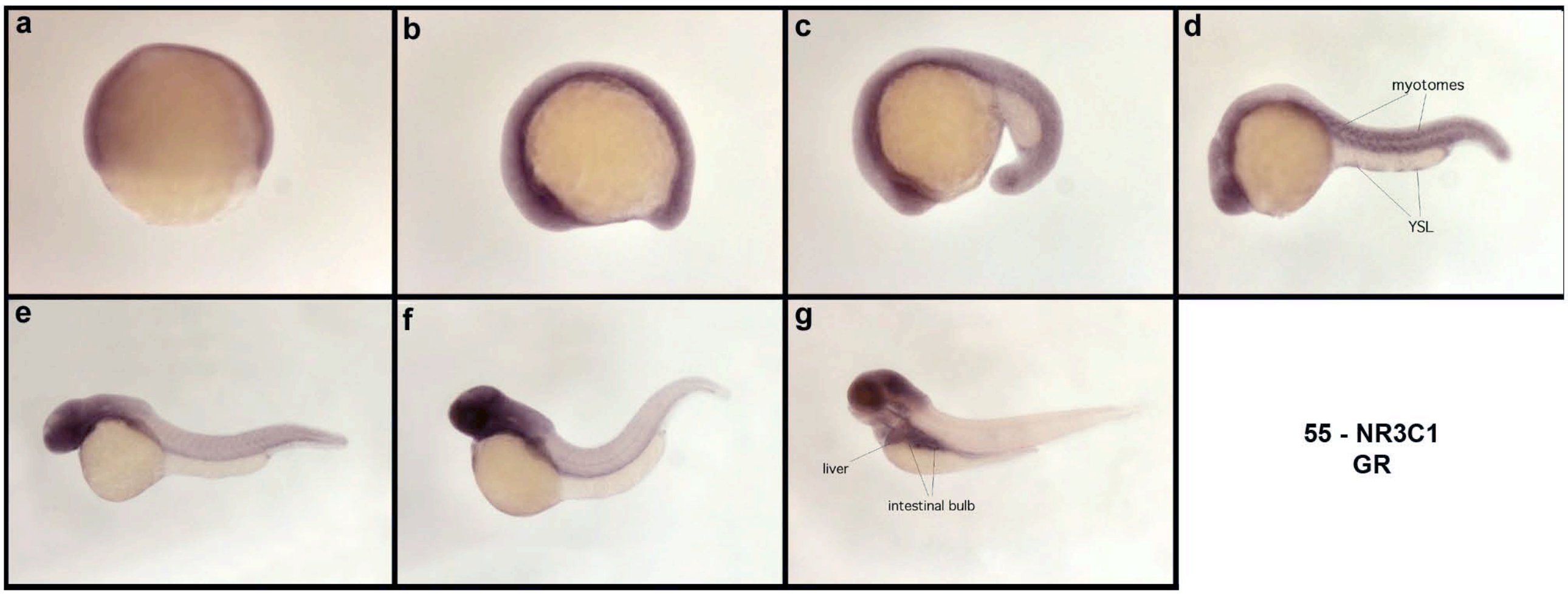

55 - NR3C1
GR

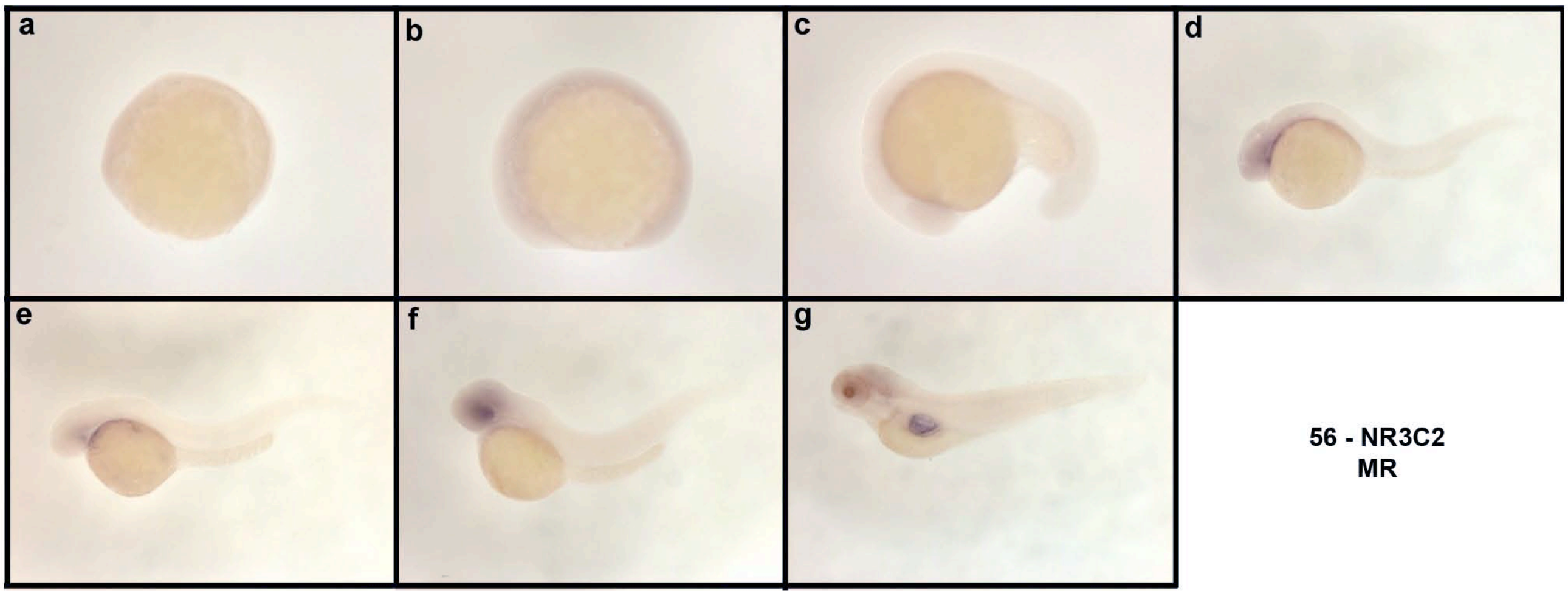

56 - NR3C2
MR

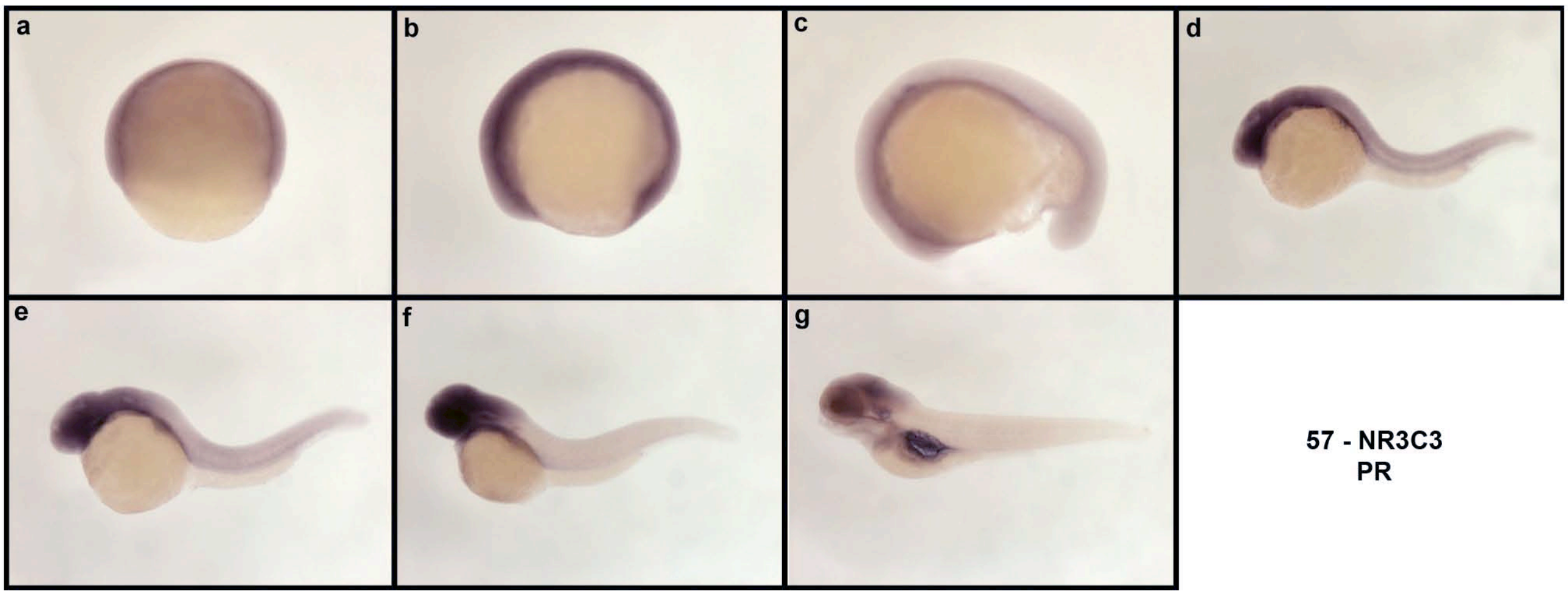

57 - NR3C3
PR

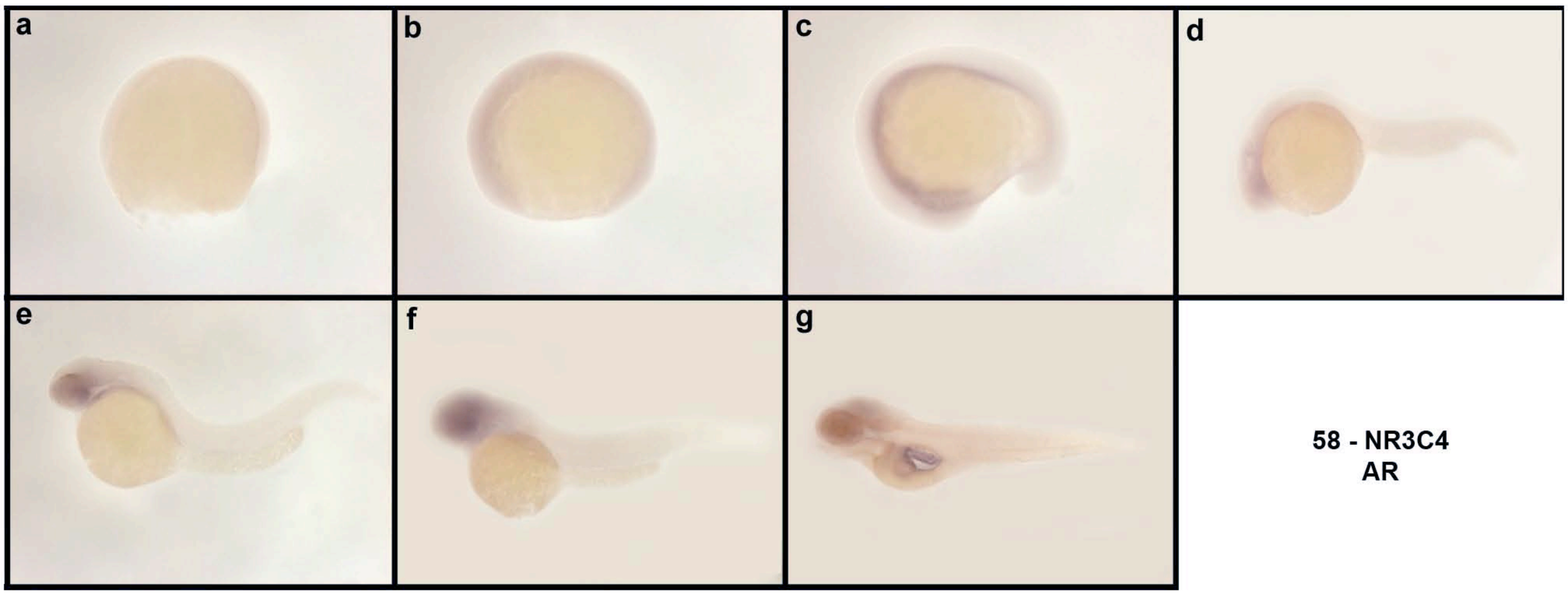

58 - NR3C4
AR

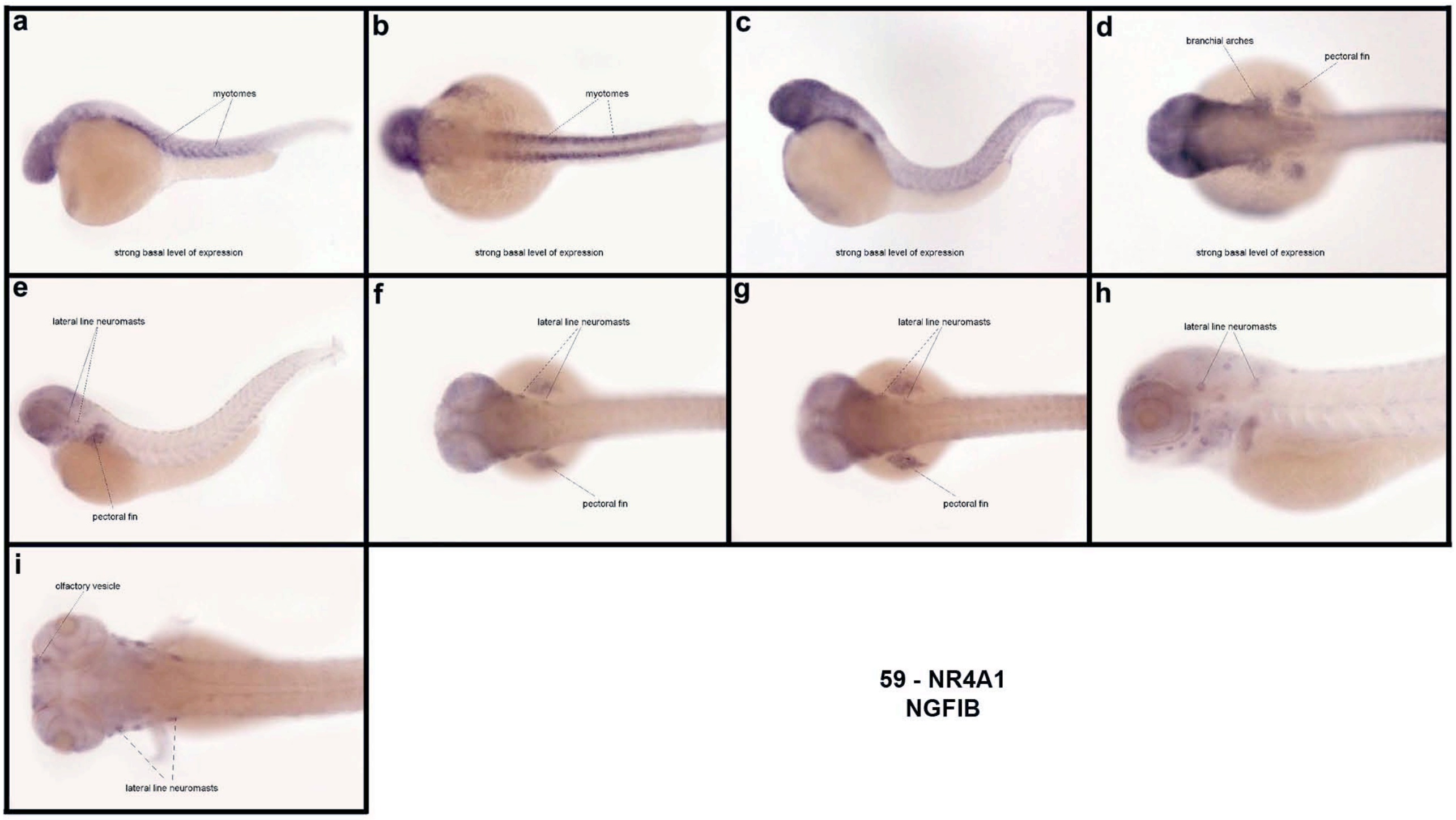

59 - NR4A1
NGFIB

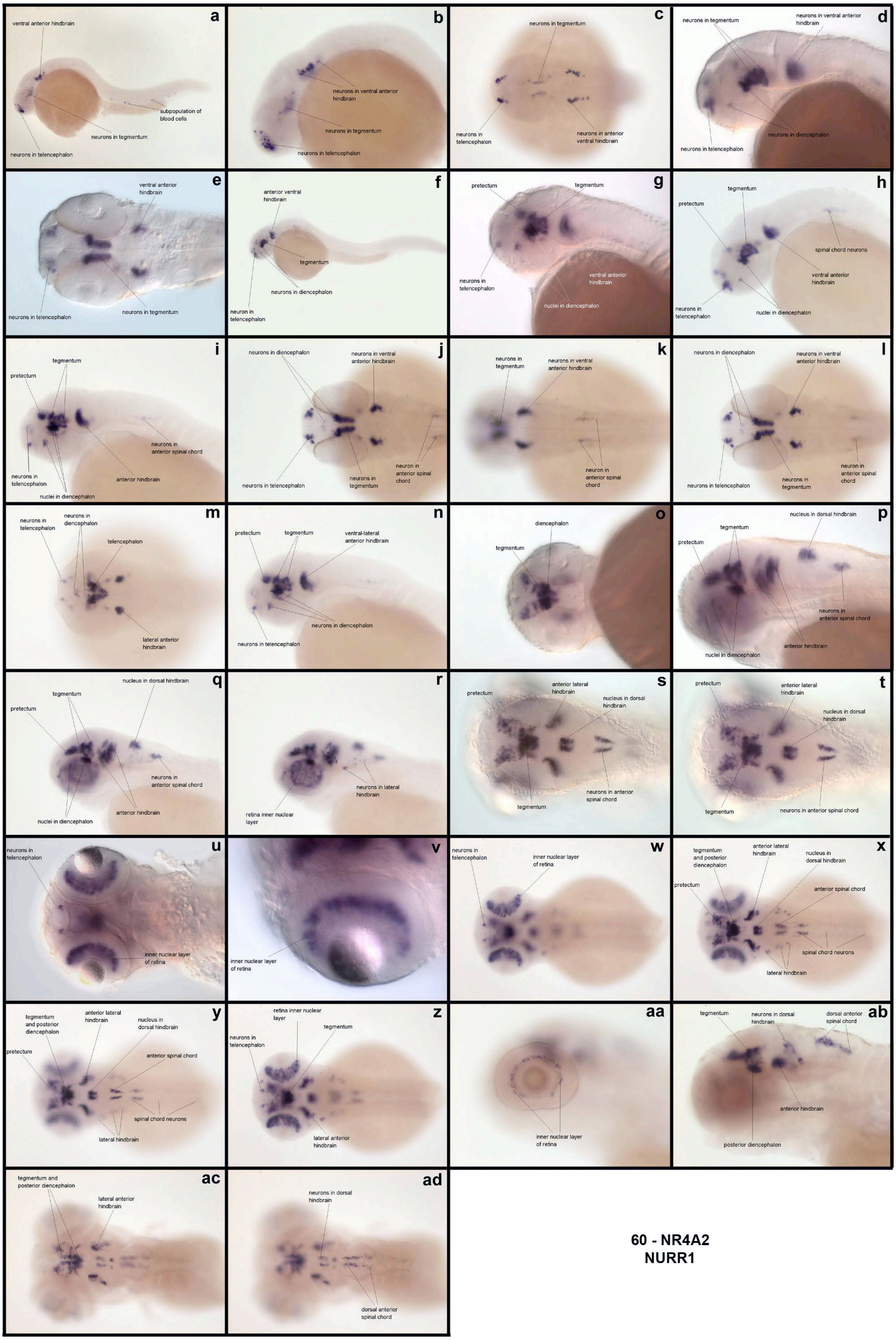

60 - NR4A2
NURR1

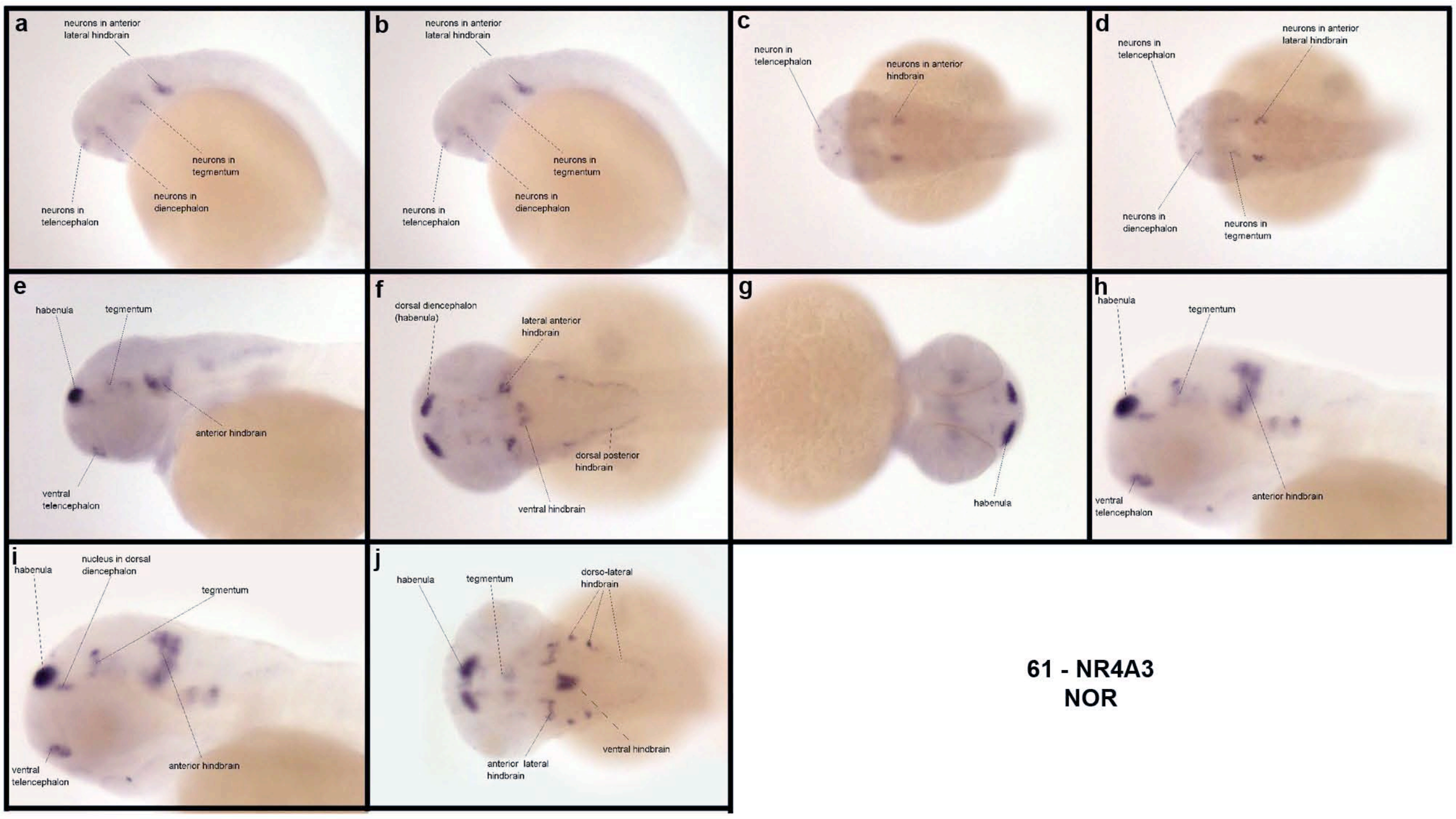
a
neurons in anterior lateral hindbrain
neurons in tegmentum
neurons in diencephalon
neurons in telencephalon
b
neurons in anterior lateral hindbrain
neurons in tegmentum
neurons in diencephalon
neurons in telencephalon
c
neuron in telencephalon
neurons in anterior hindbrain
d
neurons in telencephalon
neurons in anterior lateral hindbrain
neurons in diencephalon
neurons in tegmentum
e
habenula
tegmentum
anterior hindbrain
ventral telencephalon
f
dorsal diencephalon (habenula)
lateral anterior hindbrain
dorsal posterior hindbrain
ventral hindbrain
g
habenula
h
habenula
tegmentum
anterior hindbrain
ventral telencephalon
i
habenula
nucleus in dorsal diencephalon
tegmentum
anterior hindbrain
ventral telencephalon
j
habenula
tegmentum
dorso-lateral hindbrain
ventral hindbrain
anterior lateral hindbrain

61 - NR4A3
NOR

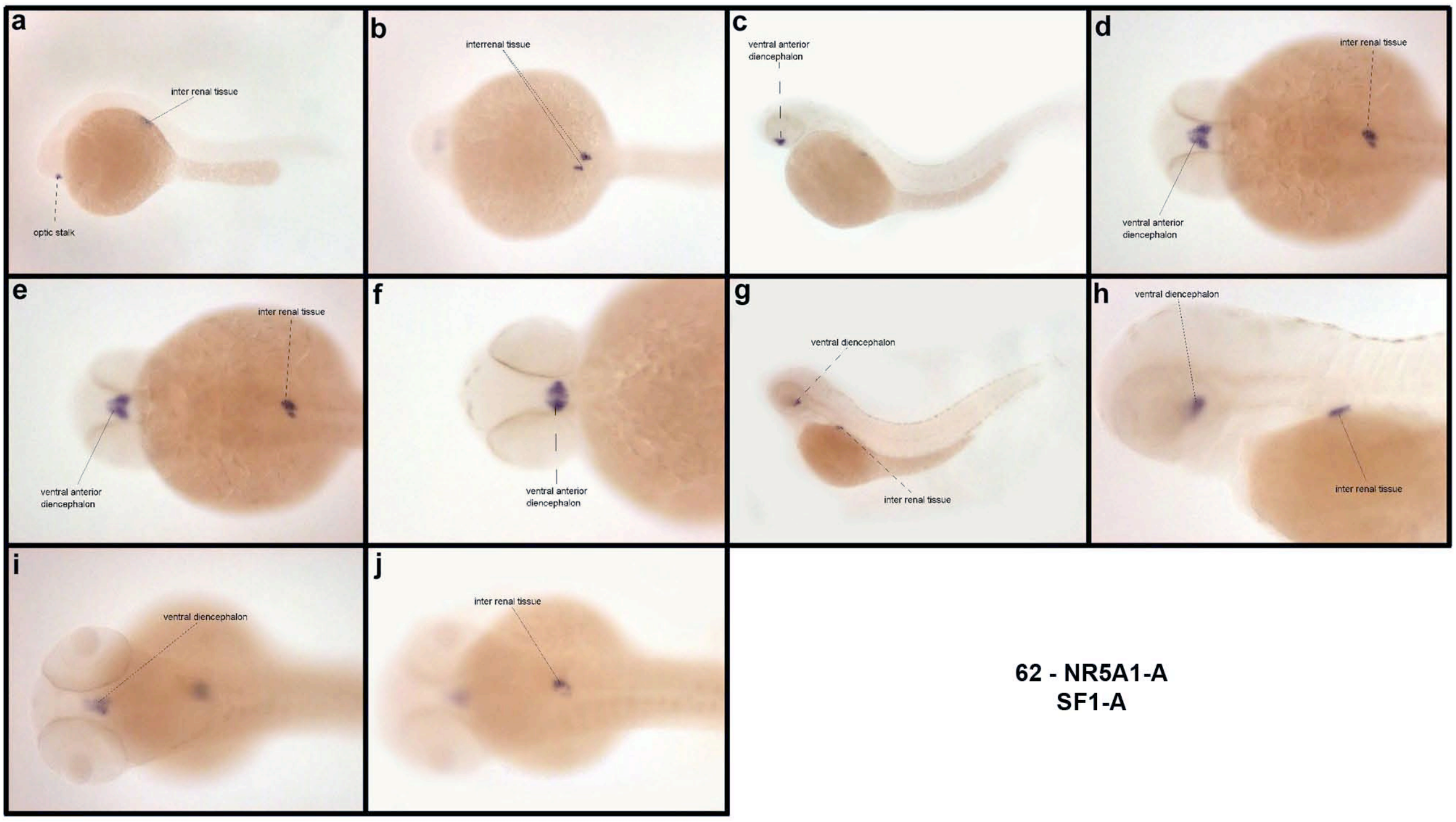

62 - NR5A1-A
SF1-A

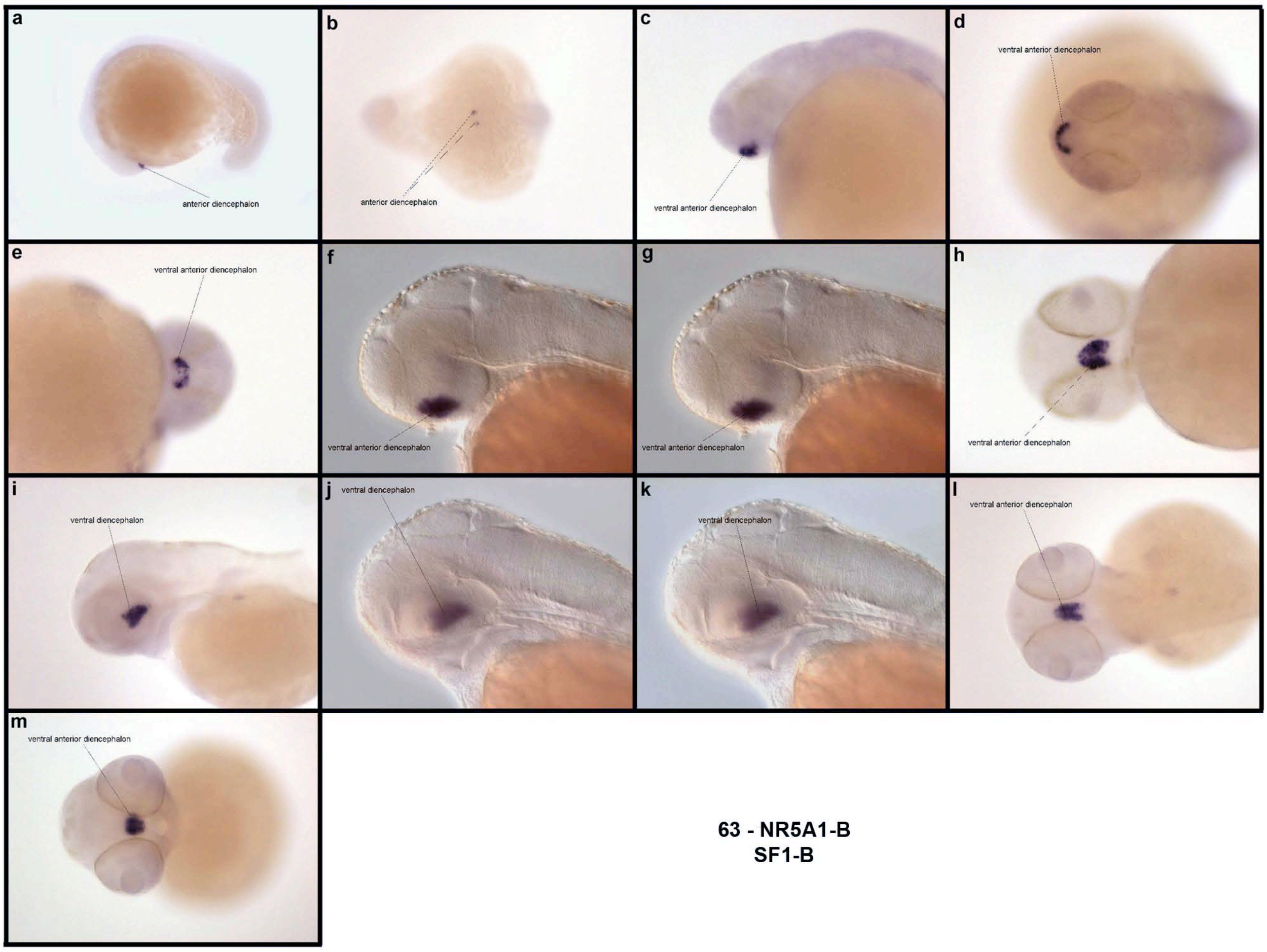

63 - NR5A1-B
SF1-B

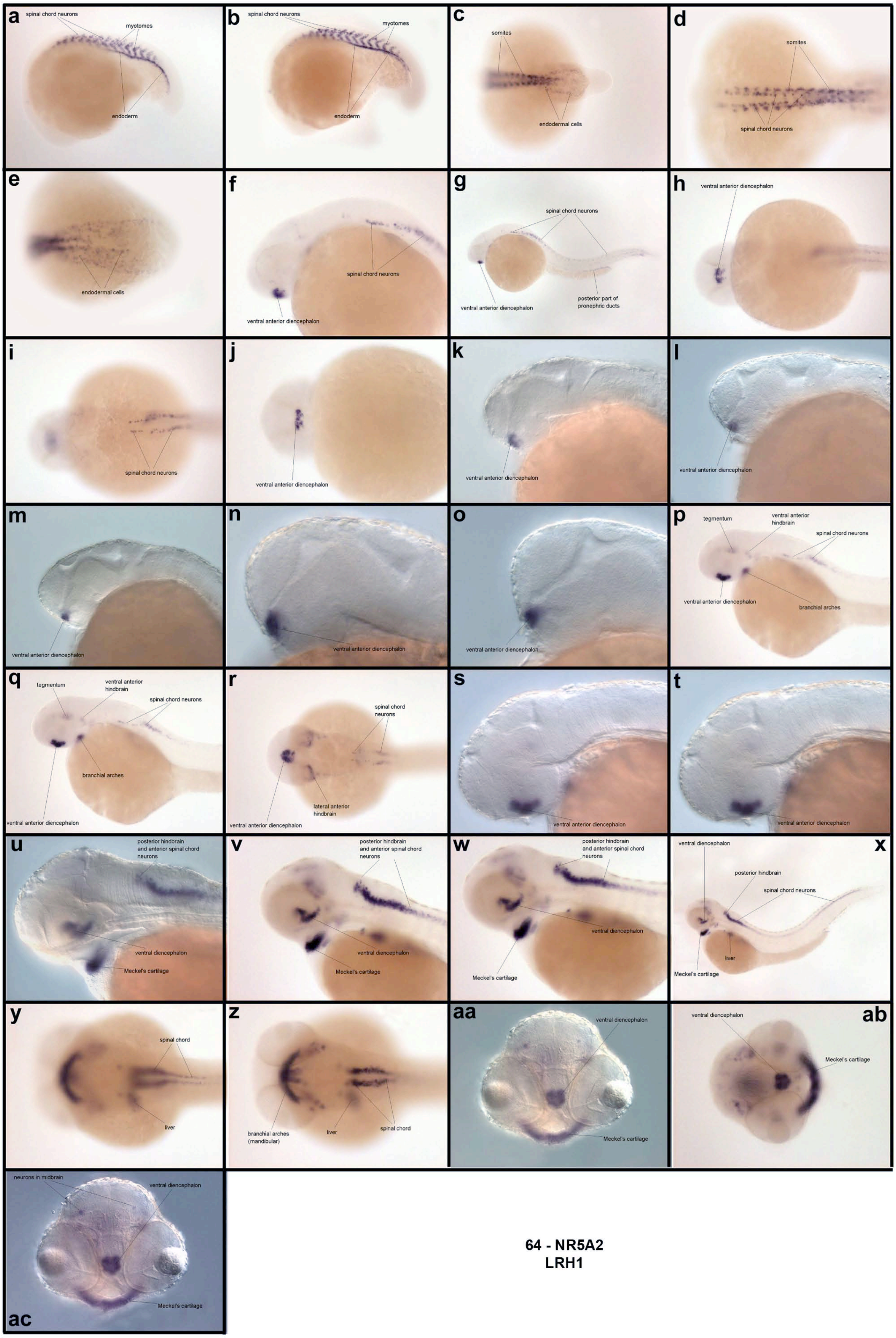

64 - NR5A2
LRH1

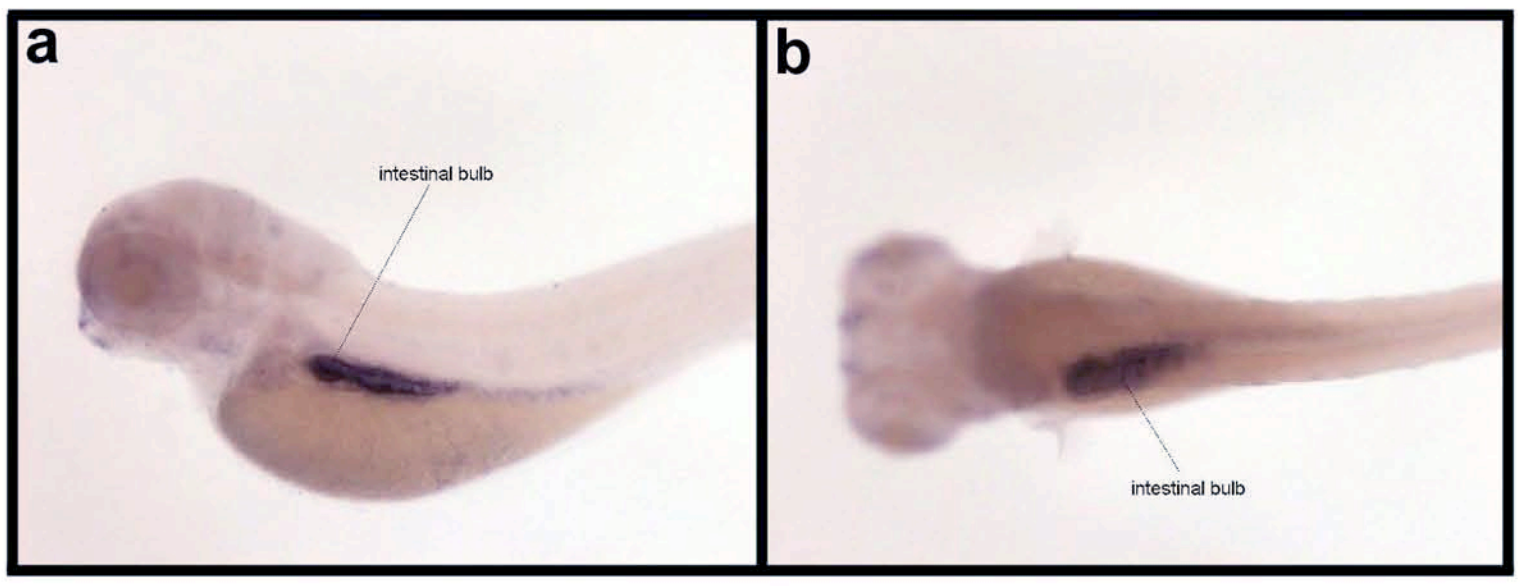

65 - NR5A5
FF1C

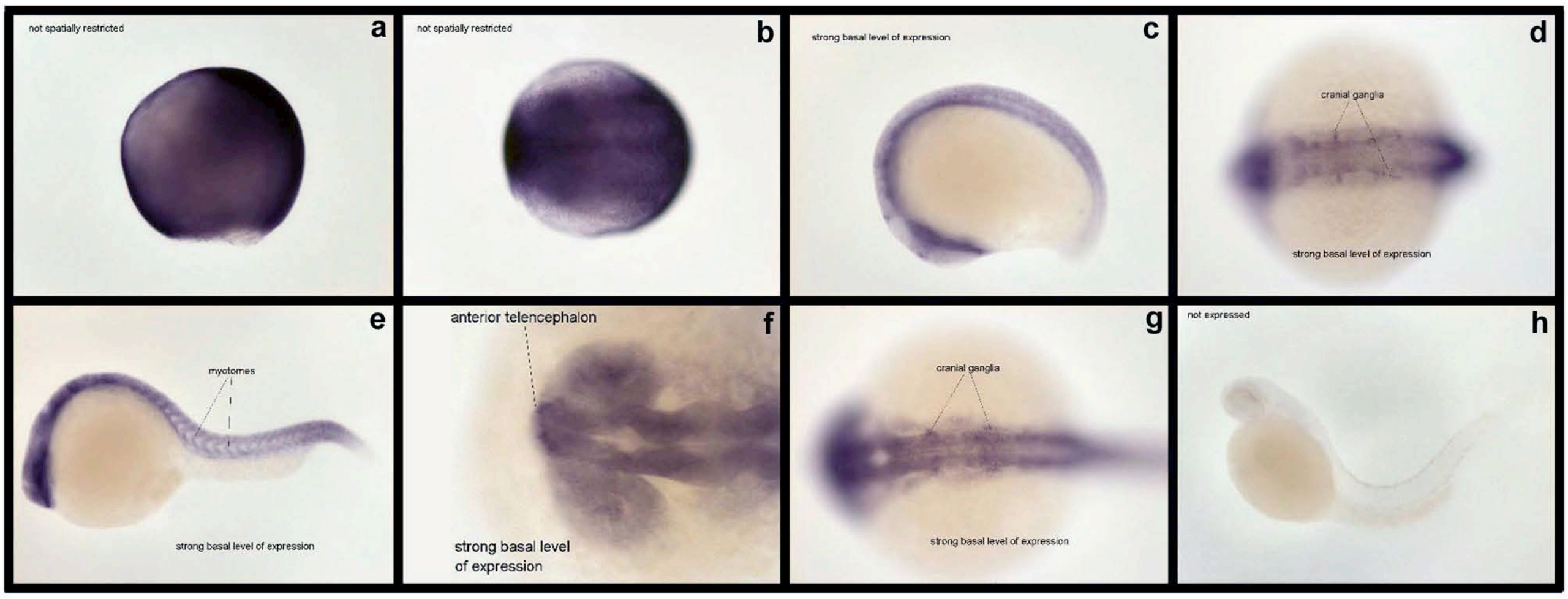

66 - NR6A1-A
GCNF-A

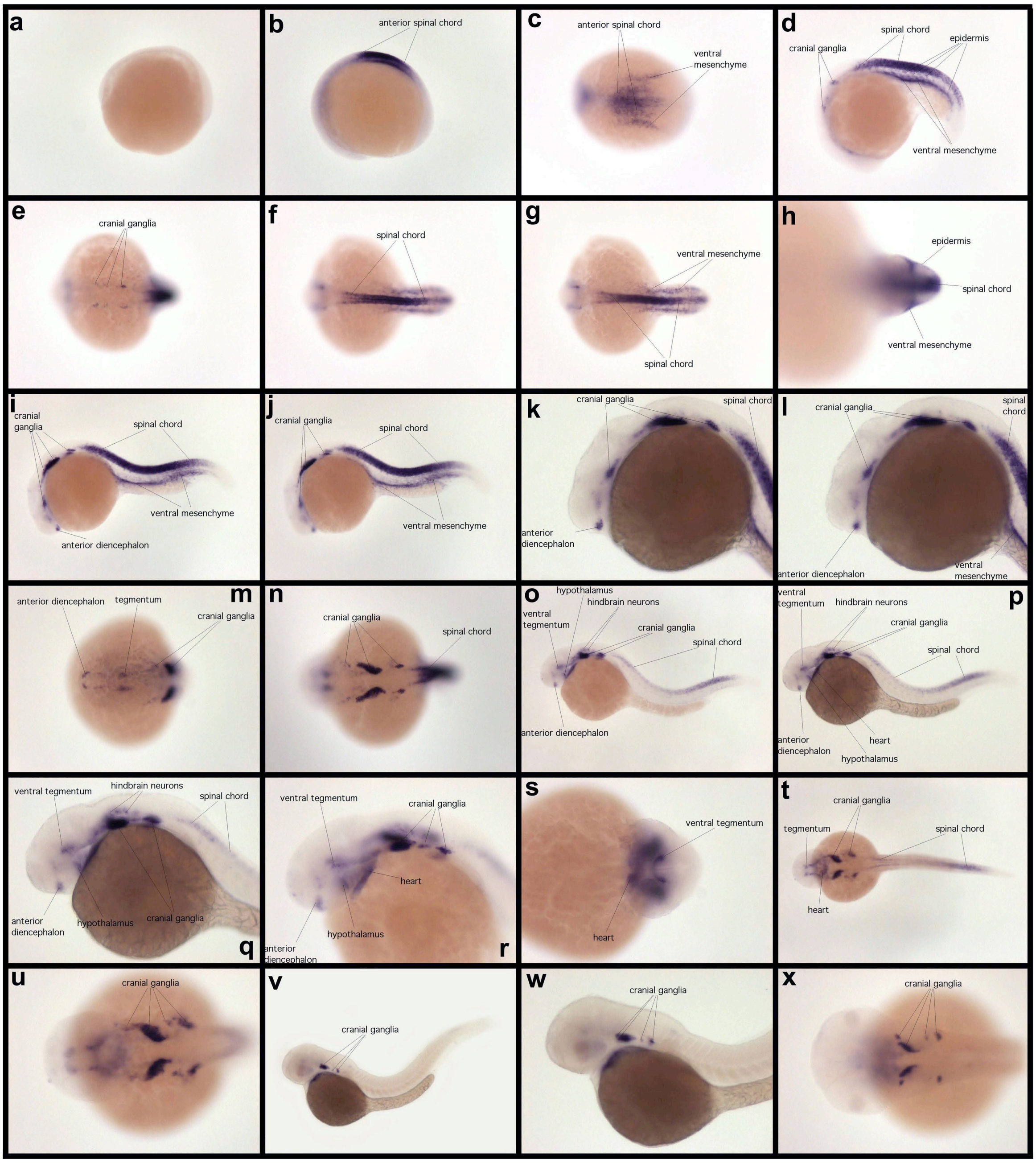

67 - NR6A1-B
GCNF-B

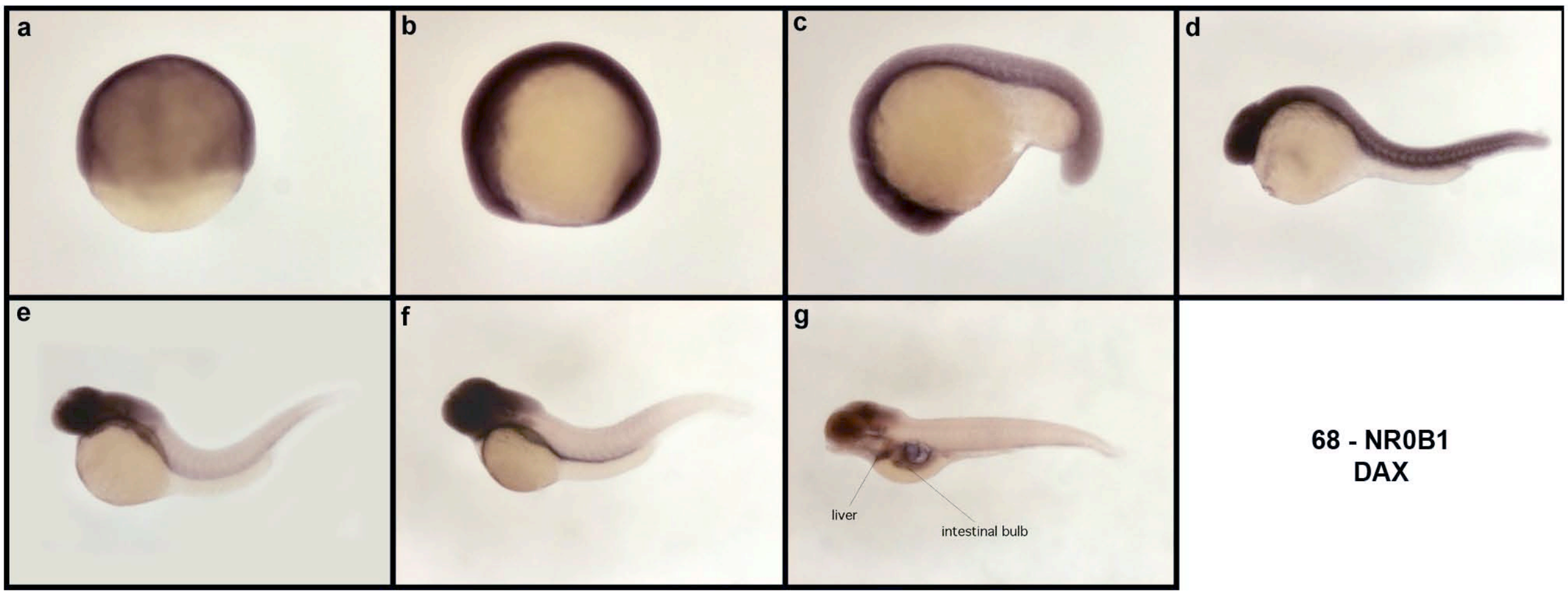

68 - NR0B1
DAX

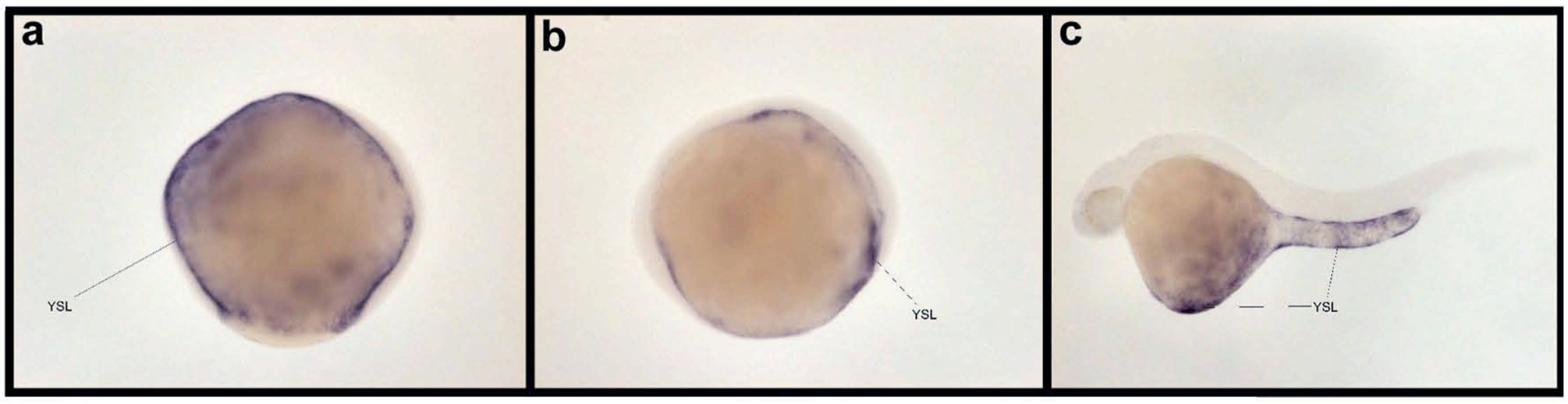

69 - NR0B2-A
SHP-A

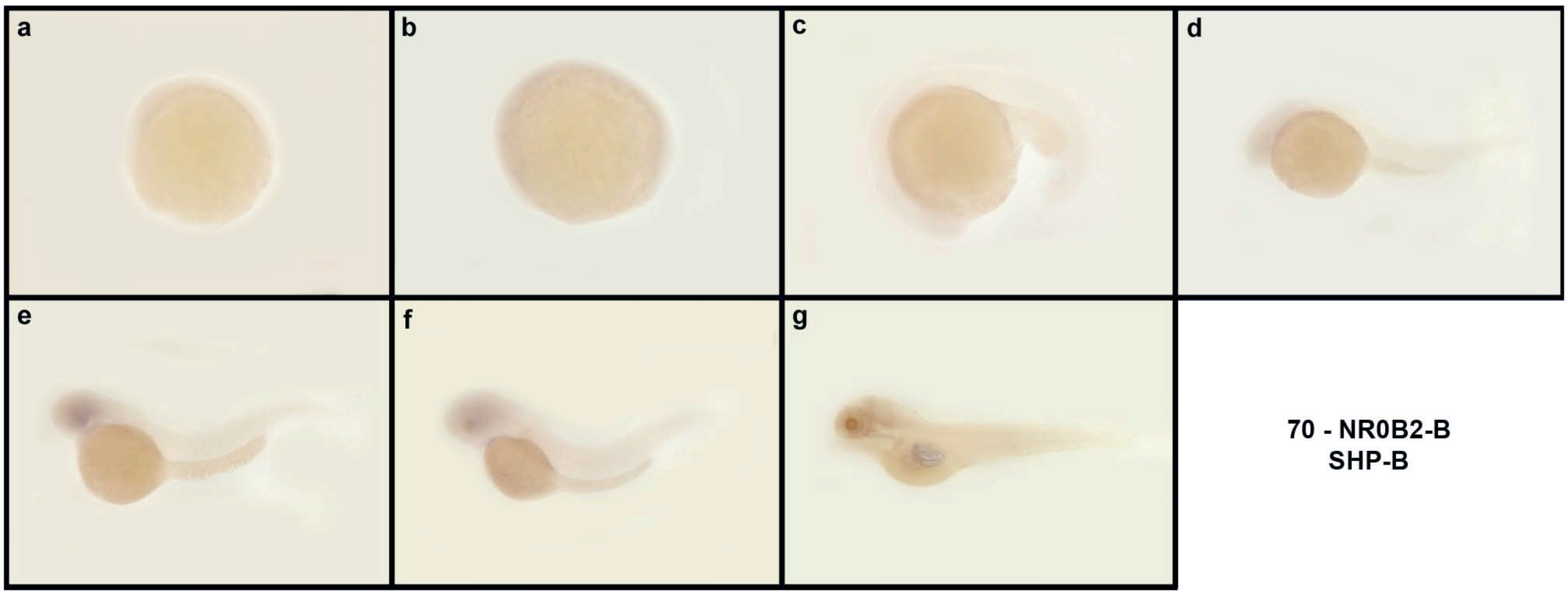

70 - NR0B2-B
SHP-B

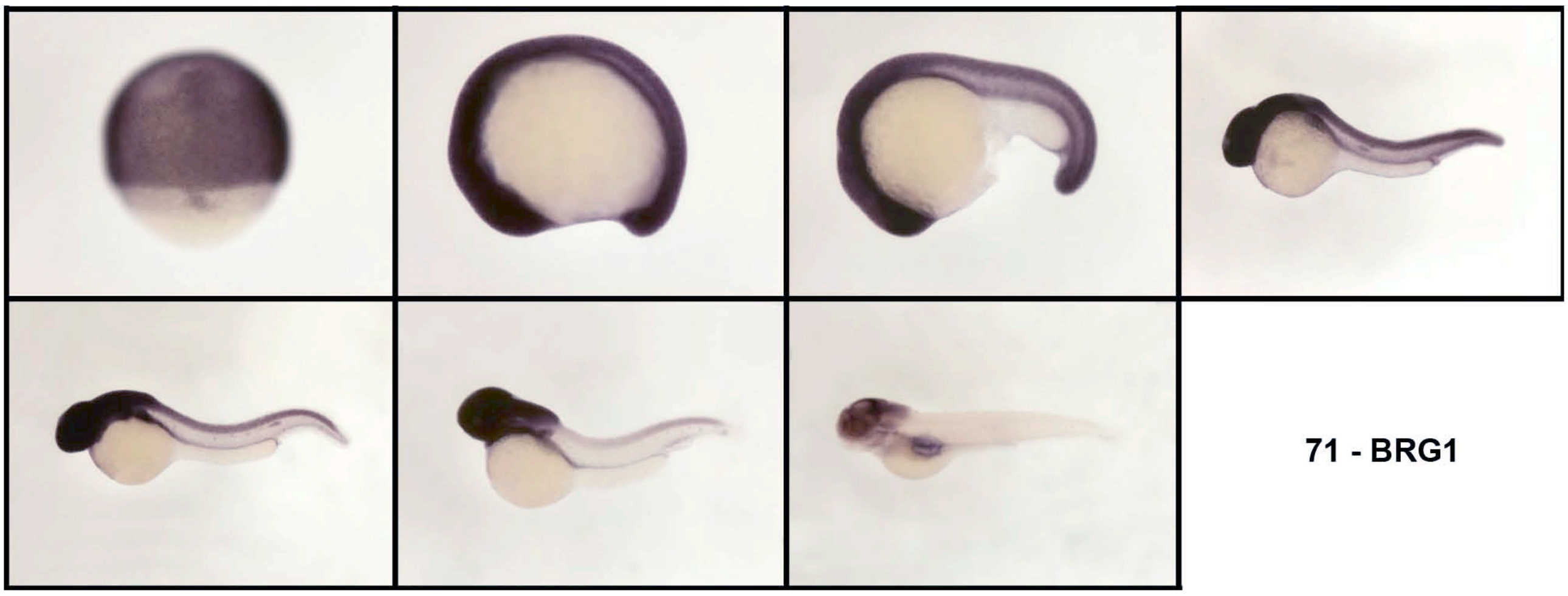

71 - BRG1

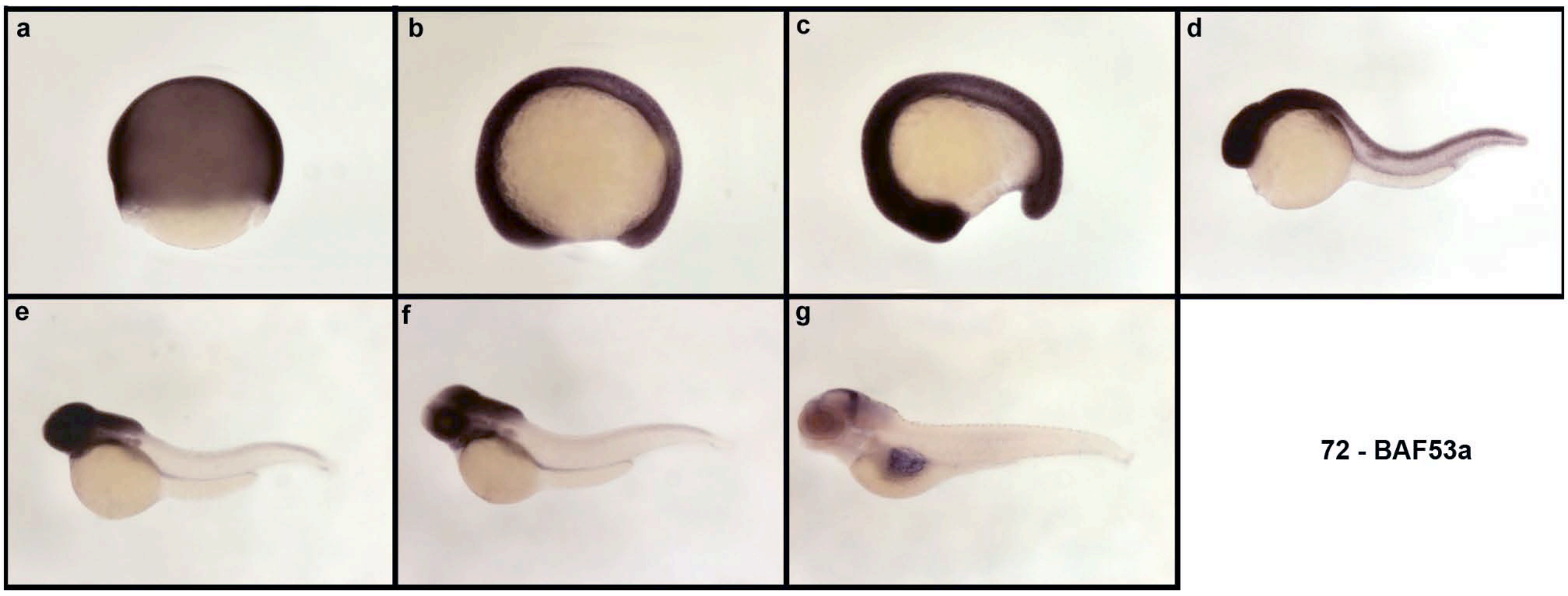

72 - BAF53a

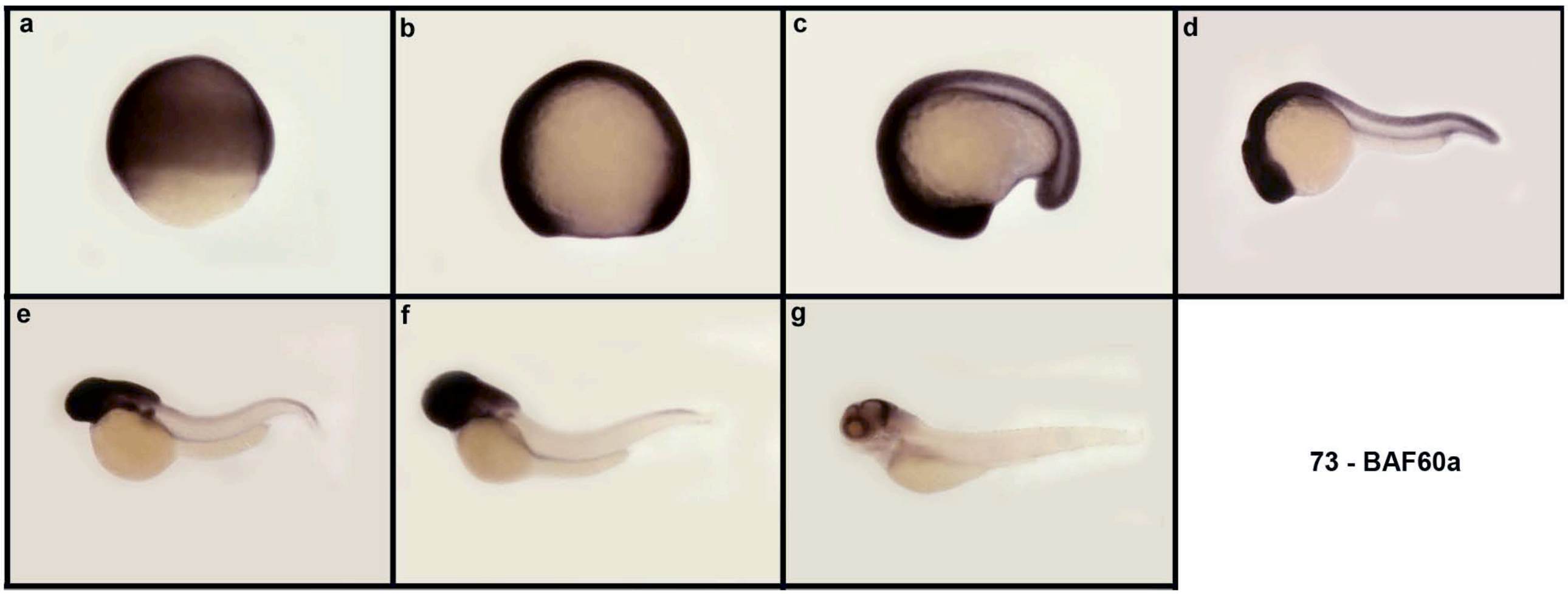

73 - BAF60a

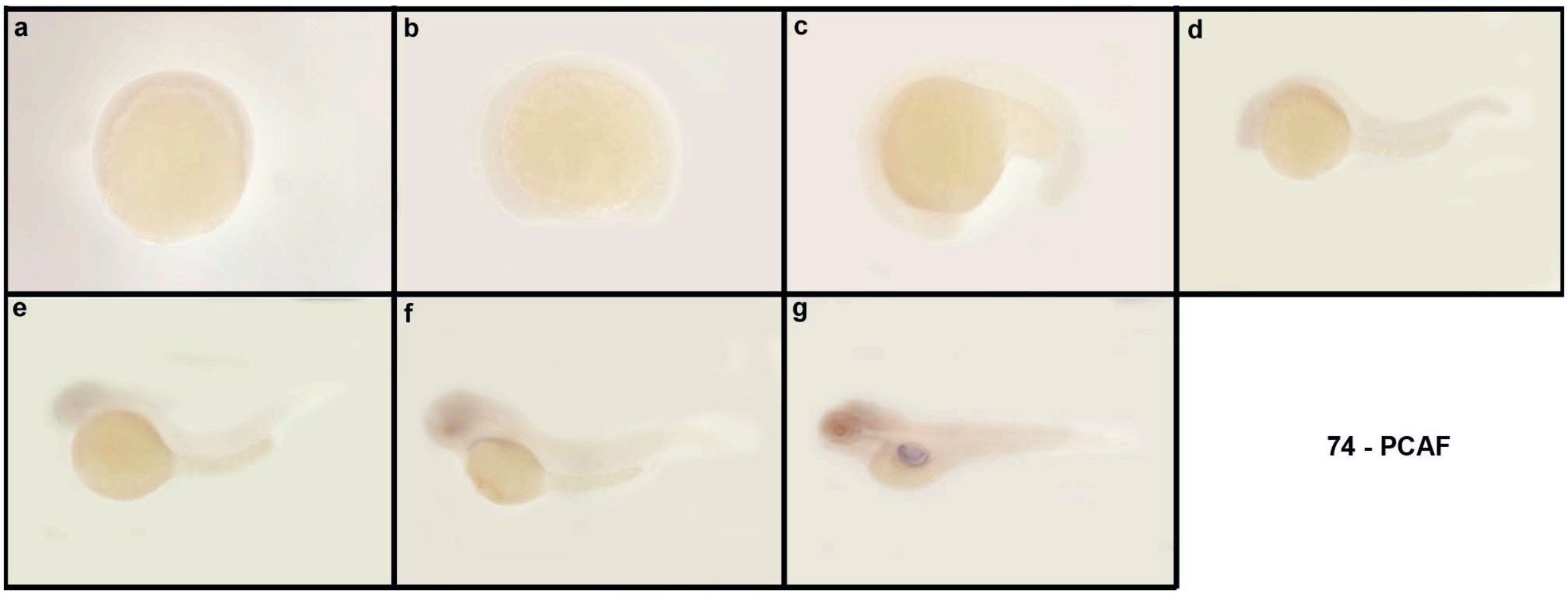

74 - PCAF

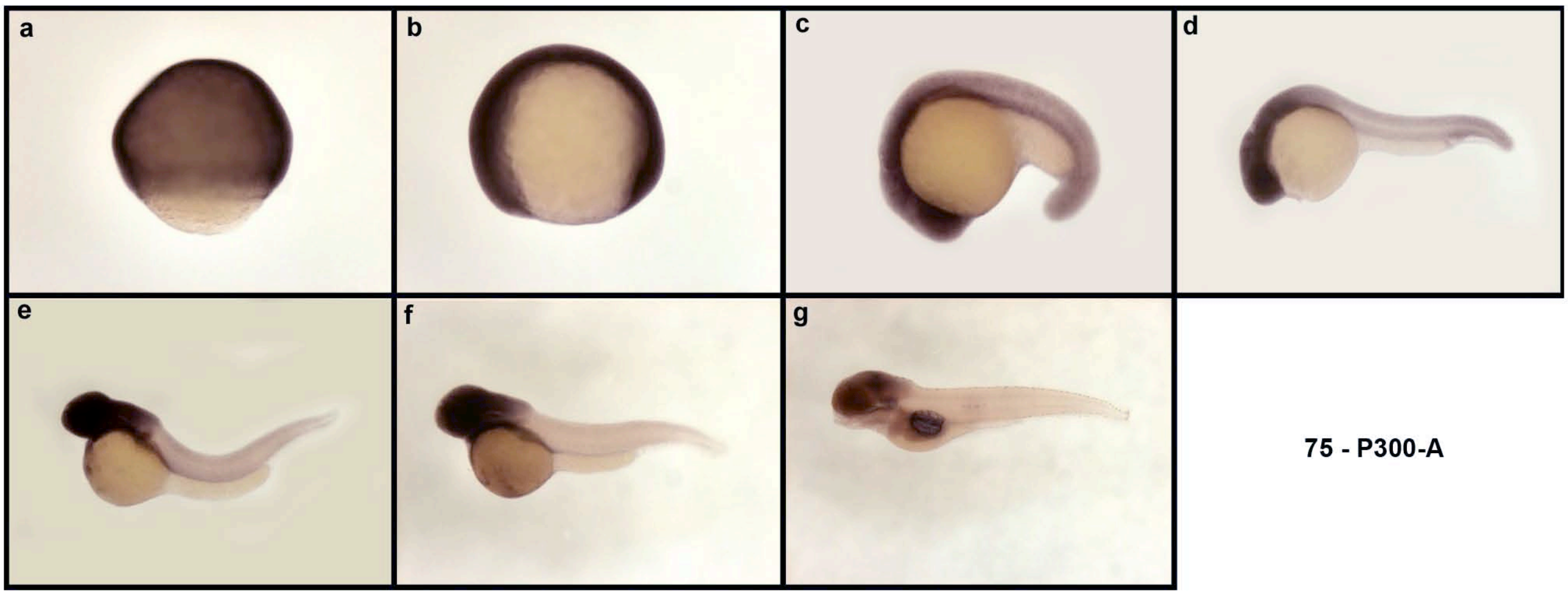

75 - P300-A

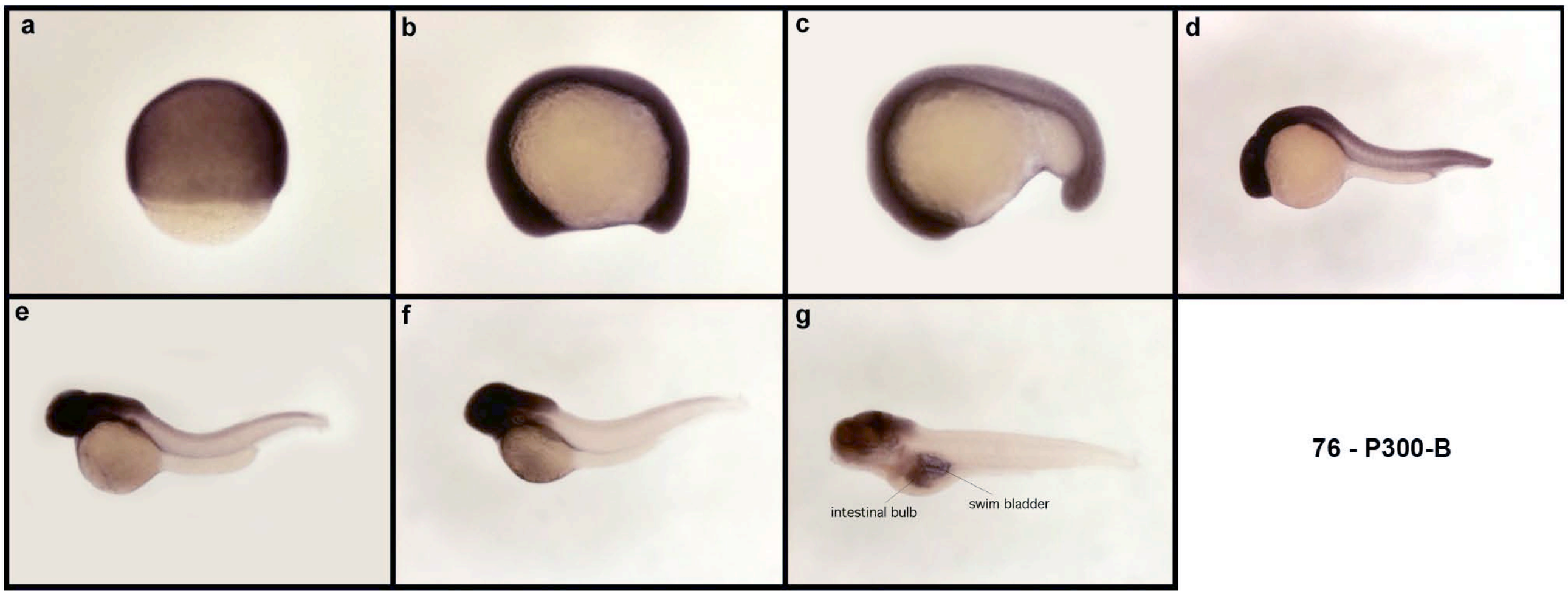

76 - P300-B

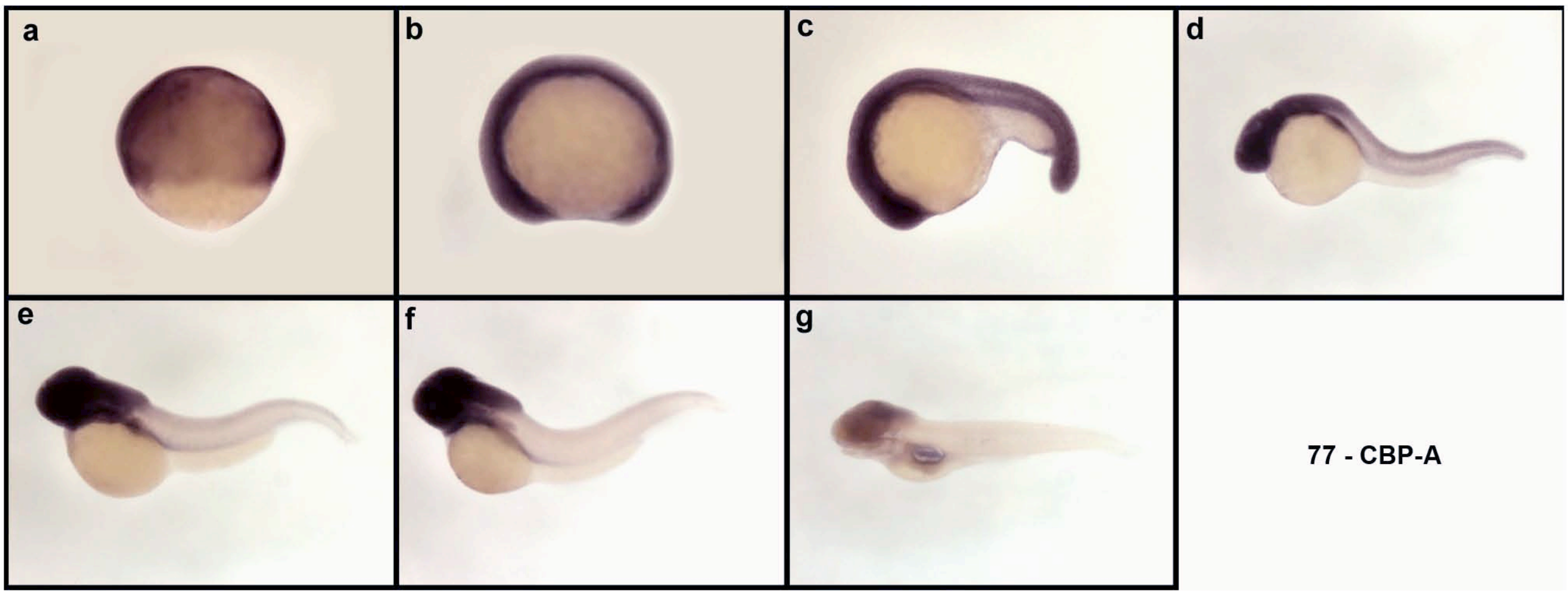
a
b
c
d
e
f
g
77 - CBP-A

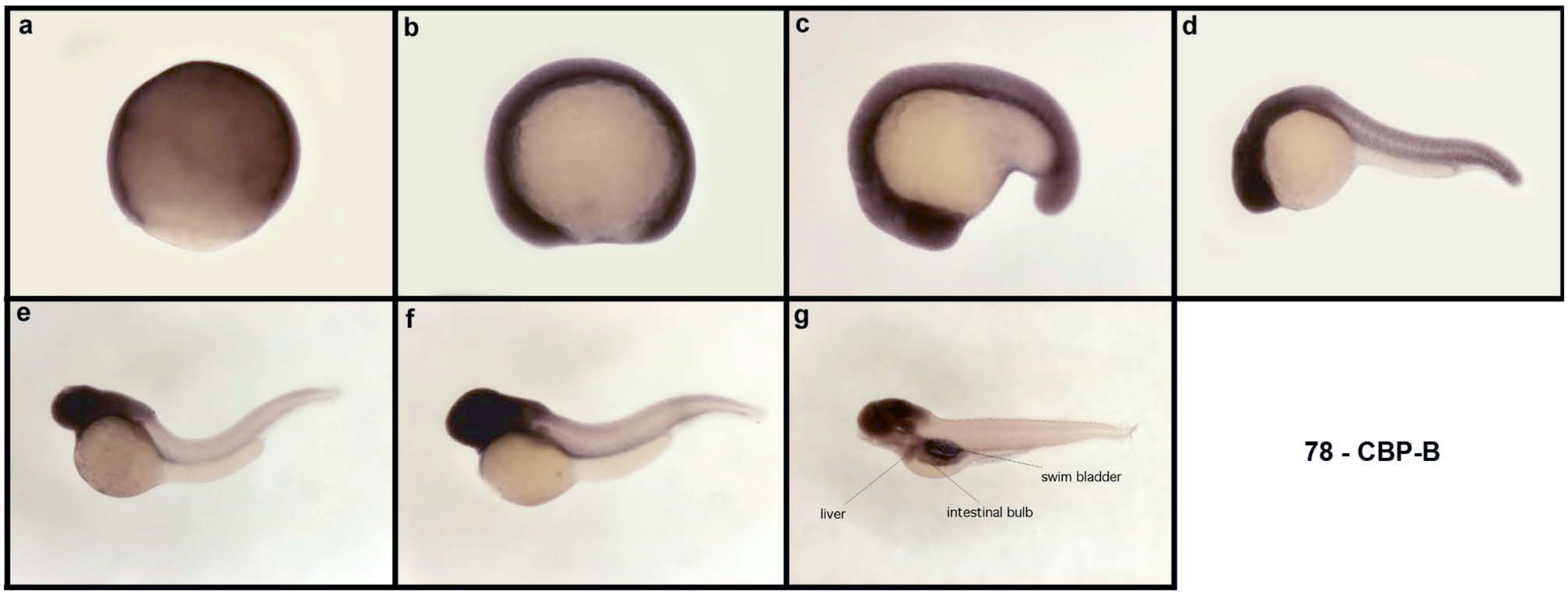

78 - CBP-B

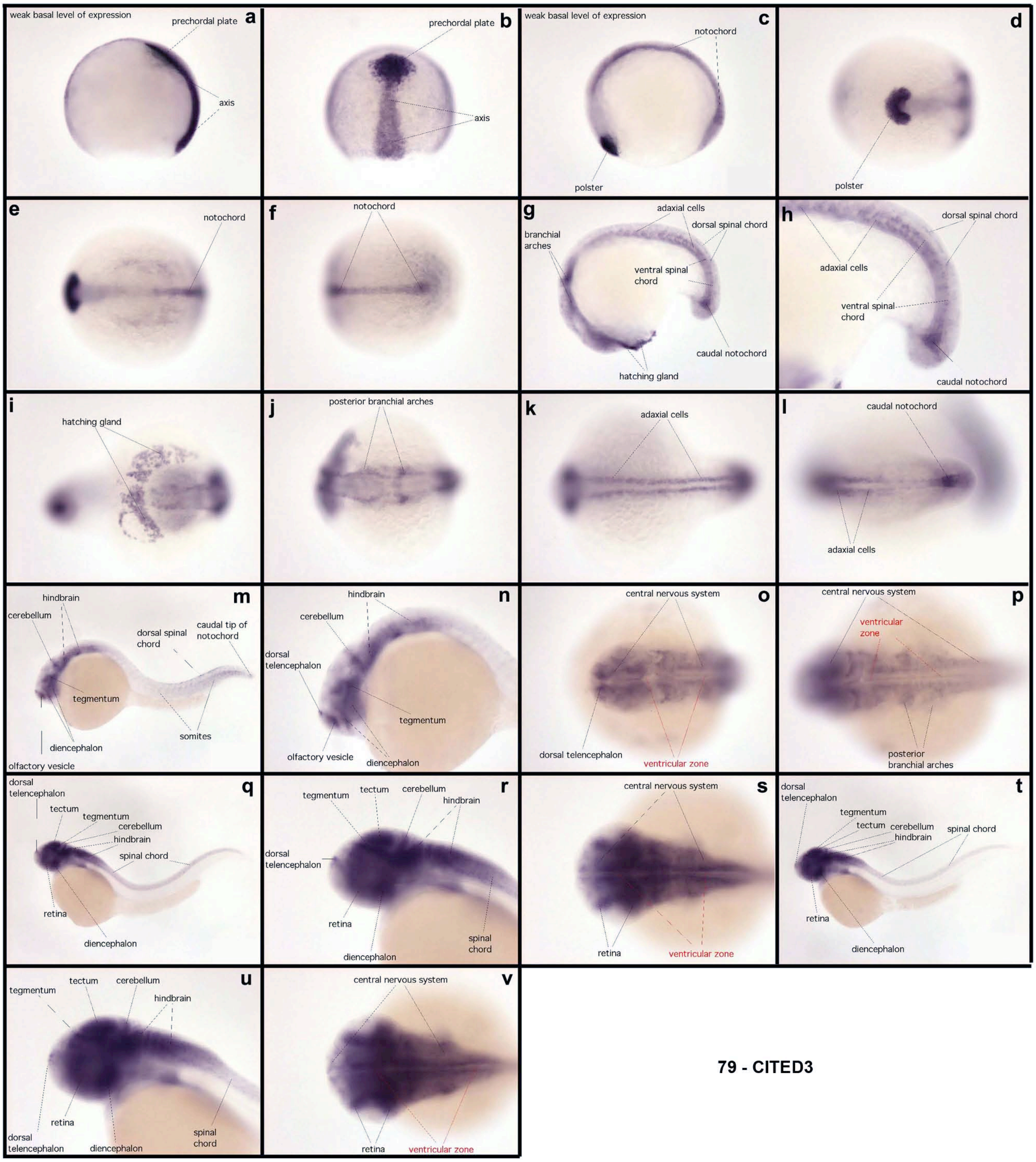
a
weak basal level of expression
prechordal plate
axis
b
prechordal plate
axis
c
weak basal level of expression
notochord
polster
d
polster
e
notochord
f
notochord
g
adaxial cells
dorsal spinal chord
branchial arches
ventral spinal chord
caudal notochord
hatching gland
h
dorsal spinal chord
adaxial cells
ventral spinal chord
caudal notochord
i
hatching gland
j
posterior branchial arches
k
adaxial cells
l
caudal notochord
adaxial cells
m
hindbrain
cerebellum
dorsal spinal chord
caudal tip of notochord
tegmentum
somites
diencephalon
olfactory vesicle
n
hindbrain
cerebellum
dorsal telencephalon
tegmentum
olfactory vesicle
diencephalon
o
central nervous system
dorsal telencephalon
ventricular zone
p
central nervous system
ventricular zone
posterior branchial arches
q
dorsal telencephalon
tectum
tegmentum
cerebellum
hindbrain
spinal chord
retina
diencephalon
r
tectum
tegmentum
cerebellum
hindbrain
dorsal telencephalon
retina
diencephalon
spinal chord
s
central nervous system
retina
ventricular zone
t
dorsal telencephalon
tegmentum
tectum
cerebellum
hindbrain
spinal chord
retina
diencephalon
u
tegmentum
tectum
cerebellum
hindbrain
retina
dorsal telencephalon
diencephalon
spinal chord
v
central nervous system
retina
ventricular zone

79 - CITED3

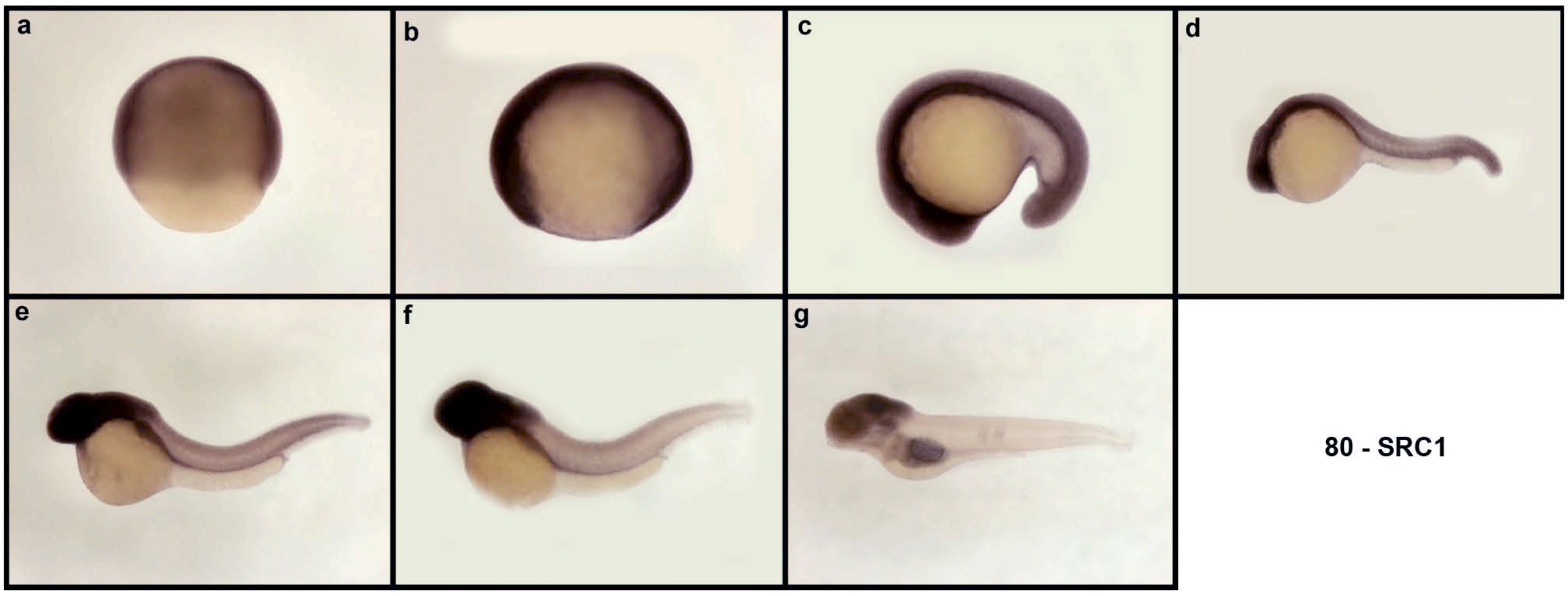

80 - SRC1

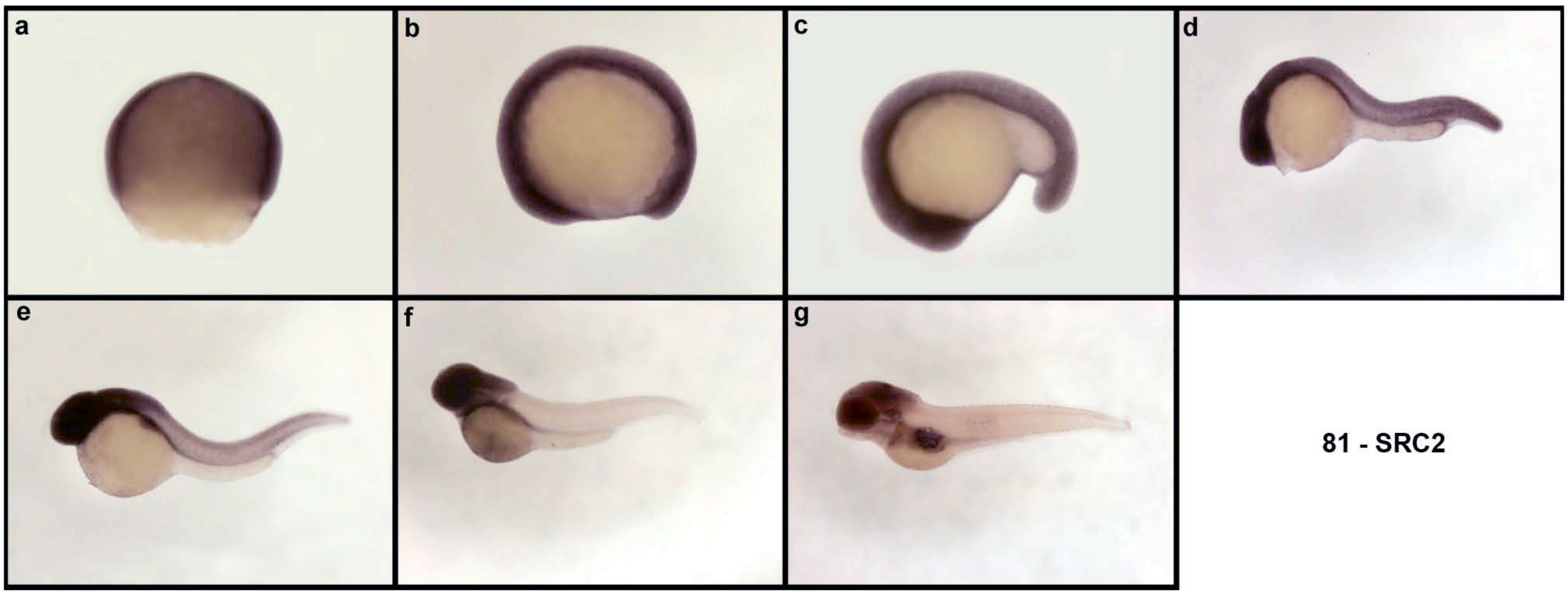

81 - SRC2

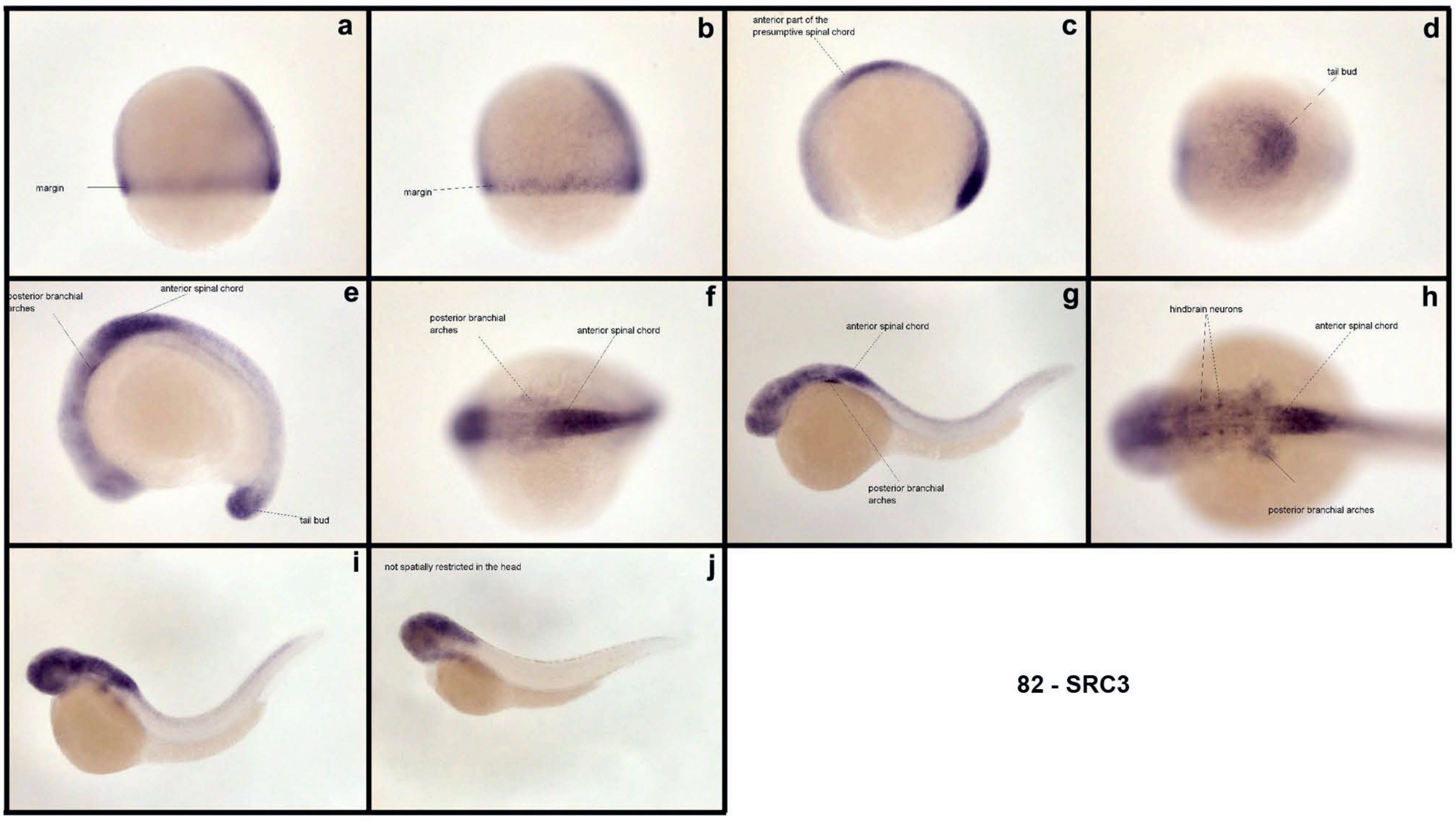
a
margin
b
margin
c
anterior part of the
presumptive spinal chord
d
tail bud
e
posterior branchial
arches
anterior spinal chord
tail bud
f
posterior branchial
arches
anterior spinal chord
g
anterior spinal chord
posterior branchial
arches
h
hindbrain neurons
anterior spinal chord
posterior branchial arches
i
j
not spatially restricted in the head

82 - SRC3

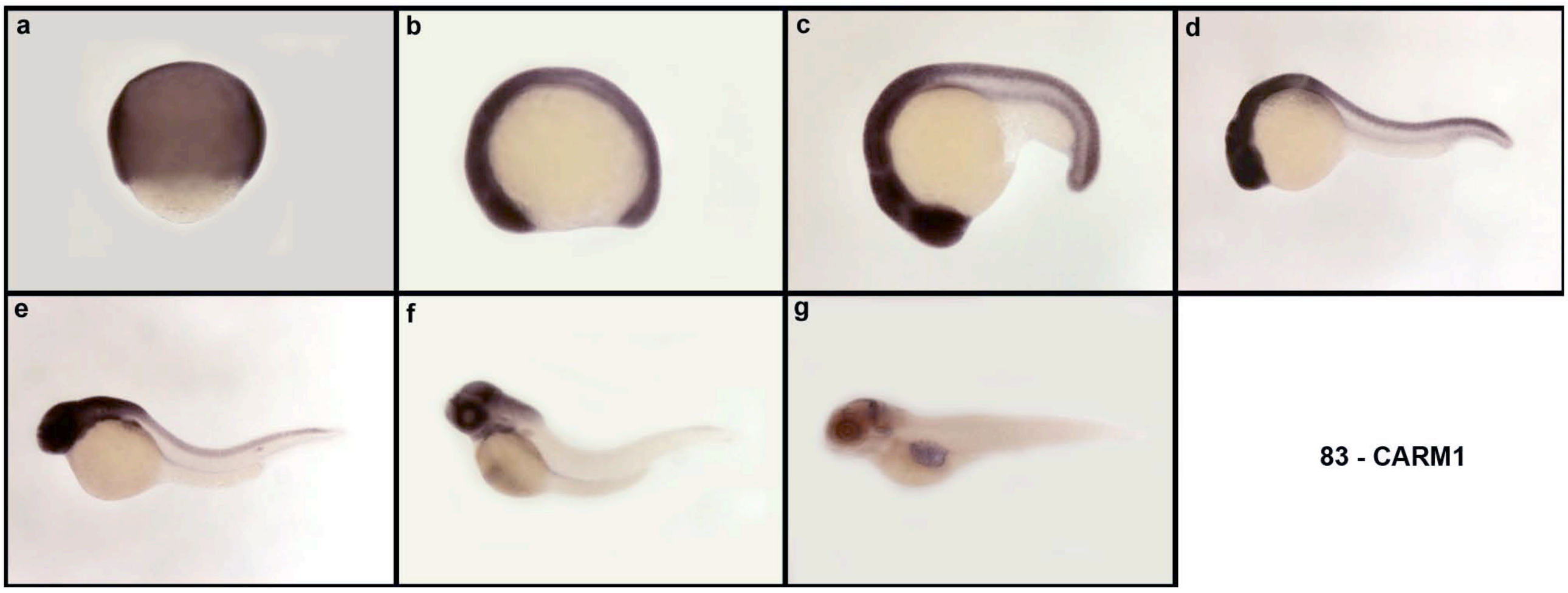

83 - CARM1

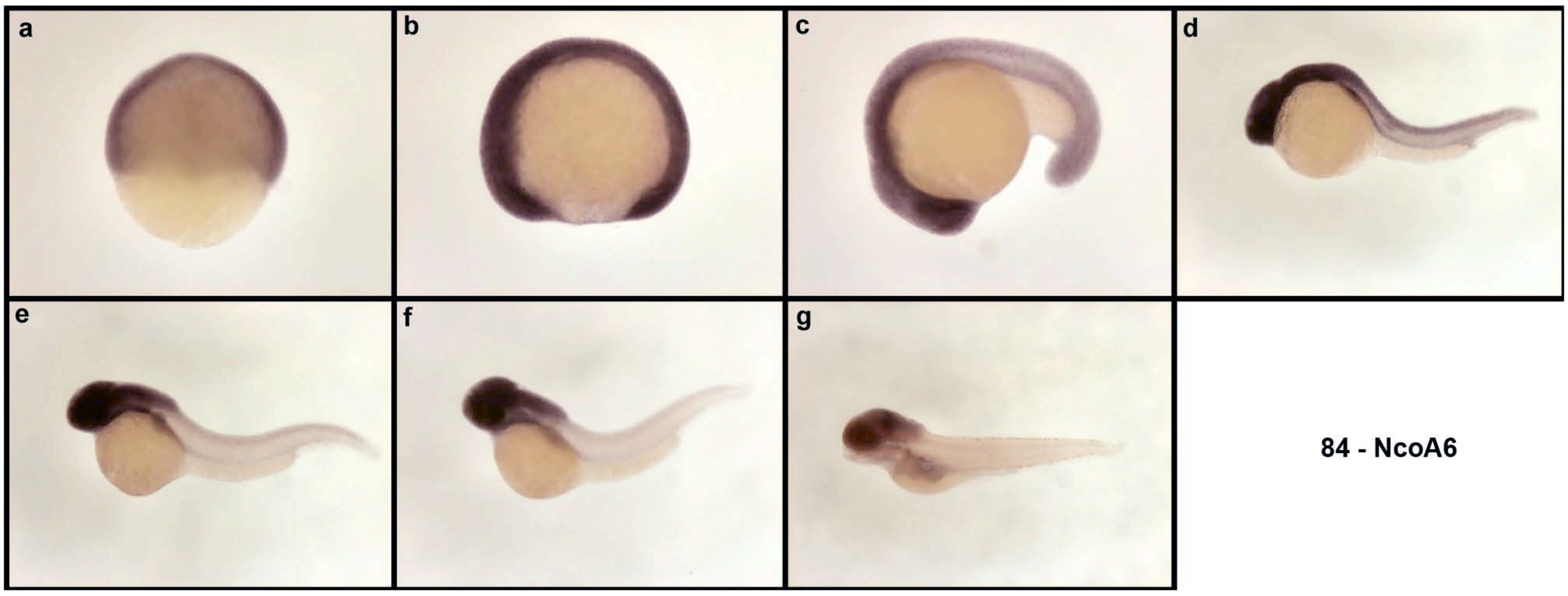

84 - NcoA6

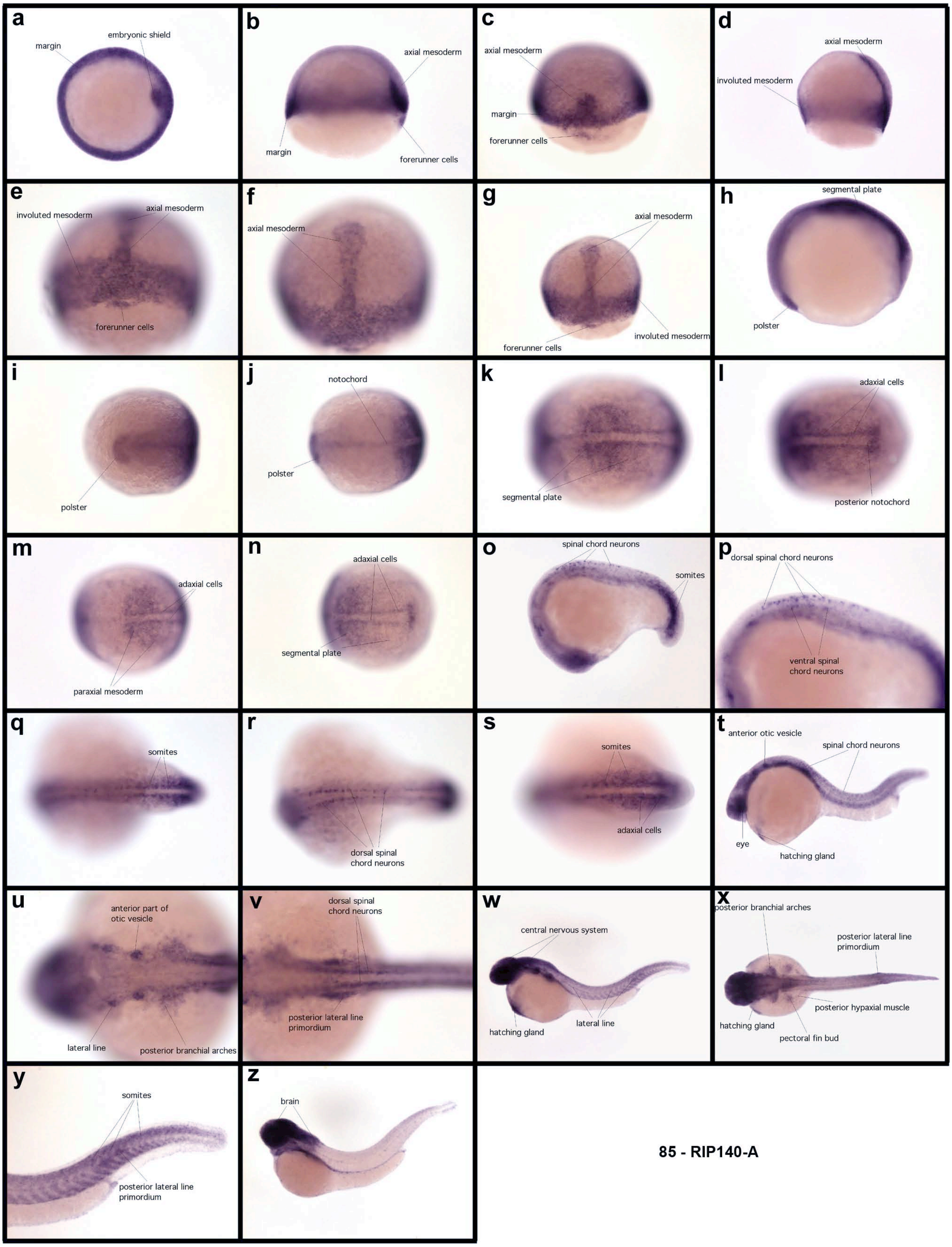
a
margin
embryonic shield
b
axial mesoderm
margin
forerunner cells
c
axial mesoderm
margin
forerunner cells
d
axial mesoderm
involuted mesoderm
e
involuted mesoderm
axial mesoderm
forerunner cells
f
axial mesoderm
g
axial mesoderm
forerunner cells
involuted mesoderm
h
segmental plate
polster
i
polster
j
notochord
polster
k
segmental plate
l
adaxial cells
posterior notochord
m
adaxial cells
paraxial mesoderm
n
adaxial cells
segmental plate
o
spinal chord neurons
somites
p
dorsal spinal chord neurons
ventral spinal
chord neurons
q
somites
r
dorsal spinal
chord neurons
s
somites
adaxial cells
t
anterior otic vesicle
spinal chord neurons
eye
hatching gland
u
anterior part of
otic vesicle
lateral line
posterior branchial arches
v
dorsal spinal
chord neurons
posterior lateral line
primordium
w
central nervous system
hatching gland
lateral line
x
posterior branchial arches
posterior lateral line
primordium
posterior hypaxial muscle
hatching gland
pectoral fin bud
y
somites
posterior lateral line
primordium
z
brain

85 - RIP140-A

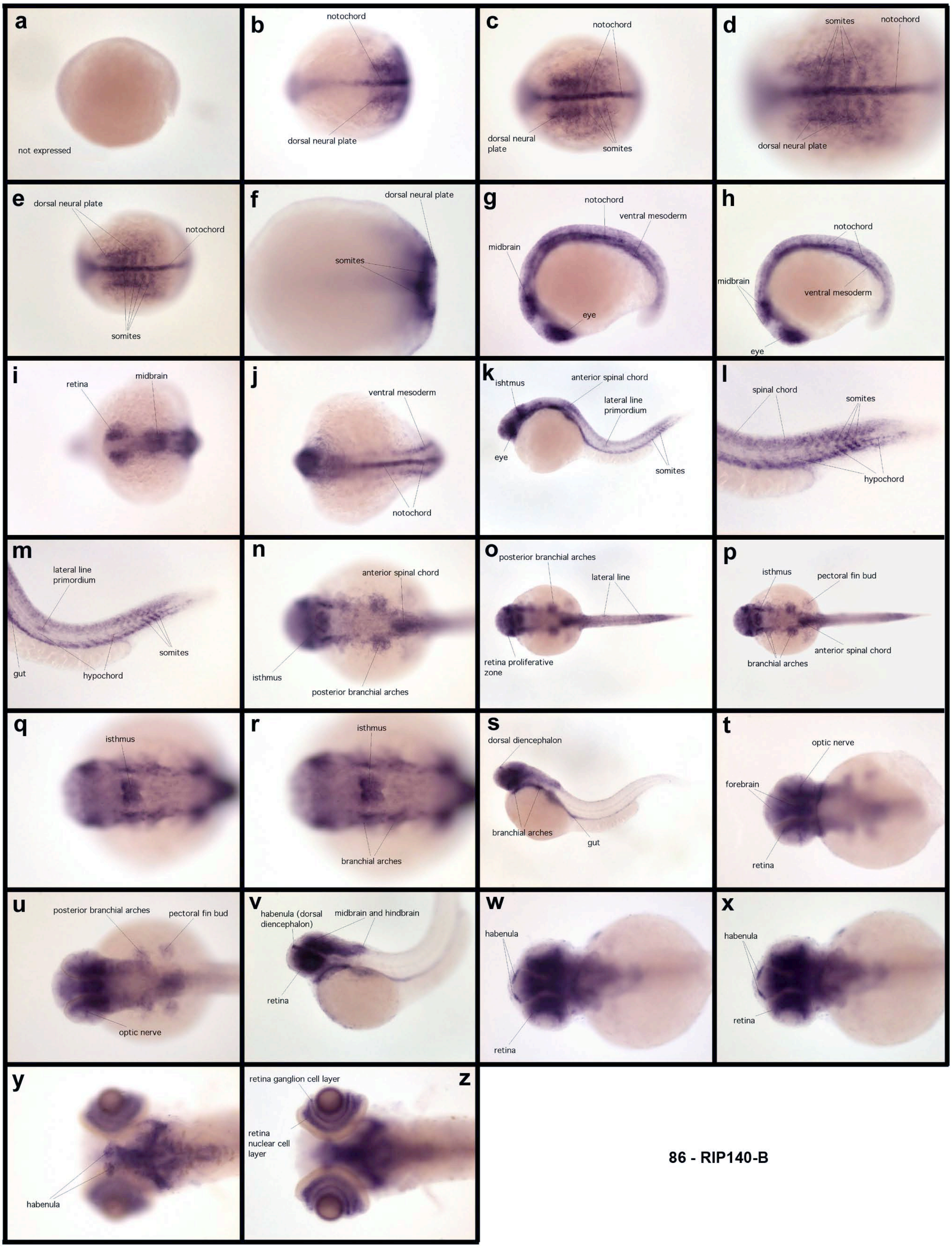
a
not expressed
b
notochord
dorsal neural plate
c
notochord
dorsal neural plate
somites
d
somites
notochord
dorsal neural plate
e
dorsal neural plate
notochord
somites
f
dorsal neural plate
somites
g
notochord
ventral mesoderm
midbrain
eye
h
notochord
midbrain
ventral mesoderm
eye
i
retina
midbrain
j
ventral mesoderm
notochord
k
ishtmus
anterior spinal chord
lateral line primordium
eye
somites
l
spinal chord
somites
hypochord
m
lateral line primordium
somites
gut
hypochord
n
anterior spinal chord
isthmus
posterior branchial arches
o
posterior branchial arches
lateral line
retina proliferative zone
p
isthmus
pectoral fin bud
anterior spinal chord
branchial arches
q
isthmus
r
isthmus
branchial arches
s
dorsal diencephalon
branchial arches
gut
t
optic nerve
forebrain
retina
u
posterior branchial arches
pectoral fin bud
optic nerve
v
habenula (dorsal diencephalon)
midbrain and hindbrain
retina
w
habenula
retina
x
habenula
retina
y
habenula
z
retina ganglion cell layer
retina nuclear cell layer

86 - RIP140-B

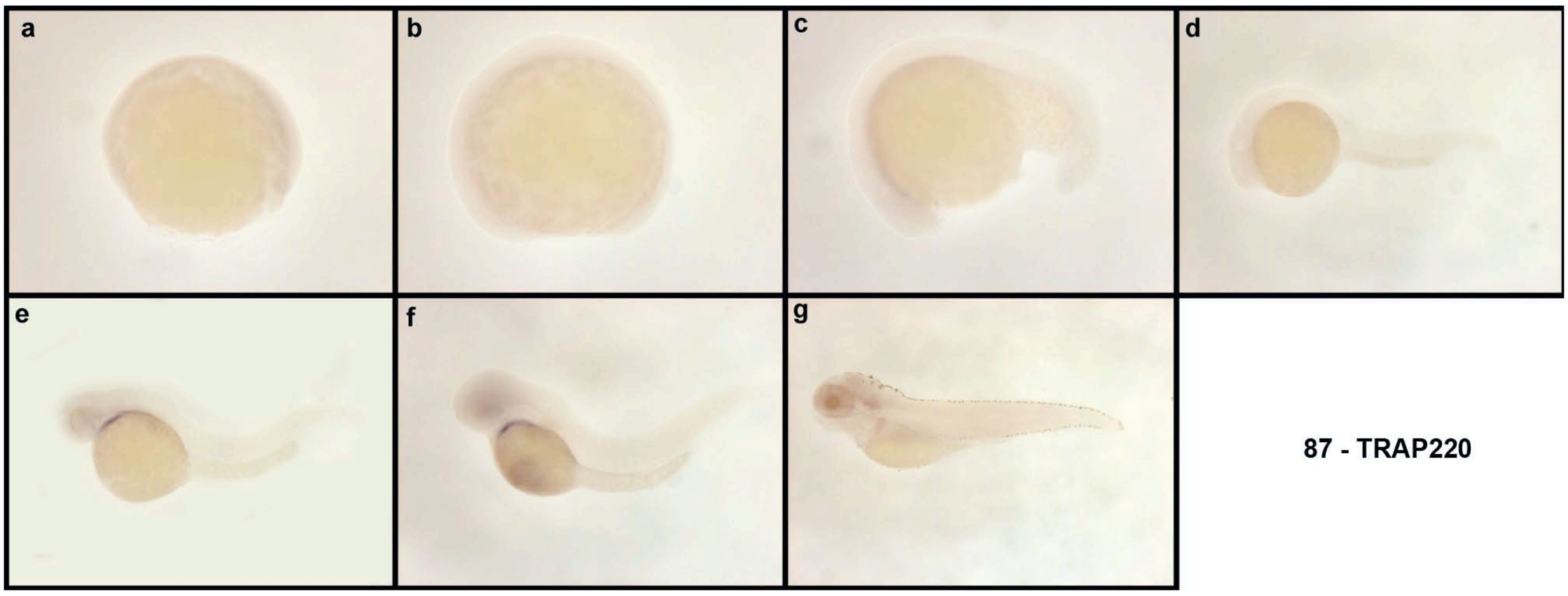

87 - TRAP220

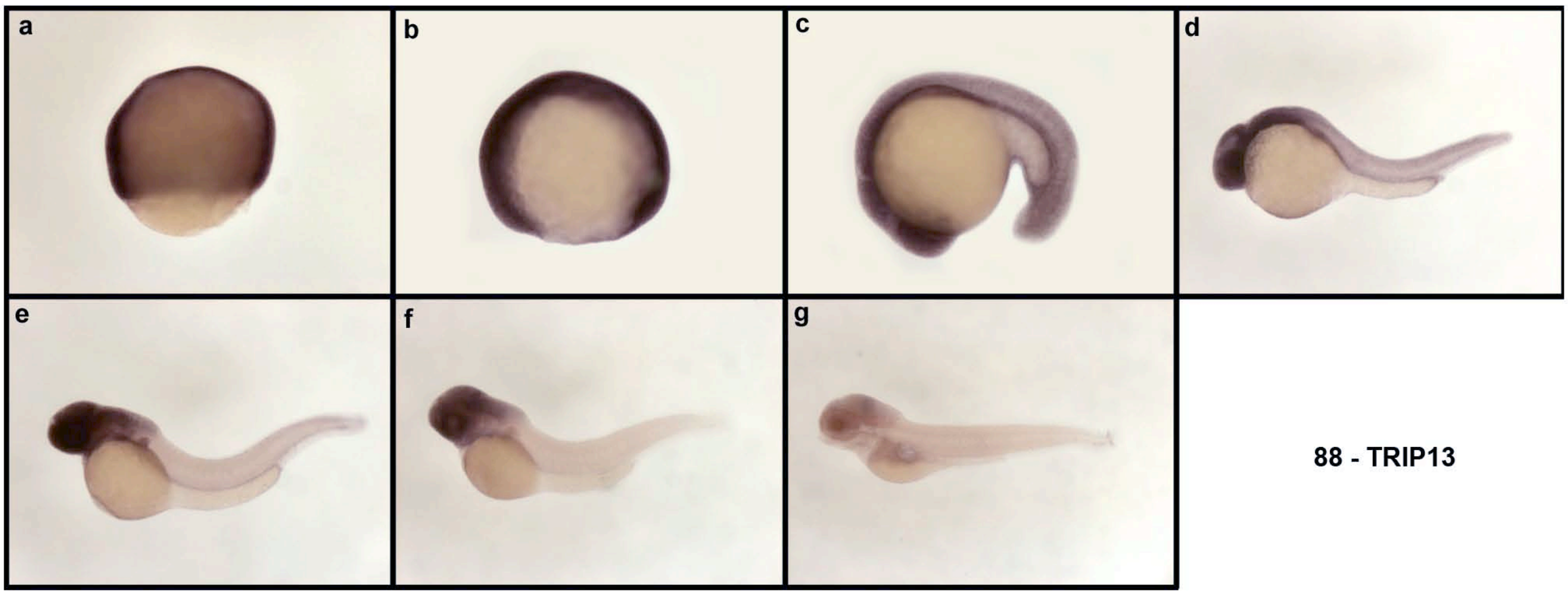

88 - TRIP13

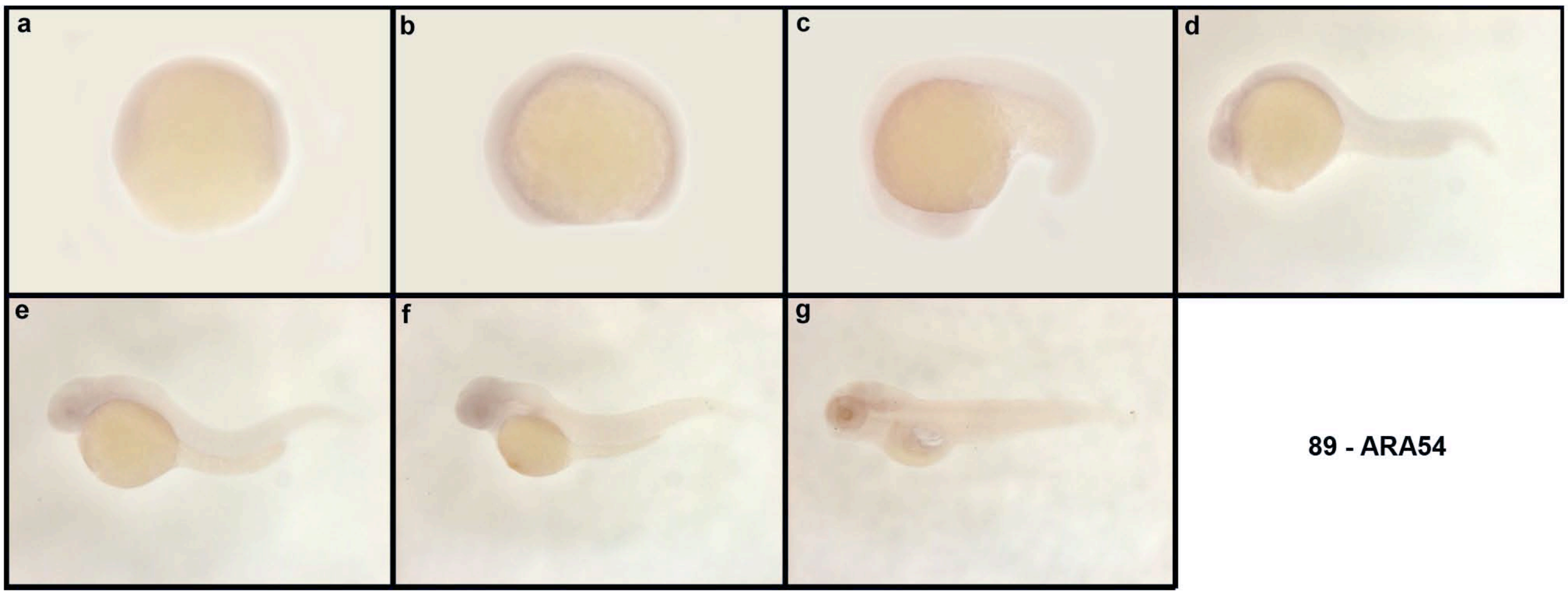

89 - ARA54

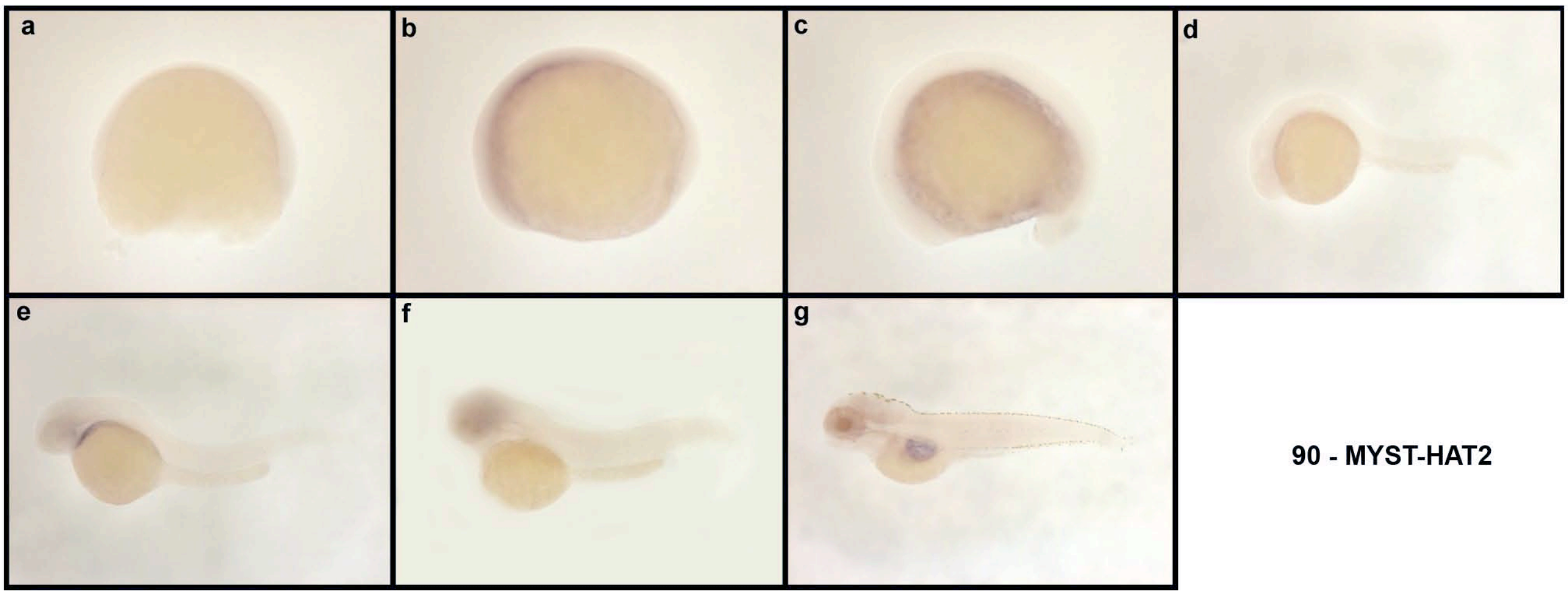

90 - MYST-HAT2

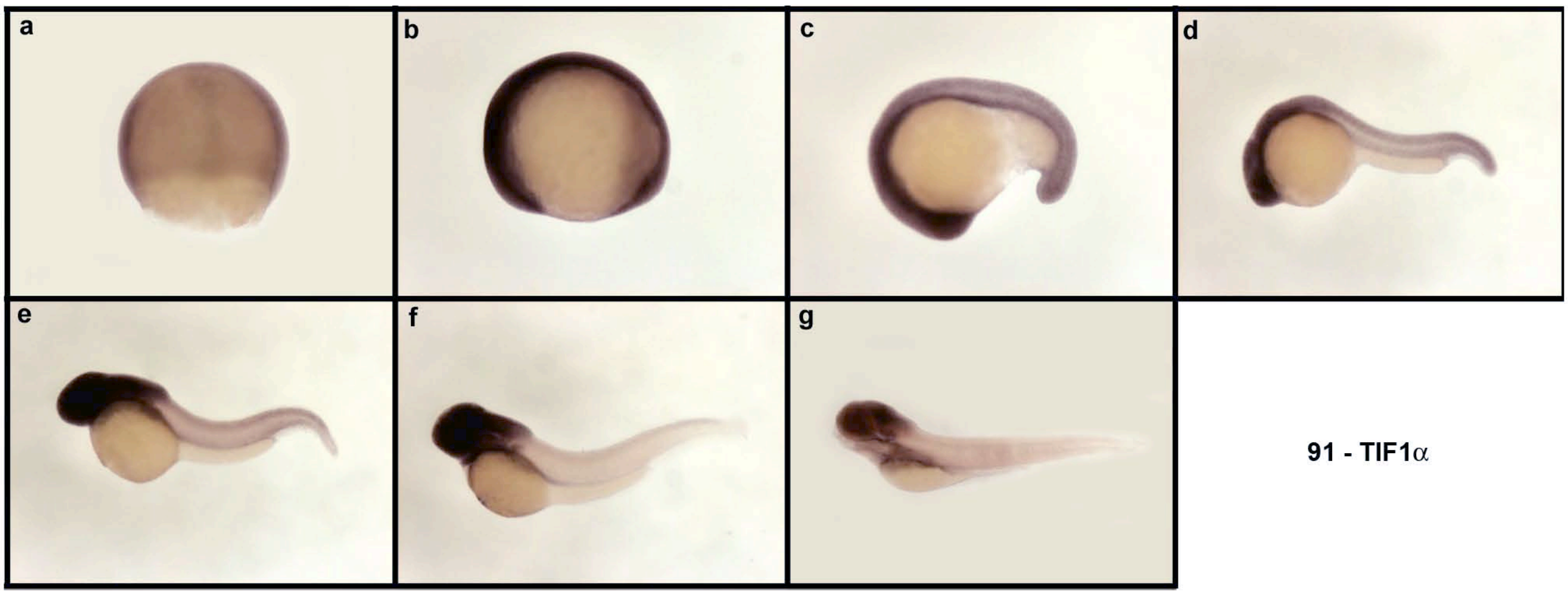

91 - TIF1α

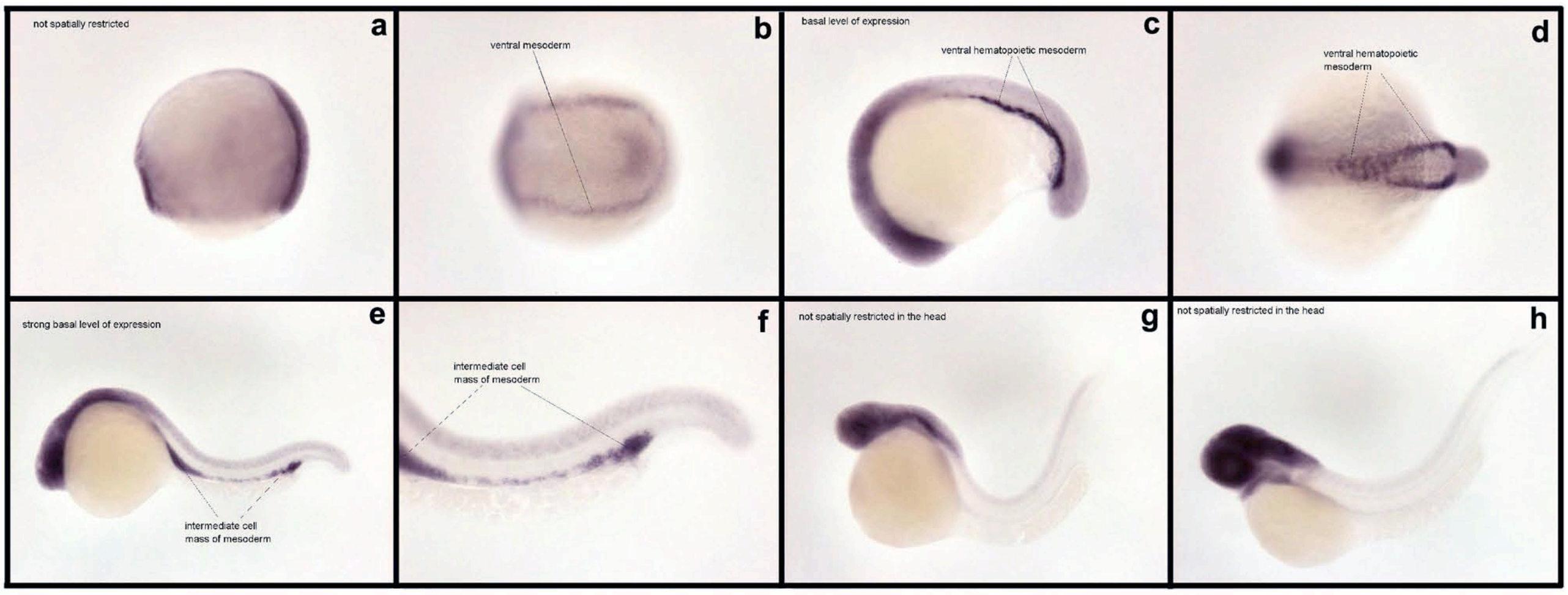
not spatially restricted
a
ventral mesoderm
b
basal level of expression
ventral hematopoietic mesoderm
c
ventral hematopoietic mesoderm
d
strong basal level of expression
intermediate cell mass of mesoderm
e
intermediate cell mass of mesoderm
f
not spatially restricted in the head
g
not spatially restricted in the head
h

92 - TIF1γ

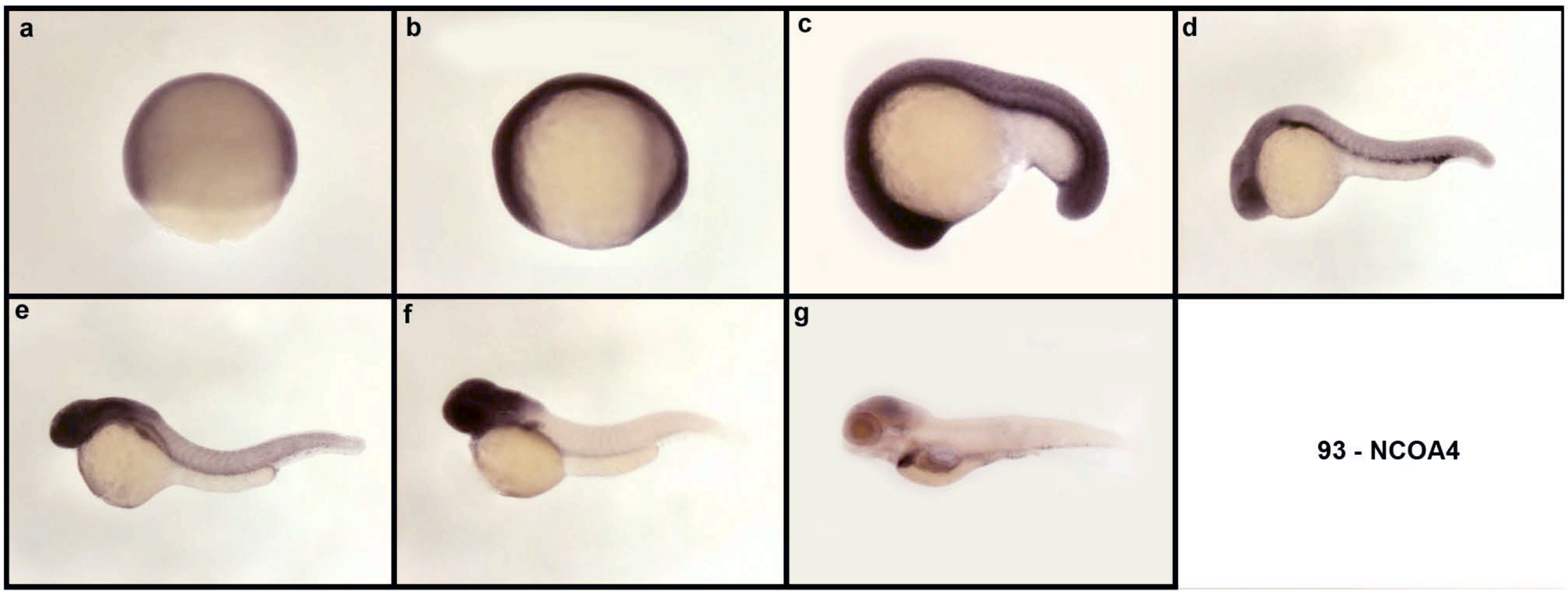

93 - NCOA4

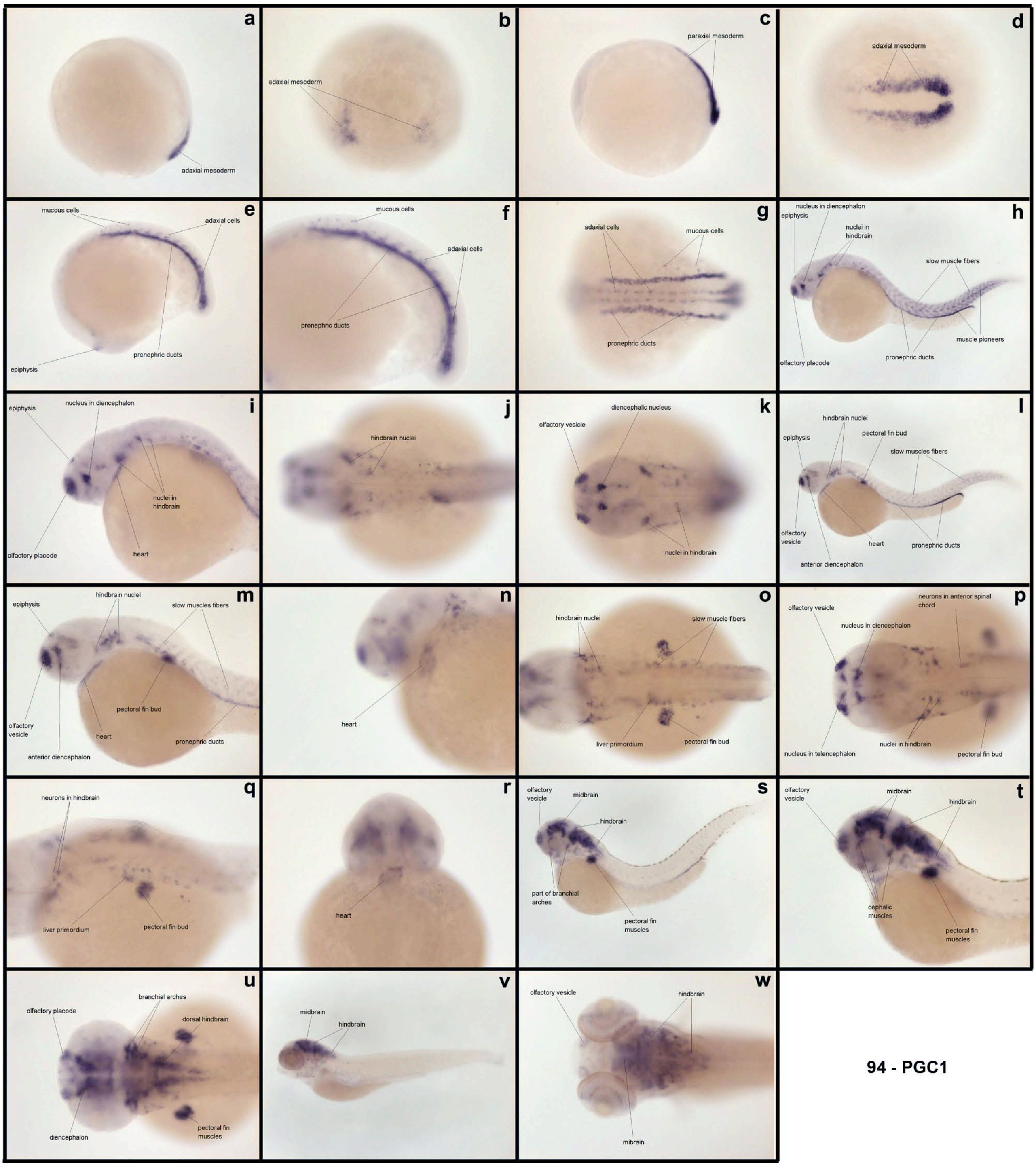
a
adaxial mesoderm
b
adaxial mesoderm
c
paraxial mesoderm
d
adaxial mesoderm
e
mucous cells
adaxial cells
epiphysis
pronephric ducts
f
mucous cells
adaxial cells
pronephric ducts
g
adaxial cells
mucous cells
pronephric ducts
h
nucleus in diencephalon
epiphysis
nuclei in hindbrain
slow muscle fibers
muscle pioneers
olfactory placode
pronephric ducts
i
epiphysis
nucleus in diencephalon
nuclei in hindbrain
olfactory placode
heart
j
hindbrain nuclei
k
diencephalic nucleus
olfactory vesicle
nuclei in hindbrain
l
hindbrain nuclei
epiphysis
pectoral fin bud
slow muscles fibers
olfactory vesicle
heart
pronephric ducts
anterior diencephalon
m
epiphysis
hindbrain nuclei
slow muscles fibers
pectoral fin bud
olfactory vesicle
heart
pronephric ducts
anterior diencephalon
n
heart
o
hindbrain nuclei
slow muscle fibers
liver primordium
pectoral fin bud
p
olfactory vesicle
neurons in anterior spinal chord
nucleus in diencephalon
nucleus in telencephalon
nuclei in hindbrain
pectoral fin bud
q
neurons in hindbrain
liver primordium
pectoral fin bud
r
heart
s
olfactory vesicle
midbrain
hindbrain
part of branchial arches
pectoral fin muscles
t
olfactory vesicle
midbrain
hindbrain
cephalic muscles
pectoral fin muscles
u
branchial arches
olfactory placode
dorsal hindbrain
diencephalon
pectoral fin muscles
v
midbrain
hindbrain
w
olfactory vesicle
hindbrain
mibrain

94 - PGC1

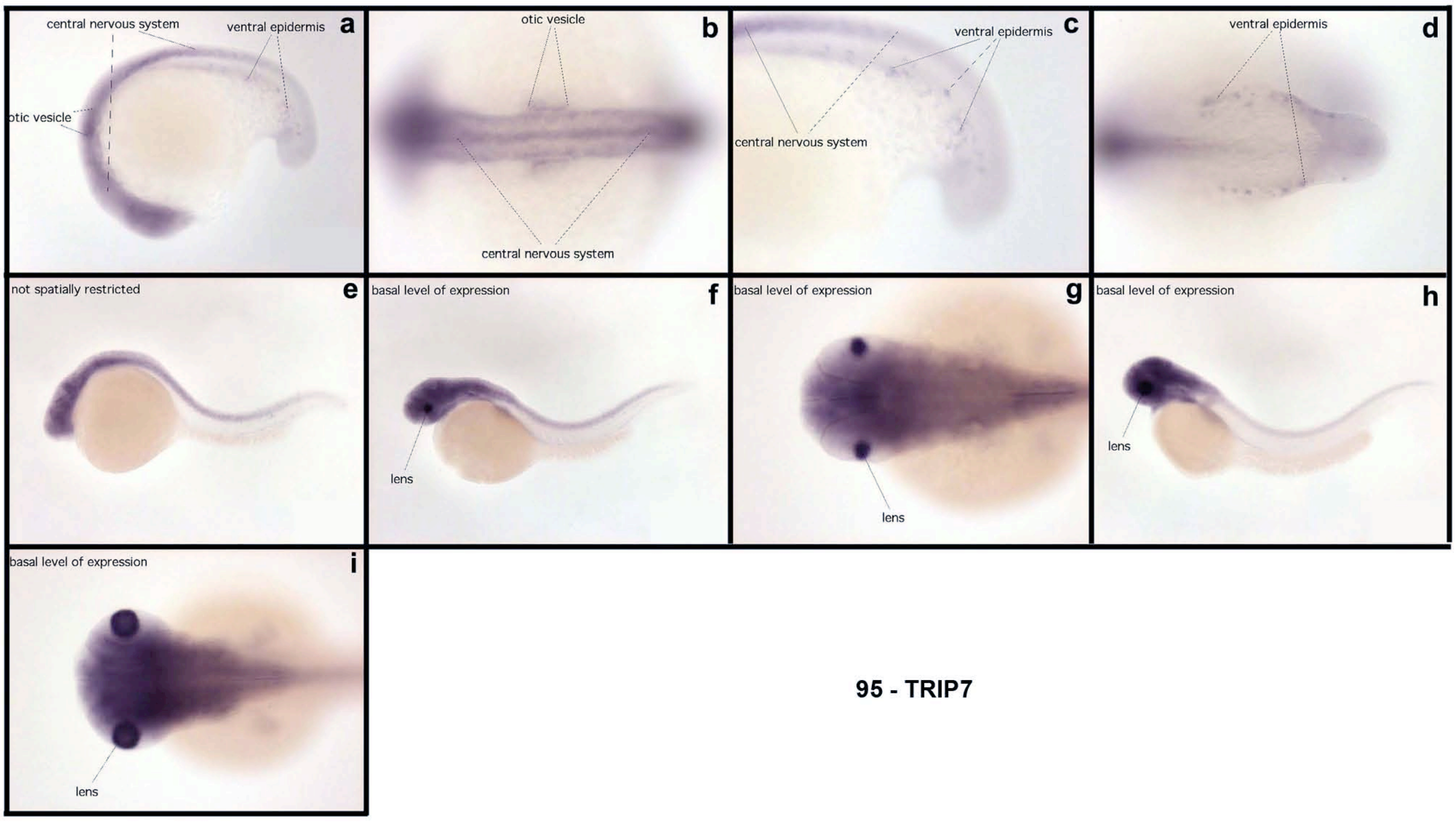
central nervous system
ventral epidermis
a
otic vesicle
otic vesicle
b
central nervous system
ventral epidermis
c
central nervous system
ventral epidermis
d
not spatially restricted
e
basal level of expression
f
lens
basal level of expression
g
lens
basal level of expression
h
lens
basal level of expression
i
lens

95 - TRIP7

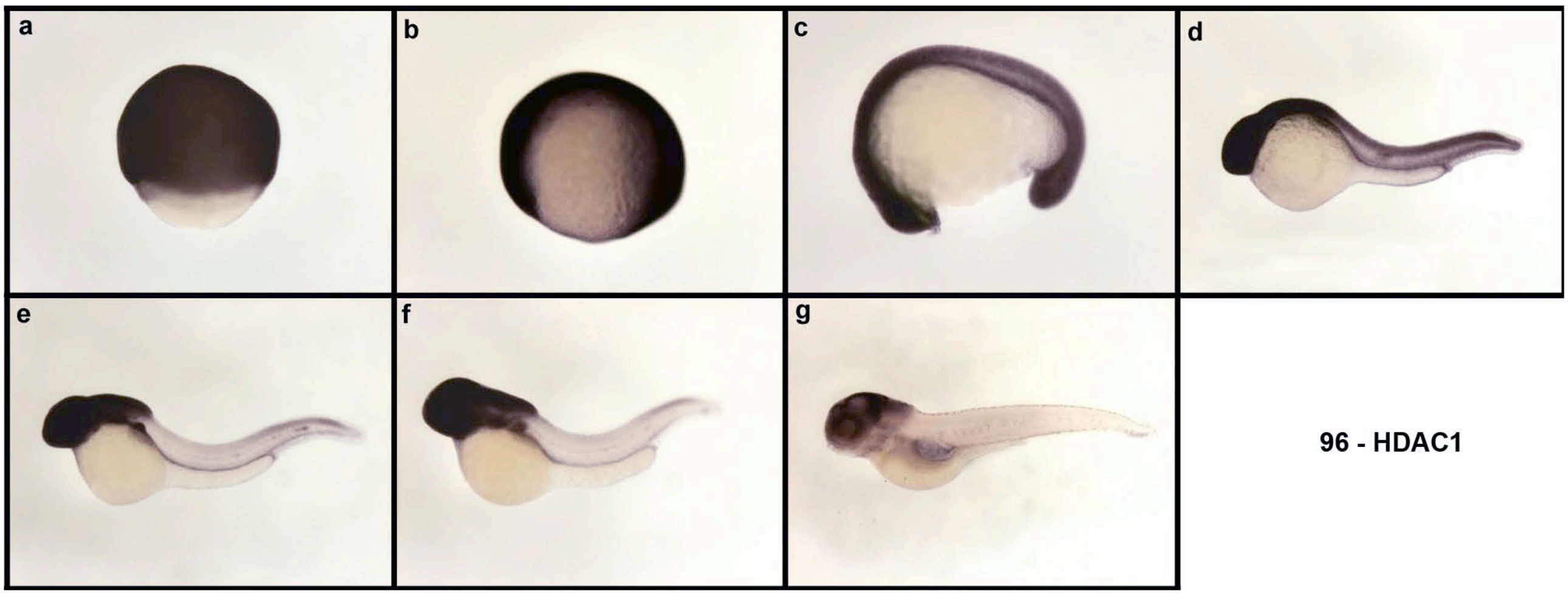

96 - HDAC1

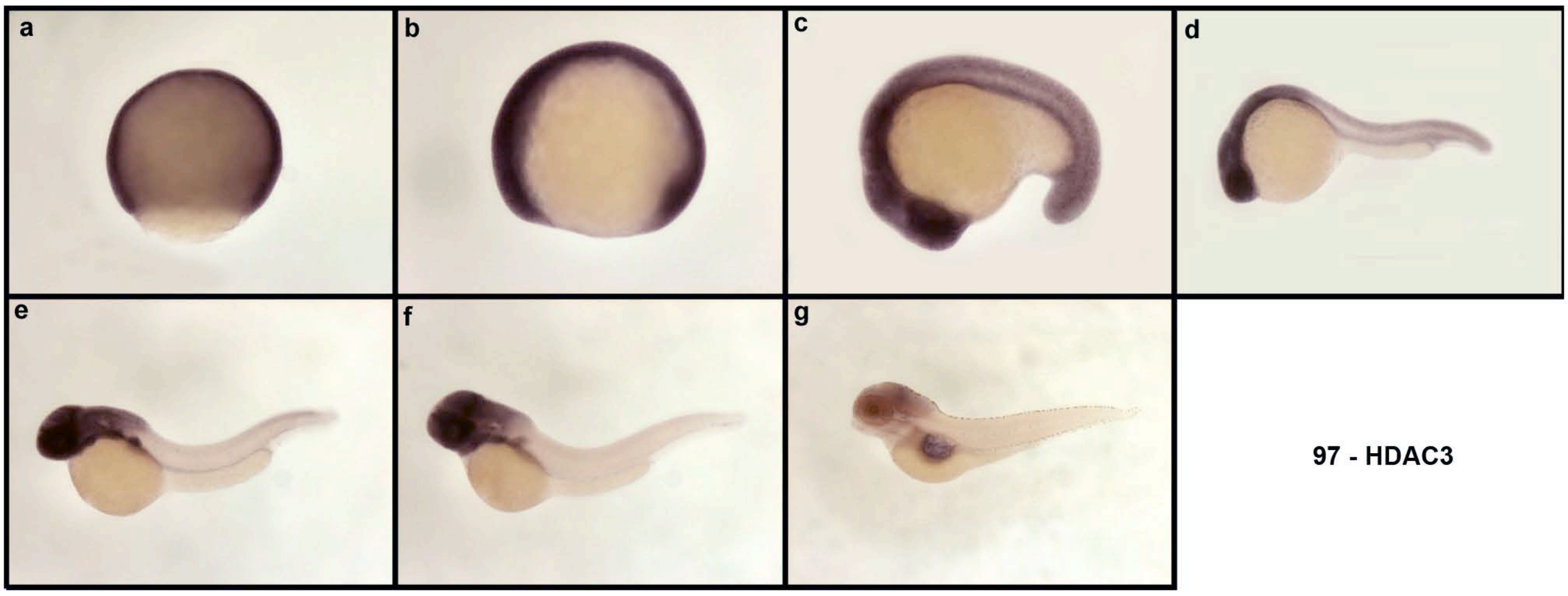

97 - HDAC3

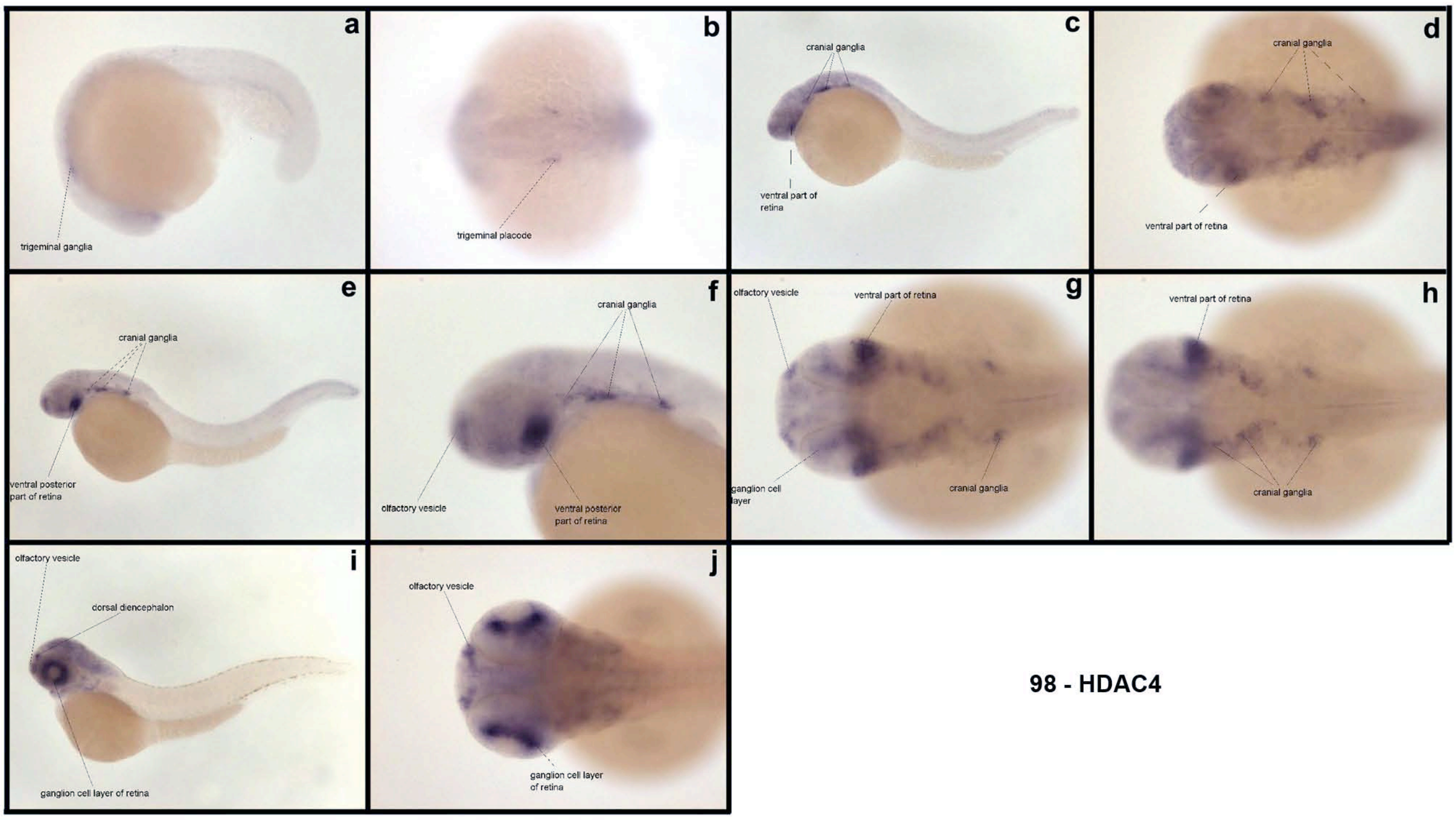
a
trigeminal ganglia
b
trigeminal placode
c
cranial ganglia
ventral part of retina
d
cranial ganglia
ventral part of retina
e
cranial ganglia
ventral posterior part of retina
f
cranial ganglia
olfactory vesicle
ventral posterior part of retina
g
olfactory vesicle
ventral part of retina
ganglion cell layer
cranial ganglia
h
ventral part of retina
cranial ganglia
i
olfactory vesicle
dorsal diencephalon
ganglion cell layer of retina
j
olfactory vesicle
ganglion cell layer of retina

98 - HDAC4

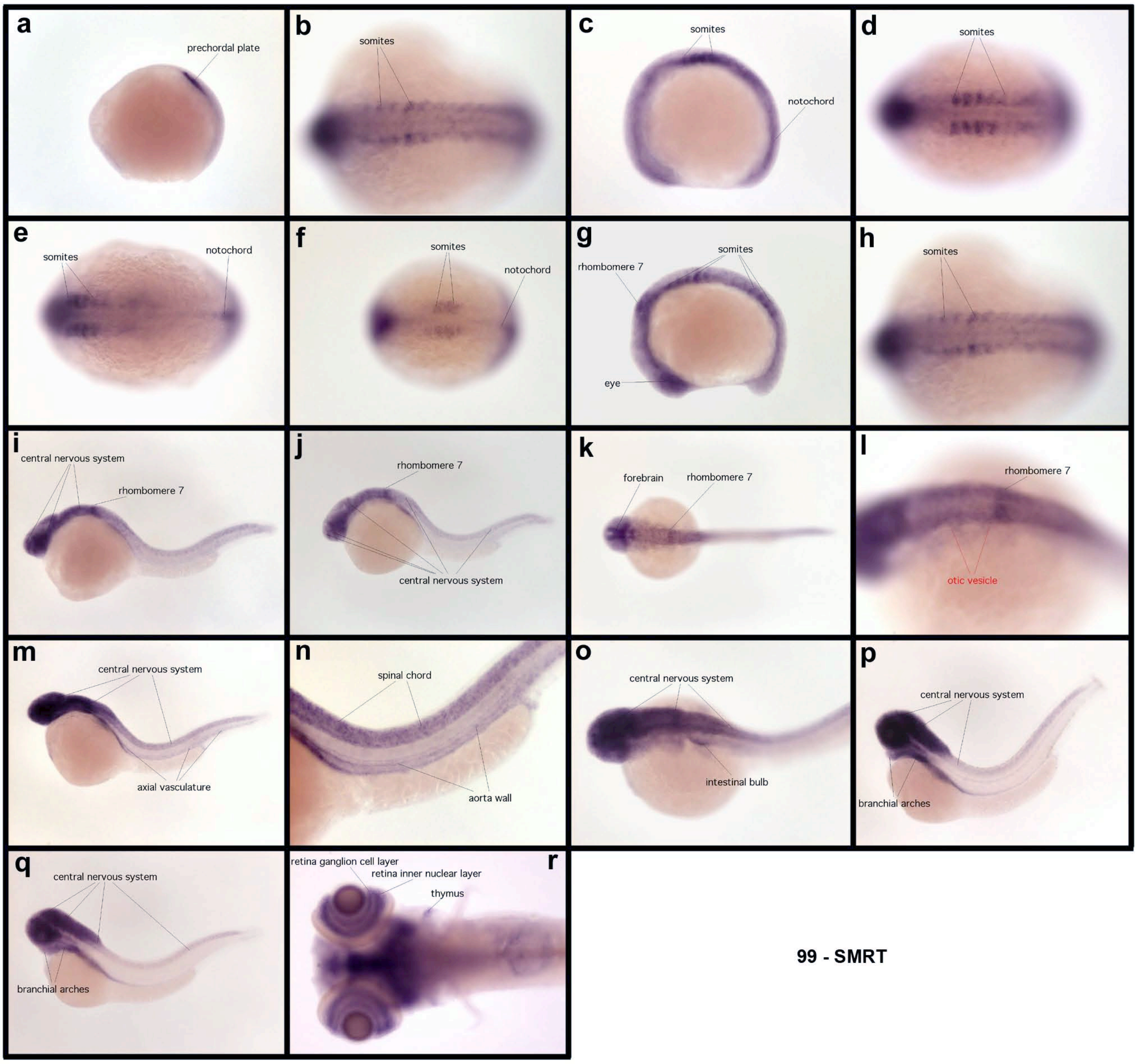
a
prechordal plate
b
somites
c
somites
notochord
d
somites
e
somites
notochord
f
somites
notochord
g
rhombomere 7
somites
eye
h
somites
i
central nervous system
rhombomere 7
j
rhombomere 7
central nervous system
k
forebrain
rhombomere 7
l
rhombomere 7
otic vesicle
m
central nervous system
axial vasculature
n
spinal chord
aorta wall
o
central nervous system
intestinal bulb
p
central nervous system
branchial arches
q
central nervous system
branchial arches
retina ganglion cell layer
retina inner nuclear layer
thymus
r

99 - SMRT

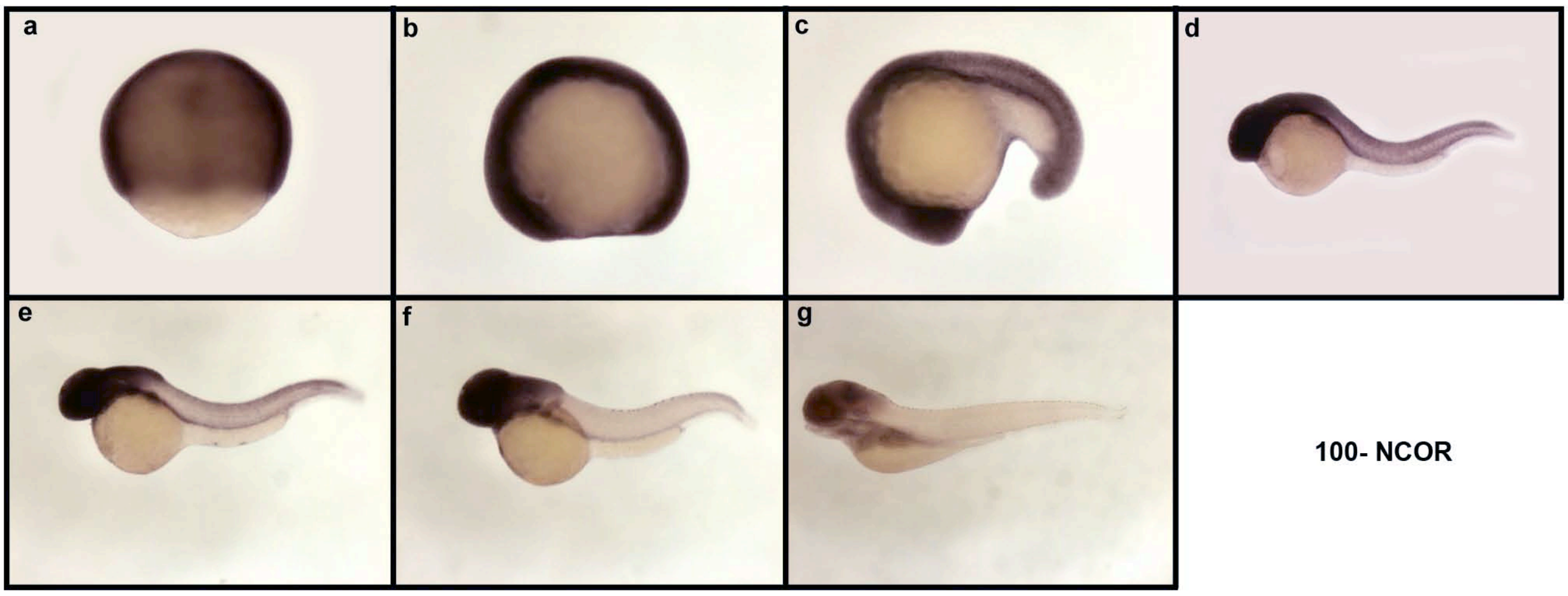

100- NCOR

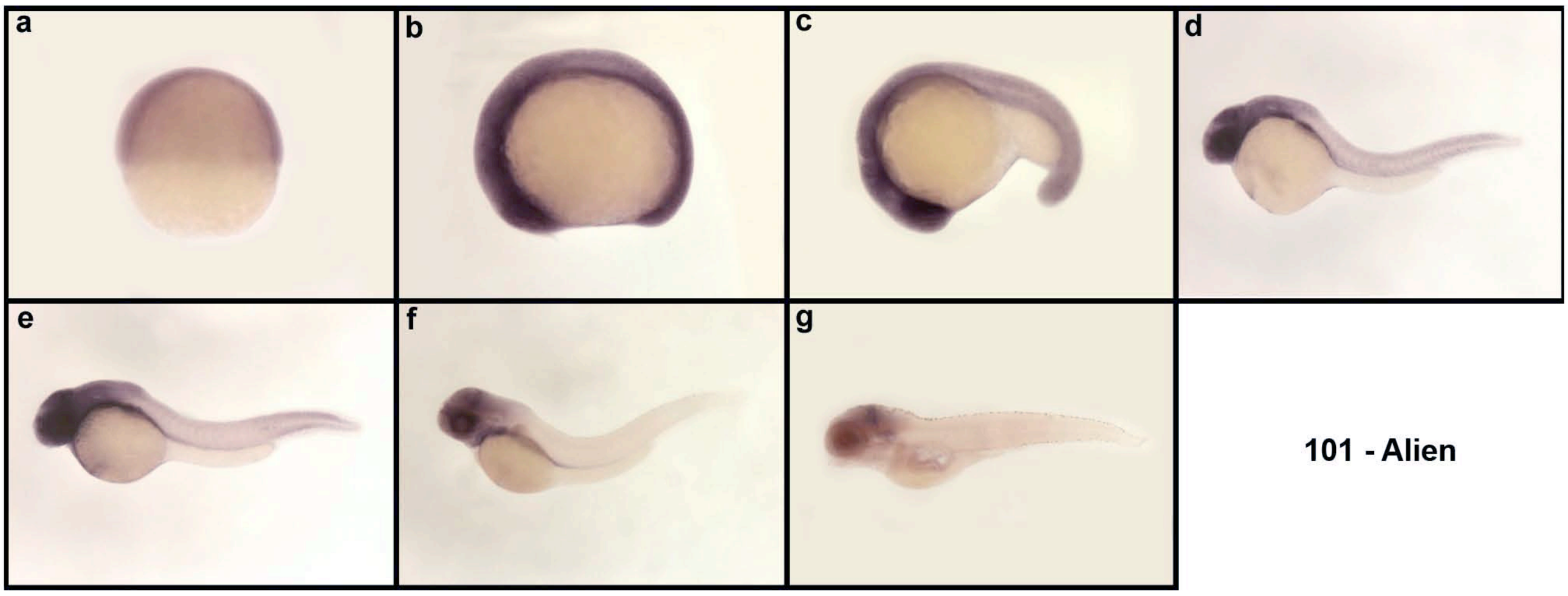

101 - Alien
