# Supplementary material for: Unexpected Novel Relational Links Uncovered by Extensive Developmental Profiling of Nuclear Receptor Expression
Source: PLoS Genet. 2007 Nov 9;3(11):e188. doi: 10.1371/journal.pgen.0030188 (PMC2065881; doi:10.1371/journal.pgen.0030188)
Supplement: Supplementary file 3 [file pgen.0030188.sg004.pdf]

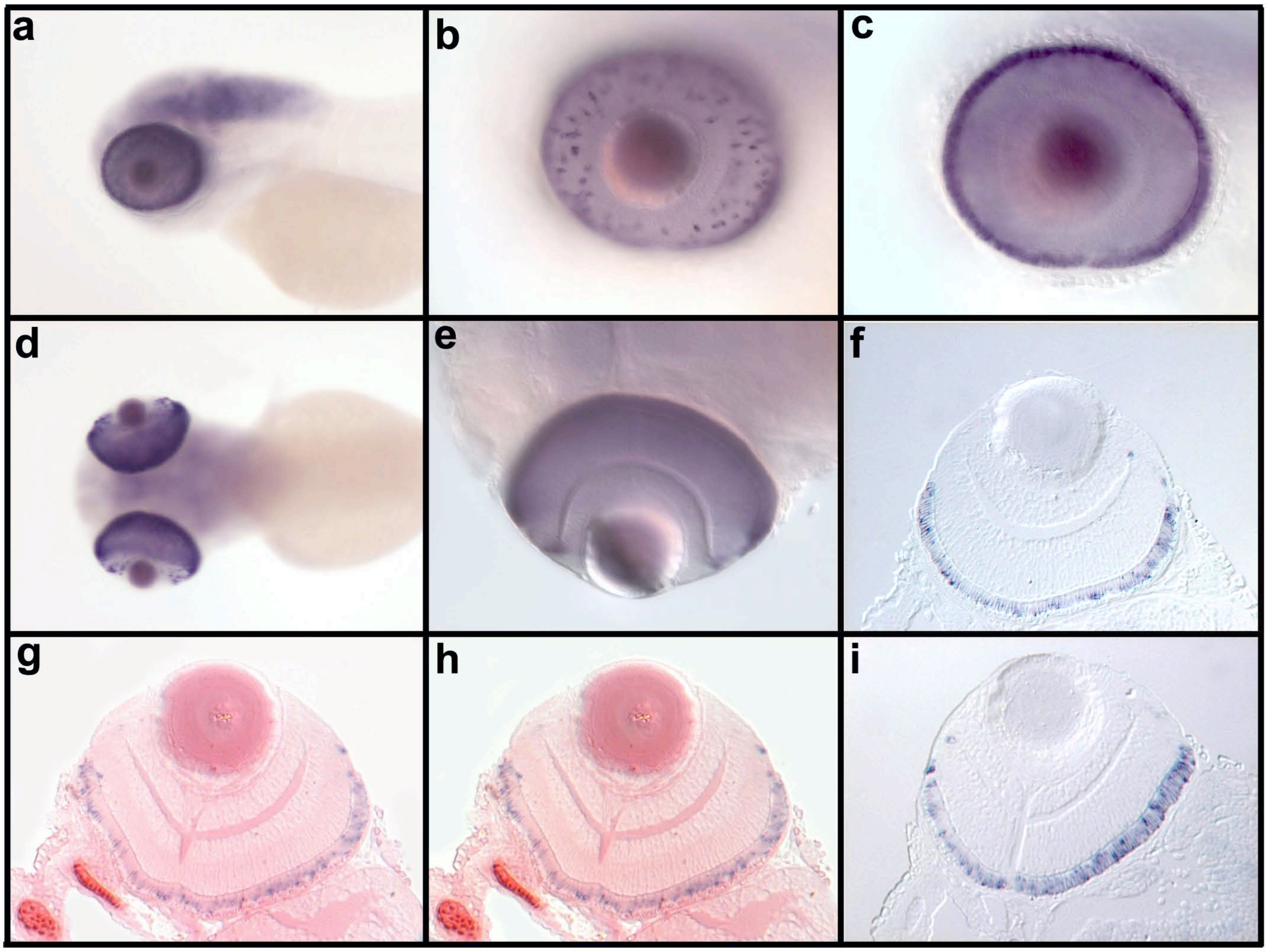

1 - TRβ

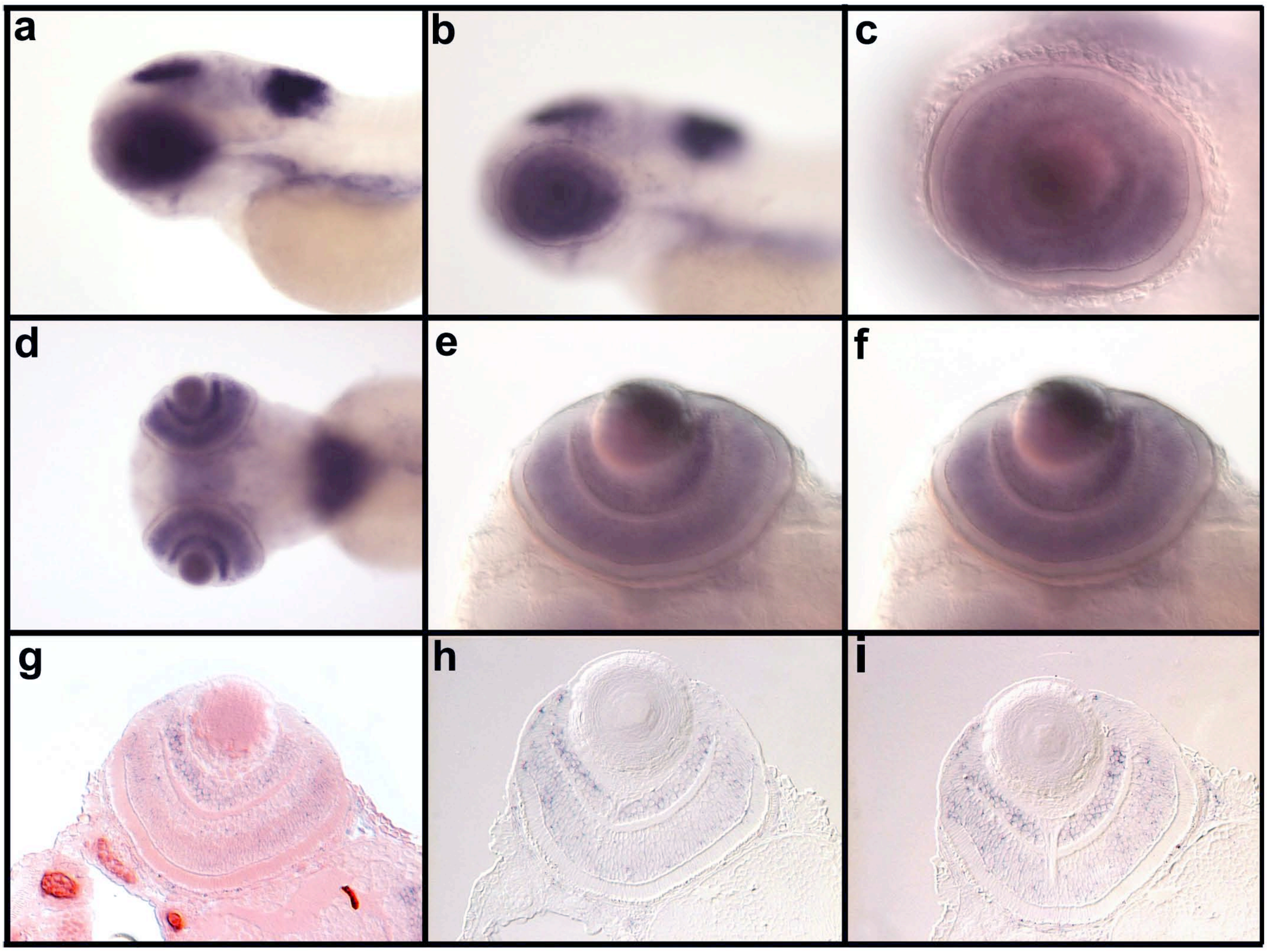

2 - RARα-A

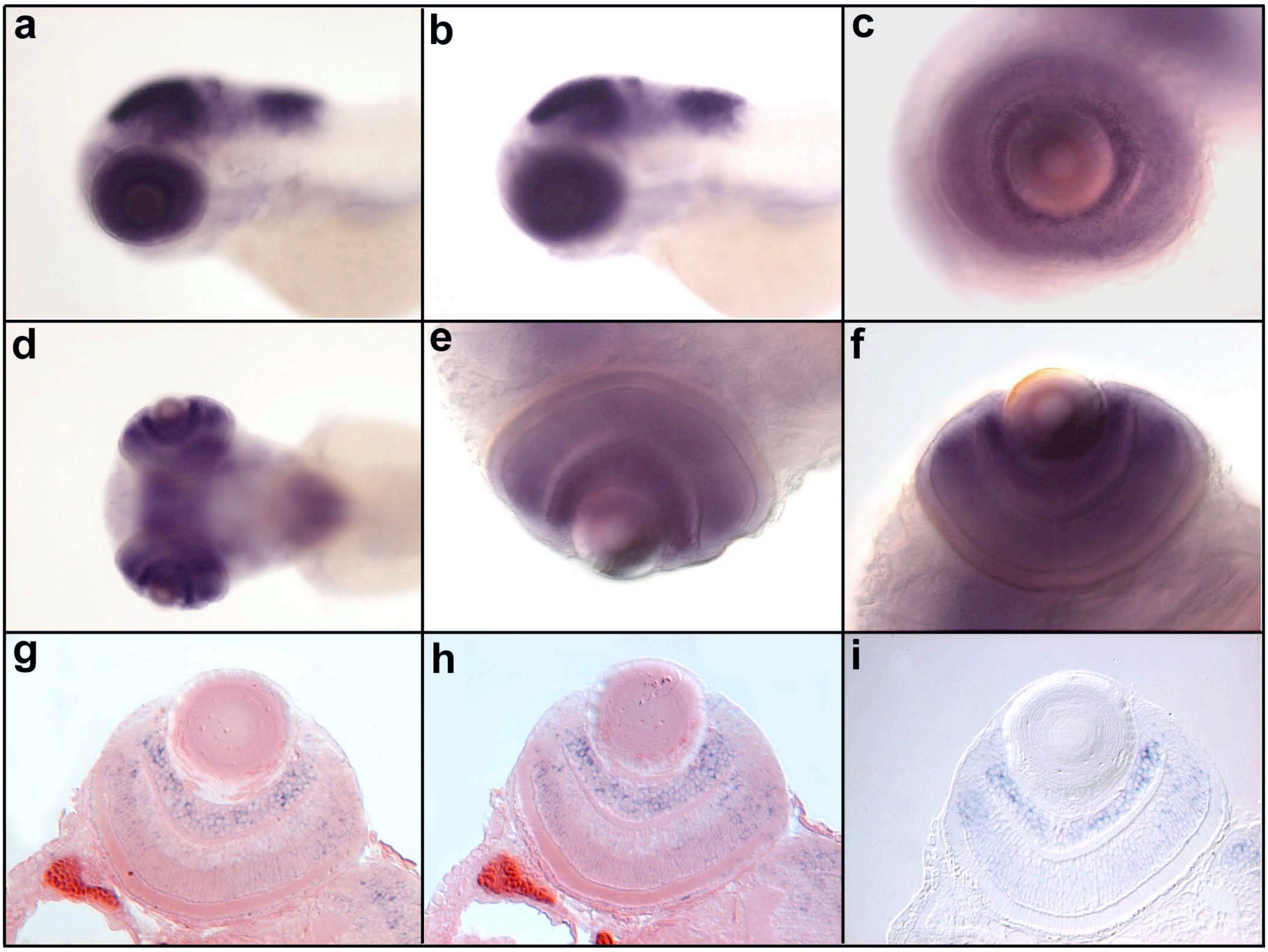

3 - RARα-B

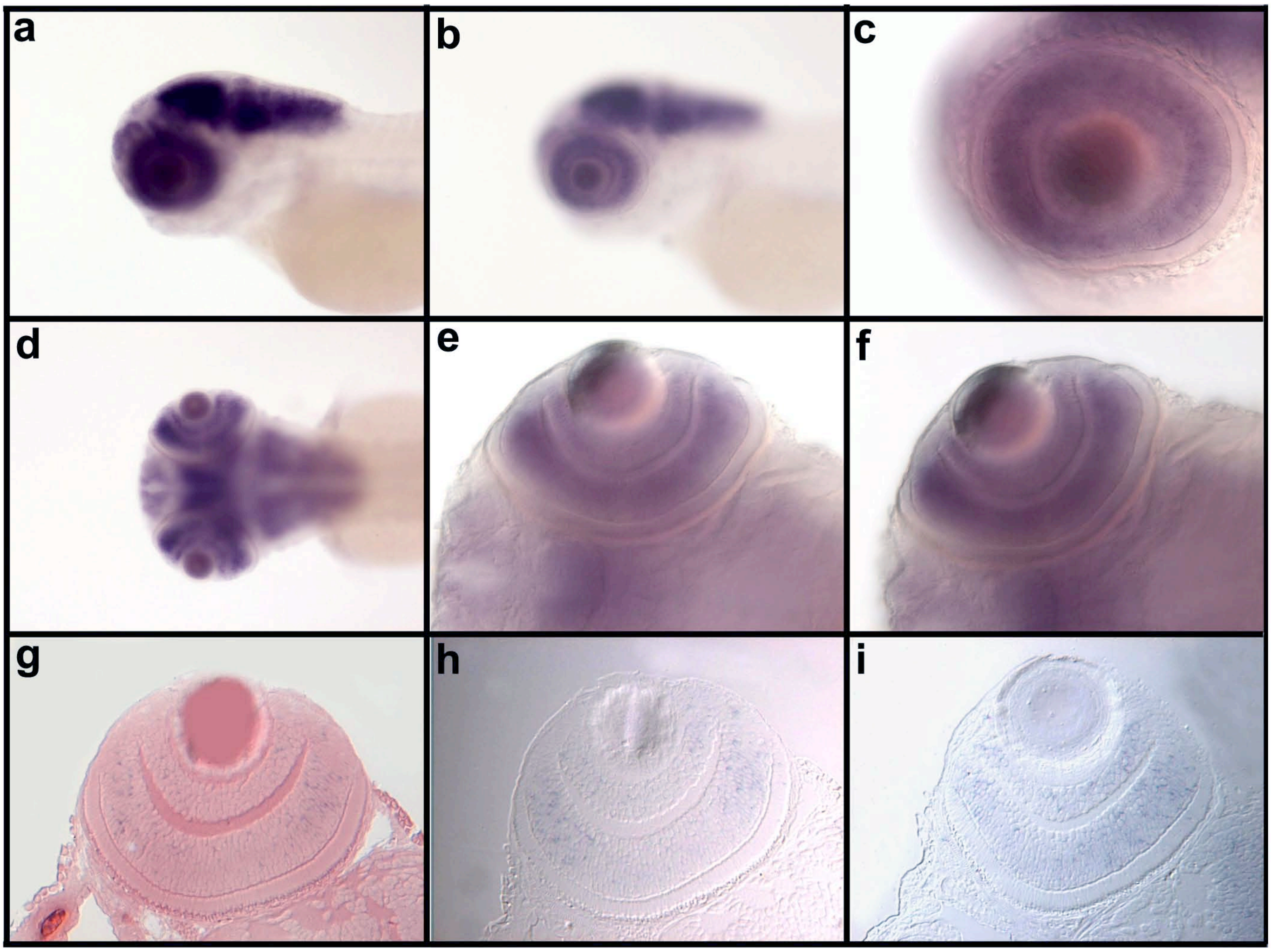

4 - Reverbα

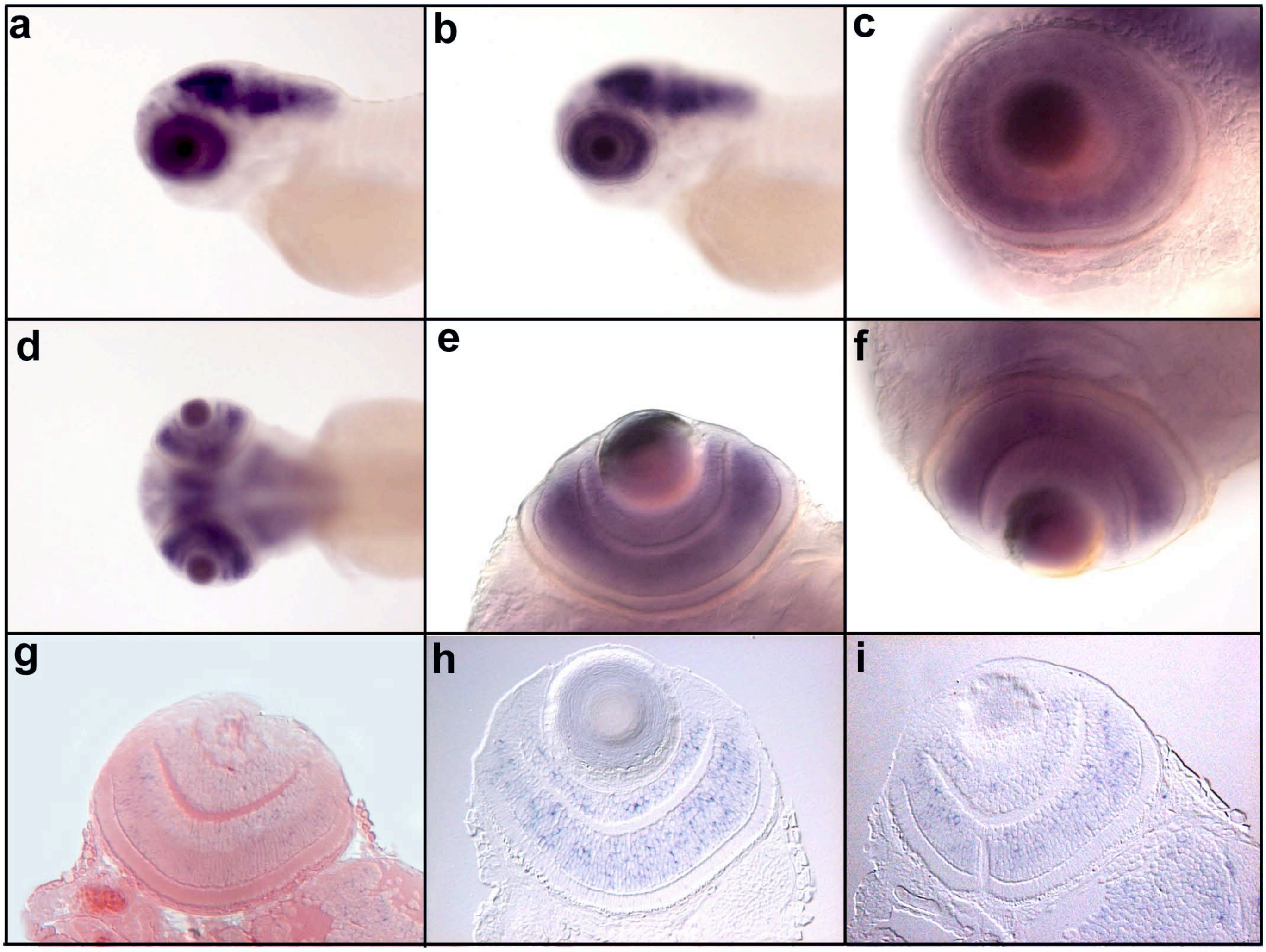

5 - ReverbβA

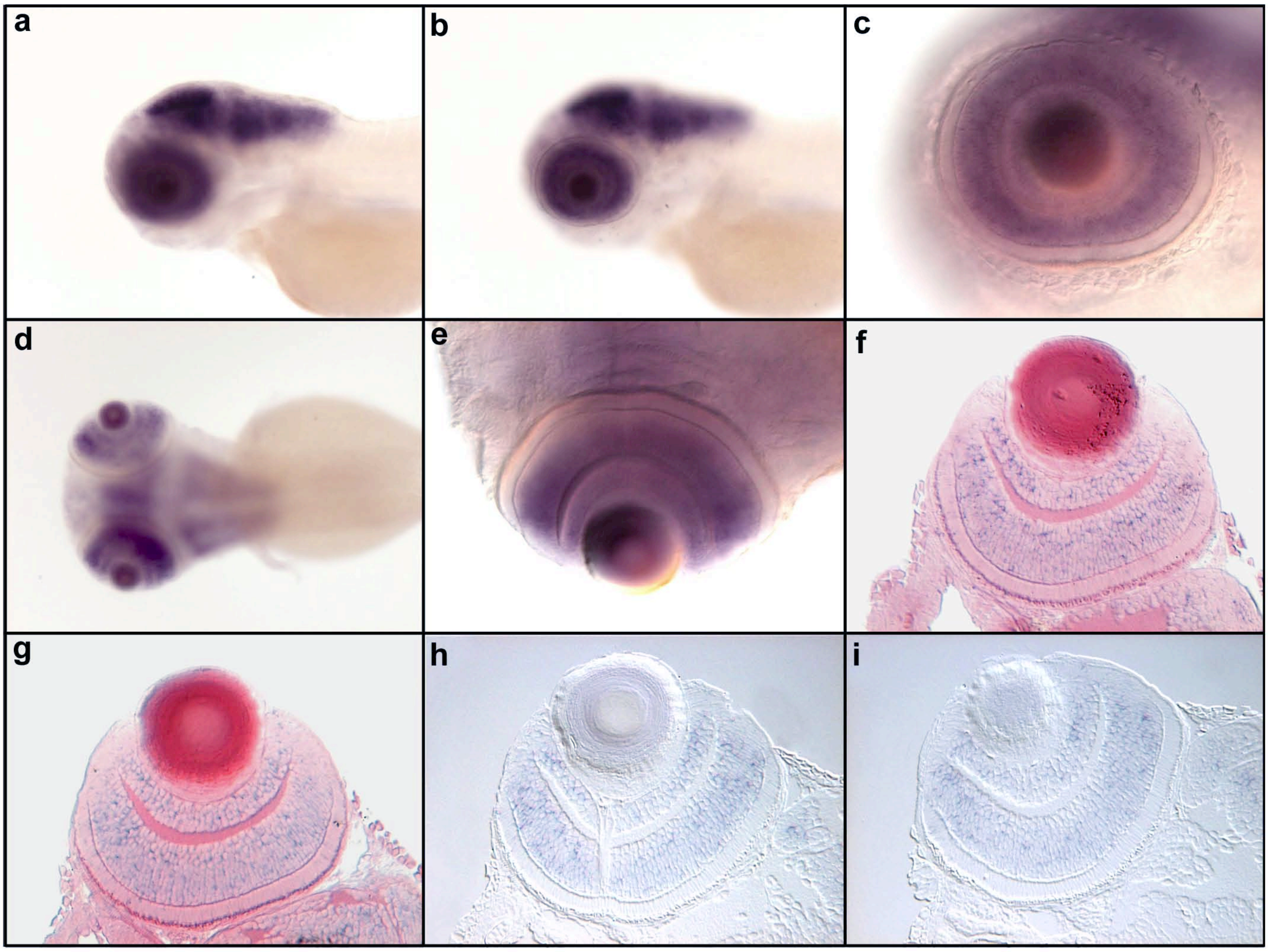

6 - Reverbβ-B

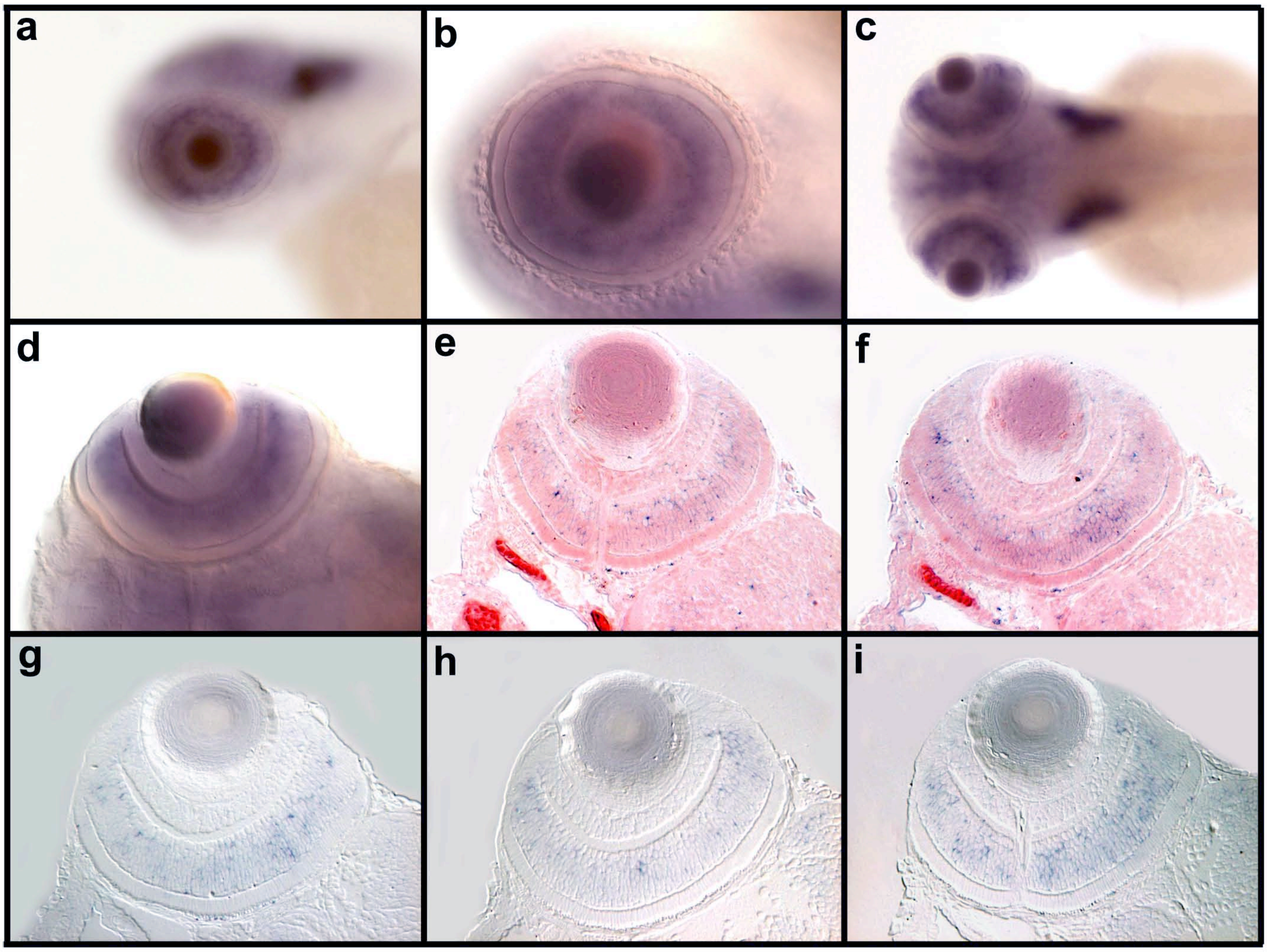

7 - Reverbγ-B

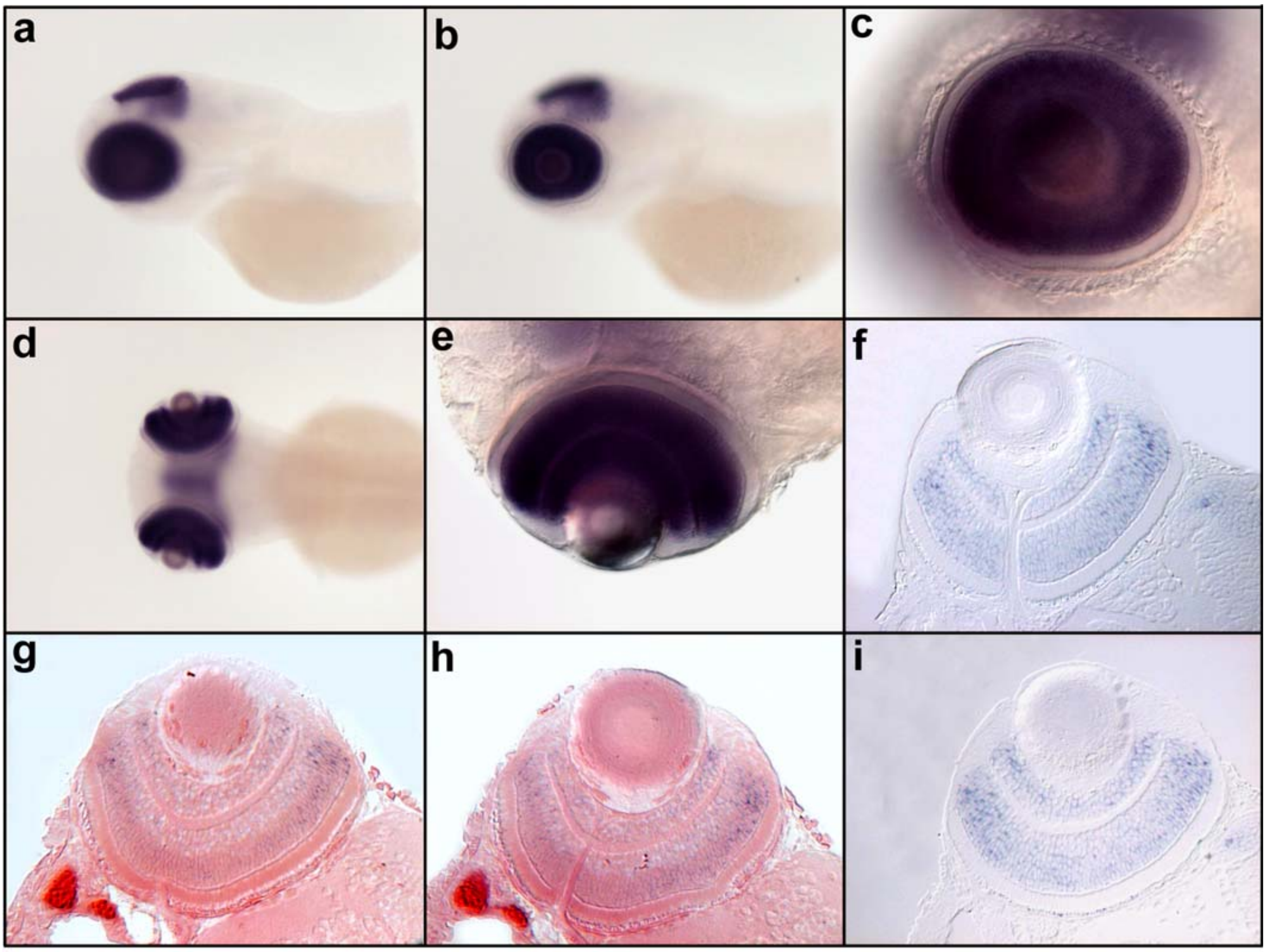

8 - RORα-A

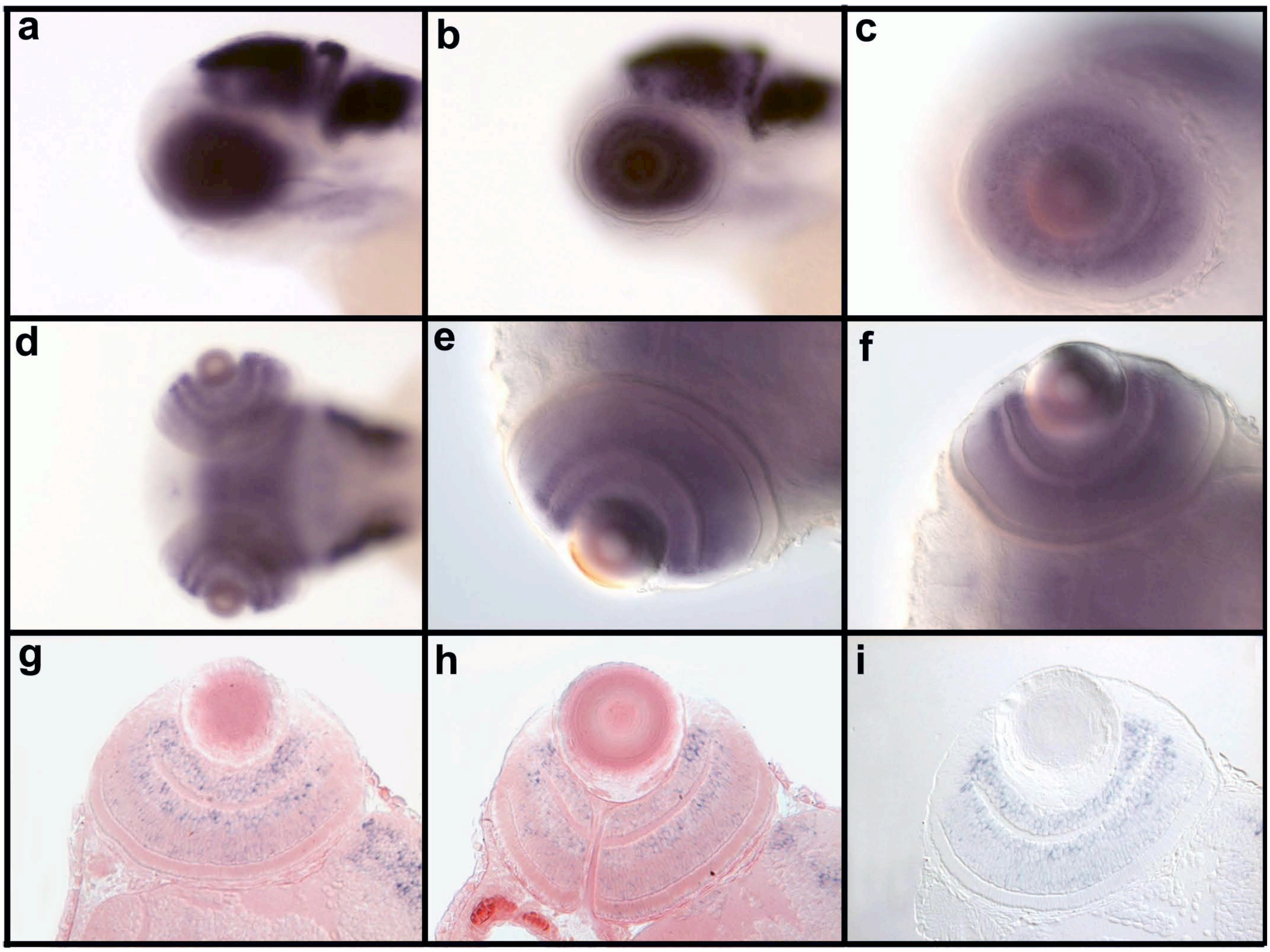

9 - RORα-B

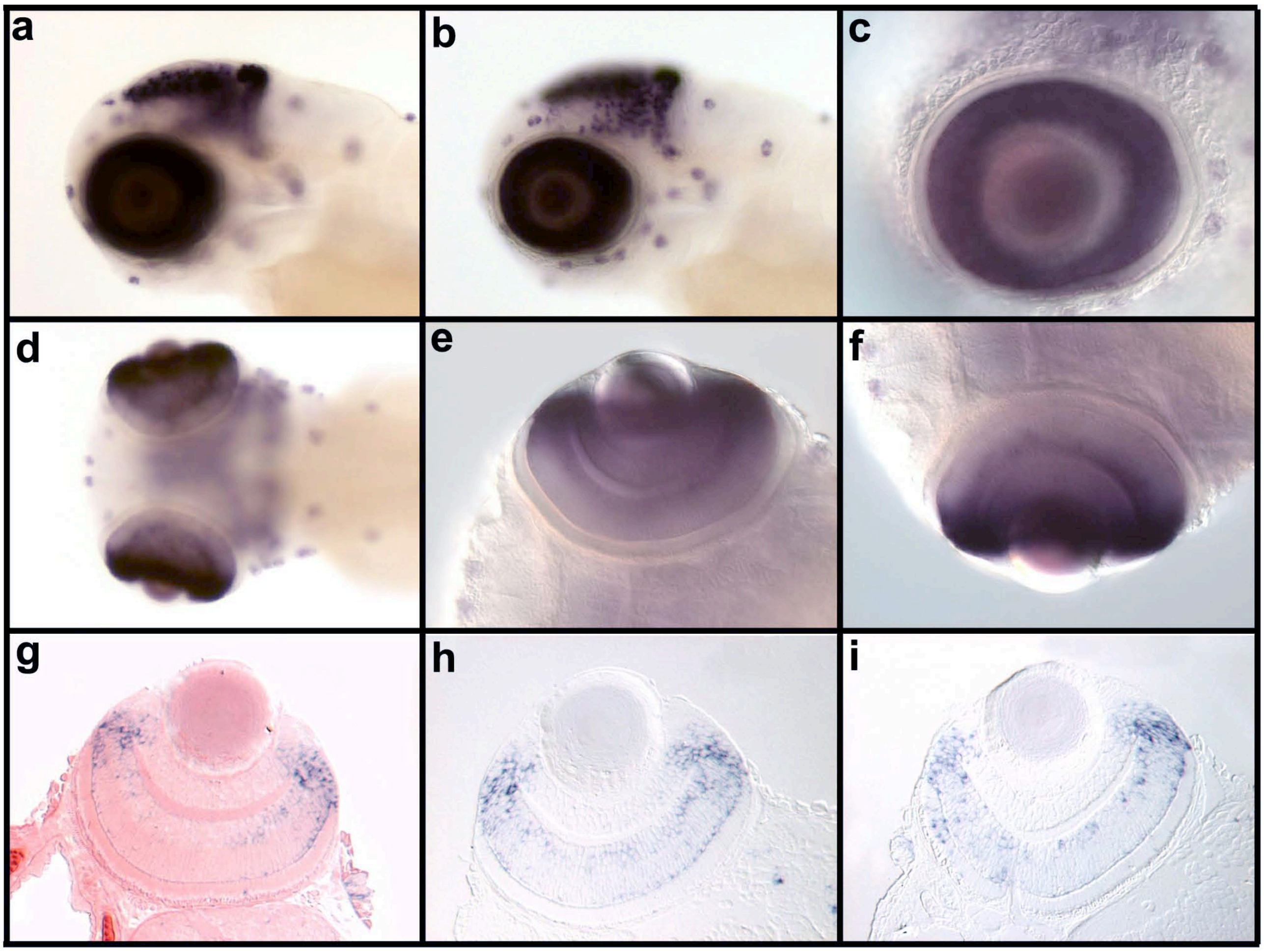

10 - RORβ

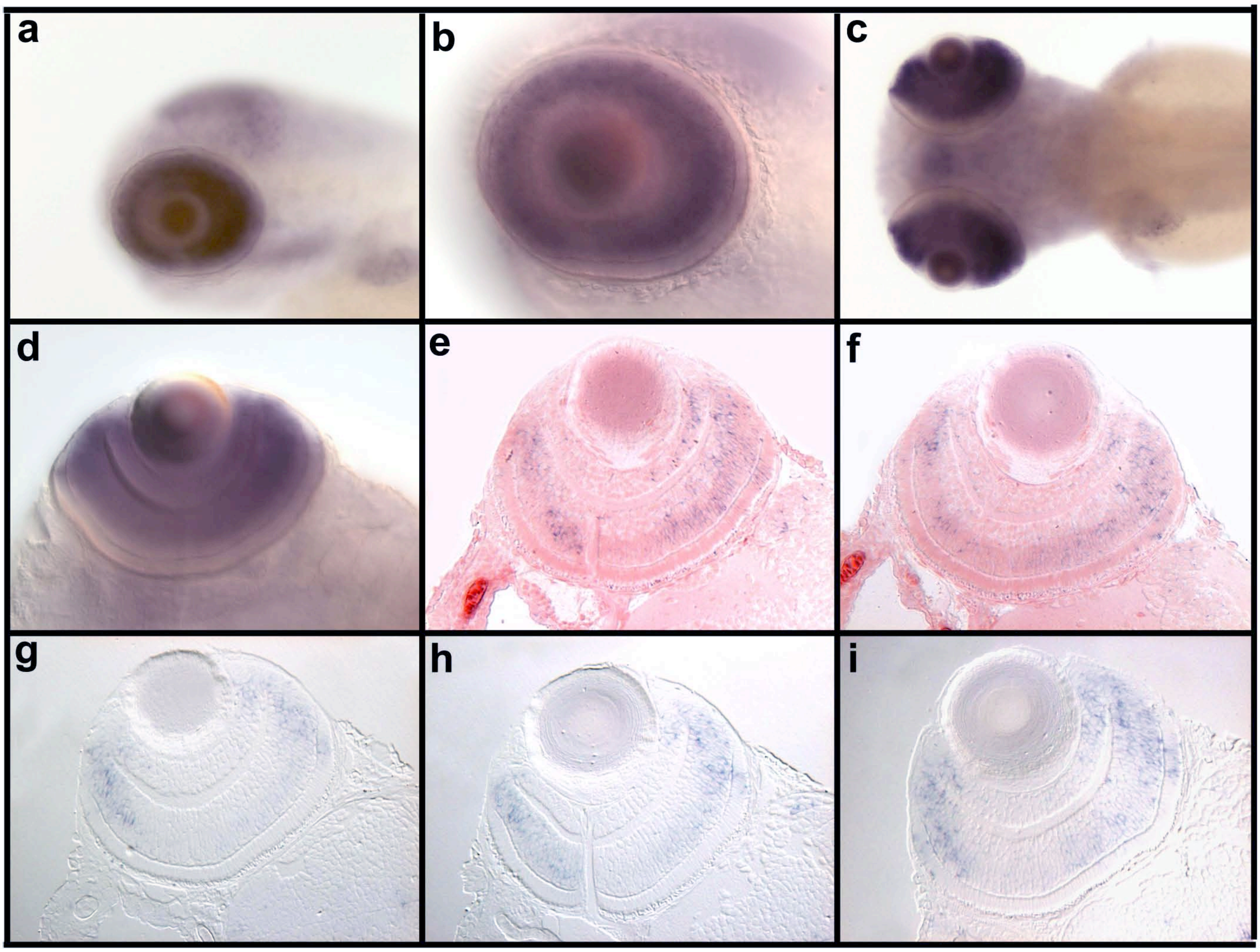

11 - RORγ-A

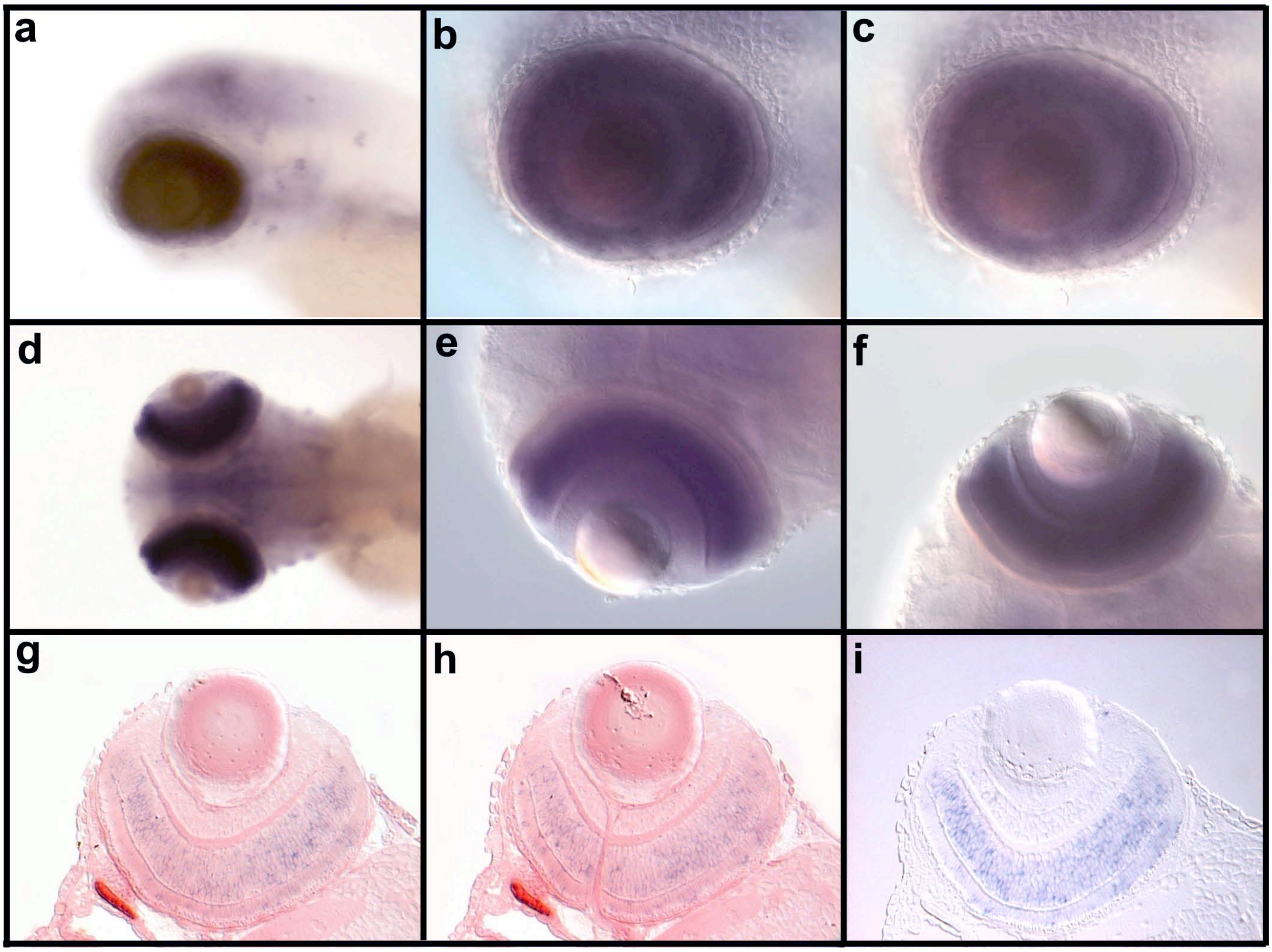

12 - RORγ-B

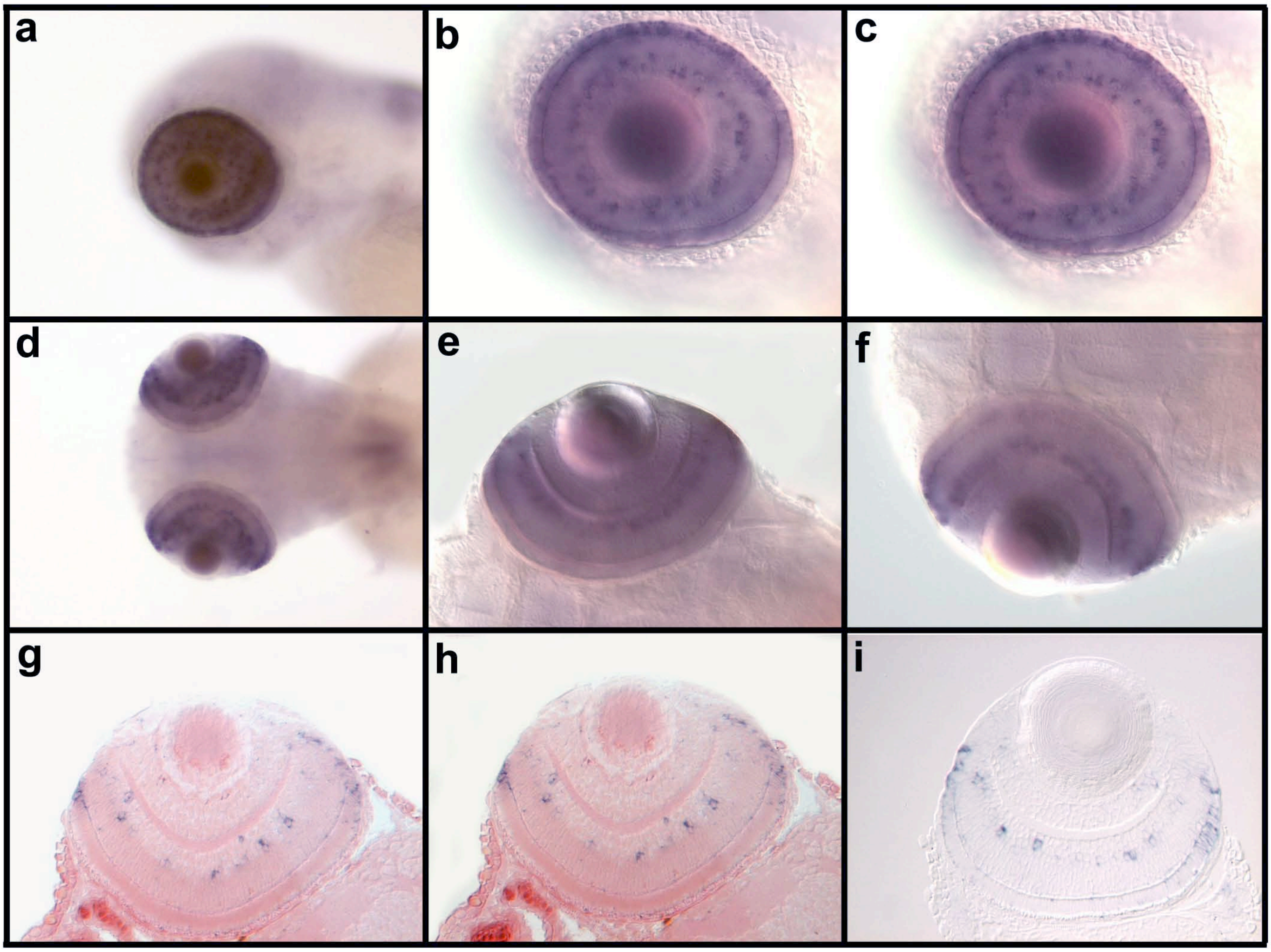

13 - RXRα-A

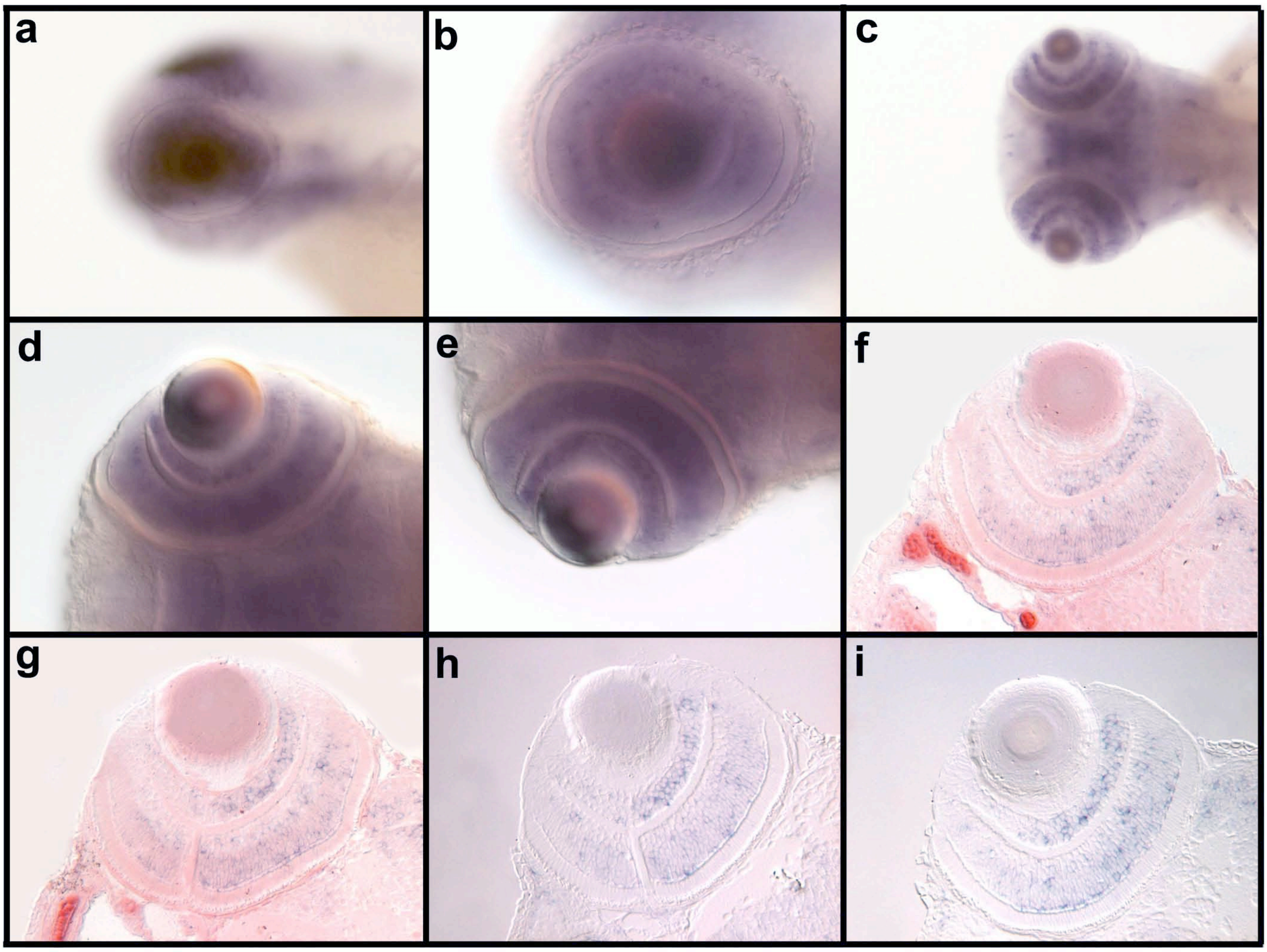

14 - RXRα-B

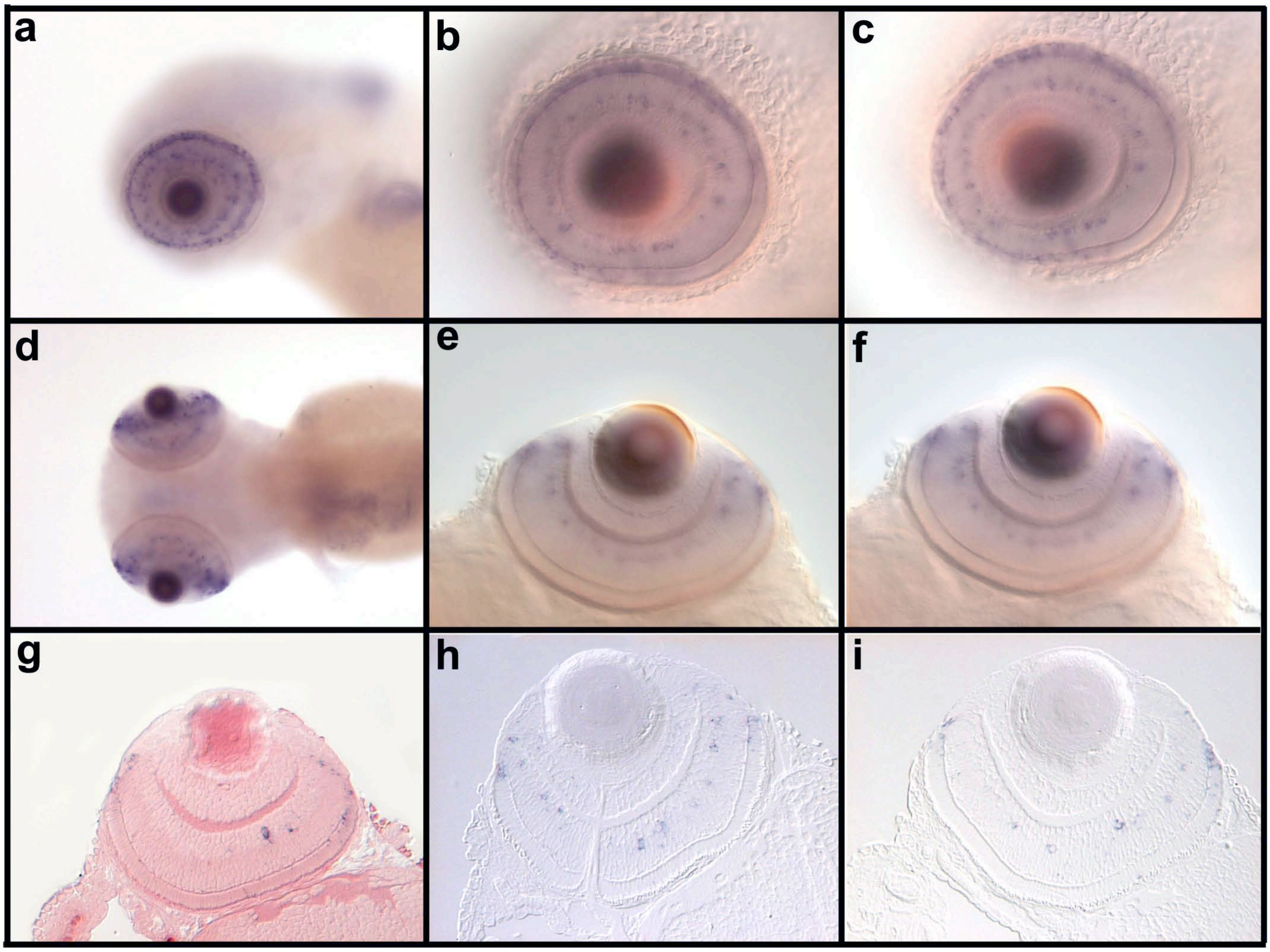

15 - RXRγ

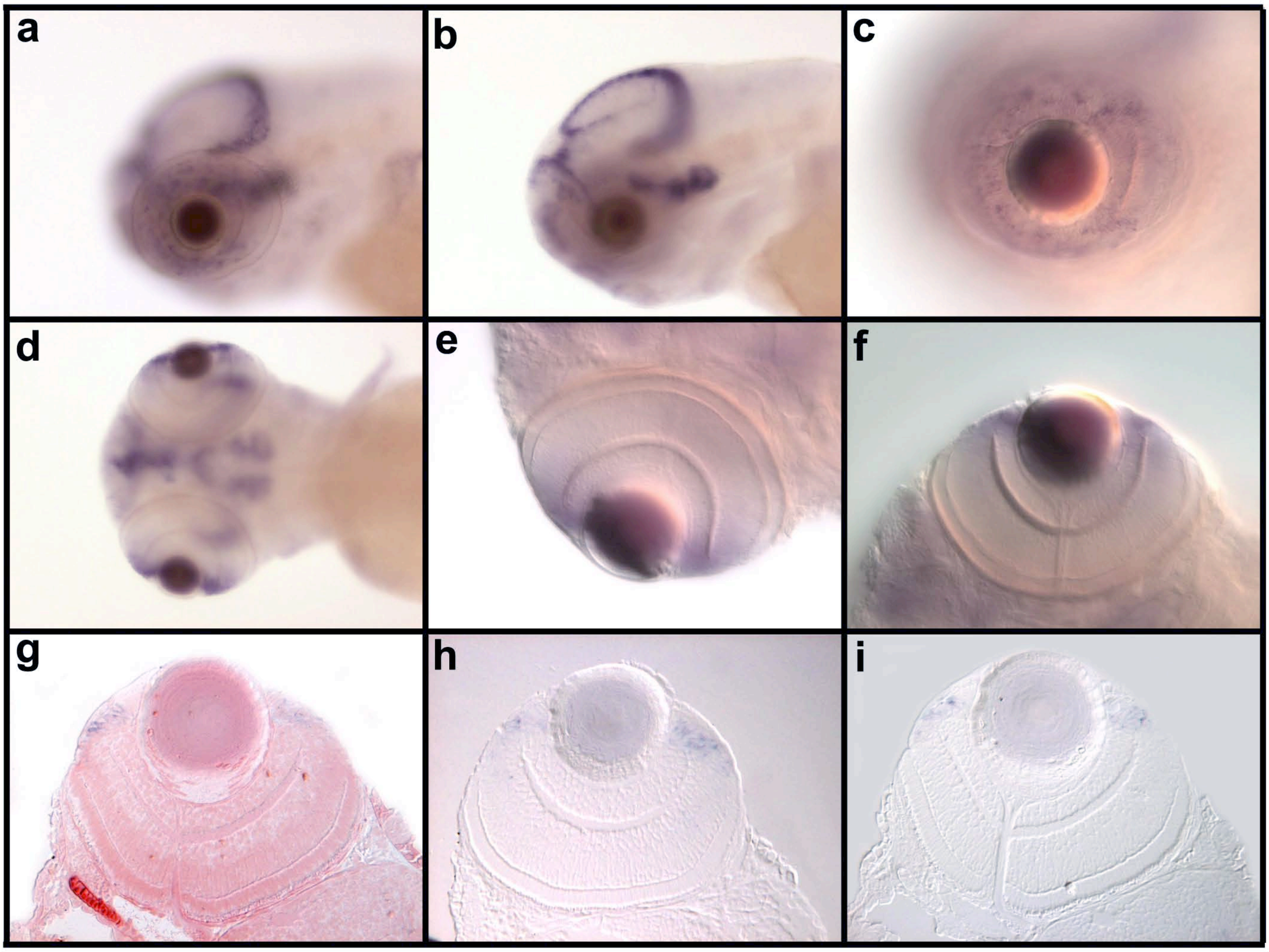

16 - TLL

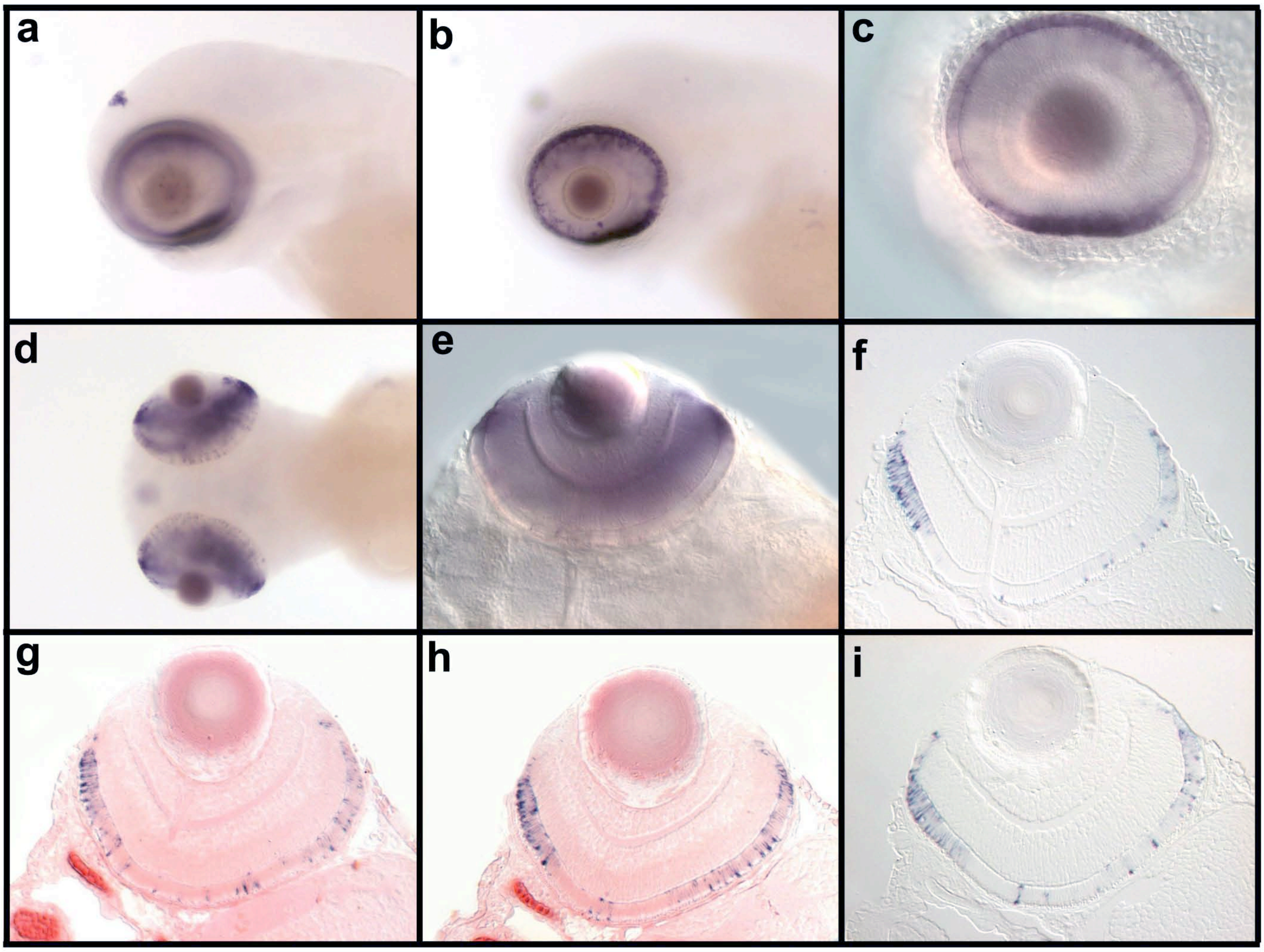

17 - PNR

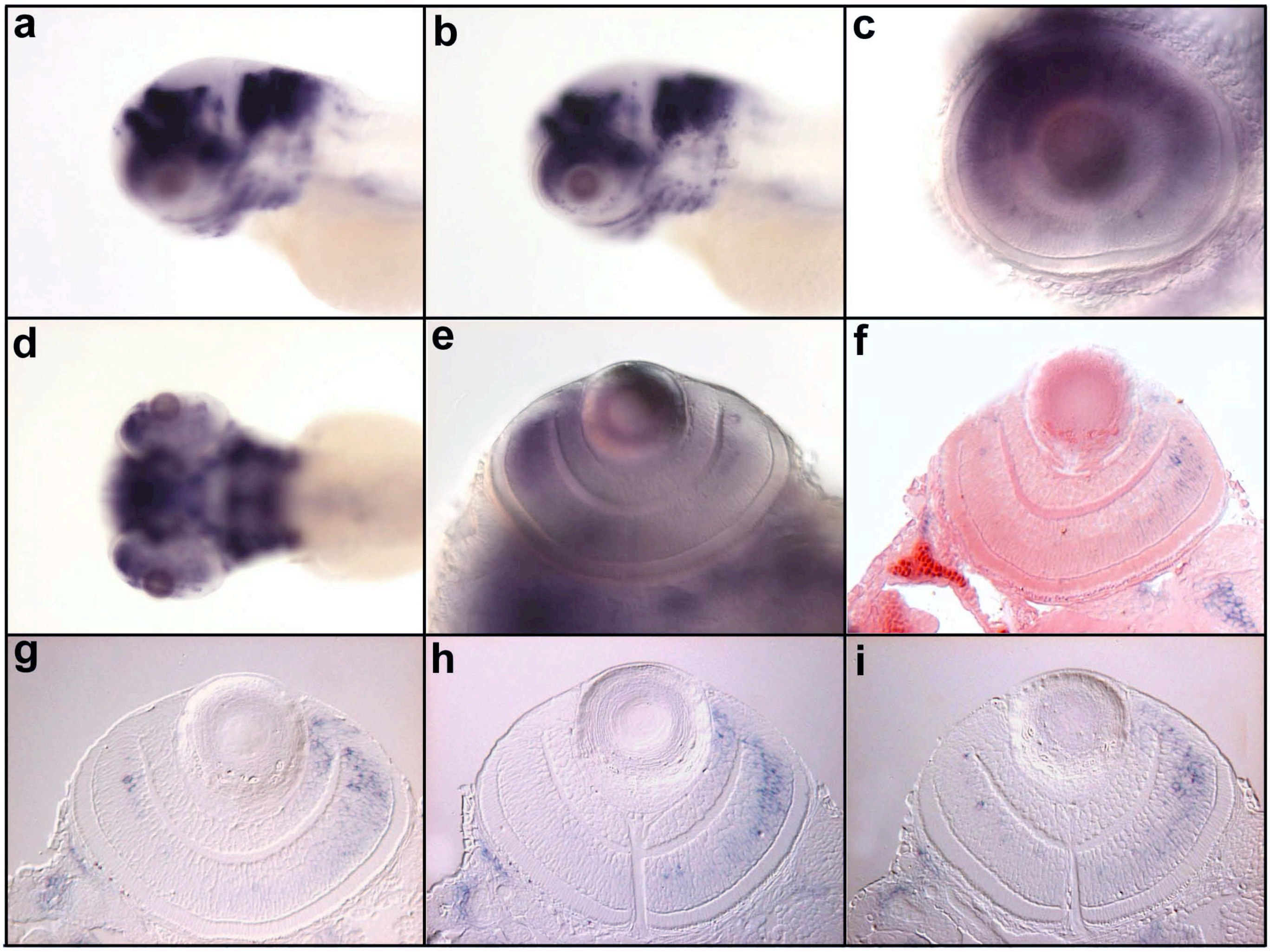

18 - COUPTFα-B

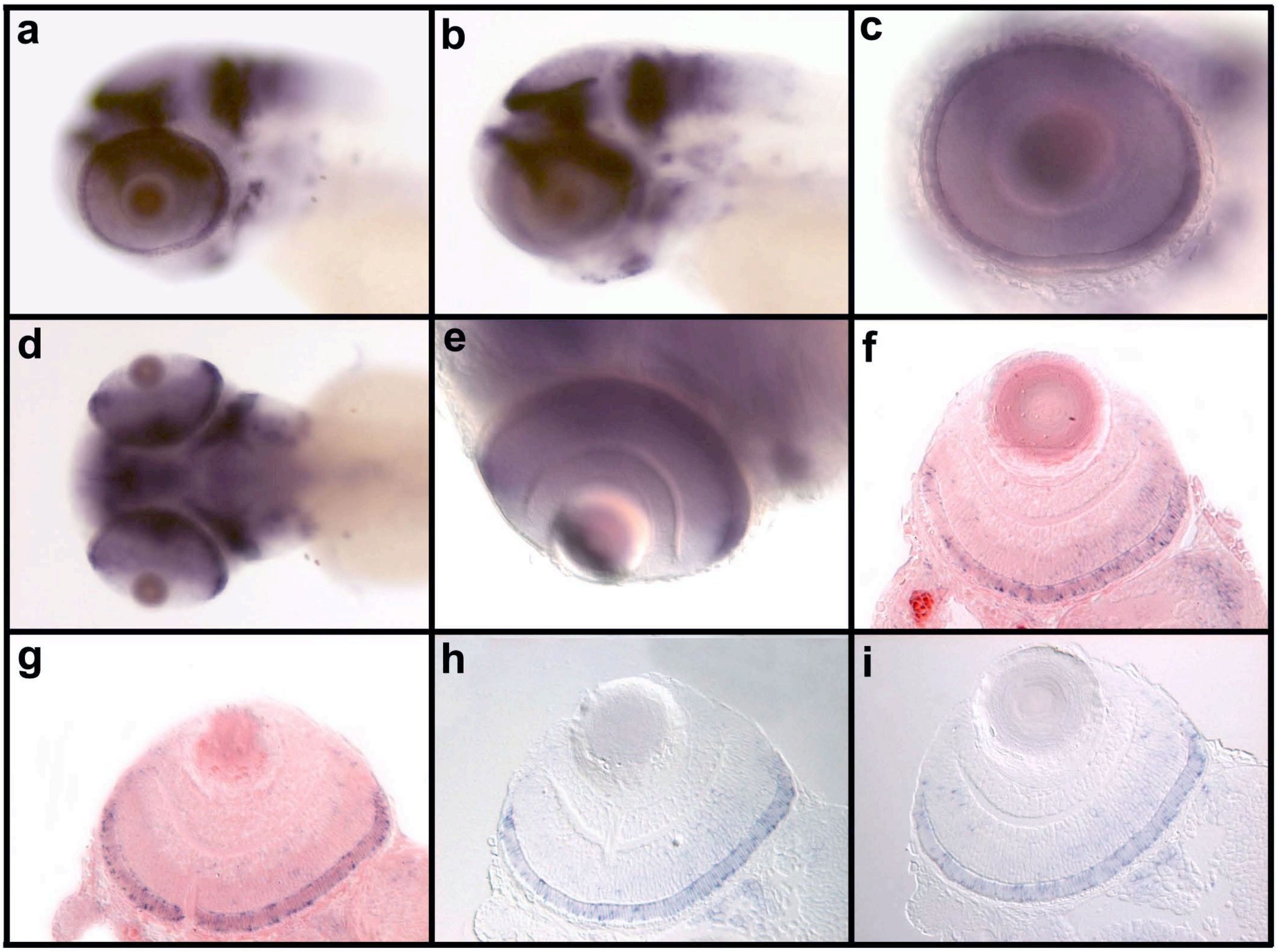

19 - COUPTFβ

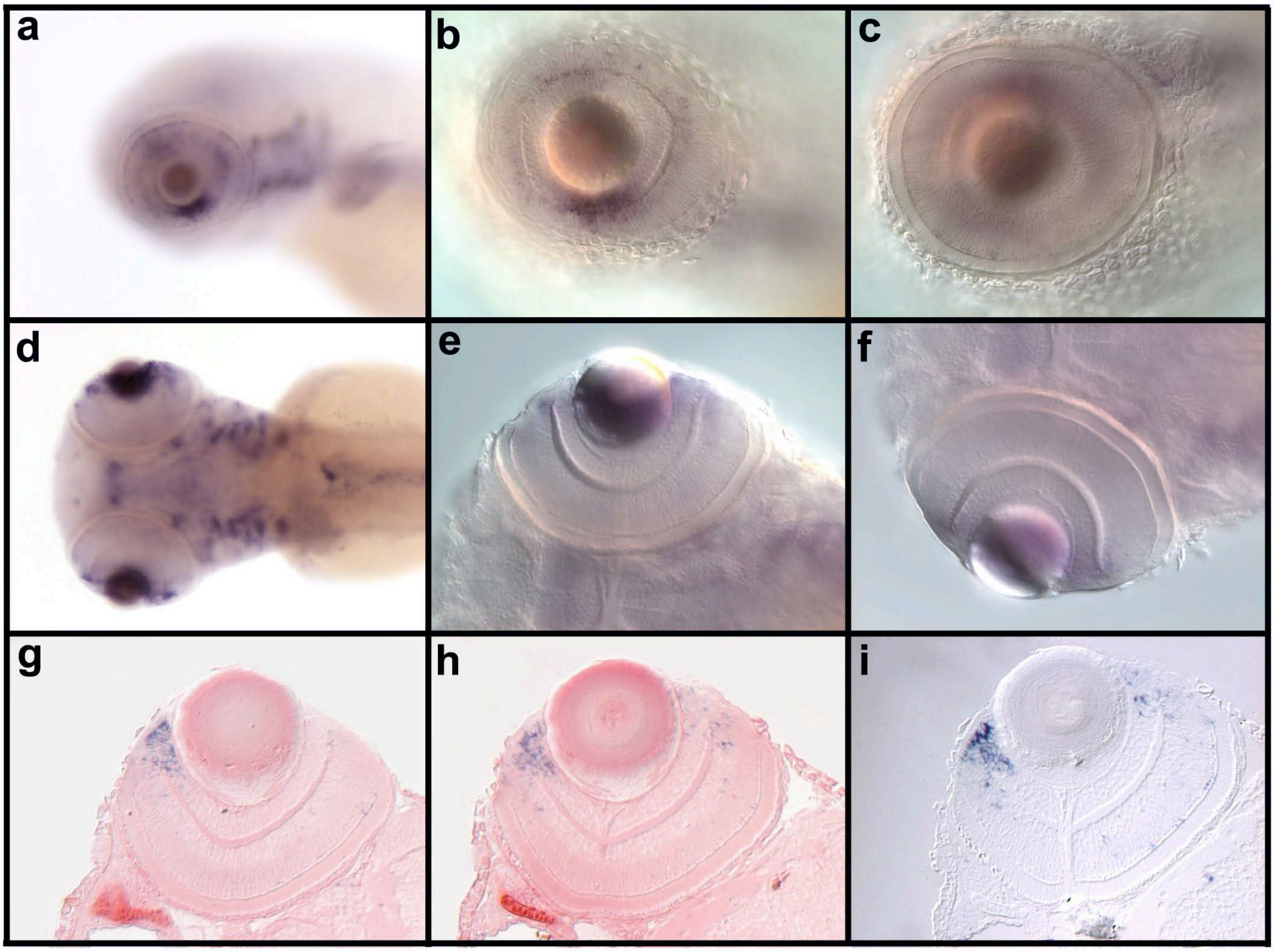

20 - COUPTFγ

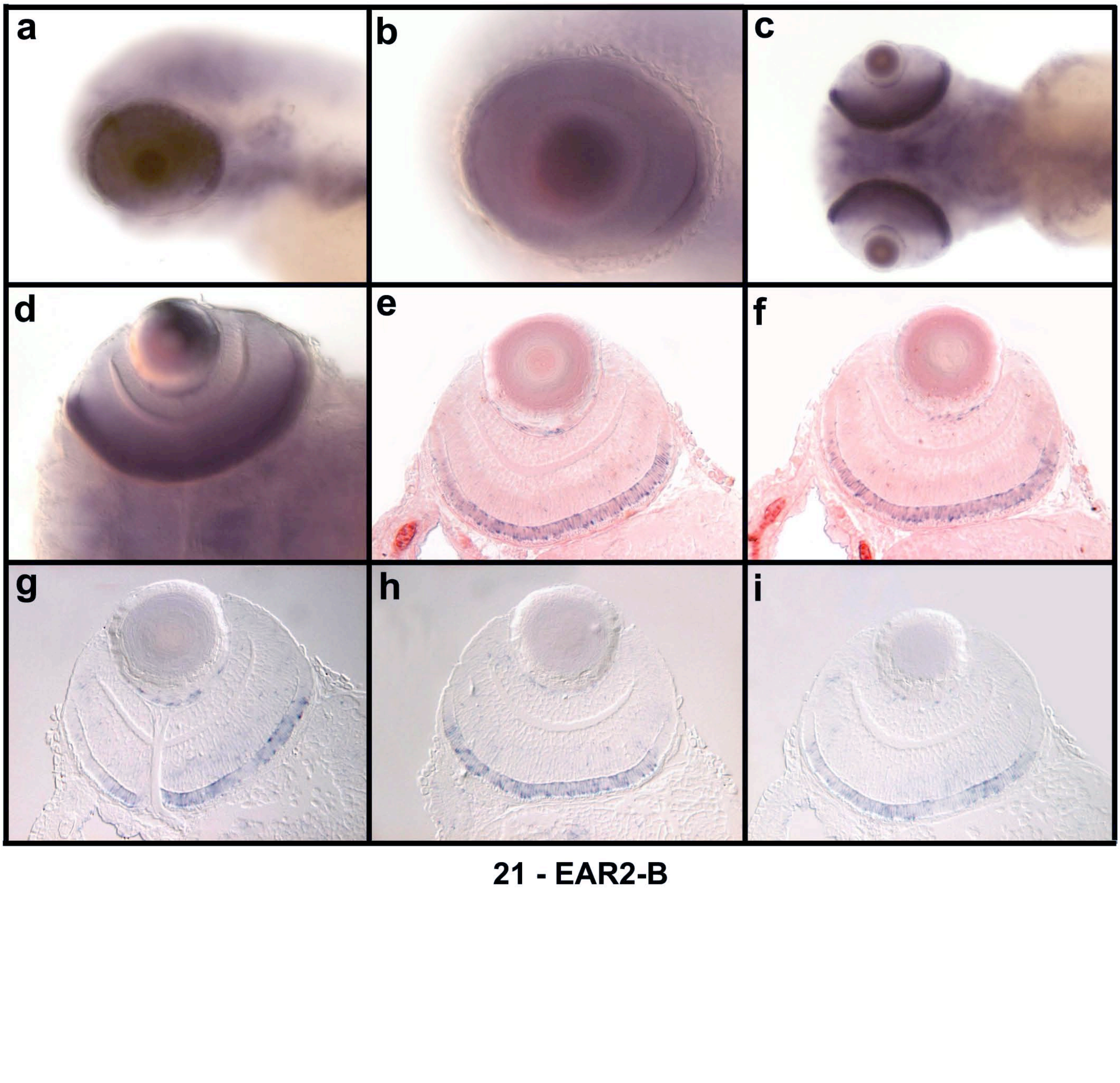

21 - EAR2-B

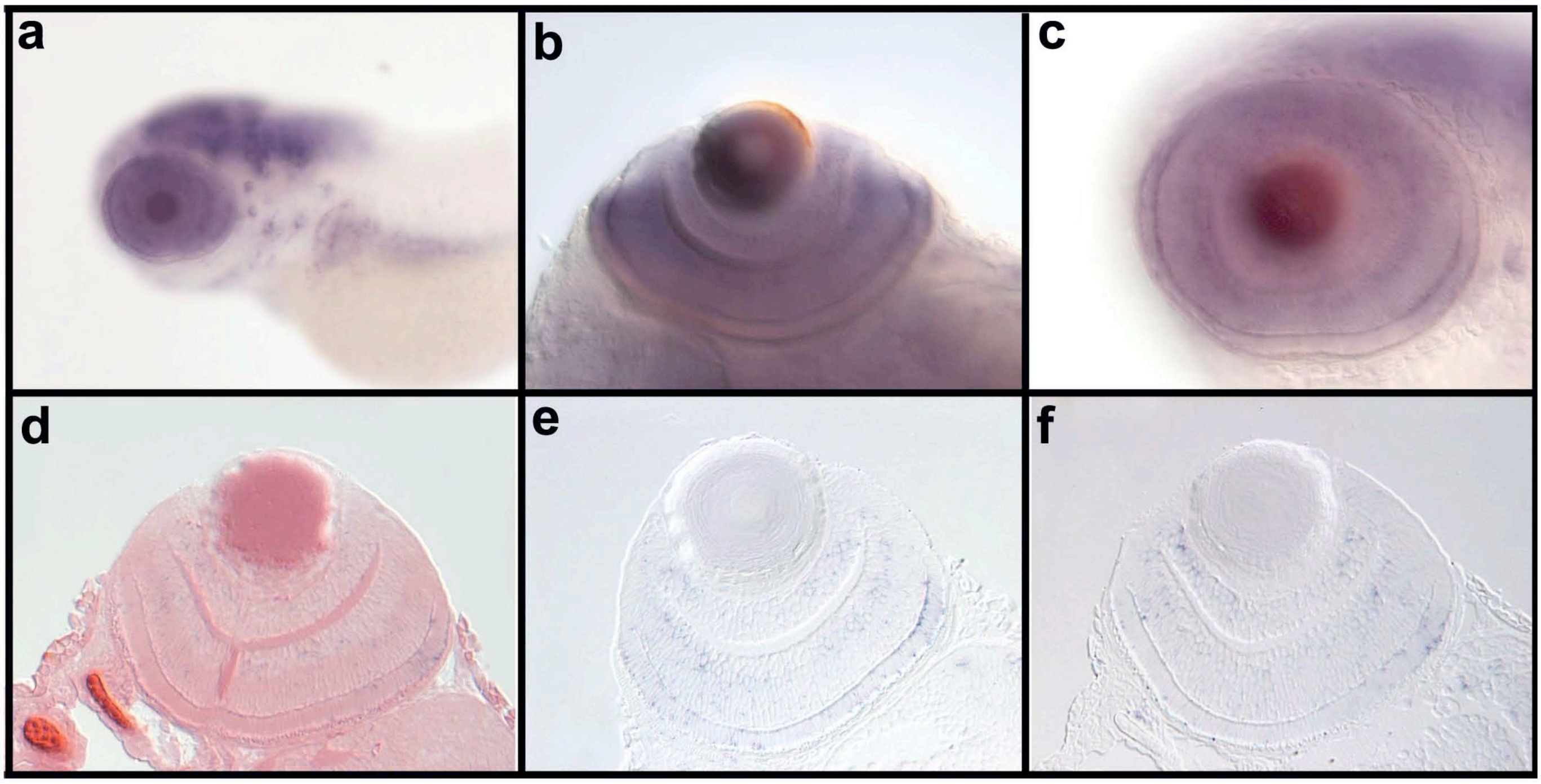

22 - ERRα

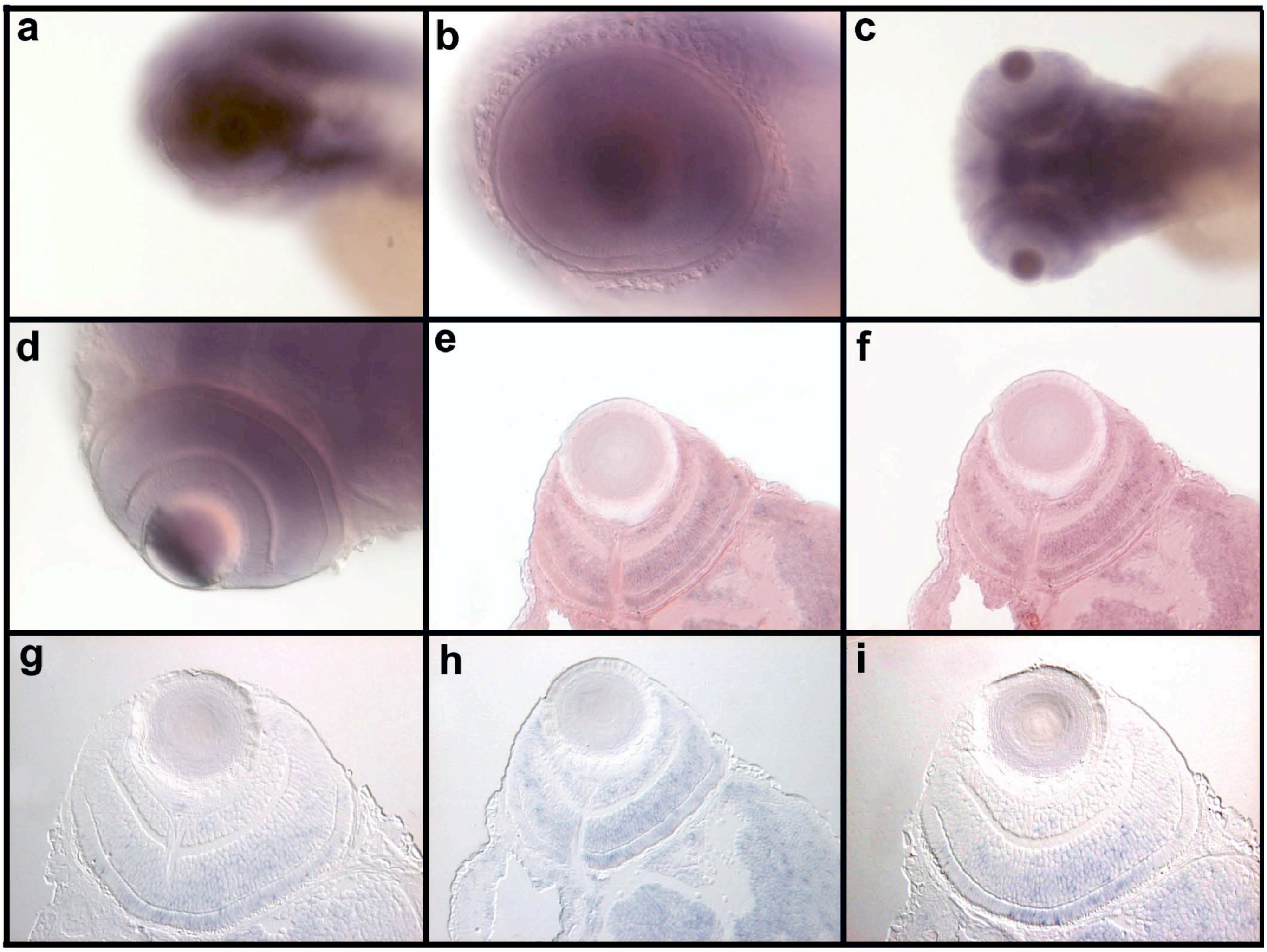

23 - ERRβγ

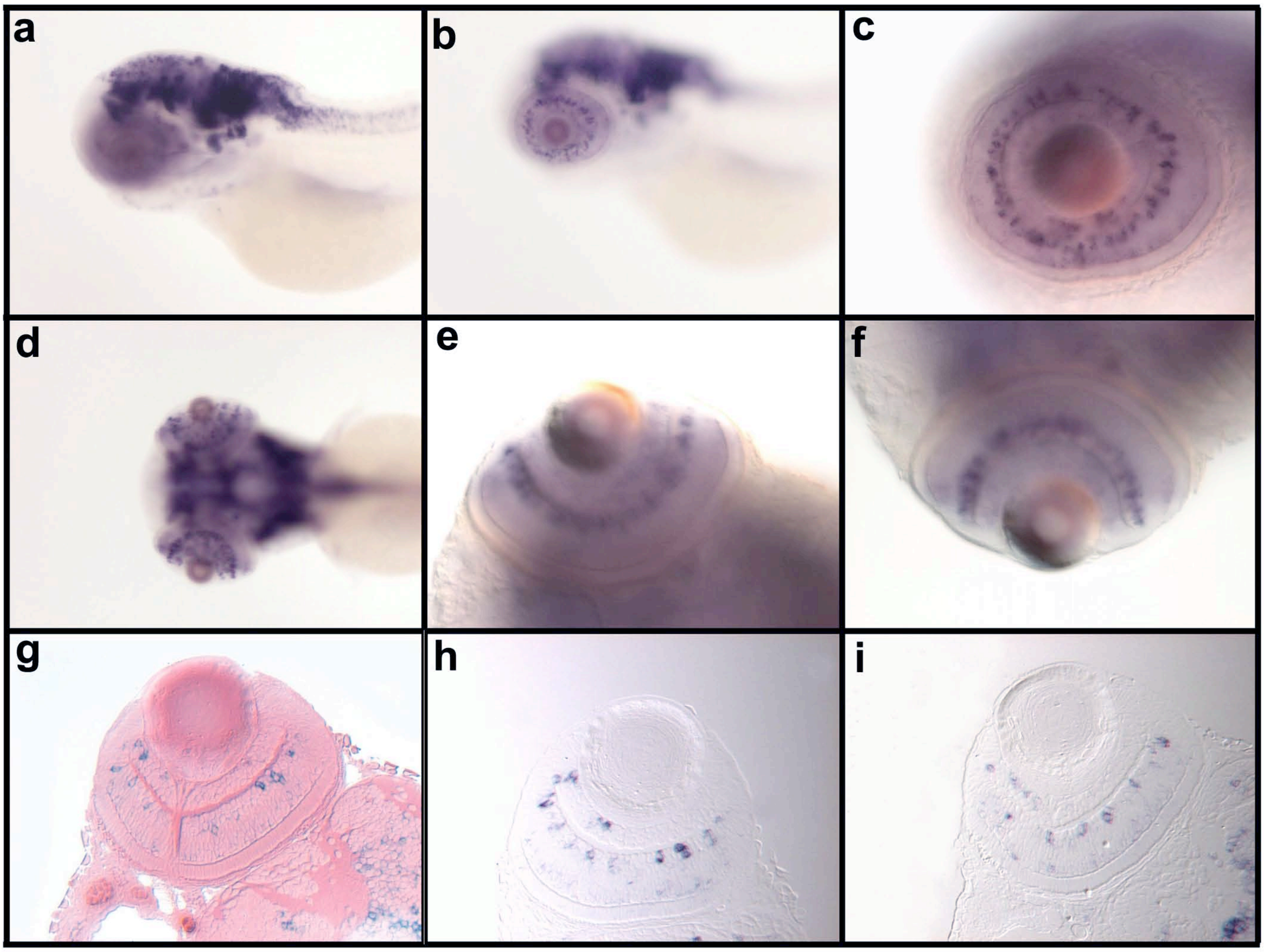

24 - ERRγ

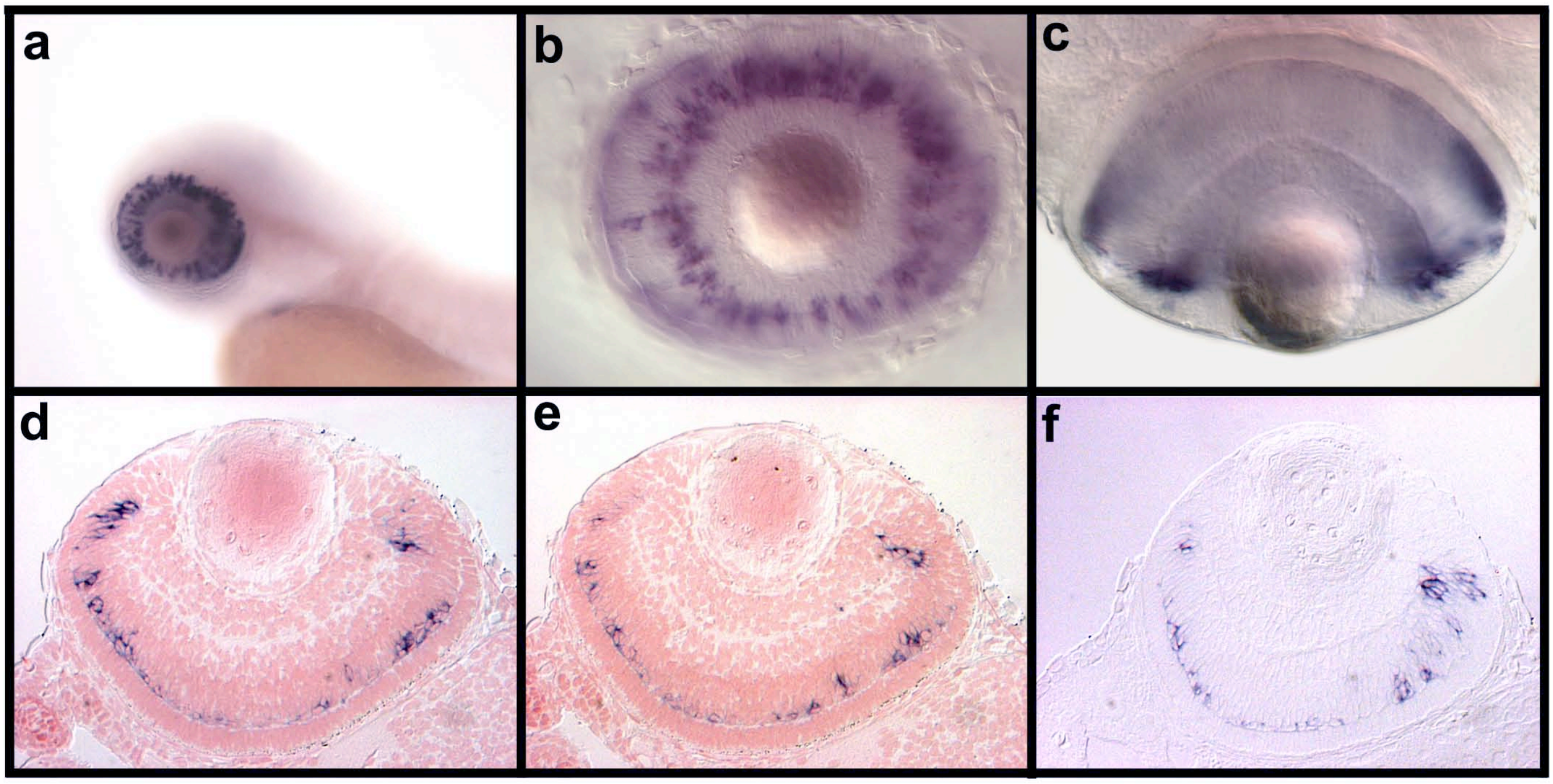

25 - ERRδ

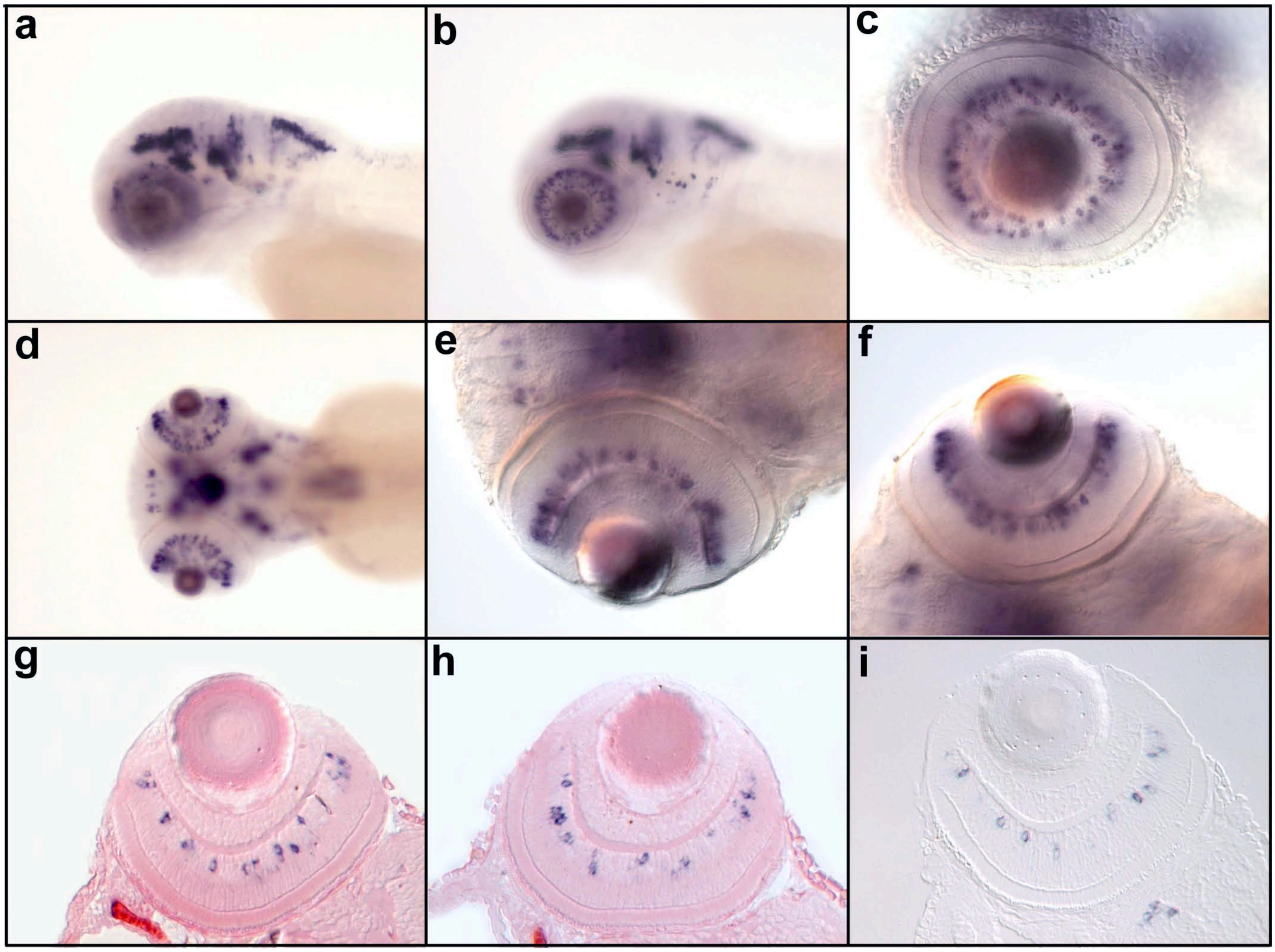

26 - NURRI
