# Supplementary material for: Unexpected Novel Relational Links Uncovered by Extensive Developmental Profiling of Nuclear Receptor Expression
Source: PLoS Genet. 2007 Nov 9;3(11):e188. doi: 10.1371/journal.pgen.0030188 (PMC2065881; doi:10.1371/journal.pgen.0030188)
Supplement: Supplementary file 4 [file pgen.0030188.sg007.pdf]

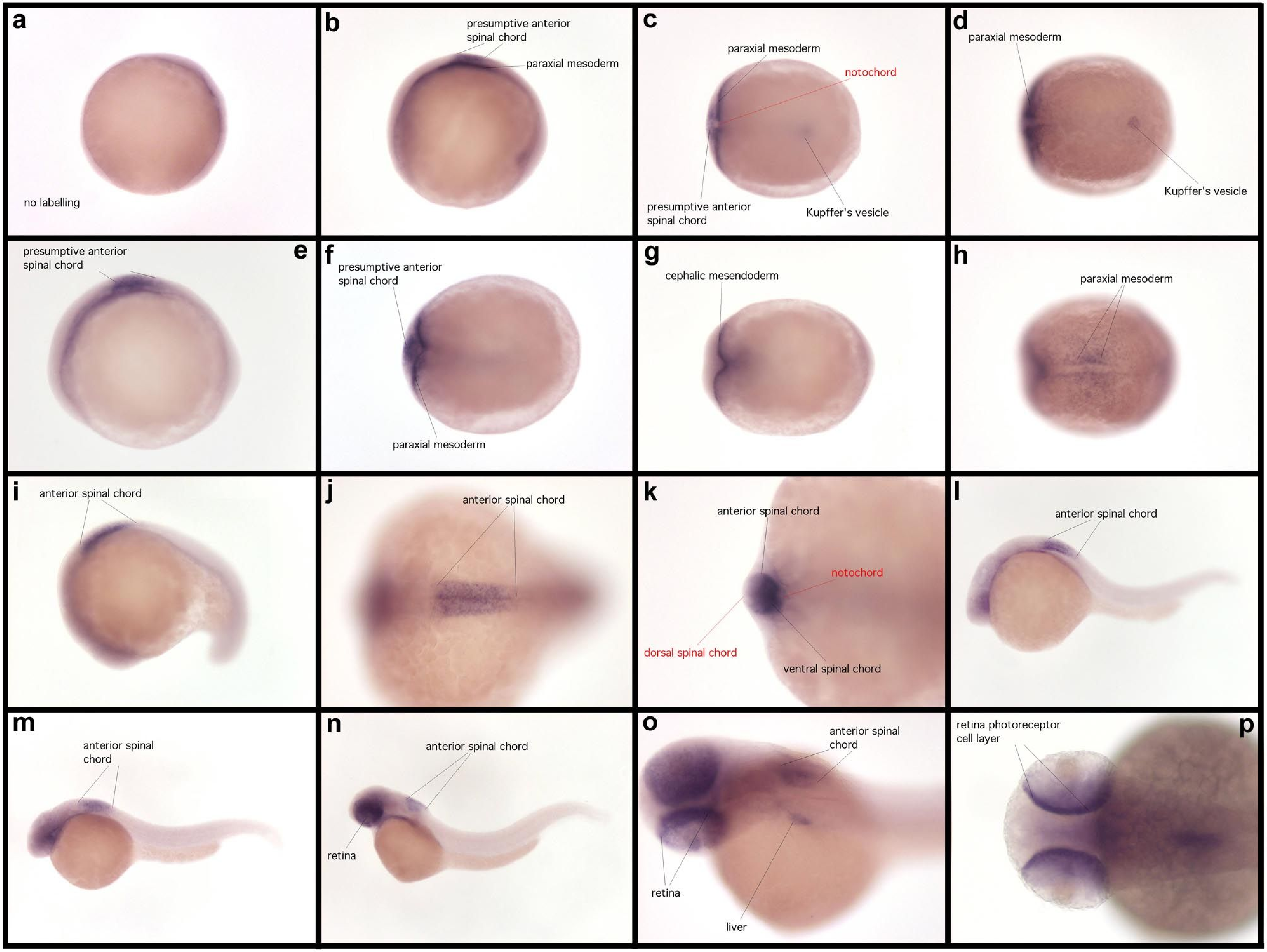
a
no labelling
b
presumptive anterior spinal chord
paraxial mesoderm
c
paraxial mesoderm
notochord
presumptive anterior spinal chord
Kupffer's vesicle
d
paraxial mesoderm
Kupffer's vesicle
e
presumptive anterior spinal chord
f
presumptive anterior spinal chord
paraxial mesoderm
g
cephalic mesendoderm
h
paraxial mesoderm
i
anterior spinal chord
j
anterior spinal chord
k
anterior spinal chord
notochord
dorsal spinal chord
ventral spinal chord
l
anterior spinal chord
m
anterior spinal chord
n
anterior spinal chord
retina
o
anterior spinal chord
retina
liver
p
retina photoreceptor cell layer

RXR γ B
