# Supplementary material for: Unexpected Novel Relational Links Uncovered by Extensive Developmental Profiling of Nuclear Receptor Expression
Source: PLoS Genet. 2007 Nov 9;3(11):e188. doi: 10.1371/journal.pgen.0030188 (PMC2065881; doi:10.1371/journal.pgen.0030188)
Supplement: Supplementary file 5 [file pgen.0030188.st001.pdf]

NOMENCLATURE	NAME	SYNONYMOUS	GENBANK N°	Probe Start-End
NR1A1-A	TRalpha-A	c-erbA	NM_131396	602-1516
NR1A1-B	TRalpha-B	c-erbA	DQ017632	1-951
NR1A2	TRbeta		NM_131340	572-1379
NR1B1-A	RARalpha-A		S74155	210-1544
NR1B1-B	RARalpha-B		L03399	188-1561
NR1B3-A	RARgamma-A		NM_131339	262-1761
NR1B3-B	RARgamma-B		DQ017621	1-630
NR1C1-A	PPARalpha-A		DQ017612	1-1419
NR1C1-B	PPARalpha-B		DQ017618	1-492
NR1C2-A	PPARbeta-A	NUC1, PPARdelta, FAAR	AF342937	1-990
NR1C2-B	PPARbeta-B	NUC1, PPARdelta, FAAR	AF342938	1-1107
NR1C3	PPARgamma	ARF6	DQ017619	1-925
NR1D1	Reverbalpha	ear1	AY391444	532-1894
NR1D2-A	Reverbbeta-A	BD73, RVR, HZF-2	AF342941	1-1211
NR1D2-B	Reverbbeta-B	BD73, RVR, HZF-2	AF342942	1-1926
NR1D4-A	Reverbgamma-A		DQ017622	1-1389
NR1D4-A	Reverbgamma-B		DQ017623	1-1060
NR1F1-A	RORalpha-A	RZRalpha	NM_201067	325-1059
NR1F1-B	RORalpha-B	RZRalpha	DQ017624	1-686
NR1F2	RORbeta	RZRbeta	DQ017626	1-873
NR1F3-A	RORgamma-A	TOR, ROCC	DQ017627	1-1125
NR1F3-B	RORgamma-B	TOR, ROCC	DQ017628	1-1443
NR1H3	LXRalpha	RLD-1	BC092160	780-1340
NR1H4	FXRalpha	RIP14, BAR, HRR-1	BC076220	757-1096
NR1H5	FXRbeta		DQ017614	1-877
NR1I1-A	VDR-A		AF164512	1-1362
NR1I1-B	VDR-B		DQ017633	1-1260
NR1I2	PXR	SXR, PAR, ONR1, BXR	AF502918	186-986
NR2A1	HNF4alpha	HNF4	AF473824	299-1014
NR2A3	HNF4beta		BC065657	1-1870
NR2A2	HNF4gamma		DQ017616	1-1239
NR2B1-A	RXRalpha-A	previously named RXRgamma	NM_131153	607-1165
NR2B1-B	RXRalpha-B		DQ017629	1-1098
NR2B2-A	RXRbeta-A	H-2RIIBP, RCoR-1, RXRdelta	U29942	388-1704
NR2B2-B	RXRbeta-B	H-2RIIBP, RCoR-1, RXRepsilon	U29941	97-1365
NR2B3	RXRgamma	previously named RXRalpha	U29940	20-1345
NR2C1	TR2	TR2-11, DOR2, DOR1	DQ017631	1-1740
NR2C2	TR4	TAK, TR2R1	DQ017625	1-941
NR2D1	TLL	TLX	BC077146	1402-1726
NR2D2	PNR	RNR	BC085568	1089-1607

NR2F1-A	COUPTFalpha-A	COUPTFI, EAR3, SVP44	BC066671	43-1278
NR2F1-B	COUPTFalpha-B		BC056748	290-1459
NR2F2	COUPTFbeta	COUPTFII, ARP-1, SVP40	NM_131183	340-1608
NR2F5	COUTFgamma	COUPTFIII, COUPTFIV, SVP46	NM_131186	650-1851
NR2F6-A	EAR2-A		BC065330	611-1217
NR2F6-B	EAR2-B		BC065332	404-1045
NR3A1	ERalpha		NM_152959	188-1897
NR3A2-A	ERbeta-A		AJ414566	80-1858
NR3A2-B	ERbeta-B		NM_180966	139-1800
NR3B1	ERRalpha	ERR1, ESRL1	AY556395	1-1311
NR3B2	ERRbeta	ERR2, Estrrb	AY556396	1-1023
NR3B3	ERRgamma	ERR3, ESRRG	AY556397	1-1308
NR3B4	ERRdelta		AY556398	1-1035
NR3B2/3	ERRbeta/gamma		AY556399	1-727
NR3C1	GR		DQ017615	1-940
NR3C2	MR		DQ017617	1-1059
NR3C3	PR		DQ017620	1-1122
NR3C4	AR		AY510448	159-897
NR4A1	NGFIB	NAK1, ST-59, TR3, nur77, N10, TIS1, NGFIBalpha	NM_001002173	912-1309
NR4A2	NURR1	NOT, TINUR, HZF-3, NGFIBbeta	BC076176	248-1889
NR4A3	NOR1	TEC, MINOR, CHN, NGFIBgamma	DQ017611	1-1785
NR5A1-A	SF1-A	FF1B, FTZ-F1, ELP, AD4BP	AF198086	112-1476
NR5A1-B	SF1-B	FF1D, FTZ-F1, ELP, AD4BP	AY212920	160-1668
NR5A2	LRH1	FF1aE, zFF1, FTF, B1F, FTZ-F1beta, FZR1	AY034061	1-1554
NR5A5	ff1C		AF327373	1-1614
NR6A1-A	GCNF-A	RTR, NCNF	AJ007703	391-1391
NR6A1-B	GCNF-B	RTR, NCNF	DQ017610	1-1257
NR0B1	DAX	AHCH	DQ017613	1-365
NR0B2-A	SHP-A		BC058069	101_691
NR0B2-B	SHP-B		DQ017630	1-705
COACTIVATOR	BRG1	SNF2b, SMARCA4	AY205256	1215-1639
COACTIVATOR	BAF53a	2810432C06Rik, Actl6a, Baf53a	BC045889	521-909
COACTIVATOR	BAF60a	SMARCD1, D15Kz1	NM_198358	196-706
COACTIVATOR	PCAF		DQ017634	1-570
COACTIVATOR	P300-A		DQ017643	1-560
COACTIVATOR	P300-B		DQ017642	1-561
COACTIVATOR	CBP-A	Crebbp	DQ017638	1-584
COACTIVATOR	CBP-B	Crebbp	DQ017641	1-716
COACTIVATOR	CITED3		NM_200078	174-941
COACTIVATOR	SRC1	ERAP160, NCoA1	DQ017644	1-531
COACTIVATOR	SRC2	TIF2, NCoA2, GRIP1	NM_131777	313-882

COACTIVATOR	SRC3	ACTR, CIP, RAC3, AIB1, TRAM1	DQ017645	1-575
COACTIVATOR	CARM1	PRMT4	NM_001003645	58-606
COACTIVATOR	NCOA6	AIB3, ASC-2, ASC2, NRC, PRIP, RAP250	DQ017635	1-708
COACTIVATOR	RIP140-A	ERAP140, Nrip1	DQ017640	1-741
COACTIVATOR	RIP140-B	ERAP140, Nrip1	DQ017639	1-716
COACTIVATOR	TRAP220	DRIP205, DRIP230, TRIP2, Pparbp, MED1, CRSP210	BC095630	1272-1725
COACTIVATOR	TRIP13		NM_200582	14-526
COACTIVATOR	ARA54	RFP14, Rnf14, TRIAD2	NM_001002087	1-458
COACTIVATOR	MYST-HAT2	MYST2, Hbo1, Hboa	BC075772	1-512
COACTIVATOR	TIF1ALPHA	Trim24, Tif1a	AY598454	1-3116
COACTIVATOR	TIF1GAMMA	Trim33, Tif1g	AY598453	1-3957
COACTIVATOR	NCOA4	ARA70, RFG, ELE1a, ELE1b	BC065638	104-1834
COACTIVATOR	PGC1	Pgc-1alpha, Pgc-1alphaa, Pgco1	DQ017637	1-688
COACTIVATOR	TRIP7	Hmgn3, HMGN3a, HMGN3b	BC085665	109-405
COREPRESSOR	HDAC1	HD1, RPD3	BC085375	15-569
COREPRESSOR	HDAC3		BC044543	413-637
COREPRESSOR	HDAC4		DQ017636	1-433
COREPRESSOR	SMRT	NcoR2, TRAC2, RFG7	XM_684906	2552-3074
COREPRESSOR	NCOR	NcoR1, RIP13, Rxrip13	BC049302	312-1220
COREPRESSOR	Alien	TRIP15	NM_001002055	51-941
