# Supplementary material for: Unexpected Novel Relational Links Uncovered by Extensive Developmental Profiling of Nuclear Receptor Expression
Source: PLoS Genet. 2007 Nov 9;3(11):e188. doi: 10.1371/journal.pgen.0030188 (PMC2065881; doi:10.1371/journal.pgen.0030188)
Supplement: Supplementary file 6 [file pgen.0030188.st002.pdf]

Bertrand et al., Supplementary Table 2					TRalpha-A	TRalpha-B	TRbeta	RARalpha-A	RARalpha-B	RARgamma-A	RARgamma-B	PPARalpha-A	PPARalpha-B	PPARbeta-A
Gastrula					0	1	0	1	1	1	0	0	0	0
	forerunner cells				0	1	0	0	0	0	0	0	0	0
	margin				0	1	0	1	1	1	0	0	0	0
	axis				0	1	0	0	0	0	0	0	0	0
		prechordal plate			0	1	0	0	0	0	0	0	0	0
	paraxial mesoderm	segmental plate			0	1	0	0	1	0	0	0	0	0
	neural plate				0	1	0	1	1	0	0	0	0	0
	epidermis				0	1	0	0	0	1	0	0	0	0
	YSL				0	0	0	0	0	0	0	0	0	0
	NR				0	1	0	0	0	0	0	0	0	0
ES					0	1	0	1	1	1	1	0	0	0
	polster				0	1	0	0	0	0	0	0	0	0
	notochord				0	1	0	0	0	0	0	0	0	0
	tailbud				0	1	0	1	1	1	1	0	0	0
	ventral mesoderm				0	1	0	1	1	0	0	0	0	0
	cephalic mesoderm				0	1	0	0	0	0	0	0	0	0
	segmental plate				0	1	0	1	1	1	0	0	0	0
		somites			0	1	0	0	0	0	0	0	0	0
		adaxial cells			0	1	0	0	0	0	0	0	0	0
	neural plate				0	1	0	1	1	1	0	0	0	0
		presumptive spinal chord			0	1	0	1	1	1	0	0	0	0
		brain			0	1	0	1	1	0	0	0	0	0
			forebrain		0	1	0	0	1	0	0	0	0	0
				telencephalon	0	1	0	0	0	0	0	0	0	0
				diencephalon	0	1	0	0	1	0	0	0	0	0
			midbrain		0	1	0	0	0	0	0	0	0	0
			hindbrain		0	1	0	1	1	1	0	0	0	0
	eye field				0	1	0	0	0	0	0	0	0	0
	floor plate				0	1	0	0	0	0	0	0	0	0
	neural crest				0	1	0	0	0	1	0	0	0	0
	epidermis				0	1	0	0	0	1	0	0	0	0
	Kupffer's vesicle				0	1	0	0	0	0	0	0	0	0
	YSL				0	0	0	0	0	0	0	0	0	0
	NR				0	1	0	0	0	0	0	0	0	0
MS					0	1	0	1	1	1	1	0	0	0
	notochord				0	1	0	0	0	0	0	0	0	0
	hematopoietic mesoderm				0	1	0	0	0	0	0	0	0	0
	paraxial mesoderm				0	1	0	1	1	0	0	0	0	0
		unsegmented			0	1	0	1	1	0	0	0	0	0
		somites			0	1	0	0	1	0	0	0	0	0
		adaxial cells			0	1	0	0	0	0	0	0	0	0
	ventral mesenchyme				0	1	0	1	1	0	0	0	0	0
	retina/eye				0	1	0	0	0	0	0	0	0	0
	brain				0	1	0	1	1	1	0	0	0	0
		forebrain			0	1	0	0	1	0	0	0	0	0
			diencephalon		0	1	0	0	1	0	0	0	0	0
		midbrain			0	1	0	0	0	0	0	0	0	0
		hindbrain			0	1	0	1	1	1	0	0	0	0
		cranial ganglia			0	1	0	0	0	0	0	0	0	0
	spinal chord				0	1	0	1	1	1	0	0	0	0
		anterior spinal chord			0	1	0	1	1	1	0	0	0	0
		posterior spinal chord			0	1	0	1	1	1	0	0	0	0
	epiphysis				0	1	0	0	0	0	0	0	0	0
	tailbud				0	1	0	1	1	1	1	0	0	0
	neural crest				0	1	0	0	0	1	0	0	0	0
	branchial arches				0	1	0	0	0	1	1	0	0	0
	pronephric ducts				0	1	0	0	0	0	0	0	0	0
	endoderm	intestine			0	1	0	0	0	0	0	0	0	0
	epidermis				0	1	0	0	0	1	0	0	0	0
	mucous cells				0	1	0	0	0	0	0	0	0	0
	hatching gland				0	1	0	0	0	0	0	0	0	0
	YSL				0	0	0	0	0	0	0	0	0	0
	NR				0	1	0	0	0	0	0	0	0	0
24 hpf					1	1	1	1	1	1	1	0	0	1
	nose				0	1	0	0	0	0	0	0	0	1
	otic vesicle				0	1	0	0	0	0	0	0	0	1
	lateral line				0	1	0	0	0	0	0	0	0	1
	head mesenchyme				0	1	0	0	0	1	0	0	0	1

	epiphysis				0	1	0	0	0	0	0	0	0	1
	adenohypophysis				0	1	0	0	0	0	0	0	0	1
	brain				1	1	0	1	1	1	0	0	0	1
		forebrain			0	1	0	0	1	0	0	0	0	1
			telencephalon		0	1	0	0	0	0	0	0	0	1
			diencephalon		0	1	0	0	1	0	0	0	0	1
		midbrain			1	1	0	0	0	0	0	0	0	1
			tegmentum		0	1	0	0	0	0	0	0	0	1
			tectum		1	1	0	0	0	0	0	0	0	1
		MHB			1	1	0	0	0	0	0	0	0	1
		hindbrain			0	1	0	1	1	1	0	0	0	1
	cranial ganglia				0	1	0	0	0	0	0	0	0	1
	spinal chord				0	1	0	1	1	1	1	0	0	1
		anterior spinal chord			0	1	0	1	1	1	0	0	0	1
	branchial arches				0	1	0	1	1	1	1	0	0	1
	retina				1	1	1	1	1	0	0	0	0	1
	lens				1	1	0	0	0	0	0	0	0	1
	tailbud				0	1	0	0	0	1	1	0	0	0
	somites				0	1	0	0	0	0	0	0	0	0
	slow muscle fibers				0	1	0	0	0	0	0	0	0	0
	mucous cells				0	1	0	0	0	0	0	0	0	0
	pronephric ducts				0	1	0	0	0	0	0	0	0	0
	interenal tissue				0	1	0	0	0	0	0	0	0	0
	intestinal bulb				0	1	0	0	0	0	0	0	0	0
	intestine				0	1	0	0	0	0	0	0	0	0
	pancrea primordium				0	1	0	0	0	0	0	0	0	0
	pharyngeal pouches				0	1	0	0	0	0	0	0	0	1
	notochord				0	1	0	0	0	0	0	0	0	0
	epidermis				1	1	0	0	0	0	0	0	0	1
	hatching gland				0	1	0	0	0	0	0	0	0	0
	YSL				0	0	0	0	0	0	0	0	0	0
	NR				1	1	0	0	0	0	0	0	0	1
36 hpf					1	1	0	1	1	1	1	0	0	1
	nose				1	1	0	0	0	0	0	0	0	1
	otic vesicle				1	1	0	0	0	0	0	0	0	1
	retina				1	1	1	1	1	0	0	0	0	1
		proliferative zone			1	1	0	0	1	0	0	0	0	1
		non proliferative zone			1	1	1	0	1	0	0	0	0	1
	lens				1	1	0	0	0	0	0	0	0	1
	lateral line				0	1	0	0	0	0	0	0	0	0
	brain				1	1	0	1	1	1	0	0	0	1
		forebrain			1	1	0	0	1	0	0	0	0	1
			telencephalon		1	1	0	0	0	0	0	0	0	1
			diencephalon		1	1	0	0	1	0	0	0	0	1
				habenula (diencephalon)	1	1	0	0	0	0	0	0	0	1
		midbrain			1	1	0	0	1	1	0	0	0	1
			tegmentum		1	1	0	0	0	0	0	0	0	1
			tectum		1	1	0	0	1	1	0	0	0	1
		MHB			1	1	0	0	0	0	0	0	0	1
		hindbrain			0	1	0	1	1	1	0	0	0	1
	spinal chord				0	1	0	1	1	1	0	0	0	1
		anterior spinal chord			0	1	0	1	1	1	0	0	0	1
	cranial ganglia				0	1	0	0	0	0	0	0	0	0
	epiphysis				1	1	0	0	0	0	0	0	0	1
	hypophysis				1	1	0	0	0	0	0	0	0	1
	head mesenchyme				1	1	0	0	0	1	0	0	0	1
	branchial arches				0	1	0	1	1	1	1	0	0	1
	pectoral fin bud				0	1	0	0	1	0	1	0	0	1
	heart				0	1	0	0	0	0	0	0	0	1
	somites				0	1	0	0	0	0	0	0	0	0
	slow muscle fibers				0	1	0	0	0	0	0	0	0	0
	pronephric ducts				0	1	0	0	0	0	0	0	0	0
	interrenal tissue				0	1	0	0	0	0	0	0	0	0
	mucous cells				0	1	0	0	0	0	0	0	0	0
	pancreas primordium				0	1	0	0	0	0	0	0	0	0
	intestine				0	1	0	0	0	0	0	0	0	0
	intestinal bulb				0	1	0	0	0	0	0	0	0	0
	liver				0	1	0	0	0	0	0	0	0	0
	proctodeum				0	1	0	0	0	0	0	0	0	0

	pharyngeal pouches				0	1	0	0	0	0	0	0	0	1
	epidermis				0	1	0	0	0	0	0	0	0	1
	hatching gland				0	1	0	0	0	0	0	0	0	0
	NR				1	1	0	0	0	0	0	0	0	1
48 hpf					1	1	1	1	1	1	1	0	0	1
	nose				1	1	0	0	0	0	0	0	0	1
	brain				1	1	0	1	1	1	0	0	0	1
		forebrain			1	1	0	0	0	0	0	0	0	1
			telencephalon		1	1	0	0	0	0	0	0	0	1
			diencephalon		1	1	0	0	1	0	0	0	0	1
				habenula (diencephalon)	1	1	0	0	0	0	0	0	0	1
		midbrain			1	1	1	1	1	1	0	0	0	1
			tegmentum		1	1	1	0	0	0	0	0	0	1
			tectum		1	1	1	1	1	1	0	0	0	1
		hindbrain			1	1	1	1	1	1	0	0	0	1
		MHB			1	1	0	0	1	0	0	0	0	1
	spinal chord				0	1	0	1	1	1	0	0	0	0
		anterior spinal chord			0	1	0	1	1	1	0	0	0	0
	cranial ganglia				0	1	0	0	0	0	0	0	0	0
	otic vesicle				1	1	0	0	0	0	0	0	0	0
	hypophysis				1	1	0	0	0	0	0	0	0	1
	epiphysis				1	1	0	0	0	0	0	0	0	1
	branchial arches				0	1	0	1	1	1	1	0	0	1
	retina				1	1	1	1	1	0	0	0	0	1
		proliferative zone			1	1	0	1	0	0	0	0	0	1
		non proliferative zone			1	1	0	1	1	0	0	0	0	1
		inner nuclear layer			1	1	0	1	1	0	0	0	0	1
		ganglion cell layer			1	1	0	1	1	0	0	0	0	1
		photoreceptor cell layer			1	1	1	0	0	0	0	0	0	1
	lateral line neuromasts				0	1	0	0	0	0	0	0	0	0
	pectoral fin				0	1	0	0	1	1	1	0	0	1
	pronephric ducts				0	1	0	0	0	0	0	0	0	0
	interrenal tissue				0	1	0	0	0	0	0	0	0	0
	heart				0	1	0	0	0	0	0	0	0	0
	liver				0	1	0	0	0	0	0	0	0	1
	pancreas				0	1	0	0	0	0	0	0	0	0
	intestin				0	1	0	0	0	0	0	0	0	0
	intestinal bulb				0	1	0	0	0	0	0	0	0	0
	pharyngeal endoderm				0	1	0	0	0	0	0	0	0	0
	epidermis				0	1	0	0	0	0	0	0	0	0
	NR				0	1	0	0	0	0	0	0	0	1

PPARbeta-B	PPARgamma	REVERBalpha	REVERBbeta-A	REVERBbeta-B	REVERBgamma-A	REVERBgamma-B	RORalpha-A	RORalpha-B	RORbeta	RORgamma-A	RORgamma-B	LXRalpha	FXRalpha	FXRbeta	VDR-A	VDR-B
1	1	0	0	0	1	0	0	0	0	0	0	0	0	0	1	0
1	1	0	0	0	1	0	0	0	0	0	0	0	0	0	1	0
1	1	0	0	0	1	0	0	0	0	0	0	0	0	0	1	0
1	1	0	0	0	1	0	0	0	0	0	0	0	0	0	1	0
1	1	0	0	0	1	0	0	0	0	0	0	0	0	0	1	0
1	1	0	0	0	1	0	0	0	0	0	0	0	0	0	1	0
1	1	0	0	0	1	0	0	0	0	0	0	0	0	0	1	0
1	1	0	0	0	1	0	0	0	0	0	0	0	0	0	1	0
0	0	0	0	0	0	0	0	0	0	0	0	0	0	0	0	0
1	1	0	0	0	1	0	0	0	0	0	0	0	0	0	1	0
1	1	0	0	0	1	0	0	0	0	1	0	0	0	0	1	0
1	1	0	0	0	1	0	0	0	0	0	0	0	0	0	1	0
1	1	0	0	0	1	0	0	0	0	0	0	0	0	0	1	0
1	1	0	0	0	1	0	0	0	0	0	0	0	0	0	1	0
1	1	0	0	0	1	0	0	0	0	0	0	0	0	0	1	0
1	1	0	0	0	1	0	0	0	0	0	0	0	0	0	1	0
1	1	0	0	0	1	0	0	0	0	0	0	0	0	0	1	0
1	1	0	0	0	1	0	0	0	0	0	0	0	0	0	1	0
1	1	0	0	0	1	0	0	0	0	0	0	0	0	0	1	0
1	1	0	0	0	1	0	0	0	0	1	0	0	0	0	1	0
1	1	0	0	0	1	0	0	0	0	0	0	0	0	0	1	0
1	1	0	0	0	1	0	0	0	0	1	0	0	0	0	1	0
1	1	0	0	0	1	0	0	0	0	1	0	0	0	0	1	0
1	1	0	0	0	1	0	0	0	0	0	0	0	0	0	1	0
1	1	0	0	0	1	0	0	0	0	1	0	0	0	0	1	0
1	1	0	0	0	1	0	0	0	0	0	0	0	0	0	1	0
1	1	0	0	0	1	0	0	0	0	0	0	0	0	0	1	0
1	1	0	0	0	1	0	0	0	0	0	0	0	0	0	1	0
1	1	0	0	0	1	0	0	0	0	0	0	0	0	0	1	0
1	1	0	0	0	1	0	0	0	0	0	0	0	0	0	1	0
1	1	0	0	0	1	0	0	0	0	0	0	0	0	0	1	0
1	1	0	0	0	1	0	0	0	0	0	0	0	0	0	1	0
0	0	0	0	0	0	0	0	0	0	0	0	0	0	0	0	0
1	1	0	0	0	1	0	0	0	0	0	0	0	0	0	1	0
1	1	0	0	0	1	0	1	0	1	1	1	0	0	0	1	1
1	1	0	0	0	1	0	0	0	0	0	0	0	0	0	1	0
1	1	0	0	0	1	0	0	0	0	0	0	0	0	0	1	0
1	1	0	0	0	1	0	0	0	0	0	0	0	0	0	1	0
1	1	0	0	0	1	0	0	0	0	0	0	0	0	0	1	0
1	1	0	0	0	1	0	0	0	0	0	0	0	0	0	1	0
1	1	0	0	0	1	0	0	0	0	0	0	0	0	0	1	0
1	1	0	0	0	1	0	0	0	0	0	0	0	0	0	1	0
1	1	0	0	0	1	0	1	0	1	0	1	0	0	0	1	0
1	1	0	0	0	1	0	1	0	0	1	1	0	0	0	1	1
1	1	0	0	0	1	0	0	0	0	1	0	0	0	0	1	1
1	1	0	0	0	1	0	0	0	0	1	0	0	0	0	1	1
1	1	0	0	0	1	0	1	0	0	0	1	0	0	0	1	1
1	1	0	0	0	1	0	0	0	0	0	0	0	0	0	1	1
1	1	0	0	0	1	0	0	0	0	0	0	0	0	0	1	0
1	1	0	0	0	1	0	0	0	0	0	1	0	0	0	1	1
1	1	0	0	0	1	0	0	0	0	0	1	0	0	0	1	1
1	1	0	0	0	1	0	0	0	0	0	0	0	0	0	1	1
1	1	0	0	0	1	0	0	0	0	1	0	0	0	0	1	0
1	1	0	0	0	1	0	0	0	0	0	0	0	0	0	1	0
1	1	0	0	0	1	0	0	0	0	0	0	0	0	0	1	0
1	1	0	0	0	1	0	0	0	0	0	0	0	0	0	1	0
1	1	0	0	0	1	0	0	0	0	0	0	0	0	0	1	1
1	1	0	0	0	1	0	0	0	0	0	0	0	0	0	1	1
1	1	0	0	0	1	0	0	0	0	0	0	0	0	0	1	0
1	1	0	0	0	1	0	0	0	0	0	0	0	0	0	1	0
1	1	0	0	0	1	0	0	0	0	0	0	0	0	0	1	0
0	0	0	0	0	0	0	0	0	0	0	0	0	0	0	0	0
1	1	0	0	0	1	0	0	0	0	0	0	0	0	0	1	0
1	1	1	0	1	1	0	1	0	1	1	1	0	0	1	1	1
1	1	0	0	0	1	0	0	0	0	0	0	0	0	0	1	0
1	1	0	0	0	1	0	0	0	0	0	0	0	0	0	1	0
1	1	0	0	0	1	0	0	0	1	0	0	0	0	0	1	0
1	1	0	0	0	1	0	0	0	0	0	0	0	0	0	1	0

1	1	1	0	1	1	0	0	0	0	1	1	0	0	0	1	0
1	1	0	0	0	1	0	0	0	0	0	0	0	0	0	1	0
1	1	0	0	0	1	0	1	0	1	1	1	0	0	0	1	1
1	1	0	0	1	1	0	0	0	0	1	0	0	0	0	1	1
1	1	0	0	1	1	0	0	0	0	0	0	0	0	0	1	1
1	1	0	0	0	1	0	0	0	0	1	0	0	0	0	1	1
1	1	0	0	0	1	0	1	0	1	0	1	0	0	0	1	1
1	1	0	0	0	1	0	0	0	0	0	0	0	0	0	1	1
1	1	0	0	0	1	0	1	0	1	0	1	0	0	0	1	1
1	1	0	0	0	1	0	0	0	0	0	0	0	0	0	1	1
1	1	0	0	0	1	0	0	0	0	0	0	0	0	0	1	1
1	1	0	0	0	1	0	0	0	0	0	0	0	0	0	1	0
1	1	0	0	0	1	0	0	0	0	0	0	0	0	0	1	1
1	1	0	0	0	1	0	0	0	0	0	0	0	0	0	1	1
1	1	0	0	0	1	0	0	0	0	0	0	0	0	0	1	0
1	1	0	0	1	1	0	1	0	1	0	1	0	0	0	1	0
1	1	0	0	0	1	0	0	0	0	0	0	0	0	0	1	0
0	0	0	0	0	1	0	0	0	0	0	0	0	0	0	1	0
0	0	0	0	0	1	0	0	0	0	0	0	0	0	0	1	0
0	0	0	0	0	1	0	0	0	0	0	0	0	0	0	1	0
0	0	0	0	0	1	0	0	0	0	0	0	0	0	0	1	0
0	0	0	0	0	1	0	0	0	0	0	0	0	0	0	1	1
0	0	0	0	0	1	0	0	0	0	0	0	0	0	0	1	0
0	0	0	0	0	1	0	0	0	0	0	0	0	0	0	1	1
0	0	0	0	0	1	0	0	0	0	0	0	0	0	0	1	1
0	0	0	0	0	1	0	0	0	0	0	0	0	0	0	1	0
0	0	0	0	0	1	0	0	0	0	0	0	0	0	0	1	0
0	0	0	0	0	1	0	0	0	0	0	0	0	0	0	1	0
1	1	0	0	0	1	0	0	0	0	0	0	0	0	0	1	0
0	0	0	0	0	1	0	0	0	0	0	0	0	0	0	1	0
0	0	0	0	0	0	0	0	0	0	0	0	0	0	1	0	0
1	1	0	0	0	1	0	0	0	0	0	0	0	0	0	1	0
1	1	1	0	0	1	1	1	1	1	1	1	0	1	1	1	1
1	1	0	0	0	1	0	0	0	0	0	1	0	0	0	1	0
1	1	0	0	0	1	0	0	0	0	0	0	0	0	0	1	0
1	1	0	0	0	1	0	1	1	1	1	1	0	0	0	1	1
1	1	0	0	0	1	0	1	0	1	0	1	0	0	0	1	1
1	1	0	0	0	1	0	1	1	1	1	1	0	0	0	1	1
1	1	0	0	0	1	0	0	0	0	0	0	0	0	0	1	0
0	0	0	0	0	1	0	0	0	1	0	0	0	0	0	1	0
1	1	0	0	0	1	0	1	1	1	1	1	0	0	0	1	1
1	1	0	0	0	1	0	0	0	0	1	0	0	0	0	1	1
1	1	0	0	0	1	0	0	0	0	0	0	0	0	0	1	1
1	1	0	0	0	1	0	0	0	0	1	0	0	0	0	1	1
1	1	0	0	0	1	0	0	0	0	0	0	0	0	0	1	0
1	1	0	0	0	1	0	1	0	1	0	1	0	0	0	1	1
1	1	0	0	0	1	0	0	0	0	0	0	0	0	0	1	1
1	1	0	0	0	1	0	1	0	1	0	1	0	0	0	1	1
1	1	0	0	0	1	0	0	0	0	0	0	0	0	0	1	1
1	1	0	0	0	1	1	0	1	0	0	0	0	0	0	1	1
1	1	0	0	0	1	0	0	0	0	0	0	0	0	0	1	1
1	1	0	0	0	1	0	0	0	0	0	0	0	0	0	1	1
0	0	0	0	0	1	0	0	0	0	0	0	0	0	0	1	0
1	1	1	0	0	1	0	0	0	0	1	1	0	0	0	1	0
1	1	0	0	0	1	0	0	0	0	0	1	0	0	0	1	0
1	1	0	0	0	1	0	0	0	0	0	0	0	0	0	1	0
1	1	0	0	0	1	0	0	0	0	0	0	0	0	0	1	1
0	0	0	0	0	1	0	0	0	0	0	0	0	0	0	1	1
0	0	0	0	0	1	0	0	0	0	0	0	0	0	0	1	0
0	0	0	0	0	1	0	0	0	0	0	0	0	0	0	1	0
0	0	0	0	0	1	0	0	0	0	0	0	0	0	0	1	0
0	0	0	0	0	1	0	0	0	0	0	0	0	0	0	1	1
0	0	0	0	0	1	0	0	0	0	0	0	0	0	0	1	0
0	0	0	0	0	1	0	0	0	0	0	0	0	0	0	1	0
0	0	0	0	0	1	0	0	0	0	0	1	0	0	0	1	0
0	0	0	0	0	1	0	0	0	0	0	0	0	0	0	1	1
0	0	0	0	0	1	0	0	0	0	0	0	0	0	0	1	1
0	0	0	0	0	1	0	0	0	0	0	0	0	1	1	1	0
0	0	0	0	0	1	0	0	0	0	0	0	0	0	0	1	0

0	0	0	0	0	1	0	0	0	0	0	0	0	0	0	1	0
0	0	0	0	0	1	0	0	0	0	0	0	0	0	0	1	0
1	1	0	0	0	1	0	0	0	0	0	0	0	0	0	1	0
0	0	0	0	0	1	0	0	0	0	0	0	0	0	0	1	0
1	1	1	1	1	1	1	1	1	1	1	1	0	1	1	1	1
1	1	0	0	0	1	0	0	0	0	0	0	0	0	0	1	0
1	1	0	1	1	1	1	1	1	1	0	1	0	0	0	1	1
1	1	0	1	1	1	0	0	0	0	0	1	0	0	0	1	1
1	1	0	1	0	1	0	0	0	0	0	0	0	0	0	1	1
1	1	0	1	1	1	0	0	0	0	0	1	0	0	0	1	1
1	1	0	0	0	1	0	0	0	0	0	0	0	0	0	1	0
1	1	1	1	1	1	1	1	1	1	1	1	0	0	0	1	1
1	1	0	1	1	1	0	0	0	0	0	0	0	0	0	1	1
1	1	1	1	1	1	1	1	1	1	1	1	0	0	0	1	1
1	1	1	1	1	1	1	0	1	0	0	0	0	0	0	1	1
1	1	0	1	0	1	0	0	0	1	0	0	0	0	0	1	1
1	1	0	0	0	1	0	0	0	0	0	0	0	0	0	1	0
1	1	0	0	0	1	0	0	0	0	0	0	0	0	0	1	0
0	0	0	0	0	1	0	0	0	0	0	0	0	0	0	1	0
0	0	0	0	0	1	0	0	0	0	0	0	0	0	0	1	0
1	1	0	0	0	1	0	0	0	0	0	0	0	0	0	1	0
1	1	1	0	1	1	0	0	0	0	1	0	0	0	0	1	0
1	1	0	0	0	1	0	0	0	0	0	0	0	0	0	1	1
1	1	1	1	1	1	1	1	1	1	1	1	0	0	0	1	1
1	1	0	0	0	1	1	0	0	1	0	1	0	0	0	1	1
1	1	1	1	1	1	0	1	1	0	1	0	0	0	0	1	0
1	1	1	1	1	1	1	1	1	1	1	1	0	0	0	1	1
1	1	1	1	1	1	0	1	1	1	1	0	0	0	0	1	1
1	1	0	0	0	1	0	0	0	0	0	0	0	0	0	1	1
0	0	0	0	0	1	0	0	0	1	0	0	0	0	0	1	0
0	0	0	0	0	1	0	0	0	0	0	0	0	0	0	1	1
0	0	0	0	0	1	0	0	0	0	0	0	0	0	0	1	0
0	0	0	0	0	1	0	0	0	0	0	0	0	0	0	1	0
0	0	0	0	0	1	0	0	0	0	0	0	0	0	0	1	0
0	0	0	0	0	1	0	0	0	0	0	0	0	1	1	1	0
0	0	0	0	0	1	0	0	0	0	0	0	0	0	0	1	0
0	0	0	0	0	1	0	0	0	0	0	0	0	0	0	1	1
0	0	0	0	0	1	0	0	0	0	0	0	0	0	0	1	1
0	0	0	0	0	1	0	0	0	0	0	0	0	0	0	1	0
1	1	0	0	0	1	0	0	0	0	0	0	0	0	0	1	0
1	1	0	0	0	1	0	0	0	0	0	0	0	0	0	1	0

PXR	HNF4alpha	HNF4beta	HNF4gamma	RXRalpha-A	RXRalpha-B	RXRbeta-A	RXRbeta-B	RXRgamma	TR2	TR4	PNR	TLL	COUPTFA-A	COUTFB	COUTFG	COUPTFA-B	EAR2-A	EAR2-B
0	1	1	0	0	0	1	1	0	1	0	0	0	0	0	0	0	1	1
0	0	0	0	0	0	1	1	0	1	0	0	0	0	0	0	0	1	0
0	0	0	0	0	0	1	1	0	1	0	0	0	0	0	0	0	1	0
0	0	0	0	0	0	1	1	0	1	0	0	0	0	0	0	0	1	1
0	0	0	0	0	0	1	1	0	1	0	0	0	0	0	0	0	1	1
0	0	0	0	0	0	1	1	0	1	0	0	0	0	0	0	0	1	1
0	0	0	0	0	0	1	1	0	1	0	0	0	0	0	0	0	1	0
0	0	0	0	0	0	1	1	0	1	0	0	0	0	0	0	0	1	0
0	1	1	0	0	0	0	0	0	0	0	0	0	0	0	0	0	0	0
0	0	0	0	0	0	1	1	0	1	0	0	0	0	0	0	0	1	0
0	1	1	0	0	0	1	1	1	1	0	0	1	1	1	1	1	1	1
0	0	0	0	0	0	1	1	1	1	0	0	0	0	0	0	0	1	0
0	0	0	0	0	0	1	1	0	1	0	0	0	0	0	0	0	1	1
0	0	0	0	0	0	1	1	0	1	0	0	0	0	0	0	0	1	0
0	0	0	0	0	0	1	1	0	1	0	0	0	1	0	0	0	1	0
0	0	0	0	0	0	1	1	0	1	0	0	0	0	0	0	0	1	0
0	0	0	0	0	0	1	1	0	1	0	0	0	1	1	1	0	1	1
0	0	0	0	0	0	1	1	0	1	0	0	0	1	1	0	0	1	0
0	0	0	0	0	0	1	1	0	1	0	0	0	0	0	0	0	1	0
0	0	0	0	0	0	1	1	1	1	0	0	1	1	1	1	1	1	1
0	0	0	0	0	0	1	1	1	1	0	0	0	0	0	0	1	1	1
0	0	0	0	0	0	1	1	0	1	0	0	1	1	1	1	1	1	1
0	0	0	0	0	0	1	1	0	1	0	0	1	1	1	1	0	1	1
0	0	0	0	0	0	1	1	0	1	0	0	0	0	0	1	0	1	1
0	0	0	0	0	0	1	1	0	1	0	0	1	1	1	1	0	1	0
0	0	0	0	0	0	1	1	0	1	0	0	0	1	1	1	1	1	1
0	0	0	0	0	0	1	1	0	1	0	0	0	0	0	1	1	1	0
0	0	0	0	0	0	1	1	0	1	0	0	0	0	0	1	0	1	0
0	0	0	0	0	0	1	1	0	1	0	0	0	0	0	0	1	1	0
0	0	0	0	0	0	1	1	0	1	0	0	0	0	0	0	0	1	0
0	0	0	0	0	0	1	1	0	1	0	0	0	0	0	0	0	1	0
0	0	0	0	0	0	1	1	0	1	0	0	0	0	0	0	0	1	0
0	1	1	0	0	0	0	0	0	0	0	0	0	0	0	0	0	0	0
0	0	0	0	0	0	1	1	0	1	0	0	0	0	0	0	0	1	0
0	1	1	0	0	1	1	1	1	1	0	1	1	1	1	1	1	1	1
0	0	0	0	0	0	1	1	0	1	0	0	0	1	0	0	0	1	0
0	0	0	0	0	0	1	1	0	1	0	0	0	0	0	0	0	1	0
0	0	0	0	1	0	1	1	0	1	0	0	0	0	1	0	0	1	0
0	0	0	0	1	0	1	1	0	1	0	0	0	0	0	0	0	1	0
0	0	0	0	0	0	1	1	0	1	0	0	0	0	1	1	0	1	1
0	0	0	0	0	0	1	1	1	1	0	0	0	1	1	0	0	1	0
0	0	0	0	0	1	1	1	0	1	0	0	1	0	1	1	0	1	0
0	0	0	0	0	1	1	1	0	1	0	0	1	1	1	1	0	1	1
0	0	0	0	0	0	1	1	0	1	0	0	1	1	1	0	1	1	1
0	0	0	0	0	0	1	1	0	1	0	0	1	1	1	0	1	1	0
0	0	0	0	0	0	1	1	0	1	0	0	1	1	1	0	1	1	1
0	0	0	0	0	1	1	1	0	1	0	0	0	1	1	1	1	1	1
0	0	0	0	0	0	1	1	0	1	0	0	0	0	0	0	0	1	0
0	0	0	0	0	1	1	1	1	1	0	0	0	1	1	1	1	1	1
0	0	0	0	0	1	1	1	1	1	0	0	0	0	1	1	1	1	1
0	0	0	0	0	0	1	1	0	1	0	0	0	0	1	0	0	1	1
0	0	0	0	0	0	1	1	0	1	0	1	0	0	0	0	0	1	0
0	0	0	0	0	1	1	1	0	1	0	0	0	0	0	0	0	1	0
0	0	0	0	0	0	1	1	0	1	0	0	0	0	0	0	0	1	0
0	0	0	0	0	1	1	1	1	1	0	0	0	0	1	1	1	1	0
0	1	0	0	0	0	1	1	0	1	0	0	0	0	0	0	0	1	0
0	0	0	0	0	0	1	1	0	1	0	0	0	0	0	0	0	1	0
0	0	0	0	0	0	1	1	0	1	0	0	0	0	0	0	0	1	0
0	0	0	0	0	0	1	1	0	1	0	0	0	0	0	0	0	1	0
0	0	1	0	0	0	0	0	0	0	0	0	0	0	0	0	0	0	0
0	0	0	0	0	0	1	1	0	1	0	0	0	0	0	0	0	1	0
1	1	1	0	0	1	1	1	1	1	1	1	1	1	1	1	1	1	1
0	0	0	0	0	0	1	1	0	1	1	0	0	0	0	0	0	1	0
0	0	0	0	0	0	1	1	0	1	1	0	0	0	0	0	0	1	0
0	0	0	0	0	0	1	1	0	1	1	0	0	0	0	0	0	1	0
0	0	0	0	0	0	1	1	0	1	1	0	0	0	0	0	0	1	0

0	0	0	0	0	0	1	1	0	1	1	1	0	0	0	0	0	1	0
1	0	0	0	0	0	1	1	0	1	1	0	0	0	0	0	0	1	0
1	0	0	0	0	1	1	1	0	1	1	0	1	1	1	1	1	1	1
1	0	0	0	0	0	1	1	0	1	1	0	1	1	1	1	1	1	1
1	0	0	0	0	0	1	1	0	1	1	0	1	1	1	1	1	1	0
1	0	0	0	0	0	1	1	0	1	1	0	1	1	1	1	1	1	1
0	0	0	0	0	0	1	1	0	1	1	0	1	1	1	1	1	1	1
0	0	0	0	0	0	1	1	0	1	1	0	0	1	1	1	1	1	1
0	0	0	0	0	0	1	1	0	1	1	0	1	0	0	0	1	1	0
0	0	0	0	0	0	1	1	0	1	1	0	0	0	0	0	0	1	1
0	0	0	0	0	1	1	1	0	1	1	0	0	1	1	1	1	1	1
0	0	0	0	0	0	1	1	0	1	1	0	0	0	1	0	0	1	0
0	0	0	0	1	1	1	1	1	1	1	0	0	1	1	1	1	1	1
0	0	0	0	0	1	1	1	1	1	1	0	0	0	1	1	1	1	1
0	0	0	0	0	1	1	1	1	1	1	0	0	0	1	1	1	1	0
0	0	0	0	0	0	1	1	0	1	1	0	1	0	1	1	0	1	0
0	0	0	0	0	0	1	1	0	1	1	0	0	0	0	0	0	1	0
0	0	0	0	1	1	1	1	0	1	1	0	0	0	0	0	0	1	0
0	0	0	0	1	1	1	1	0	1	1	0	0	0	0	1	0	1	0
0	0	0	0	0	0	1	1	0	1	1	0	0	0	0	0	0	1	0
0	0	0	0	0	0	1	1	0	1	1	0	0	0	0	0	0	1	0
0	1	0	0	0	0	1	1	0	1	1	0	0	0	0	0	0	1	0
0	0	0	0	0	0	1	1	0	1	1	0	0	0	0	0	0	1	0
0	1	0	0	0	0	1	1	0	1	1	0	0	0	0	0	0	1	0
0	1	0	0	0	0	1	1	0	1	1	0	0	0	0	0	0	1	1
0	0	0	0	0	0	1	1	0	1	1	0	0	0	0	0	0	1	0
0	0	0	0	0	0	1	1	0	1	1	0	0	0	0	0	0	1	0
0	0	0	0	0	0	1	1	0	1	1	0	0	0	0	0	0	1	0
0	0	0	0	0	0	1	1	0	1	1	0	0	0	0	0	0	1	0
0	0	0	0	0	0	1	1	0	1	1	0	0	0	0	0	0	1	0
0	0	1	0	0	0	0	0	0	0	0	0	0	0	0	0	0	0	0
0	0	0	0	0	0	1	1	0	1	1	0	0	0	0	0	0	1	0
1	1	0	0	1	1	1	1	1	1	1	1	1	1	1	1	1	1	1
0	0	0	0	0	0	1	1	0	1	1	0	0	0	0	0	0	1	0
0	0	0	0	0	0	1	1	0	1	1	0	0	0	0	0	0	1	0
0	0	0	0	0	0	1	1	1	1	1	1	1	0	1	1	0	1	0
0	0	0	0	0	0	1	1	0	1	1	0	1	0	1	1	0	1	0
0	0	0	0	0	0	1	1	1	1	1	1	1	0	0	0	0	1	0
0	0	0	0	0	0	1	1	0	1	1	0	0	0	0	0	0	1	1
0	0	0	0	0	0	1	1	0	1	1	0	0	0	0	0	0	1	0
1	0	0	0	0	1	1	1	0	1	1	0	1	1	1	1	1	1	1
1	0	0	0	1	0	1	1	0	1	1	0	1	1	1	1	1	1	1
1	0	0	0	1	0	1	1	0	1	1	0	1	0	1	0	1	1	0
1	0	0	0	1	0	1	1	0	1	1	0	1	1	1	1	1	1	1
0	0	0	0	0	0	1	1	0	1	1	0	0	0	0	0	0	1	0
0	0	0	0	1	0	1	1	0	1	1	0	1	1	1	0	1	1	1
0	0	0	0	1	0	1	1	0	1	1	0	0	1	1	0	1	1	1
0	0	0	0	1	0	1	1	0	1	1	0	1	0	0	0	1	1	0
0	0	0	0	1	0	1	1	0	1	1	0	0	0	0	1	0	1	0
0	0	0	0	1	1	1	1	0	1	1	0	0	1	1	1	1	1	1
0	0	0	0	1	1	1	1	1	1	1	0	0	1	1	1	1	1	1
0	0	0	0	1	1	1	1	1	1	1	0	0	0	1	1	1	1	1
0	0	0	0	0	0	1	1	0	1	1	0	0	0	1	0	0	1	0
0	0	0	0	0	0	1	1	0	1	1	1	0	0	0	0	0	1	0
1	0	0	0	0	0	1	1	0	1	1	0	0	0	0	0	0	1	0
0	0	0	0	0	0	1	1	0	1	1	0	0	0	0	0	0	1	0
0	0	0	0	0	1	1	1	1	1	1	0	0	1	1	1	1	1	0
0	0	0	0	1	1	1	1	0	1	1	0	0	0	0	0	0	1	0
0	0	0	0	0	0	1	1	0	1	1	0	0	0	0	0	0	1	0
0	0	0	0	0	0	1	1	0	1	1	0	0	0	0	0	0	1	0
0	0	0	0	0	0	1	1	0	1	1	0	0	0	0	0	0	1	0
0	1	0	0	0	0	1	1	0	1	1	0	0	0	0	0	0	1	0
0	0	0	0	0	0	1	1	0	1	1	0	0	0	0	0	0	1	0
0	0	0	0	0	0	1	1	0	1	1	0	0	0	0	0	0	1	0
0	0	0	0	0	0	1	1	0	1	1	0	0	0	0	0	0	1	0
0	1	0	0	0	0	1	1	0	1	1	0	0	0	0	0	0	1	0
0	1	0	0	0	0	1	1	0	1	1	0	0	0	0	0	0	1	0
0	1	0	0	0	0	1	1	0	1	1	0	0	0	0	0	0	1	0
0	0	0	0	0	0	1	1	0	1	1	0	0	0	0	0	0	1	0

0	0	0	0	0	0	1	1	0	1	1	0	0	0	0	0	0	1	0
0	0	0	0	0	0	1	1	0	1	1	0	0	0	0	0	0	1	0
0	0	0	0	0	0	1	1	0	1	1	0	0	0	0	0	0	1	0
0	0	0	0	0	0	1	1	0	1	1	0	0	0	0	0	0	1	0
1	1	0	0	1	1	1	1	1	1	1	1	1	1	1	1	1	1	1
0	0	0	0	0	0	1	1	0	1	1	0	0	0	0	0	0	1	0
1	0	0	0	1	1	1	1	0	1	1	0	1	1	1	1	1	1	0
1	0	0	0	1	0	1	1	0	1	1	0	1	1	1	0	1	1	0
1	0	0	0	1	0	1	1	0	1	1	0	1	0	1	0	1	1	0
1	0	0	0	1	0	1	1	0	1	1	0	1	1	1	0	1	1	0
0	0	0	0	0	0	1	1	0	1	1	0	0	0	0	0	0	1	0
0	0	0	0	1	1	1	1	0	1	1	0	1	1	1	0	1	1	0
0	0	0	0	1	0	1	1	0	1	1	0	0	1	1	0	1	1	0
0	0	0	0	1	1	1	1	0	1	1	0	1	1	0	0	1	1	0
0	0	0	0	1	1	1	1	0	1	1	0	0	1	1	1	1	1	0
0	0	0	0	1	0	1	1	0	1	1	0	0	0	0	0	0	1	0
0	0	0	0	1	1	1	1	1	1	1	0	0	1	1	1	0	1	1
0	0	0	0	1	1	1	1	1	1	1	0	0	0	1	1	0	1	0
0	0	0	0	0	0	1	1	0	1	1	0	0	0	1	0	0	1	0
0	0	0	0	0	0	1	1	0	1	1	0	0	1	0	0	0	1	0
1	0	0	0	0	0	1	1	0	1	1	0	0	0	0	0	0	1	0
0	0	0	0	0	0	1	1	0	1	1	1	0	0	0	0	0	1	0
0	0	0	0	0	1	1	1	0	1	1	0	0	1	1	1	1	1	0
0	0	0	0	0	1	1	1	1	1	1	1	1	0	1	1	0	1	0
0	0	0	0	0	0	1	1	0	1	1	0	1	0	1	1	0	1	0
0	0	0	0	0	1	1	1	1	1	1	1	0	0	1	0	0	1	0
0	0	0	0	0	0	1	1	1	1	1	0	0	0	1	0	0	1	0
0	0	0	0	0	1	1	1	0	1	1	0	0	0	1	0	0	1	0
0	0	0	0	0	0	1	1	1	1	1	1	0	0	0	0	0	1	0
0	0	0	0	0	0	1	1	0	1	1	0	0	0	0	0	0	1	0
0	0	0	0	1	1	1	1	0	1	1	0	0	0	0	0	0	1	0
0	1	0	0	0	0	1	1	0	1	1	0	0	0	0	0	0	1	0
0	0	0	0	0	0	1	1	0	1	1	0	0	0	0	0	0	1	0
0	0	0	0	0	0	1	1	0	1	1	0	0	0	0	0	0	1	0
1	1	0	0	0	0	1	1	0	1	1	0	0	0	0	0	0	1	0
0	0	0	0	0	0	1	1	0	1	1	0	0	0	0	0	0	1	0
0	1	0	0	0	0	1	1	0	1	1	0	0	0	0	0	0	1	0
1	1	0	0	0	0	1	1	0	1	1	0	0	0	0	0	0	1	0
0	0	0	0	0	0	1	1	0	1	1	0	0	0	0	0	0	1	0
0	0	0	0	0	0	1	1	0	1	1	0	0	0	0	0	0	1	0
0	0	0	0	0	0	1	1	0	1	1	0	0	0	0	0	0	1	0

ERalpha	ERbeta-A	ERbeta-B	ERRalpha	ERRbeta	ERRgamma	ERRbeta/gamm	ERRdelta	GR	MR	PR	AR	NGFIB	NURR1	NOR1A	LRH1	SF1-A	FF1C	SF1-B
0	0	1	1	0	0	1	0	1	0	1	0	1	0	0	0	0	0	0
0	0	1	0	0	0	1	0	1	0	1	0	1	0	0	0	0	0	0
0	0	1	1	0	0	1	0	1	0	1	0	1	0	0	0	0	0	0
0	0	1	0	0	0	1	0	1	0	1	0	1	0	0	0	0	0	0
0	0	1	0	0	0	1	0	1	0	1	0	1	0	0	0	0	0	0
0	0	1	0	0	0	1	0	1	0	1	0	1	0	0	0	0	0	0
0	0	1	0	0	0	1	0	1	0	1	0	1	0	0	0	0	0	0
0	0	1	0	0	0	1	0	1	0	1	0	1	0	0	0	0	0	0
0	0	1	0	0	0	0	0	0	0	1	0	0	0	0	0	0	0	0
0	0	1	0	0	0	1	0	1	0	1	0	1	0	0	0	0	0	0
0	0	1	1	0	1	1	0	1	0	1	0	1	0	0	0	0	0	0
0	0	1	0	0	0	1	0	1	0	1	0	1	0	0	0	0	0	0
0	0	1	0	0	0	1	0	1	0	1	0	1	0	0	0	0	0	0
0	0	1	0	0	1	1	0	1	0	1	0	1	0	0	0	0	0	0
0	0	1	1	0	0	1	0	1	0	1	0	1	0	0	0	0	0	0
0	0	1	0	0	0	1	0	1	0	1	0	1	0	0	0	0	0	0
0	0	1	1	0	0	1	0	1	0	1	0	1	0	0	0	0	0	0
0	0	1	0	0	0	1	0	1	0	1	0	1	0	0	0	0	0	0
0	0	1	0	0	0	1	0	1	0	1	0	1	0	0	0	0	0	0
0	0	1	0	0	0	1	0	1	0	1	0	1	0	0	0	0	0	0
0	0	1	0	0	0	1	0	1	0	1	0	1	0	0	0	0	0	0
0	0	1	0	0	0	1	0	1	0	1	0	1	0	0	0	0	0	0
0	0	1	0	0	0	1	0	1	0	1	0	1	0	0	0	0	0	0
0	0	1	0	0	0	1	0	1	0	1	0	1	0	0	0	0	0	0
0	0	1	0	0	0	1	0	1	0	1	0	1	0	0	0	0	0	0
0	0	1	0	0	0	1	0	1	0	1	0	1	0	0	0	0	0	0
0	0	1	0	0	0	1	0	1	0	1	0	1	0	0	0	0	0	0
0	0	1	0	0	0	1	0	1	0	1	0	1	0	0	0	0	0	0
0	0	1	0	0	0	1	0	1	0	1	0	1	0	0	0	0	0	0
0	0	1	0	0	0	1	0	1	0	1	0	1	0	0	0	0	0	0
0	0	1	0	0	0	1	0	1	0	1	0	1	0	0	0	0	0	0
0	0	1	1	0	0	1	0	1	0	1	0	1	0	0	0	0	0	0
0	0	1	0	0	0	0	0	0	0	1	0	0	0	0	0	0	0	0
0	0	1	0	0	0	1	0	1	0	1	0	1	0	0	0	0	0	0
0	0	1	1	1	1	1	1	1	0	1	0	1	0	0	1	0	0	1
0	0	1	0	0	0	1	0	1	0	1	0	1	0	0	0	0	0	0
0	0	1	0	0	0	1	0	1	0	1	0	1	0	0	0	0	0	0
0	0	1	1	0	0	1	0	1	0	1	0	1	0	0	0	0	0	0
0	0	1	0	0	0	1	0	1	0	1	0	1	0	0	0	0	0	0
0	0	1	1	0	0	1	0	1	0	1	0	1	0	0	1	0	0	0
0	0	1	1	0	0	1	0	1	0	1	0	1	0	0	0	0	0	0
0	0	1	0	0	0	1	0	1	0	1	0	1	0	0	0	0	0	0
0	0	1	1	0	0	1	0	1	0	1	0	1	0	0	0	0	0	0
0	0	1	1	1	0	1	0	1	0	1	0	1	0	0	0	0	0	1
0	0	1	0	0	0	1	0	1	0	1	0	1	0	0	0	0	0	1
0	0	1	0	0	0	1	0	1	0	1	0	1	0	0	0	0	0	1
0	0	1	1	0	0	1	0	1	0	1	0	1	0	0	0	0	0	0
0	0	1	0	1	0	1	0	1	0	1	0	1	0	0	0	0	0	0
0	0	1	0	0	0	1	0	1	0	1	0	1	0	0	0	0	0	0
0	0	1	0	1	0	1	0	1	0	1	0	1	0	0	1	0	0	0
0	0	1	0	0	0	1	0	1	0	1	0	1	0	0	0	0	0	0
0	0	1	0	0	0	1	0	1	0	1	0	1	0	0	0	0	0	0
0	0	1	0	0	0	1	0	1	0	1	0	1	0	0	0	0	0	0
0	0	1	1	0	1	1	0	1	0	1	0	1	0	0	0	0	0	0
0	0	1	0	0	0	1	0	1	0	1	0	1	0	0	0	0	0	0
0	0	1	0	0	0	1	0	1	0	1	0	1	0	0	0	0	0	0
0	0	1	1	1	1	1	0	1	0	1	0	1	0	0	0	0	0	0
0	0	1	0	0	0	1	0	1	0	1	0	1	0	0	1	0	0	0
0	0	1	0	0	0	1	0	1	0	1	0	1	0	0	0	0	0	0
0	0	1	1	0	0	1	1	1	0	1	0	1	0	0	0	0	0	0
0	0	1	0	0	0	1	0	1	0	1	0	1	0	0	0	0	0	0
0	0	1	0	0	0	0	0	0	0	1	0	0	0	0	0	0	0	0
0	0	1	0	0	0	1	0	1	0	1	0	1	0	0	0	0	0	0
0	0	1	1	1	1	1	1	1	0	1	0	1	1	1	1	1	0	1
0	0	1	0	0	0	1	0	1	0	1	0	1	0	0	0	0	0	0
0	0	1	0	0	0	1	0	1	0	1	0	1	0	0	0	0	0	0
0	0	1	0	0	0	1	0	1	0	1	0	1	0	0	0	0	0	0
0	0	1	0	0	0	1	0	1	0	1	0	1	0	0	0	0	0	0

0	0	1	1	1	1	1	0	1	0	1	0	1	0	0	0	0	0	0
0	0	1	0	0	0	1	0	1	0	1	0	1	0	0	0	0	0	0
0	0	1	1	1	1	1	0	1	0	1	0	1	1	1	1	1	0	1
0	0	1	1	1	1	1	0	1	0	1	0	1	1	1	1	1	0	1
0	0	1	1	0	0	1	0	1	0	1	0	1	1	1	0	0	0	0
0	0	1	0	1	1	1	0	1	0	1	0	1	0	1	1	1	0	1
0	0	1	1	1	1	1	0	1	0	1	0	1	1	1	0	0	0	0
0	0	1	0	1	1	1	0	1	0	1	0	1	1	1	0	0	0	0
0	0	1	1	0	0	1	0	1	0	1	0	1	0	0	0	0	0	0
0	0	1	1	0	0	1	0	1	0	1	0	1	0	0	0	0	0	0
0	0	1	0	1	1	1	0	1	0	1	0	1	1	1	0	0	0	0
0	0	1	0	0	1	1	0	1	0	1	0	1	0	0	0	0	0	0
0	0	1	1	1	1	1	0	1	0	1	0	1	0	0	1	0	0	0
0	0	1	0	0	0	1	0	1	0	1	0	1	0	0	1	0	0	0
0	0	1	0	0	0	1	0	1	0	1	0	1	0	0	0	0	0	0
0	0	1	1	0	0	1	1	1	0	1	0	1	0	0	0	0	0	0
0	0	1	0	0	0	1	0	1	0	1	0	1	0	0	0	0	0	0
0	0	1	0	0	1	1	0	1	0	1	0	1	0	0	0	0	0	0
0	0	1	1	0	0	1	0	1	0	1	0	1	0	0	0	0	0	0
0	0	1	1	0	0	1	0	1	0	1	0	1	0	0	0	0	0	0
0	0	1	1	0	0	1	1	1	0	1	0	1	0	0	0	0	0	0
0	0	1	1	1	0	1	0	1	0	1	0	1	0	0	0	0	0	0
0	0	1	0	0	0	1	0	1	0	1	0	1	0	0	0	1	0	0
0	0	1	0	0	0	1	0	1	0	1	0	1	0	0	1	0	0	0
0	0	1	0	0	0	1	0	1	0	1	0	1	0	0	1	0	0	0
0	0	1	0	0	0	1	0	1	0	1	0	1	0	0	0	0	0	0
0	0	1	0	0	0	1	0	1	0	1	0	1	0	0	0	0	0	0
0	0	1	0	0	0	1	0	1	0	1	0	1	0	0	0	0	0	0
0	0	1	0	0	0	1	0	1	0	1	0	1	0	0	0	0	0	0
0	0	1	0	0	0	1	0	1	0	1	0	1	0	0	0	0	0	0
0	0	1	0	0	0	0	0	1	0	1	0	0	0	0	0	0	0	0
0	0	1	0	0	0	1	0	1	0	1	0	1	0	0	0	0	0	0
0	1	1	1	1	1	1	1	1	0	1	0	1	1	1	1	1	0	1
0	0	1	0	0	0	1	0	1	0	1	0	1	0	0	0	0	0	0
0	0	1	0	0	0	1	0	1	0	1	0	1	0	0	0	0	0	0
0	0	1	1	0	0	1	1	1	0	1	0	1	0	0	0	0	0	0
0	0	1	1	0	0	1	0	1	0	1	0	1	0	0	0	0	0	0
0	0	1	1	0	0	1	1	1	0	1	0	1	0	0	0	0	0	0
0	0	1	0	0	0	1	0	1	0	1	0	1	0	0	0	0	0	0
0	0	1	0	0	0	1	0	1	0	1	0	1	0	0	0	0	0	0
0	1	1	1	1	1	1	0	1	0	1	0	1	1	1	1	1	0	1
0	1	1	1	1	1	1	0	1	0	1	0	1	1	1	1	1	0	1
0	1	1	1	0	0	1	0	1	0	1	0	1	1	1	0	0	0	0
0	1	1	0	1	1	1	0	1	0	1	0	1	1	1	1	1	0	1
0	0	1	0	0	0	1	0	1	0	1	0	1	0	1	0	0	0	0
0	0	1	1	1	1	1	0	1	0	1	0	1	1	1	1	0	0	0
0	0	1	0	1	1	1	0	1	0	1	0	1	1	1	1	0	0	0
0	0	1	1	0	0	1	0	1	0	1	0	1	0	0	0	0	0	0
0	0	1	1	0	0	1	0	1	0	1	0	1	0	0	0	0	0	0
0	0	1	1	1	1	1	0	1	0	1	0	1	1	1	1	0	0	0
0	0	1	0	0	1	1	0	1	0	1	0	1	1	0	1	0	0	0
0	0	1	0	0	0	1	0	1	0	1	0	1	1	0	1	0	0	0
0	0	1	0	0	1	1	0	1	0	1	0	1	0	0	0	0	0	0
0	0	1	1	1	1	1	1	1	0	1	0	1	0	0	0	0	0	0
0	0	1	0	0	0	1	0	1	0	1	0	1	0	0	0	0	0	0
0	0	1	0	0	0	1	0	1	0	1	0	1	0	0	0	0	0	0
0	0	1	0	0	0	1	0	1	0	1	0	1	0	0	1	0	0	0
0	0	1	0	0	0	1	0	1	0	1	0	1	0	0	0	0	0	0
0	0	1	0	0	0	1	0	1	0	1	0	1	0	0	0	0	0	0
0	0	1	0	0	0	1	0	1	0	1	0	1	0	0	0	0	0	0
0	0	1	1	0	0	1	0	1	0	1	0	1	0	0	0	0	0	0
0	0	1	1	1	0	1	0	1	0	1	0	1	0	0	0	0	0	0
0	0	1	0	0	0	1	0	1	0	1	0	1	0	0	0	1	0	0
0	0	1	1	0	0	1	1	1	0	1	0	1	0	0	0	0	0	0
0	1	1	0	0	0	1	0	1	0	1	0	1	0	0	0	0	0	0
0	0	1	0	0	0	1	0	1	0	1	0	1	0	0	0	0	0	0
0	0	1	0	0	0	1	0	1	0	1	0	1	0	0	0	0	0	0
0	1	1	0	0	0	1	0	1	0	1	0	1	0	0	0	0	0	0
0	0	1	0	0	0	1	0	1	0	1	0	1	0	0	0	0	0	0

0	0	1	0	0	0	1	0	1	0	1	0	1	0	0	0	0	0	0
0	0	1	0	0	0	1	0	1	0	1	0	1	0	0	0	0	0	0
0	0	1	0	0	0	1	0	1	0	1	0	1	0	0	0	0	0	0
0	0	1	0	0	0	1	0	1	0	1	0	1	0	0	0	0	0	0
0	1	1	1	1	1	1	1	1	0	1	0	1	1	1	1	1	0	1
0	0	1	0	0	0	1	0	1	0	1	0	0	0	0	0	0	0	0
0	1	1	1	1	1	1	0	1	0	1	0	0	1	1	1	1	0	1
0	1	1	1	1	1	1	0	1	0	1	0	0	1	1	0	0	0	1
0	0	1	1	0	0	1	0	1	0	1	0	0	1	1	0	0	0	0
0	1	1	0	1	1	1	0	1	0	1	0	0	1	1	1	1	0	1
0	0	1	0	0	0	1	0	1	0	1	0	0	0	1	0	0	0	0
0	0	1	1	1	1	1	0	1	0	1	0	0	1	1	1	0	0	0
0	0	1	1	1	1	1	0	1	0	1	0	0	1	1	1	0	0	0
0	0	1	1	1	1	1	0	1	0	1	0	0	0	0	1	0	0	0
0	0	1	1	1	1	1	0	1	0	1	0	0	1	1	1	0	0	0
0	0	1	1	0	0	1	0	1	0	1	0	0	1	0	0	0	0	0
0	0	1	1	1	1	1	0	1	0	1	0	0	1	0	1	0	0	0
0	0	1	1	1	1	1	0	1	0	1	0	0	1	0	1	0	0	0
0	0	1	0	0	1	1	0	1	0	1	0	0	0	0	0	0	0	0
0	0	1	0	0	0	1	0	1	0	1	0	0	0	0	0	0	0	0
0	0	1	0	0	0	1	0	1	0	1	0	0	0	0	0	0	0	0
0	0	1	0	0	0	1	0	1	0	1	0	0	0	0	0	0	0	0
0	0	1	1	0	0	1	0	1	0	1	0	0	0	0	1	0	0	0
0	0	1	1	0	1	1	1	1	0	1	0	0	1	0	0	0	0	0
0	0	1	0	0	0	1	0	1	0	1	0	0	0	0	0	0	0	0
0	0	1	1	0	1	1	1	1	0	1	0	0	1	0	0	0	0	0
0	0	1	1	0	1	1	1	1	0	1	0	0	1	0	0	0	0	0
0	0	1	1	0	0	1	0	1	0	1	0	0	0	0	0	0	0	0
0	0	1	1	0	0	1	0	1	0	1	0	0	0	0	0	0	0	0
0	0	1	0	0	0	1	0	1	0	1	0	1	0	0	0	0	0	0
0	0	1	1	0	0	1	0	1	0	1	0	0	0	0	0	0	0	0
0	0	1	1	1	0	1	0	1	0	1	0	0	0	0	0	0	0	0
0	0	1	0	0	0	1	0	1	0	1	0	0	0	0	0	1	0	0
0	0	1	0	0	0	1	0	1	0	1	0	0	0	0	0	0	0	0
0	1	1	0	0	0	1	0	1	0	1	0	0	0	0	1	0	0	0
0	1	1	0	0	0	1	0	1	0	1	0	0	0	0	0	0	0	1
0	0	1	0	0	0	1	0	1	0	1	0	0	0	0	0	0	0	0
0	0	1	0	0	0	1	0	1	0	1	0	0	0	0	0	0	0	0
0	0	1	0	0	0	1	0	1	0	1	0	1	0	0	0	0	0	0
0	0	1	0	0	0	1	0	1	0	1	0	1	0	0	0	0	0	0
0	0	1	0	0	0	1	0	1	0	1	0	0	0	0	0	0	0	0

GCNF-A	GCNF-B	SHP-A	SHP-B	DAX	SRC1	SRC2	SRC3	CBP-A	CBP-B	P300-A	P300-B	BRG1	PCAF	CARM1	RIP140-A	RIP140-B	NCOA6	BAF53
1	0	1	0	1	1	1	1	1	1	1	1	1	0	1	1	0	1	1
1	0	0	0	1	1	1	0	1	1	1	1	1	0	1	1	0	1	1
1	0	0	0	1	1	1	1	1	1	1	1	1	0	1	1	0	1	1
1	0	0	0	1	1	1	1	1	1	1	1	1	0	1	1	0	1	1
1	0	0	0	1	1	1	0	1	1	1	1	1	0	1	1	0	1	1
1	0	0	0	1	1	1	0	1	1	1	1	1	0	1	1	0	1	1
1	0	0	0	1	1	1	0	1	1	1	1	1	0	1	0	0	1	1
1	0	0	0	1	1	1	0	1	1	1	1	1	0	1	0	0	1	1
0	0	1	0	0	0	0	0	0	0	0	0	0	0	0	0	0	0	0
1	0	0	0	1	1	1	0	1	1	1	1	1	0	1	0	0	1	1
1	0	1	0	1	1	1	1	1	1	1	1	1	0	1	1	1	1	1
1	0	0	0	1	1	1	0	1	1	1	1	1	0	1	1	0	1	1
1	0	0	0	1	1	1	0	1	1	1	1	1	0	1	1	1	1	1
1	0	0	0	1	1	1	1	1	1	1	1	1	0	1	0	0	1	1
1	0	0	0	1	1	1	0	1	1	1	1	1	0	1	0	0	1	1
1	0	0	0	1	1	1	0	1	1	1	1	1	0	1	0	0	1	1
1	0	0	0	1	1	1	0	1	1	1	1	1	0	1	1	1	1	1
1	0	0	0	1	1	1	0	1	1	1	1	1	0	1	1	1	1	1
1	0	0	0	1	1	1	0	1	1	1	1	1	0	1	1	0	1	1
1	0	0	0	1	1	1	1	1	1	1	1	1	0	1	0	1	1	1
1	0	0	0	1	1	1	1	1	1	1	1	1	0	1	0	1	1	1
1	0	0	0	1	1	1	0	1	1	1	1	1	0	1	0	0	1	1
1	0	0	0	1	1	1	0	1	1	1	1	1	0	1	0	0	1	1
1	0	0	0	1	1	1	0	1	1	1	1	1	0	1	0	0	1	1
1	0	0	0	1	1	1	0	1	1	1	1	1	0	1	0	0	1	1
1	0	0	0	1	1	1	0	1	1	1	1	1	0	1	0	0	1	1
1	0	0	0	1	1	1	0	1	1	1	1	1	0	1	0	0	1	1
1	0	0	0	1	1	1	0	1	1	1	1	1	0	1	0	0	1	1
1	0	0	0	1	1	1	0	1	1	1	1	1	0	1	0	0	1	1
1	0	0	0	1	1	1	0	1	1	1	1	1	0	1	0	0	1	1
1	0	0	0	1	1	1	0	1	1	1	1	1	0	1	0	0	1	1
1	0	0	0	1	1	1	0	1	1	1	1	1	0	1	0	0	1	1
0	0	1	0	0	0	0	0	0	0	0	0	0	0	0	0	0	0	0
1	0	0	0	1	1	1	0	1	1	1	1	1	0	1	0	0	1	1
1	1	1	0	1	1	1	1	1	1	1	1	1	0	1	1	1	1	1
1	0	0	0	1	1	1	0	1	1	1	1	1	0	1	0	1	1	1
1	0	0	0	1	1	1	0	1	1	1	1	1	0	1	0	0	1	1
1	1	0	0	1	1	1	0	1	1	1	1	1	0	1	0	1	1	1
1	1	0	0	1	1	1	0	1	1	1	1	1	0	1	0	0	1	1
1	0	0	0	1	1	1	0	1	1	1	1	1	0	1	1	1	1	1
1	0	0	0	1	1	1	0	1	1	1	1	1	0	1	1	0	1	1
1	0	0	0	1	1	1	0	1	1	1	1	1	0	1	0	1	1	1
1	0	0	0	1	1	1	0	1	1	1	1	1	0	1	0	1	1	1
1	0	0	0	1	1	1	0	1	1	1	1	1	0	1	0	1	1	1
1	0	0	0	1	1	1	0	1	1	1	1	1	0	1	0	0	1	1
1	0	0	0	1	1	1	0	1	1	1	1	1	0	1	0	0	1	1
1	0	0	0	1	1	1	0	1	1	1	1	1	0	1	0	1	1	1
1	0	0	0	1	1	1	0	1	1	1	1	1	0	1	0	0	1	1
1	0	0	0	1	1	1	0	1	1	1	1	1	0	1	1	0	1	1
1	0	0	0	1	1	1	1	1	1	1	1	1	0	1	1	0	1	1
1	0	0	0	1	1	1	1	1	1	1	1	1	0	1	0	0	1	1
1	0	0	0	1	1	1	0	1	1	1	1	1	0	1	0	0	1	1
1	0	0	0	1	1	1	0	1	1	1	1	1	0	1	0	0	1	1
1	1	0	0	1	1	1	1	1	1	1	1	1	0	1	0	0	1	1
1	0	0	0	1	1	1	0	1	1	1	1	1	0	1	0	0	1	1
1	0	0	0	1	1	1	1	1	1	1	1	1	0	1	0	0	1	1
1	0	0	0	1	1	1	0	1	1	1	1	1	0	1	0	0	1	1
1	0	0	0	1	1	1	0	1	1	1	1	1	0	1	0	0	1	1
1	0	0	0	1	1	1	0	1	1	1	1	1	0	1	0	0	1	1
1	0	0	0	1	1	1	0	1	1	1	1	1	0	1	0	0	1	1
1	0	0	0	1	1	1	0	1	1	1	1	1	0	1	1	0	1	1
0	0	1	0	0	0	0	0	0	0	0	0	0	0	0	0	0	0	0
1	0	0	0	1	1	1	0	1	1	1	1	1	0	1	0	0	1	1
1	1	1	0	1	1	1	1	1	1	1	1	1	0	1	1	1	1	1
1	0	0	0	1	1	1	0	1	1	1	1	1	0	1	0	0	1	1
1	0	0	0	1	1	1	0	1	1	1	1	1	0	1	1	0	1	1
1	0	0	0	1	1	1	0	1	1	1	1	1	0	1	1	1	1	1
1	0	0	0	1	1	1	0	1	1	1	1	1	0	1	0	0	1	1

1	0	0	0	1	1	1	0	1	1	1	1	1	0	1	0	0	1	1
1	0	0	0	1	1	1	0	1	1	1	1	1	0	1	0	0	1	1
1	0	0	0	1	1	1	1	1	1	1	1	1	0	1	0	1	1	1
1	0	0	0	1	1	1	1	1	1	1	1	1	0	1	0	0	1	1
1	0	0	0	1	1	1	1	1	1	1	1	1	0	1	0	0	1	1
1	0	0	0	1	1	1	1	1	1	1	1	1	0	1	0	0	1	1
1	0	0	0	1	1	1	1	1	1	1	1	1	0	1	0	0	1	1
1	0	0	0	1	1	1	1	1	1	1	1	1	0	1	0	0	1	1
1	0	0	0	1	1	1	1	1	1	1	1	1	0	1	0	0	1	1
1	0	0	0	1	1	1	1	1	1	1	1	1	0	1	0	1	1	1
1	0	0	0	1	1	1	1	1	1	1	1	1	0	1	0	0	1	1
1	0	0	0	1	1	1	0	1	1	1	1	1	0	1	1	0	1	1
1	1	0	0	1	1	1	1	1	1	1	1	1	0	1	0	1	1	1
1	0	0	0	1	1	1	1	1	1	1	1	1	0	1	0	1	1	1
1	0	0	0	1	1	1	1	1	1	1	1	1	0	1	1	1	1	1
1	0	0	0	1	1	1	0	1	1	1	1	1	0	1	1	1	1	1
1	0	0	0	1	1	1	0	1	1	1	1	1	0	1	0	0	1	1
1	1	0	0	1	1	1	0	1	1	1	1	1	0	1	0	0	1	1
1	1	0	0	1	1	1	0	1	1	1	1	1	0	1	1	1	1	1
1	0	0	0	1	1	1	0	1	1	1	1	1	0	1	0	0	1	1
1	0	0	0	1	1	1	0	1	1	1	1	1	0	1	0	0	1	1
1	0	0	0	1	1	1	0	1	1	1	1	1	0	1	0	0	1	1
1	0	0	0	1	1	1	0	1	1	1	1	1	0	1	0	0	1	1
1	0	0	0	1	1	1	0	1	1	1	1	1	0	1	0	1	1	1
1	0	0	0	1	1	1	0	1	1	1	1	1	0	1	0	1	1	1
1	0	0	0	1	1	1	0	1	1	1	1	1	0	1	0	0	1	1
1	0	0	0	1	1	1	0	1	1	1	1	1	0	1	0	0	1	1
1	0	0	0	1	1	1	0	1	1	1	1	1	0	1	0	0	1	1
1	0	0	0	1	1	1	0	1	1	1	1	1	0	1	0	0	1	1
1	0	0	0	1	1	1	0	1	1	1	1	1	0	1	1	0	1	1
0	0	1	0	0	0	0	0	0	0	0	0	0	0	0	0	0	0	0
1	0	0	0	1	1	1	0	1	1	1	1	1	0	1	0	0	1	1
0	1	0	0	1	1	1	1	1	1	1	1	1	0	1	1	1	1	1
0	0	0	0	1	1	1	0	1	1	1	1	1	0	1	0	0	1	1
0	0	0	0	1	1	1	0	1	1	1	1	1	0	1	1	0	1	1
0	0	0	0	1	1	1	0	1	1	1	1	1	0	1	1	1	1	1
0	0	0	0	1	1	1	0	1	1	1	1	1	0	1	1	1	1	1
0	0	0	0	1	1	1	0	1	1	1	1	1	0	1	0	1	1	1
0	0	0	0	1	1	1	0	1	1	1	1	1	0	1	0	0	1	1
0	0	0	0	1	1	1	0	1	1	1	1	1	0	1	1	0	1	1
0	0	0	0	1	1	1	1	1	1	1	1	1	0	1	1	1	1	1
0	0	0	0	1	1	1	1	1	1	1	1	1	0	1	1	1	1	1
0	0	0	0	1	1	1	1	1	1	1	1	1	0	1	1	1	1	1
0	0	0	0	1	1	1	1	1	1	1	1	1	0	1	0	1	1	1
0	0	0	0	1	1	1	0	1	1	1	1	1	0	1	0	0	1	1
0	0	0	0	1	1	1	1	1	1	1	1	1	0	1	1	1	1	1
0	0	0	0	1	1	1	1	1	1	1	1	1	0	1	1	1	1	1
0	0	0	0	1	1	1	1	1	1	1	1	1	0	1	1	1	1	1
0	0	0	0	1	1	1	1	1	1	1	1	1	0	1	1	1	1	1
0	0	0	0	1	1	1	1	1	1	1	1	1	0	1	1	1	1	1
0	1	0	0	1	1	1	1	1	1	1	1	1	0	1	1	1	1	1
0	0	0	0	1	1	1	1	1	1	1	1	1	0	1	1	1	1	1
0	0	0	0	1	1	1	0	1	1	1	1	1	0	1	1	0	1	1
0	0	0	0	1	1	1	0	1	1	1	1	1	0	1	0	0	1	1
0	0	0	0	1	1	1	0	1	1	1	1	1	0	1	0	0	1	1
0	0	0	0	1	1	1	0	1	1	1	1	1	0	1	0	0	1	1
0	1	0	0	1	1	1	1	1	1	1	1	1	0	1	1	1	1	1
0	1	0	0	1	1	1	0	1	1	1	1	1	0	1	1	1	1	1
0	0	0	0	1	1	1	0	1	1	1	1	1	0	1	0	0	1	1
0	0	0	0	1	1	1	0	1	1	1	1	1	0	1	1	0	1	1
0	0	0	0	1	1	1	0	1	1	1	1	1	0	1	0	0	1	1
0	0	0	0	1	1	1	0	1	1	1	1	1	0	1	0	0	1	1
0	0	0	0	1	1	1	0	1	1	1	1	1	0	1	0	0	1	1
0	0	0	0	1	1	1	0	1	1	1	1	1	0	1	0	0	1	1
0	0	0	0	1	1	1	0	1	1	1	1	1	0	1	0	0	1	1
0	0	0	0	1	1	1	0	1	1	1	1	1	0	1	0	1	1	1
0	0	0	0	1	1	1	0	1	1	1	1	1	0	1	0	0	1	1
0	0	0	0	1	1	1	0	1	1	1	1	1	0	1	0	0	1	1
0	0	0	0	1	1	1	0	1	1	1	1	1	0	1	0	0	1	1

0	0	0	0	1	1	1	0	1	1	1	1	1	0	1	0	0	1	1
0	0	0	0	1	1	1	0	1	1	1	1	1	0	1	0	0	1	1
1	0	0	0	1	1	1	0	1	1	1	1	1	0	1	1	0	1	1
0	0	0	0	1	1	1	0	1	1	1	1	1	0	1	0	0	1	1
0	1	0	0	1	1	1	1	1	1	1	1	1	0	1	1	1	1	1
0	0	0	0	1	1	1	0	1	1	1	1	1	0	1	1	0	1	1
0	0	0	0	1	1	1	1	1	1	1	1	1	0	1	1	1	1	1
0	0	0	0	1	1	1	1	1	1	1	1	1	0	1	1	1	1	1
0	0	0	0	1	1	1	1	1	1	1	1	1	0	1	1	1	1	1
0	0	0	0	1	1	1	1	1	1	1	1	1	0	1	1	1	1	1
0	0	0	0	1	1	1	0	1	1	1	1	1	0	1	0	1	1	1
0	0	0	0	1	1	1	1	1	1	1	1	1	0	1	1	1	1	1
0	0	0	0	1	1	1	1	1	1	1	1	1	0	1	1	1	1	1
0	0	0	0	1	1	1	1	1	1	1	1	1	0	1	1	1	1	1
0	0	0	0	1	1	1	1	1	1	1	1	1	0	1	1	1	1	1
0	0	0	0	1	1	1	1	1	1	1	1	1	0	1	1	1	1	1
0	0	0	0	1	1	1	0	1	1	1	1	1	0	1	0	0	1	1
0	0	0	0	1	1	1	0	1	1	1	1	1	0	1	0	0	1	1
0	0	0	0	1	1	1	0	1	1	1	1	1	0	1	1	0	1	1
0	0	0	0	1	1	1	0	1	1	1	1	1	0	1	0	0	1	1
0	0	0	0	1	1	1	0	1	1	1	1	1	0	1	0	0	1	1
0	0	0	0	1	1	1	0	1	1	1	1	1	0	1	0	0	1	1
0	1	0	0	1	1	1	1	1	1	1	1	1	0	1	1	0	1	1
0	0	0	0	1	1	1	0	1	1	1	1	1	0	1	1	1	1	1
0	0	0	0	1	1	1	0	1	1	1	1	1	0	1	1	0	1	1
0	0	0	0	1	1	1	0	1	1	1	1	1	0	1	1	1	1	1
0	0	0	0	1	1	1	0	1	1	1	1	1	0	1	1	1	1	1
0	0	0	0	1	1	1	0	1	1	1	1	1	0	1	1	1	1	1
0	0	0	0	1	1	1	0	1	1	1	1	1	0	1	1	0	1	1
0	1	0	0	1	1	1	0	1	1	1	1	1	0	1	0	0	1	1
0	1	0	0	1	1	1	0	1	1	1	1	1	0	1	0	0	1	1
0	0	0	0	1	1	1	0	1	1	1	1	1	0	1	0	0	1	1
0	0	0	0	1	1	1	0	1	1	1	1	1	0	1	0	0	1	1
0	0	0	0	1	1	1	0	1	1	1	1	1	0	1	0	0	1	1
0	0	0	0	1	1	1	0	1	1	1	1	1	0	1	0	0	1	1
0	0	0	0	1	1	1	0	1	1	1	1	1	0	1	0	0	1	1
0	0	0	0	1	1	1	0	1	1	1	1	1	0	1	0	0	1	1
0	0	0	0	1	1	1	0	1	1	1	1	1	0	1	0	0	1	1
0	0	0	0	1	1	1	0	1	1	1	1	1	0	1	0	0	1	1
0	0	0	0	1	1	1	0	1	1	1	1	1	0	1	0	0	1	1
0	0	0	0	1	1	1	0	1	1	1	1	1	0	1	0	0	1	1

TRAP220	MYST-HAT	NCOA4	PGC1	TRIP13	ARA54	Cited3	Baf60	NCoR	SMRT	TRIP7	Alien	HDAC1	HDAC3	HDAC4	TBL1RHDAC3s	TIF1alpha	TIF1gamma
0	0	1	1	1	0	1	1	1	1	0	1	1	1	0	1	1	1
0	0	1	0	1	0	0	1	1	0	0	1	1	1	0	1	1	1
0	0	1	0	1	0	0	1	1	0	0	1	1	1	0	1	1	1
0	0	1	0	1	0	1	1	1	0	0	1	1	1	0	1	1	1
0	0	1	0	1	0	1	1	1	1	0	1	1	1	0	1	1	1
0	0	1	1	1	0	0	1	1	0	0	1	1	1	0	1	1	1
0	0	1	0	1	0	0	1	1	0	0	1	1	1	0	1	1	1
0	0	1	0	1	0	0	1	1	0	0	1	1	1	0	1	1	1
0	0	0	0	0	0	0	0	0	0	0	0	0	0	0	0	0	0
0	0	1	0	1	0	0	1	1	0	0	1	1	1	0	1	1	1
0	0	1	1	1	0	1	1	1	1	0	1	1	1	0	1	1	1
0	0	1	0	1	0	1	1	1	0	0	1	1	1	0	1	1	1
0	0	1	0	1	0	1	1	1	1	0	1	1	1	0	1	1	1
0	0	1	0	1	0	0	1	1	0	0	1	1	1	0	1	1	1
0	0	1	0	1	0	0	1	1	0	0	1	1	1	0	1	1	1
0	0	1	0	1	0	0	1	1	0	0	1	1	1	0	1	1	1
0	0	1	1	1	0	0	1	1	0	0	1	1	1	0	1	1	1
0	0	1	1	1	0	0	1	1	1	0	1	1	1	0	1	1	1
0	0	1	1	1	0	0	1	1	0	0	1	1	1	0	1	1	1
0	0	1	0	1	0	0	1	1	0	0	1	1	1	0	1	1	1
0	0	1	0	1	0	0	1	1	0	0	1	1	1	0	1	1	1
0	0	1	0	1	0	0	1	1	0	0	1	1	1	0	1	1	1
0	0	1	0	1	0	0	1	1	0	0	1	1	1	0	1	1	1
0	0	1	0	1	0	0	1	1	0	0	1	1	1	0	1	1	1
0	0	1	0	1	0	0	1	1	0	0	1	1	1	0	1	1	1
0	0	1	0	1	0	0	1	1	0	0	1	1	1	0	1	1	1
0	0	1	0	1	0	0	1	1	0	0	1	1	1	0	1	1	1
0	0	1	0	1	0	0	1	1	0	0	1	1	1	0	1	1	1
0	0	1	0	1	0	0	1	1	0	0	1	1	1	0	1	1	1
0	0	1	0	1	0	0	1	1	0	0	1	1	1	0	1	1	1
0	0	1	0	1	0	0	1	1	0	0	1	1	1	0	1	1	1
0	0	1	0	1	0	0	1	1	0	0	1	1	1	0	1	1	1
0	0	1	0	1	0	0	1	1	0	0	1	1	1	0	1	1	1
0	0	0	0	0	0	0	0	0	0	0	0	0	0	0	0	0	0
0	0	1	0	1	0	0	1	1	0	0	1	1	1	0	1	1	1
0	0	1	1	1	0	1	1	1	1	1	1	1	1	1	1	1	1
0	0	1	0	1	0	1	1	1	0	0	1	1	1	0	1	1	1
0	0	1	0	1	0	0	1	1	0	0	1	1	1	0	1	1	1
0	0	1	0	1	0	0	1	1	1	0	1	1	1	0	1	1	1
0	0	1	0	1	0	0	1	1	0	0	1	1	1	0	1	1	1
0	0	1	0	1	0	1	1	1	1	0	1	1	1	0	1	1	1
0	0	1	1	1	0	1	1	1	0	0	1	1	1	0	1	1	1
0	0	1	0	1	0	0	1	1	0	0	1	1	1	0	1	1	1
0	0	1	0	1	0	0	1	1	1	0	1	1	1	0	1	1	1
0	0	1	0	1	0	0	1	1	1	1	1	1	1	0	1	1	1
0	0	1	0	1	0	0	1	1	0	1	1	1	1	0	1	1	1
0	0	1	0	1	0	0	1	1	0	1	1	1	1	0	1	1	1
0	0	1	0	1	0	0	1	1	0	1	1	1	1	0	1	1	1
0	0	1	0	1	0	0	1	1	1	1	1	1	1	0	1	1	1
0	0	1	0	1	0	0	1	1	0	0	1	1	1	1	1	1	1
0	0	1	0	1	0	1	1	1	0	1	1	1	1	0	1	1	1
0	0	1	0	1	0	0	1	1	0	1	1	1	1	0	1	1	1
0	0	1	0	1	0	1	1	1	0	1	1	1	1	0	1	1	1
0	0	1	1	1	0	0	1	1	0	0	1	1	1	0	1	1	1
0	0	1	0	1	0	0	1	1	0	0	1	1	1	0	1	1	1
0	0	1	0	1	0	0	1	1	0	0	1	1	1	0	1	1	1
0	0	1	0	1	0	1	1	1	0	0	1	1	1	0	1	1	1
0	0	1	1	1	0	0	1	1	0	0	1	1	1	0	1	1	1
0	0	1	0	1	0	0	1	1	0	0	1	1	1	0	1	1	1
0	0	1	0	1	0	0	1	1	0	1	1	1	1	0	1	1	1
0	0	1	1	1	0	0	1	1	0	0	1	1	1	0	1	1	1
0	0	1	0	1	0	1	1	1	0	0	1	1	1	0	1	1	1
0	0	0	0	0	0	0	0	0	0	0	0	0	0	0	0	0	0
0	0	1	0	1	0	0	1	1	0	0	1	1	1	0	1	1	1
0	0	1	1	1	0	1	1	1	1	1	1	1	1	1	1	1	1
0	0	1	1	1	0	1	1	1	0	0	1	1	1	0	1	1	1
0	0	1	0	1	0	0	1	1	0	0	1	1	1	0	1	1	1
0	0	1	0	1	0	0	1	1	0	0	1	1	1	0	1	1	1
0	0	1	0	1	0	0	1	1	0	0	1	1	1	0	1	1	1

0	0	1	1	1	0	0	1	1	0	0	1	1	1	0	1	1	1
0	0	1	0	1	0	0	1	1	0	0	1	1	1	0	1	1	1
0	0	1	1	1	0	1	1	1	1	1	1	1	1	0	1	1	1
0	0	1	1	1	0	1	1	1	1	1	1	1	1	0	1	1	1
0	0	1	0	1	0	1	1	1	1	1	1	1	1	0	1	1	1
0	0	1	1	1	0	1	1	1	1	1	1	1	1	0	1	1	1
0	0	1	0	1	0	1	1	1	1	1	1	1	1	0	1	1	1
0	0	1	0	1	0	1	1	1	1	1	1	1	1	0	1	1	1
0	0	1	0	1	0	1	1	1	1	1	1	1	1	0	1	1	1
0	0	1	0	1	0	1	1	1	1	1	1	1	1	0	1	1	1
0	0	1	1	1	0	1	1	1	1	1	1	1	1	0	1	1	1
0	0	1	0	1	0	0	1	1	1	0	1	1	1	1	1	1	1
0	0	1	0	1	0	1	1	1	1	0	1	1	1	0	1	1	1
0	0	1	0	1	0	1	1	1	1	0	1	1	1	0	1	1	1
0	0	1	0	1	0	1	1	1	0	0	1	1	1	0	1	1	1
0	0	1	0	1	0	1	1	1	1	0	1	1	1	1	1	1	1
0	0	1	0	1	0	0	1	1	0	0	1	1	1	0	1	1	1
0	0	1	0	1	0	0	1	1	0	0	1	1	1	0	1	1	1
0	0	1	0	1	0	1	1	1	0	0	1	1	1	0	1	1	1
0	0	1	1	1	0	0	1	1	0	0	1	1	1	0	1	1	1
0	0	1	1	1	0	0	1	1	0	0	1	1	1	0	1	1	1
0	0	1	1	1	0	0	1	1	0	0	1	1	1	0	1	1	1
0	0	1	0	1	0	0	1	1	0	0	1	1	1	0	1	1	1
0	0	1	0	1	0	0	1	1	0	0	1	1	1	0	1	1	1
0	0	1	0	1	0	0	1	1	0	0	1	1	1	0	1	1	1
0	0	1	0	1	0	0	1	1	0	0	1	1	1	0	1	1	1
0	0	1	0	1	0	0	1	1	0	0	1	1	1	0	1	1	1
0	0	1	0	1	0	1	1	1	0	0	1	1	1	0	1	1	1
0	0	1	0	1	0	0	1	1	0	0	1	1	1	0	1	1	1
0	0	1	0	1	0	0	1	1	0	0	1	1	1	0	1	1	1
0	0	0	0	0	0	0	0	0	0	0	0	0	0	0	0	0	0
0	0	1	0	1	0	0	1	1	0	0	1	1	1	0	1	1	1
0	0	1	1	1	0	1	1	1	1	1	1	1	1	1	1	1	1
0	0	1	1	1	0	0	1	1	0	0	1	1	1	1	1	1	1
0	0	1	0	1	0	0	1	1	0	0	1	1	1	0	1	1	1
0	0	1	0	1	0	1	1	1	1	0	1	1	1	1	1	1	1
0	0	1	0	1	0	0	1	1	1	1	1	1	1	1	1	1	1
0	0	1	0	1	0	1	1	1	1	1	1	1	1	1	1	1	1
0	0	1	0	1	0	0	1	1	0	1	1	1	1	0	1	1	1
0	0	1	0	1	0	0	1	1	0	0	1	1	1	0	1	1	1
0	0	1	1	1	0	1	1	1	1	1	1	1	1	0	1	1	1
0	0	1	1	1	0	1	1	1	1	1	1	1	1	0	1	1	1
0	0	1	0	1	0	1	1	1	1	1	1	1	1	0	1	1	1
0	0	1	1	1	0	1	1	1	1	1	1	1	1	0	1	1	1
0	0	1	0	1	0	0	1	1	0	0	1	1	1	0	1	1	1
0	0	1	0	1	0	1	1	1	1	1	1	1	1	0	1	1	1
0	0	1	0	1	0	1	1	1	1	1	1	1	1	0	1	1	1
0	0	1	0	1	0	1	1	1	1	1	1	1	1	0	1	1	1
0	0	1	0	1	0	1	1	1	1	1	1	1	1	0	1	1	1
0	0	1	1	1	0	1	1	1	1	1	1	1	1	0	1	1	1
0	0	1	1	1	0	1	1	1	1	1	1	1	1	0	1	1	1
0	0	1	1	1	0	1	1	1	1	1	1	1	1	0	1	1	1
0	0	1	0	1	0	0	1	1	0	0	1	1	1	1	1	1	1
0	0	1	1	1	0	0	1	1	0	0	1	1	1	0	1	1	1
0	0	1	0	1	0	0	1	1	0	0	1	1	1	0	1	1	1
0	0	1	0	1	0	0	1	1	0	0	1	1	1	0	1	1	1
0	0	1	0	1	0	0	1	1	0	0	1	1	1	0	1	1	1
0	0	1	1	1	0	0	1	1	0	0	1	1	1	0	1	1	1
0	0	1	1	1	0	0	1	1	0	0	1	1	1	0	1	1	1
0	0	1	0	1	0	0	1	1	0	0	1	1	1	0	1	1	1
0	0	1	1	1	0	0	1	1	0	0	1	1	1	0	1	1	1
0	0	1	1	1	0	0	1	1	0	0	1	1	1	0	1	1	1
0	0	1	0	1	0	0	1	1	0	0	1	1	1	0	1	1	1
0	0	1	0	1	0	0	1	1	0	0	1	1	1	0	1	1	1
0	0	1	0	1	0	0	1	1	0	0	1	1	1	0	1	1	1
0	0	1	0	1	0	0	1	1	0	0	1	1	1	0	1	1	1
0	0	1	0	1	0	0	1	1	1	0	1	1	1	0	1	1	1
0	0	1	1	1	0	0	1	1	0	0	1	1	1	0	1	1	1
0	0	1	0	1	0	0	1	1	0	0	1	1	1	0	1	1	1

0	0	1	0	1	0	0	1	1	0	0	1	1	1	0	1	1	1
0	0	1	0	1	0	0	1	1	0	0	1	1	1	0	1	1	1
0	0	1	0	1	0	0	1	1	0	0	1	1	1	0	1	1	1
0	0	1	0	1	0	0	1	1	0	0	1	1	1	0	1	1	1
0	0	1	1	1	0	1	1	1	1	1	1	1	1	1	1	1	1
0	0	0	1	1	0	0	1	1	1	0	1	1	1	1	1	1	1
0	0	1	1	1	0	1	1	1	1	1	1	1	1	1	1	1	1
0	0	1	1	1	0	1	1	1	1	1	1	1	1	1	1	1	1
0	0	1	0	1	0	1	1	1	1	1	1	1	1	0	1	1	1
0	0	1	1	1	0	1	1	1	1	1	1	1	1	1	1	1	1
0	0	1	0	1	0	0	1	1	0	0	1	1	1	0	1	1	1
0	0	1	1	1	0	1	1	1	1	1	1	1	1	1	1	1	1
0	0	1	1	1	0	1	1	1	1	1	1	1	1	1	1	1	1
0	0	1	1	1	0	1	1	1	1	1	1	1	1	1	1	1	1
0	0	1	1	1	0	1	1	1	1	1	1	1	1	1	1	1	1
0	0	1	0	1	0	1	1	1	1	1	1	1	1	1	1	1	1
0	0	1	0	1	0	1	1	1	1	1	1	1	1	0	1	1	1
0	0	1	0	1	0	1	1	1	1	1	1	1	1	0	1	1	1
0	0	1	0	1	0	0	1	1	0	0	1	1	1	0	1	1	1
0	0	1	0	1	0	0	1	1	0	0	1	1	1	0	1	1	1
0	0	1	0	1	0	0	1	1	0	0	1	1	1	0	1	1	1
0	0	1	0	1	0	0	1	1	0	0	1	1	1	0	1	1	1
0	0	1	1	1	0	0	1	1	0	0	1	1	1	0	1	1	1
0	0	1	0	1	0	1	1	1	1	1	1	1	1	1	1	1	1
0	0	1	0	1	0	0	1	1	1	1	1	1	1	1	1	1	1
0	0	1	0	1	0	1	1	1	1	1	1	1	1	1	1	1	1
0	0	1	0	1	0	1	1	1	1	1	1	1	1	0	1	1	1
0	0	1	0	1	0	1	1	1	1	1	1	1	1	1	1	1	1
0	0	1	0	1	0	1	1	1	0	1	1	1	1	0	1	1	1
0	0	1	0	1	0	0	1	1	0	0	1	1	1	0	1	1	1
0	0	1	1	1	0	0	1	1	0	0	1	1	1	0	1	1	1
0	0	1	0	1	0	0	1	1	0	0	1	1	1	0	1	1	1
0	0	1	0	1	0	0	1	1	0	0	1	1	1	0	1	1	1
0	0	1	0	1	0	0	1	1	0	0	1	1	1	0	1	1	1
0	0	1	0	1	0	0	1	1	0	0	1	1	1	0	1	1	1
0	0	1	0	1	0	0	1	1	0	0	1	1	1	0	1	1	1
0	0	1	0	1	0	0	1	1	0	0	1	1	1	0	1	1	1
0	0	1	0	1	0	0	1	1	0	0	1	1	1	0	1	1	1
0	0	1	0	1	0	0	1	1	0	0	1	1	1	0	1	1	1
0	0	1	0	1	0	0	1	1	0	0	1	1	1	0	1	1	1
0	0	1	0	1	0	0	1	1	0	0	1	1	1	0	1	1	1
