# Supplementary material for: Unexpected Novel Relational Links Uncovered by Extensive Developmental Profiling of Nuclear Receptor Expression
Source: PLoS Genet. 2007 Nov 9;3(11):e188. doi: 10.1371/journal.pgen.0030188 (PMC2065881; doi:10.1371/journal.pgen.0030188)
Supplement: Supplementary file 7 [file pgen.0030188.st004.pdf]

gene1	gene2	group	acceleration	p-value	Ka	Ks	Ka/Ks	Exp Div
COUPTFA	COUPTFX	II	6.1	1.0E-07	.079	1.351	.059	.407
EAR2-A	EAR2-B	II	2.1	6.4E-01	.085	1.038	.082	.759
ERβA	ERβB	III	2.3	6.7E-01	.323	2.102	.154	.934
ERRβ	ERRβγ	III	5.6	1.0E-07	.165	1.046	.158	.809
SF1B	SF1D	V	1.5	1.3E-01	.216	1.550	.140	.500
GCNF-A	GCNF-B	VI	7.1	1.0E-07	.238	1.260	.189	.937
PPARαA	PPARαB	I	2.9	4.2E-03	.193	1.140	.169	0.000
PPARβA	PPARβB	I	1.2	3.9E-01	.080	1.349	.059	.504
RARαA	RARαB	I	3.2	7.6E-01	.075	1.580	.047	.317
RARγA	RARγB	I	1.4	1.7E-01	.028	1.123	.025	.786
RevβA	RevβB	I	2.2	3.1E-02	.251	1.979	.127	.389
REVγA	REVγB	I	2.9	7.3E-01	.235	1.657	.142	.951
RORαA	RORαB	I	6.2	1.0E-07	.154	1.398	.110	.545
RORγA	RORγB	I	1.1	2.8E-01	2.240	999	999	.660
RXRαA	RXRαB	II	1.9	6.0E-02	.036	1.780	.020	.689
RXRβA	RXRβB	II	3.4	6.5E-04	.096	1.254	.077	0.000
SHP-A	SHP-B	O	4.2	2.9E-05	.522	999	999	1.000
TRαA	TRαB	I	1.4	4.7E-01	.031	.524	.060	.722
VDR-A	VDR-B	I	2.0	6.1E-01	.077	1.106	.070	.623

Bertrand et al., Sup Table 4
