# Supplementary material for: Unexpected Novel Relational Links Uncovered by Extensive Developmental Profiling of Nuclear Receptor Expression
Source: PLoS Genet. 2007 Nov 9;3(11):e188. doi: 10.1371/journal.pgen.0030188 (PMC2065881; doi:10.1371/journal.pgen.0030188)
Supplement: Supplementary file 8 [file pgen.0030188.st005.pdf]

STAGE	anatomical structure				abbreviation
Gastrula					G-tot
Gastrula	forerunner cells				G-forerunner
Gastrula	margin				G-marg
Gastrula	axis				G-marg-ax
Gastrula	axis	prechordale plate			G-preplate
Gastrula	paraxial mesoderm	segmental plate			G-mesoparax
Gastrula	Neural plate				G-neuplate
Gastrula	epidermis				G-epi
Gastrula	YSL				G-YSL
Gastrula	NR				G-NR
ES					ES-tot
ES	polster				ES-polster
ES	notochorde				ES-noto
ES	tailbud				ES-tail
ES	ventral mesoderm				ES-mesoV
ES	cephalic mesoderm				ES-mesoC
ES	segmental plate				ES-segplate
ES	segmental plate	somites			ES-som
ES	segmental plate	adaxial cells			ES-adaxialC
ES	Neural plate				ES-neuplate
ES	Neural plate	presomptive spinal chord			ES-spi
ES	Neural plate	Brain			ES-B
ES	Neural plate	Brain	forebrain		ES-B-for
ES	Neural plate	Brain	forebrain	telencephalon	ES-B-for-tel
ES	Neural plate	Brain	forebrain	diencephalon	ES-B-for-di
ES	Neural plate	Brain	midbrain		ES-B-MB
ES	Neural plate	Brain	Hindbrain		ES-B-HB
ES	eye field				ES-eye
ES	Floor plate				ES-floorplate
ES	neural crest				ES-NCC
ES	epidermis				ES-epi
ES	Kupffer's vesicle				ES-KUP
ES	YSL				ES-YSL
ES	NR				ES-NR
MS					MS-tot
MS	notochorde				MS-noto
MS	hematopoietic mesoderm				MS-mesohemato
MS	paraxial mesoderm				MS-mesoparax
MS	paraxial mesoderm	unsegmented			MS-mesoparax-unseg
MS	paraxial mesoderm	somites			MS-som
MS	paraxial mesoderm	adaxial cells			MS-adaxialC
MS	ventral mesenchyme				MS-mesenV
MS	retina/eye				MS-retina
MS	Brain				MS-B
MS	Brain	forebrain			MS-B-FB
MS	Brain	forebrain	diencephalon		MS-B-FB-di
MS	Brain	midbrain			MS-B-MB
MS	Brain	Hindbrain			MS-B-HB
MS	Brain	cranial ganglia			MS-crangang
MS	Spinal chord				MS-spi
MS	Spinal chord	anterior spinal chord			MS-spi-ant
MS	Spinal chord	posterior spinal chord			MS-spi-post
MS	epiphysis				MS-epiphy
MS	tailbud				MS-tail
MS	neural crest				MS-NCC
MS	branchial arches				MS-branch
MS	pronephric ducts				MS-product
MS	endoderm	intestine			MS-endo
MS	epidermis				MS-epi
MS	mucous cells				MS-mucousC
MS	hatching gland				MS-hatchgland
MS	YSL				MS-YSL
MS	NR				MS-NR
24-h					24-tot
24-h	nose				24-nose
24-h	otic vesicle				24-oticves
24-h	lateral line				24-latline
24-h	head mesenchyme				24-mesenhead
24-h	epiphysis				24-epiphy
24-h	adenohypophysis				24-adenohypo
24-h	Brain				24-B
24-h	Brain	forebrain			24-B-FB
24-h	Brain	forebrain	telencephalon		24-B-FB-tel
24-h	Brain	forebrain	diencephalon		24-B-FB-di
24-h	Brain	midbrain			24-B-MB
24-h	Brain	midbrain	tegmentum		24-B-MB-antteg
24-h	Brain	midbrain	tectum		24-B-MB-tec
24-h	Brain	MHB			24-B-MHB
24-h	Brain	Hindbrain			24-B-HB
24-h	cranial ganglia				24-B-crangang
24-h	Spinal chord				24-spi
24-h	Spinal chord	anterior spinal chord			24-spi-ant
24-h	branchial arches				24-branch
24-h	retina				24-retina
24-h	lens				24-lens
24-h	tailbud				24-tail
24-h	somites				24-som
24-h	slow muscle fibers				24-slowmusclefib
24-h	mucous cells				24-mucousC
24-h	pronephric ducts				24-product
24-h	interenal tissue				24-interrenaltissue
24-h	intestinal bulb				24-intesbulb
24-h	intestin				24-intestin
24-h	pancrea primordium				24-pancrea

24-h	pharyngeal pouches				24-pharpouch
24-h	notochorde				24-postnoto
24-h	epidermis				24-epidermis
24-h	hatching gland				24-hatchgland
24-h	YSL				24-YSL
24-h	NR				24-NR
36-h					36-tot
36-h	nose				36-nose
36-h	otic vesicle				36-oticvesicle
36-h	retina				36-retina
36-h	retina	proliferative zone			36-retina-prolifzone
36-h	retina	non proliferative zone			36-retina-nonprolifzone
36-h	lens				36-lens
36-h	lateral line				36-latline
36-h	Brain				36-B
36-h	Brain	forebrain			36-B-FB
36-h	Brain	forebrain	telencephalon		36-B-FB-tel
36-h	Brain	forebrain	diencephalon		36-B-FB-di
36-h	Brain	forebrain	diencephalon	habenula (diencephalon)	36-FB-di-habenula
36-h	Brain	midbrain			36-B-MB
36-h	Brain	midbrain	tegmentum		36-B-MB-teg
36-h	Brain	midbrain	tectum		36-B-tec
36-h	Brain	MHB			36-B-MHB
36-h	Brain	hindbrain			36-B-HB
36-h	Spinal chord				36-spi
36-h	Spinal chord	anterior spinal chord			36-spi-ant
36-h	cranial ganglia				36-crangang
36-h	epiphysis				36-epiphy
36-h	hypophysis				36-hypophy
36-h	head mesenchyme				36-mesenhead
36-h	branchial arches				36-branch
36-h	pectoral fin bud				36-pecfinbud
36-h	heart				36-heart
36-h	somites				36-som
36-h	slow muscle fibers				36-slowmusclefi
36-h	pronephric ducts				36-product
36-h	interenal tissue				36-interrenaltissue
36-h	mucous cells				36-mucousC
36-h	pancrea primordium				36-pancrea
36-h	intestine				36-intestine
36-h	intestinal bulb				36-intesbulb
36-h	liver				36-liver
36-h	proctodeum				36-proctodeum
36-h	pharyngeal pouches				36-pharpouch
36-h	epidermis				36-epi
36-h	hatching gland				36-hatchgland
36-h	NR				36-NR
48-h					48-tot
48-h	nose				48-nose
48-h	Brain				48-B
48-h	Brain	forebrain			48-B-FB
48-h	Brain		telencephalon		48-B-FB-tel
48-h	Brain		diencephalon		48-B-FB-di
48-h	Brain			habenula (diencephalon)	48-B-FB-di-habenula
48-h	Brain	midbrain			48-B-MB
48-h	Brain		tegmentum		48-B-Mbantteg
48-h	Brain		tectum		48-B-MB-tec
48-h	Brain	Hindbrain			48-B-HB
48-h	Brain	MHB			48-B-MHB
48-h	Spinal chord				48-spi
48-h	Spinal chord	anterior spinal chord			48-spi-ant
48-h	cranial ganglia				48-B-crangangl
48-h	otic vesicle				48-oticvesicle
48-h	hypophysis				48-hypophy
48-h	epiphysis				48-epiphy
48-h	branchial arches				48-branch
48-h	retina				48-retina
48-h	retina	proliferative zone			48-retina-prolifzone
48-h	retina	non proliferative zone			48-retina-nonprolifzone
48-h	retina	inner nuclear layer			48-retina-inner
48-h	retina	ganglion cell layer			48-retina-ganglion
48-h	retina	photoreceptor cell layer			48-retina-photo
48-h	lateral line neuromasts				48-latlineneuro
48-h	pectoral fin				48-pecfin
48-h	pronephric ducts				48-product
48-h	interenal tissue				48-interrenaltissue
48-h	heart				48-heart
48-h	liver				48-liver
48-h	pancrea				48-pancrea
48-h	intestin				48-intestine
48-h	intestinal bulb				48-intesbulb
48-h	pharyngeal endoderm				48-pharendo
48-h	epidermis				48-epi
48-h	NR				48-NR
