# Supplementary material for: Unexpected Novel Relational Links Uncovered by Extensive Developmental Profiling of Nuclear Receptor Expression
Source: PLoS Genet. 2007 Nov 9;3(11):e188. doi: 10.1371/journal.pgen.0030188 (PMC2065881; doi:10.1371/journal.pgen.0030188)
Supplement: Supplementary file 9 [file pgen.0030188.sd001.doc]

Text S1
Detailed Description of Expression Patterns Shown in Figure S3 and Figure S4

Supplementary Figure 3 - 1 : Expression of  TRα-A (NR1A1-A)
Expression of this gene is not spatially restricted during embryonic and larval stages
(a) embryo at gastrula stage,  (b) at early somitogenesis, (c) at middle of somitogenesis, (d) at 24 hpf, (e) at 36 hpf, (f) at 48 hpf and (g) at 5 dpf.

Supplementary Figure 3 - 2 : Expression of  TRα-B (NR1A1-B)
Expression of this gene is not spatially restricted during embryonic and larval stages
(a) embryo at gastrula stage,  (b) at early somitogenesis, (c) at middle of somitogenesis, (d) at 24 hpf, (e) at 36 hpf, (f) at 48 hpf and (g) at 5 dpf.

Supplementary Figure 3 - 3 : Expression of  TRβ (NR1A2)
(a) embryo at 24 hpf in lateral view and (b) in dorsal. (c) embryo at 36 hpf in lateral view and (d) in dorsal view showing expression in the non proliferative part of the retina. (e) embryo at 48 hpf in lateral view and (f) in dorsal view, showing expression in retina and a weak expression in midbrain and hindbrain.

Supplementary Figure 3 - 4 : Expression of  RARα-A (NR1B1-A)
(a - e) embryo at early somitogenesis in lateral view (a) and dorsal views (b - e) from anterior to posterior. (f - k) embryo at the middle of somitogenesis. (f - h) lateral views (i - k) dorsal views. In (h) the yolk has been dissected off. (l - n) embryo at 24 hpf in lateral view (l) and dorsal views (m - n). (o - q) embryo at 36 hpf in lateral view (o) and dorsal views (p - q). (r - s) embryo at 48 hpf in lateral view (r) and dorsal view (s). There is no detectable expression at gastrula stage.

Supplementary Figure 3 - 5 : Expression of  RARá-B (NR1B1-B)
(a - c) embryo at gastrula stage in lateral view (a, b) and dorsal view (c). (d - g) embryo at early somitogenesis in lateral view (d) and dorsal view from anterior to posterior (e - g). (h - o) embryo at the middle of somitogenesis in lateral view (h, i) and in dorsal view (j - o). (m) high magnification of the hindbrain expression at the level of the otic vesicle. (p) embryo at 24 hpf and (q) at 36 hpf in lateral view. (r - s) embryo at 36 hpf in dorsal view at the level of the head. (t - v) embryo at 48 hpf in lateral view (t, u) and in dorsal view (v) at the level of the head.

Supplementary Figure 3 - 6 : Expression of  RARγ-A (NR1B3-A)
(a, b) embryo at gastrula stage in lateral view (a) and in vegetal pole view dorsal to the left. (c - f) embryo at early somitogenesis in dorsal views from anterior to posterior. (g, h) embryo at the middle of somitogenesis in lateral view (g) and dorsal view of the head region (h). (i - k) embryo at 24 hpf in lateral view (i) and dorsal view of the head region (j, k). (l - n) embryo at 36 hpf in lateral view (l) and dorsal view of the head region (m, n). (o) lateral view (o) and dorsal view (p) at 48 hpf.

Supplementary Figure 3 - 7 : Expression of  RARγ-B (NR1B3-B)
(a, b) Embryo at early somitogensis in lateral view (a) and dorsal view of the tail bud region (b). (c - e) embryo at middle of somitogenesis in lateral view (c), dorsal view of the hindbrain region (d) and in optical cross section at the level of the hindbrain  (e). (f - h) embryo at 24 hpf in lateral view (f, g) and dorsal view (h). (i- k) embryo at 36 hpf in dorsal view (i), frontal view dorsal to the right (j) and lateral view (k). (l - o) Embryo at 48 hpf in lateral view (l) dorsal view (m, n) and frontal view with dorsal to the right (o). There is no detectable expression at gastrula stage.

Supplementary Figure 3 - 8 : Expression of  PPARα-A (NR1C1-A)
There is no detectable expression of this gene during embryonic and larval stages. (a) embryo at gastrula stage,  (b) at early somitogenesis, (c) at middle of somitogenesis, (d) at 24 hpf, (e) at 36 hpf, (f) at 48 hpf and (g) at 5 dpf.

Supplementary Figure 3 - 9 : Expression of  PPARα-B (NR1C1-B)
There is no detectable expression of this gene during embryonic and larval stages. (a) embryo at gastrula stage,  (b) at early somitogenesis, (c) at middle of somitogenesis, (d) at 24 hpf, (e) at 36 hpf, (f) at 48 hpf and (g) at 5 dpf.

Supplementary Figure 3 - 10 : Expression of  PPARâ-A (NR1C2-A)
(a) embryo at 24 hpf and (b) at 36 hpf showing a basal level of expression in the whole embryo. At 48 hpf in addition to this general expression (c) a specific expression in liver is observed (d). (e - f) embryo at 5 dpf in lateral view (e) and in oblique view (f). There is no detectable expression before 24 hpf.

Supplementary Figure 3 - 11 : Expression of  PPARβ-B (NR1C2-B)
Expression of this gene is not spatially restricted during embryonic and larval stages. (a) embryo at gastrula stage,  (b) at early somitogenesis, (c) at middle of somitogenesis, (d) at 24 hpf, (e) at 36 hpf, (f) at 48 hpf and (g) at 5 dpf.

Supplementary Figure 3 - 12 : Expression of  PPARγ (NR1C3)
Expression of this gene is not spatially restricted during embryonic stages
(a) embryo at gastrula stage,  (b) at early somitogenesis, (c) at middle of somitogenesis, (d) at 24 hpf, (e) at 36 hpf, (f) at 48 hpf and (g) at 5 dpf. (g) embryo at 5 dpf showing the expression of PPARg in endodermal derivatives, liver, intestine and swim bladder.

Supplementary Figure 3 - 13 : Expression of  Reverbα (NR1D1)
This gene is not expressed before 24 hpf when it is first detected in the epiphysis (not shown). There is no expression at 36 hpf. (a - b) embryo at 48 hpf in lateral and dorsal view. (c - d) embryo at 5 dpf in lateral and dorsal view.

Supplementary Figure 3 - 14 : Expression of  Reverbβ-A (NR1D2-A)
This gene is not expressed before 48 hpf (a - b) in lateral and dorsal view. (c - f) embryo at 5 dpf in lateral view (c, e) and in dorsal view (d, f).

Supplementary Figure 3 - 15 : Expression of  Reverbβ-B (NR1D2-B)
This gene is not expressed before 24 hpf (a - d) in lateral view (a, b) and frontal view (d). There is no expression at 36 hpf. (e, f) embryo at 48 hpf in lateral view and dorsal view. (g - i) embryo at 5 dpf in dorsal view (g) and lateral view (h, i).

Supplementary Figure 3 - 16 : Expression of  Reverbγ-A (NR1D4-A)
Expression of this gene is not spatially restricted at gastrula (a), early somitogensis (b), middle of somitogenesis (c), 24 hpf (d), 36 hpf (e). At 48 hpf in addition to a general basal level of expression, specific labelling is observed in lateral part of the hindbrain and in retina. (f, i) dorsal view, (g) lateral view and (h) ventral view.

Supplementary Figure 3 - 17 : Expression of  Reverbγ-B (NR1D4-A)
This gene is not expressed before 36 hpf. At this stage (a - c) expression is observed in lateral part of the hindbrain (a) lateral view, (b, c) dorsal view. (d - f) 48 hpf in lateral view (d) and dorsal view (e, f).

Supplementary Figure 3 - 18 : Expression of  RORá-A (NR1F1-A)
This gene is not expressed before the middle of somitogenesis. (a, b) middle of somitogenesis in lateral view (a) and dorsal view of the head region (b). (c, d) embryo at 24 hpf in lateral view (c) and dorsal view of the head region (d). (e, f) embryo at 36 hpf in lateral view (e) and dorsal view of the head region (f). (g, h) embryo at 48 hpf in lateral view (g) and dorsal view of the head region (h). (i, j) embryo at 5 dpf in lateral view (i) and dorsal view of the head region (j).

Supplementary Figure 3 - 19 : Expression of  RORá-B (NR1F1-B)
This gene is not expressed before 36 hpf. (a, c) embryo at 36 hpf in lateral view (a, b) and in dorsal view of the head region (c). (d - f) embryo at 48 hpf in lateral view (d, e) and dorsal view of the head region (f). (g) lateral view and (h) dorsal view of the head region at 5 dpf.

Supplementary Figure 3 - 20 : Expression of  RORâ (NR1F2)
This gene is not expressed before the middle of somitogenesis. (a, b) embryo at the middle of somitogenesis in lateral view (a) and dorsal view o fthe head region (b). (c, d) embryo at 24 hpf in lateral view (c) and in a dorsal view at the level of the head region (d). (e, f) embryo at 36 hpf in lateral view (e) and dorsal view of the head region (f). (g, h) embryo at 48 hpf in lateral view (g) and in dorsal view of the head region (h). (i - k) embryo at 5 dpf in lateral view (i) and in dorsal view of the head region (j, k).

Supplementary Figure 3 - 21 : Expression of  RORγ-A (NR1F3-A)
This gene is not expressed at gastrula stage. (a, b) embryo at early somitogenesis in lateral view (a) and in dorsal view (b). (c, d) embryo at the middle of somitogenesis in lateral view (c) and dorsal view of the head region (d). (e) embryo at 24 hpf in lateral view. (f, g) embryo at 36 hpf in lateral view (f) and dorsal view of the head region (g). (h, i) embryo at 48 hpf in lateral view (h) and in dorsal view of head region (i). (j) embryo at 5 dpf in lateral view (j) and in dorsal view of the head region (k).

Supplementary Figure 3 - 22 : Expression of  RORγ-B (NR1F3-B)
This gene is not expressed before the middle of somitogenesis. (a- c) embryo at the middle of somitogenesis in lateral view (a) and dorsal view of the head region (b) and of trunk region (c). (d) embryo at 24 hpf in lateral view. (e - j) embryo at 36 hpf in lateral view (e-h) and in dorsal view (i, j). (k, l) embryo at 48 hpf in lateral view (k) and dorsal view of the head region. (m, n) embryo at 5 dpf in lateral view (m) and dorsal view of the head region (n).

Supplementary Figure 3 - 23 : Expression of  LXRá (NR1H3)
There is no detectable expression of this gene during embryonic and larval stages. (a) embryo at gastrula stage,  (b) at early somitogenesis, (c) at middle of somitogenesis, (d) at 24 hpf, (e) at 36 hpf, (f) at 48 hpf and (g) at 5 dpf.

Supplementary Figure 3 - 24 : Expression of  FXRá (NR1H4)
There is no detectable expression of this gene until 48 hpf. (a, b) embryo at 48 hpf in lateral view (a) and dorsal view (b).

Supplementary Figure 3 - 25 : Expression of  FXRâ (NR1H5)
There is no detectable expression of this gene until 24 hpf. (a) embryo at gastrula stage, (b) embryo at early somitogenesis, (c) embryo at the middle of somitogenesis, (d) embryo at 24 hpf, (e) embryo at 36 hpf in lateral view. (f - h) embryo at 36 hpf in dorsal views.

Supplementary Figure 3 - 26 : Expression of  VDR-A (NR1I1-A)
Expression of this gene is not spatially restricted during embryonic and larval stages. (a) embryo at gastrula stage,  (b) at early somitogenesis, (c) at middle of somitogenesis, (d) at 24 hpf, (e) at 36 hpf, (f) at 48 hpf and (g) at 5 dpf.

Supplementary Figure 3 - 27 : Expression of  VDR-B (NR1I1-B)
There is no detectable expression of this gene until the middle of somitogenesis. (a - c) embryo at the middle of somitogenesis in lateral view (a) and in dorsal view of the trunk (b) and of the head region (c). (d, f) embryo at 24 hpf in lateral view (d) oblique view (e) and in dorsal view of the trunk region (f). (g - i) embryo at 36 hpf in oblique view (g) and in dorsal views (h, i). (j - l) embryo at 48 hpf in lateral view of the trunk region (j) of the head (k) and dorsal view of the head region (l).

Supplementary Figure 3 - 28 : Expression of  PXR (NR1I2)
There is no detectable expression of this gene until 24 hpf. (a - f) embryo at 24 hpf in lateral view (a, b). High magnification of the anterior part of the head (c, d). frontal view (e, f). (g - l) embryo at 36 hpf in lateral view (g - i). High magnification of the head (j, k) and in dosral view (l). (m - v) embryo at 48 hpf in lateral view (m, o - r) and in dorsal view (n, s, t) and in ventral view (u, v).

Supplementary Figure 3 - 29 : Expression of  HNF4á (NR2A1)
(a, b) embryo at gastula stage in lateral view (a) and vegetal pole view (b). (c, d) embryo at early somitogenesis in lateral view (c). High magnification of the yolk in lateral view (d). (e, f) embryo at the middle of somitogenesis in lateral view (e) and in dorsal view (f). (g, h) embryo at 24 hpf in lateral view (g) and in dorsal view (h). (i, j) embryo at 36 hpf in lateral view (i) and in dorsal view (j). (k, l) embryo at 48 hpf in lateral view (k) and in dorsal view (l). (m, n) embryo at 5 dpf in lateral view (m) and in dorsal view (n)

Supplementary Figure 3 - 30 : Expression of  HNF4â (NR2A3)
(a) embryo at gastrula stage, (b) embryo at early somitogensis, (c) embryo a middle of somitogenesis and (d) at 24 hpf in lateral views. There is no detectable expression of this gene during embryonic and early larval stages after 24 hpf.

Supplementary Figure 3 - 31 : Expression of  HNF4γ (NR2A2)
Expression of this gene is not spatially restricted during embryonic and larval stages. (a) embryo at gastrula stage,  (b) at early somitogenesis, (c) at middle of somitogenesis, (d) at 24 hpf, (e) at 36 hpf, (f) at 48 hpf and (g) at 5 dpf.

Supplementary Figure 3 - 32 : Expression of  RXRá-A (NR2B1-A)
There is no detectable expression of this gene at gastrula stage (a) and early somitogenesis (b). (c) embryo at the middle of somitogenesis, (d) at 24 hpf and (e) at 36 hpf, in lateral views. (f, g) embryo at 36 hpf in dorsal (f) and oblique view (g). (h) embryo at 48 hpf in dorsal view.

Supplementary Figure 3 - 33 : Expression of  RXRá-B (NR2B1-B)
There is no detectable expression of this gene before the middle of somitogenesis. (a - d) embryo at the middle of somitogenesis in lateral view (a) and in dorsal view of the trunk (b) of the head (c) and of the tail bud (d). (e, f) embryo at 24 hpf in lateral view (e) and in dorsal view (f). (g, h) embryo at 36 hpf in lateral view (g) and in dorsal view (h). (i, j) embryo at 48 hpf in lateral view (i) and dorsal view (j). (k) embryo at 5 dpf in lateral view.

Supplementary Figure 3 - 34 : Expression of  RXRâ-A (NR2B2-A)
Expression of this gene is not spatially restricted during embryonic and larval stages. (a) embryo at gastrula stage,  (b) at early somitogenesis, (c) at middle of somitogenesis, (d) at 24 hpf, (e) at 36 hpf, (f) at 48 hpf and (g) at 5 dpf.

Supplementary Figure 3 - 35 : Expression of  RXRâ-B (NR2B2-B)
Expression of this gene is not spatially restricted during embryonic and larval stages. (a) embryo at gastrula stage,  (b) at early somitogenesis, (c) at middle of somitogenesis, (d) at 24 hpf, (e) at 36 hpf, (f) at 48 hpf and (g) at 5 dpf.

Supplementary Figure 3 - 36 : Expression of  RXRγ (NR2B3)
There is no detectable expression of this gene at gastrula stage. (a - d) embryo at early somitogenesis in lateral views (a, b) and dorsal views (c, d). (e, j) embryo at the middle of somitogenesis in lateral view (e, f), in transverse optical cross sections (g,h) in frontal views (i, j). (k - o) embryo at 24 hpf in lateral view (k - m), dorsal views (n, o). (p - v) embryo at 36 hpf in dorsal view (p, u, v) and lateral views (q-t). (w - ab) embryo at 48 hpf in lateral view (w - z) and in dorsal view (aa, ab).

Supplementary Figure 3 - 37 : Expression of  TR2 (NR2C1)
Expression of this gene is not spatially restricted during embryonic and larval stages. (a) embryo at gastrula stage,  (b) at early somitogenesis, (c) at middle of somitogenesis, (d) at 24 hpf, (e) at 36 hpf, (f) at 48 hpf and (g) at 5 dpf.

Supplementary Figure 3 - 38 : Expression of  TR4 (NR2C2)
Expression of this gene is not spatially restricted during embryonic and larval stages. (a, b) embryo at 24 hpf in lateral view (a) and in dorsal view (b). (c, d) embryo at 36 hpf in lateral view (c) and in dorsal view (d). (e, f) embryo at 48 hpf in lateral view (e) and dorsal view (f). (g, h) embryo at 5 dpf in lateral view (g) and in dorsal view (h).

Supplementary Figure 3 - 39 : Expression of  TLL (NR2D1)
There is no detectable expression of this gene at gastrula stage. (a, c) embryo at early somitogenesis in lateral view (a) and in dorsal view of the head region (b) and in transverse optical cross section of the forebrain (c) . (d - f) embryo at the middle of somitogensis in lateral view (d, g) in dorsal view (e, h) and in transverse optical cross section of the forebrain (f). (i, l) embryo at 24 hpf in lateral views (i, j), dorsal view (k) and in transverse optical cross section of the forebrain (l). (m - p) embryo at 36 hpf in lateral views (m, n) dorsal views (o) and in transverse optical cross section of the forebrain (p). (q - s) embryo at 48 hpf in lateral views (q, r) and in transverse optical cross section of the forebrain (s).

Supplementary Figure 3 - 40 : Expression of  PNR (NR2D2)
There is no detectable expression of this gene before the middle of somitogenesis. (a, b) embryo in lateral view (a) and in transverse optical cross-section at the level of the epiphysis (b). (c, d) embryo at 24 hpf in lateral view (c) and in transverse optical cross-section at the level of the epiphysis (d). (e - i) embryo at 36 hpf in lateral views (e, f), high magnification of the retina (g) in frontal view (h, i). (j - n) embryo at 48 hpf in lateral view (j, k) dorsal view (l, m), high magnification of the retina (in horizontal optical cross section, n). (o, q) embryo at 5 dpf in lateral view of the head (o) and of the eye (p). (q) is a high magnification of the eye.

Supplementary Figure 3 - 41 : Expression of  COUPTFá-A (NR2F1-A)
There is no detectable expression of this gene at gastrula stage. (a, b) embryo at early somitogenesis in dorsal view of the trunk (a) and of the head region (b). (c - e) embryo at the middle of somitogenesis in lateral view (c) and in dorsal view of the head region (d) or the trunk (e). (f - j) embryo at 24 hpf in lateral  view (f, g) and dorsal views of the head region (h - j). (k - m) embryo at 36 hpf in lateral view (k), dorsal view (l) and oblique view (m). (n, o) embryo at 48 hpf in lateral view (n) and dorsal view (o) of the head. (p) embryo at 5 dpf in lateral view.

Supplementary Figure 3 - 42 : Expression of  COUPTFá-B (NR2F1-B)
There is no detectable expression of this gene at gastrula stage. (a, b) embryo at the 10 somite stage in lateral view (a) and in dorsal view at the level of the head (b). (c - e) embryo at the middle of somitogenesis in lateral view (c) and in dorsal view of the trunk (d) and of the head (e). (f - i) embryo at 24 hpf in lateral view (f, g), dorsal view (h) and in oblique view (i). (j - q) embryo at 36 hpf in lateral veiw (j, k, m - o), in dorsal view (l, p) and in oblique view (m, q). (r - t) embryo at 48 hpf in lateral view (r, s) and dorsal view (t).

Supplementary Figure 3 - 43 : Expression of  COUPTFâ (NR2F2)
There is no detectable expression of this gene at gastrula stage. (a, b) embryo at early somitogenesis in lateral view (a) and dorsal view of the head (b) and of the trunk (c). (d - f) embryo at the middle of somitogenesis in lateral view (d) and in dorsal view of the forebrain (e) and of the hindbrain (f). (g - j) embryo at 24 hpf in lateral views (g, h) and in dorsal views of the head region (i, j). (k - n) embryo at 36 hpf in lateral views (k, l) and dorsal views of the head region (m, n). (o, p) embryo at 48 hpf in lateral view. (q - s) embryo at 5 dpf in lateral view (q, r) and dorsal view of the head region (s).

Supplementary Figure 3 - 44 : Expression of  COUPTFγ (NR2F5)
There is no detectable expression of this gene at gastrula stage. (a) embryo in dorsal view in the anterior head region (anterior to the top) at the bud stage. (b, e) embryo at early somitogenesis, in lateral view (b) and in dorsal views (c - e) from trunk to head. (f - j) embryo at the middle of somitogenesis in lateral view (f) and in dorsal views (g - j) from posterior to anterior. (k - r) expression at 24 hpf in lateral views (k - n), dorsal views (o, p), frontal view (q) and oblique view (r). (s, t) embryo at 36 hpf in lateral views.

Supplementary Figure 3 - 45 : Expression of  EAR2-A (NR2F6-A)
Expression of this gene is not spatially restricted at gastrula (a) and early somitogenesis (b). (c) embryo  at middle of somitogenesis in lateral view. (d) embryo at 24 hpf, (e) embryo at 36 hpf, (f) embryo at 48 hpf in lateral views. (g) embryo at 48 hpf in dorsal view of the head.

Supplementary Figure 3 - 46 : Expression of  EAR2-B (NR2F6-B)
(a, b) embryo at gastrula stage in dorsal view, (c - f) embryo at early somitogenesis in lateral view (c) and  in dorsal view of the posterior part (d), of the head region (e) and of the trunk (f). (g - i) embryo at the middle of somitogenesis in lateral view (g) and in dorsal view of the trunk (h) and of the head (i). (j - l) embryo at 24 hpf in lateral views. (m - p) embryo at 36 hpf in lateral views (m , n) and in dorsal views (o, p). (q) embryo at 48 hpf in lateral view. (r - u) embryo at 5 dpf in lateral views (r - t) and in dorsal view (u).

Supplementary Figure 3 - 47 : Expression of  ERá (NR3A1)
There is no detectable expression of this gene during embryonic and larval stages. (a) embryo at gastrula stage,  (b) at early somitogenesis, (c) at middle of somitogenesis, (d) at 24 hpf, (e) at 36 hpf, (f) at 48 hpf and (g) at 5 dpf.

Supplementary Figure 3 - 48 : Expression of  ERâ-A (NR3A2-A)
There is no detectable expression of this gene before 24 hpf. (a, b) embryo at 24 hpf in lateral view (a) and dorsal view of the trunk (b). (c - g) embryo at 36 hpf in lateral views (c - e) and dorsal views (f, g). (h - n) embryo at 48 hpf in lateral views (h - j) and in dorsal views (k - n).

Supplementary Figure 3 - 49 : Expression of  ERâ-B (NR3A2-B)
Expression of this gene is not spatially restricted during embryonic and larval stages. (a) embryo at gastrula stage,  (b) at early somitogenesis, (c) at middle of somitogenesis, (d) at 24 hpf, (e) at 36 hpf, (f) at 48 hpf and (g) at 5 dpf.

Supplementary Figure 3 - 50 : Expression of  ERRá (NR3B1)
(a, b) embryo at gastrula stage in lateral view (a) and in vegetal pole view (b). (c - e) embryo at early somitogenesis in lateral view (c) and in dorsal view of the posterior part (d) and of the trunk region (e). (f - i) embryo at the middle of somitogenesis in lateral view (f) and in dorsal views (g - i). (j - m) embryo at 24 hpf in lateral views (j - l) and in dorsal view (m). (n - p) embryo at 36 hpf in lateral views. (q - s) embryo at 48 hpf in lateral view (q) and dorsal views of the head (r, s).

Supplementary Figure 3 - 51 : Expression of  ERRâ (NR3B2)
There is no detectable expression of this gene before the middle of somitogenesis. (a - c) embryo at the middle of somitogenesis in lateral view (a) and in dorsal views (b, c). (d - f) embryo at 24 hpf  in lateral views. (g - m) embryo at 36 hpf in lateral views (g - i) and in dorsal views (j - m). (n, o) embryo at 48 hpf in lateral view (n) and dorsal view (o).

Supplementary Figure 3 - 52 : Expression of  ERRγ (NR3B3)
There is no detectable expression of this gene at gastrula stage. (a, b) embryo at early somitogenesis in lateral view (a) and in dorsal view of the tail bud (b). (c - e) embryo at the middle of somitogenesis in lateral views (c, d) and in dorsal view of the posterior part (e). (f - m) embryo at 24 hpf in lateral views (f - i), and in dorsal views (j - m). (n - t) embryo at 36 hpf in lateral views (n - q) dorsal views (r, s) and frontal view (t). (u - w) embryo at 48 hpf in lateral view. (w) high magnification on the trunk region. (x - z) embryo at 48 hpf in dorsal views of the head (x, y) and in lateral view (z). (aa) embryo at 5 dpf in dorsal view.

Supplementary Figure 3 - 53 : Expression of  ERRδ (NR3B4)
There is no detectable expression of this gene before the middle of somitogenesis. (a, b) embryo at the middle of somitogensis in lateral view (a) and in dorsal view (b). (c - f) embryo at 24hpf in lateral views (c - e) and in dorsal view (f). (g - j) embryo at 36 hpf in lateral view (g, h) and in dorsal view (i, j). (k - m) embryo at 48 hpf in lateral views (k, l) and in dorsal view (m).

Supplementary Figure 3 - 54 : Expression of  ERRâ/g (NR3B2/3)
Expression of this gene is not spatially restricted during embryonic and larval stages. (a) embryo at gastrula stage,  (b) at early somitogenesis, (c) at middle of somitogenesis, (d) at 24 hpf, (e) at 36 hpf, (f) at 48 hpf and (g) at 5 dpf.

Supplementary Figure 3 - 55 : Expression of  GR (NR3C1)
Expression of this gene is not spatially restricted during embryonic stages. (a) embryo at gastrula stage,  (b) at early somitogenesis, (c) at middle of somitogenesis, (d) at 24 hpf, (e) at 36 hpf, (f) at 48 hpf. (g) embryo at 5 dpf.

Supplementary Figure 3 - 56 : Expression of  MR (NR3C2)
There is no detectable expression of this gene during embryonic and larval stages. (a) embryo at gastrula stage,  (b) at early somitogenesis, (c) at middle of somitogenesis, (d) at 24 hpf, (e) at 36 hpf, (f) at 48 hpf and (g) at 5 dpf.

Supplementary Figure 3 - 57 : Expression of  PR (NR3C3)
Expression of this gene is not spatially restricted during embryonic and larval stages. (a) embryo at gastrula stage,  (b) at early somitogenesis, (c) at middle of somitogenesis, (d) at 24 hpf, (e) at 36 hpf, (f) at 48 hpf and (g) at 5 dpf.

Supplementary Figure 3 - 58 : Expression of  AR (NR3C4)
There is no detectable expression of this gene during embryonic and larval stages. (a) embryo at gastrula stage,  (b) at early somitogenesis, (c) at middle of somitogenesis, (d) at 24 hpf, (e) at 36 hpf, (f) at 48 hpf and (g) at 5 dpf.

Supplementary Figure 3 - 59 : Expression of  NGFIB (NR4A1)
There is no detectable expression of this gene before 24 hpf. (a, b) embryo at 24 hpf in lateral view (a) and in dorsal view (b). (c - d) embryo at 36 hpf in lateral view (c) and dorsal view (d). (e - g) embryo at 48 hpf in lateral view (e) and in dorsal view (j, g). (h, i) embryo at 5 dpf in lateral view (h) and in dorsal view (i).

Supplementary Figure 3 - 60 : Expression of  NURR1 (NR4A2)
There is no detectable expression of this gene before 24 hpf. (a, c) embryo at 24 hpf in lateral view (a, b) and in dorsal view of the head (c). (d - o) embryo at 36 hpf in lateral views (d, f - i, n), in dorsal views (j - m) and in frontal view (o). (p - z) embryo at 48 hpf in lateral views (p - r) and in dorsal views (s - z). (v) is a high magnification of the eye. (aa - ad) embryo at 5 dpf in lateral views (aa, ab) and dorsal views (ac, ad).

Supplementary Figure 3 - 61 : Expression of  NOR1 (NR4A3)
There is no detectable expression of this gene before 24 hpf. (a, d) embryo at 24 hpf in lateral view (a b) and in dorsal view of the head (c, d). (e - g) embryo at 36 hpf in lateral view (e) dorsal view (f) and front view dorsal to the right (g). (h - j) embryo at 48 hpf in lateral views (h, i) and in dorsal view (j).

Supplementary Figure 3 - 62 : Expression of  SF1-A (NR5A1-A)
There is no detectable expression of this gene before 24 hpf. (a, b) embryo at 24 hpf in lateral view (a) and in dorsal view (b). (c - f) embryo at 36 hpf in lateral view (c), dorsal view (d, e) and frontal view (f). (g - j) embryo at 48 hpf in lateral views (g, h) and dorsal views (i, j). 

Supplementary Figure 3 - 63 : Expression of  SF1-B (NR5A1-B)
There is no detectable expression of this gene before the middle of somitogenesis. (a, b) embryo at the middle of somitogenesis in lateral view (a) and in dorsal view (b). (c - e) embryo at 24 hpf in lateral view (c), dorsal view (d) and frontal view dorsal to the right (e). (f - h) embryo at 36 hpf in lateral views (f, g) and frontal view dorsal to the left (h). (i - m) embryo at 48 hpf in lateral view (i - k), dorsal view (l) and frontal view dorsal to the left (m).

Supplementary Figure 3 - 64 : Expression of  LRH1 (NR5A2)
There is no detectable expression of this gene before the middle of somitogenesis. (a - e) embryo at the middle of somitogenesis in lateral view (a, b) and in dorsal views of the trunk (c, d) and of the tail region (e). (f - j) embryo at 24 hpf in lateral views (f, g), dorsal views (h, i) and frontal view, dorsal to the left (j). (k - t) embryo at 36 hpf in lateral views (k - q, s, t) and in dorsal view (r). (u - ac) embryo at 48 hpf in lateral views (u - x) dorsal views (y, z) and frontal views dorsal to the top (aa, ac) and dorsal to the left (ab).

Supplementary Figure 3 - 65 : Expression of  FF1C (NR5A5)
There is no detectable expression of this gene during embryonic development. (a, b) embryo at the 5 dpf in lateral view (a) and in dorsal view (b).

Supplementary Figure 3 - 66 : Expression of  GCNF-A (NR6A1-A)
Expression of this gene is not spatially restricted during gastrula (a) and early somitogenesis (b) stages. (c, d) embryo at the middle of somitogenesis in lateral view (c) and dorsal view of the hindbrain region (d). (e - g) embryo at 24 hpf in lateral view (e) and in dorsal views (f, g). (h) embryo at 36 hpf in lateral view. There is no detectable expession during embryonic development after the 24 hpf stage.

Supplementary Figure 3 - 67 : Expression of  GCNF-B (NR6A1-B)
There is no detectable expression of this gene at gastrula stage (a). (b, c) embryo at early somitogenesis in lateral view (b) and in dorsal view (c). (d, h) embryo at the middle of somitogenesis in lateral view (d), dorsal views (e - g) and in a transverse optical cross section of the tail (dorsal to the right, h). (i - n) embryo at 24 hpf in lateral views (i - l) and in dorsal view (m, n). (o - u) embryo at 36 hpf in lateral views (o - q), oblique view (r), frontal view dorsal to the right (s) and dorsal views (t, u). (v - x) embryo at 48 hpf in lateral view (v, w) and dorsal view (x).

Supplementary Figure 3 - 68 : Expression of  DAX (NR0B1)
Expression of this gene is not spatially restricted during embryonic larval stages. (a) embryo at gastrula stage,  (b) at early somitogenesis, (c) at middle of somitogenesis, (d) at 24 hpf, (e) at 36 hpf, (f) at 48 hpf . (g) embryo at 5 dpf  showing expression in liver, intestinal bulb and swim bladder.

Supplementary Figure 3 - 69 : Expression of  SHP-A (NR0B2-A)
(a) embryo at gastrula stage in lateral view. (b) embryo at early somitogenesis in lateral view. (c) embryo at 24 hpf in lateral view. There is no detectable expression during embryonic development after the 24 hpf stage.

Supplementary Figure 3 - 70 : Expression of  SHP-B (NR0B2-B)
There is no detectable expression of this gene during embryonic and larval stages. (a) embryo at gastrula stage,  (b) at early somitogenesis, (c) at middle of somitogenesis, (d) at 24 hpf, (e) at 36 hpf, (f) at 48 hpf and (g) at 5 dpf.

Supplementary Figure 3 - 71 : Expression of  BRG1 
Expression of this gene is not spatially restricted during embryonic and larval stages except for the lateral line at 24 and 36 hpf. (a) embryo at gastrula stage,  (b) at early somitogenesis, (c) at middle of somitogenesis, (d) at 24 hpf, (e) at 36 hpf, (f) at 48 hpf and (g) at 5 dpf.

Supplementary Figure 3 - 72 : Expression of  BAF53a 
Expression of this gene is not spatially restricted during embryonic and larval stages. (a) embryo at gastrula stage,  (b) at early somitogenesis, (c) at middle of somitogenesis, (d) at 24 hpf, (e) at 36 hpf, (f) at 48 hpf and (g) at 5 dpf.

Supplementary Figure 3 - 73 : Expression of  BAF60a 
Expression of this gene is not spatially restricted during embryonic and larval stages. (a) embryo at gastrula stage,  (b) at early somitogenesis, (c) at middle of somitogenesis, (d) at 24 hpf, (e) at 36 hpf, (f) at 48 hpf and (g) at 5 dpf.

Supplementary Figure 3 - 74 : Expression of  PCAF 
There is no detectable expression of this gene during embryonic and larval stages. (a) embryo at gastrula stage,  (b) at early somitogenesis, (c) at middle of somitogenesis, (d) at 24 hpf, (e) at 36 hpf, (f) at 48 hpf and (g) at 5 dpf.

Supplementary Figure 3 - 75 : Expression of  P300-A 
Expression of this gene is not spatially restricted during embryonic and larval stages. (a) embryo at gastrula stage,  (b) at early somitogenesis, (c) at middle of somitogenesis, (d) at 24 hpf, (e) at 36 hpf, (f) at 48 hpf and (g) at 5 dpf.

Supplementary Figure 3 - 76 : Expression of  P300-B 
Expression of this gene is not spatially restricted during embryonic stages. (a) embryo at gastrula stage,  (b) at early somitogenesis, (c) at middle of somitogenesis, (d) at 24 hpf, (e) at 36 hpf, (f) at 48 hpf. (g) embryo at 5 dpf in lateral view showing expression in intestinal bulb and swim bladder.

Supplementary Figure 3 - 77 : Expression of  CBP-A 
Expression of this gene is not spatially restricted during embryonic and larval stages. (a) embryo at gastrula stage,  (b) at early somitogenesis, (c) at middle of somitogenesis, (d) at 24 hpf, (e) at 36 hpf, (f) at 48 hpf and (g) at 5 dpf.

Supplementary Figure 3 - 78 : Expression of  CBP-B 
This gene is not spatially restricted during embryonic stages. (a) embryo at gastrula stage,  (b) at early somitogenesis, (c) at middle of somitogenesis, (d) at 24 hpf, (e) at 36 hpf, (f) at 48 hpf. (g) embryo at 5 dpf. showing expression in endodermal derivatives, liver, intestine and swim bladder

Supplementary Figure 3 - 79 : Expression of  CITED3 
(a,b) embryo at gastrula stage in lateral (a) and dorsal view (b). (c - f) embryo at early somitogenesis in lateral view (c) and in dorsal view (d - e) from anterior to posterior. (g - l) embryo at the middle of somitogenesis in lateral views (g, h) and in dorsal views (i - l) from anterior to posterior. (m - p) embyo at 24 hpf in lateral views (m, n) and in dorsal views (o, p). (q - v) embryo at 48 hpf in lateral views (q, r, t, u) and in dorsal views (s, v).

Supplementary Figure 3 - 80 : Expression of  SRC1 
Expression of this gene is not spatially restricted during embryonic and larval stages. (a) embryo at gastrula stage,  (b) at early somitogenesis, (c) at middle of somitogenesis, (d) at 24 hpf, (e) at 36 hpf, (f) at 48 hpf and (g) at 5 dpf.

Supplementary Figure 3 - 81 : Expression of  SRC2 
Expression of this gene is not spatially restricted during embryonic and larval stages. (a) embryo at gastrula stage,  (b) at early somitogenesis, (c) at middle of somitogenesis, (d) at 24 hpf, (e) at 36 hpf, (f) at 48 hpf and (g) at 5 dpf.

Supplementary Figure 3 - 82 : Expression of  SRC3 
(a, b) embryo at gastrula stage in lateral views. (c, d) embryo at ealy somitogenesis in lateral view (c) and dorsal view of the tail bud region (d). (e, f) embryo at the middle of somitogenesis in lateral view (e) and in dorsal view (f). (g, h) embryo at 24 hpf in lateral view (g) and in dorsal view (h). (i) embryo at 36 hpf in lateral view. (j) embryo at 48 hpf in lateral view.

Supplementary Figure 3 - 83 : Expression of  CARM1 
Expression of this gene is not spatially restricted during embryonic and larval stages. (a) embryo at gastrula stage,  (b) at early somitogenesis, (c) at middle of somitogenesis, (d) at 24 hpf, (e) at 36 hpf, (f) at 48 hpf and (g) at 5 dpf.

Supplementary Figure 3 - 84 : Expression of  NCOA6 
Expression of this gene is not spatially restricted during embryonic and larval stages. (a) embryo at gastrula stage,  (b) at early somitogenesis, (c) at middle of somitogenesis, (d) at 24 hpf, (e) at 36 hpf, (f) at 48 hpf and (g) at 5 dpf.

Supplementary Figure 3 - 85 : Expression of  RIP140-A 
(a - c) embryo at the onset of gastrulation (shield stage) in animal pole view (a), lateral view (b) and dorsal view. (d - g) embryo at gastrula stage (75% epiboly) in lateral view (d) and dorsal views (e - g). (h - n) embryo at early somitogenesis in lateral view (h) and dorsal views (i-n). (o - s) embryo at the middle of somitogensis in lateral view (o, p) and in dorsal view (q - s). (t - v) embryo at 24 hpf in lateral view (t) and in dorsal views (u, v). (w, y) embryo at 36 hpf in lateral views (w, y) and in dorsal view (x). (z) embryo at 48 hpf in lateral view.

Supplementary Figure 3 - 86 : Expression of  RIP140-B 
There is no detectable expression at gastrula stage (a). (b -f) embryo at early somitogenesis in dorsal views (b - e). (f) transverse optical cross-section of the trunk region (dorsal to the right). (g - j) embryo at the middle of somitogenesis in lateral views (g, h) and in dorsal views (i, j). (k - r) embryo at 24 hpf in lateral views (k - m) and in dorsal views (n - r). (s - u) embryo at 36 hpf in lateral view (s) and dorsal views (t, u). (v - x) embryo at 48 hpf in lateral view (v) and in dorsal views (w, x). (y, z) embryo at 5 dpf in dorsal views.

Supplementary Figure 3 - 87 : Expression of  TRAP220 
There is no detectable expression of this gene during embryonic and larval stages
(a) embryo at gastrula stage,  (b) at early somitogenesis, (c) at middle of somitogenesis, (d) at 24 hpf, (e) at 36 hpf, (f) at 48 hpf and (g) at 5 dpf.

Supplementary Figure 3 - 88 : Expression of  TRIP13 
Expression of this gene is not spatially restricted during embryonic and larval stages. (a) embryo at gastrula stage,  (b) at early somitogenesis, (c) at middle of somitogenesis, (d) at 24 hpf, (e) at 36 hpf, (f) at 48 hpf and (g) at 5 dpf.

Supplementary Figure 3 - 89 : Expression of  ARA54 
There is no detectable expression of this gene during embryonic and larval stages. (a) embryo at gastrula stage,  (b) at early somitogenesis, (c) at middle of somitogenesis, (d) at 24 hpf, (e) at 36 hpf, (f) at 48 hpf and (g) at 5 dpf.

Supplementary Figure 3 - 90 : Expression of  MYST-HAT2 
There is no detectable expression of this gene during embryonic and larval stages. (a) embryo at gastrula stage,  (b) at early somitogenesis, (c) at middle of somitogenesis, (d) at 24 hpf, (e) at 36 hpf, (f) at 48 hpf and (g) at 5 dpf.

Supplementary Figure 3 - 91 : Expression of  TIF1α
There is no detectable expression of this gene during embryonic and larval stages. (a) embryo at gastrula stage,  (b) at early somitogenesis, (c) at middle of somitogenesis, (d) at 24 hpf, (e) at 36 hpf, (f) at 48 hpf and (g) at 5 dpf.

Supplementary Figure 3 - 92 : Expression of  TIF1γ 
(a) embryo at gastrula stage in lateral view. (b) embryo at early somitogenesis in dorsal view of the posterior region. (c, d) embryo at the middle of somitogenesis in lateral view (c) and in dorsal view (d). (e, f) embryo at 24 hpf in lateral views. (g) embryo at 36 hpf in lateral view. (h) embryo at 48 hpf in lateral view.

Supplementary Figure 3 - 93 : Expression of  NCOA4 
Expression of this gene is not spatially restricted during embryonic and larval stages. (a) embryo at gastrula stage,  (b) at early somitogenesis, (c) at middle of somitogenesis, (d) at 24 hpf, (e) at 36 hpf, (f) at 48 hpf and (g) at 5 dpf.

Supplementary Figure 3 - 94 : Expression of  PGC1
(a, b) embryo at gastrula stage in lateral view (a) and dorsal view (b). (c, d) embryo at early somitogenesis in lateral view (c) and in dorsal view (d). (e - g) embryo at middle of somitogenesis in lateral view (e, f) and dorsal views (g). (h - k) embryo at 24 hpf in lateral views (h,i) and dorsal views (j, k). (l - r) embryo at 36 hpf in lateral view (l, m), oblique view (n, q), dorsal views (o, p) and frontal view (r). (s - u) embryo at 48 hpf in lateral views (s, t) and dorsal view (u). (v, w) embryo at 5 dpf in lateral view (v) and dorsal view (w).

Supplementary Figure 3 - 95 : Expression of  TRIP7 
There is no detectable expression of this gene before the middle of somitogenesis. (a, d) embryo at the middle of somitogenesis in lateral views (a, c) and in dorsal views (b,d). (e) embryo at 24 hpf. (f, g) embryo at 36 hpf in lateral view (f) and dorsal view (g). (h, i) embryo at 48 hpf in lateral view (h) and in dorsal view (i).

Supplementary Figure 3 - 96 : Expression of  HDAC1 
Expression of this gene is not spatially restricted during embryonic and larval stages. (a) embryo at gastrula stage,  (b) at early somitogenesis, (c) at middle of somitogenesis, (d) at 24 hpf, (e) at 36 hpf, (f) at 48 hpf and (g) at 5 dpf.

Supplementary Figure 3 - 97 : Expression of  HDAC3 
Expression of this gene is not spatially restricted during embryonic and larval stages. (a) embryo at gastrula stage,  (b) at early somitogenesis, (c) at middle of somitogenesis, (d) at 24 hpf, (e) at 36 hpf, (f) at 48 hpf and (g) at 5 dpf.

Supplementary Figure 3 - 98 : Expression of  HDAC4 
There is no detectable expression for this gene before the middle of somitogenesis. (a, b) embryo at the middle of somitogenesis in lateral view (a) and dorsal view (b). (c, d) embryo at 24 hpf in lateral view (c) and dorsal view (d). (e - h) embryo at 36 hpf in lateral views (e, f) and dorsal views (g, h). (i, j) embryo at 48 hpf in lateral view (i) and dorsal view (j).

Supplementary Figure 3 - 99 : Expression of  SMRT
(a) embryo at the gastrula stage in lateral view. (b - f) embryo at early somitogenesis in lateral view (c) and in dorsal views (b, d - f). (g, h) embryo at the middle of somitogenesis in lateral view (g) and in dorsal view (h). (i - l) embryo at 24 hpf in lateral view (i, j), dorsal view (k) and oblique view (l). (m - o) embryo at 36 hpf in lateral view (m, n) and oblique view (o). (p, q) embryo at 48 hpf in lateral views. (r) embryo at 5 dpf in dorsal view.
 
Supplementary Figure 3 - 100 : Expression of  NCOR 
Expression of this gene is not spatially restricted during embryonic and larval stages. (a) embryo at gastrula stage,  (b) at early somitogenesis, (c) at middle of somitogenesis, (d) at 24 hpf, (e) at 36 hpf, (f) at 48 hpf and (g) at 5 dpf.

Supplementary Figure 3 - 101 : Expression of  Alien
Expression of this gene is not spatially restricted during embryonic and larval stages. (a) embryo at gastrula stage,  (b) at early somitogenesis, (c) at middle of somitogenesis, (d) at 24 hpf, (e) at 36 hpf, (f) at 48 hpf and (g) at 5 dpf.

Supplementary Figure 4 -  1 : expression of TRβ in the retina.
(a, b) lateral and (d) dorsal views of the head. (b) lateral view of the distal part of the eye. (c) parasagittal optical cross-section. (f - i) thin sections. TRβ is expressed in the photoreceptor (outer nuclear layer) cell layer as well as in few cells proximal to the proliferative zone for the ganglion and for the inner nuclear cell layer.

Supplementary Figure 4 -  2 : expression of RARα−A in the retina.
(a, b) lateral and (d) dorsal views of the head. (c) lateral view and (e - f) dorsal view of the eye. (g  - i) thin sections. RARα-A is expressed in the ganglion and in the inner nuclear layer but not in the outer nuclear layer. Expression is very weak in the proliferative zone and is stronger in the ganglion cell layer than in the inner nuclear layer.

Supplementary Figure 4 -  3 : expression of RARα−B in the retina.
(a, b) lateral and (d) dorsal views of the head. (c) lateral view and (e - f) dorsal view of the eye. (g  - i) thin sections. RARα-B is expressed in the ganglion and in the inner nuclear layer but not in the outer nuclear layer. Expression is very weak in the proliferative zone and is stronger in the ganglion cell layer than in the inner nuclear layer.

Supplementary Figure 4 -  4 : expression of Reverbα in the retina.
(a, b) lateral and (d) dorsal views of the head. (c) lateral and (e) dorsal views of the eye. (f  - i) thin sections. Reverbα is expressed in the ganglion and in the inner nuclear layer but not in the outer nuclear layer. Expression is very weak in the proliferative zone.

Supplementary Figure 4 -  5 : expression of Reverbβ−A in the retina.
(a, b) lateral and (d) dorsal views of the head. (c) lateral view and (e - f) dorsal view of the eye. (g  - i) thin sections. Reverbβ-A is expressed in the ganglion and in the inner nuclear layer but not in the outer nuclear layer and in the proliferative zone.

Supplementary Figure 4 -  6 : expression of Reverbβ−B in the retina.
(a, b) lateral and (d) dorsal views of the head. (c) lateral view and (e - f) dorsal view of the eye. (g  - i) thin sections. Reverbα is expressed in the ganglion and in the inner nuclear layer but not in the outer nuclear layer. Expression is very weak in the proliferative zone.

Supplementary Figure 4 -  7 : expression of Reverbγ−B in the retina.
(a) lateral and (c) dorsal views of the head. (b) lateral view and (d) dorsal view of the eye. (e  - i) thin sections. Reverbα is expressed in the inner nuclear layer and in few cells of the distal part of the ganglion cell layer. Expression is very weak in the proliferative zone.

Supplementary Figure 4 -  8 : expression of RORα−A in the retina.
(a, b) lateral and (d) dorsal views of the head. (c) lateral view and (e - f) dorsal view of the eye. (f  - i) thin sections. RORα-A is expressed in the ganglion and in the inner nuclear layer but not in the outer nuclear layer or in the proliferative zone.

Supplementary Figure 4 -  9 : expression of RORα−B in the retina.
(a, b) lateral and (d) dorsal views of the head. (c) lateral view and (e - f) dorsal view of the eye. (g  - i) thin sections. RORα-A is expressed in the ganglion and in the distal part of the inner nuclear layer but not in the outer nuclear layer or in the proliferative zone.

Supplementary Figure 4 -  10 : expression of RORβ in the retina.
(a, b) lateral and (d) dorsal views of the head. (c) lateral view and (e - f) dorsal view of the eye. (g  - i) thin sections. RORβ is expressed in the proliferative zone as well as in the proximal and in the distal part of the inner cell layer.

Supplementary Figure 4 -  11 : expression of RORγ−A in the retina.
(a) lateral and (c) dorsal views of the head. (b) lateral view and (d) dorsal view of the eye. (e  - i) thin sections. RORγ−A is expressed in the proliferative zone as well as in the lateral part of the three layers of the retina.

Supplementary Figure 4 -  12 : expression of RORγ−B in the retina.
(a) lateral and (d) dorsal views of the head. (b, c) lateral view and (d, e) dorsal view of the eye. (g  - i) thin sections. RORγ−B is expressed in the inner nuclear layer. A weak expression is also observed in the central part of the outer nuclear layer. There is no expression in the proliferative zone.

Supplementary Figure 4 -  13 : expression of RXRα−A in the retina.
(a) lateral and (d) dorsal views of the head. (b, c) lateral view and (d, e) dorsal view of the eye. (g  - i) thin sections. RXRα−A is expressed in a subpopulation of cells in the inner nuclear layer as well as in the outer nuclear layer near the marginal zone. 

Supplementary Figure 4 -  14 : expression of RXRα−B in the retina.
(a) lateral and (c) dorsal views of the head. (b) lateral view and (d, e) dorsal view of the eye. (f  - i) thin sections. RXRα−B is expressed in the ganglion cell layer and in the inner nuclear layer with less expression in the ventral part of these cell layers. There is no expression in the outer nuclear layer and in the proliverative zone. 

Supplementary Figure 4 -  15 : expression of RXRγ in the retina.
(a) lateral and (d) dorsal views of the head. (b, c) lateral view and (e, f) dorsal view of the eye. (g  - i) thin sections. RXRγ is expressed in a subpopulation of cells in the the inner nuclear layer as well as in the marginal zone of the outer nuclear layer. 

Supplementary Figure 4 -  16 : expression of TLL in the retina.
(a, b) lateral and (d) dorsal views of the head. (c) lateral view and (e, f) dorsal view of the eye. (g  - i) thin sections. TLL is expressed in the proliferative zone of the retina. 

Supplementary Figure 4 -  17 : expression of PNR in the retina.
(a, b) lateral and (d) dorsal views of the head. (c) lateral view and (e) dorsal view of the eye. (f  - i) thin sections. PNR is expressed in the outer nuclear layer with more labelled cells near the marginal zone. 

Supplementary Figure 4 -  18 : expression of COUPTFα-B in the retina.
(a, b) lateral and (d) dorsal views of the head. (c) lateral view and (e) dorsal view of the eye. (f  - i) thin sections. COUPTFα-B is expressed in the dorsal part of the ganglion and of the inner nuclear layer. 

Supplementary Figure 4 -  19 : expression of COUPTFβ in the retina.
(a, b) lateral and (d) dorsal views of the head. (b, c) lateral view and (e) dorsal view of the eye. (f  - i) thin sections. COUPTFβ is expressed in the outer nuclear layer. 

Supplementary Figure 4 -  20 : expression of COUPTFγ in the retina.
(a) lateral and (d) dorsal views of the head. (c) lateral view and (e, f) dorsal view of the eye. (g  - i) thin sections. COUPTFγ is expressed mainly in the ventral part of the proliferative zone. A weak expression is also observed in the dorsal part of the porliferative zone as well as in some cells in the dorsal part of the inner nuclear layer. 

Supplementary Figure 4 -  21 : expression of EAR2-B in the retina.
(a) lateral and (c) dorsal views of the head. (b) lateral view and (d) dorsal view of the eye. (e  - i) thin sections. EAR2-B is expressed in the outer nuclear layer except near the marginal zoneL. A weak expression is also observed in the peripheral cells of the lens and in the dorsal part of the inner nuclear layer. 

Supplementary Figure 4 -  22 : expression of ERRα in the retina.
(a) lateral views of the head. (b) dorsal view and (c) lateral view of the eye. (d, f) thin sections. ERRα is weakly expressed in the three layers of the retina with a stronger intensity in the dorsal part. 

Supplementary Figure 4 -  23 : expression of ERRβγ in the retina.
(a) lateral and (c) dorsal views of the head. (b) lateral view and (d) dorsal view of the eye. (e  - i) thin sections. ERRβγ is expressed in the three layers of the retina. 

Supplementary Figure 4 -  24 : expression of ERRγ in the retina.
(a, b ) lateral and (c) dorsal views of the head. (c) lateral view and (e, f) dorsal view of the eye. (g  - i) thin sections. ERRγ is expressed in in a subpopulation of cells in the inner nuclear layer and in a subpopulation of cell in the ventral part of the ganglion cell layer. 

Supplementary Figure 4 -  25 : expression of ERRδ in the retina.
(a) lateral view of the head. (b) lateral view and (c) dorsal view of the eye. (d  - f) thin sections. ERRδ is expressed in the proximal part and in the marginal part of the inner nuclear layer. 

Supplementary Figure 4 -  26 : expression of NURR1 in the retina.
(a, b) lateral and (c) dorsal views of the head. (c) lateral view and (e, f) dorsal view of the eye. (g  - i) thin sections. NURR1 is expressed in a subpopulation of cells of the inner nuclear layer.
