# Supplementary material for: Unexpected Novel Relational Links Uncovered by Extensive Developmental Profiling of Nuclear Receptor Expression
Source: PLoS Genet. 2007 Nov 9;3(11):e188. doi: 10.1371/journal.pgen.0030188 (PMC2065881; doi:10.1371/journal.pgen.0030188)
Supplement: Supplementary file 10 [file pgen.0030188.st003.doc]

Bertrand et al.,

Supplementary Table 3

**Not detected	Ubiquitously expressed	Restricted expression**

**Gastrula**	TRalpha-A	TRalpha-B	RARalpha-A

TRbeta	PPARbeta-B	RARalpha-B

RARgamma-B	PPARgamma	RARgamma-A

PPARalpha-A	Rev-erbgamma-A	HNF4alpha

PPARalpha-B	VDR-A	HNF4beta

PPARbeta-A	RXRbeta-A	EAR2-B

Rev-erbalpha	RXRbeta-B	ERRalpha

Rev-erbbeta-A	TR2	SHP-A

Rev-erbbeta-B	EAR2-A

Rev-erbgamma-B	ERbeta-B

RORalpha-A	ERRbeta/gamma

RORalpha-B	PR

RORbeta	GR

RORgamma-A	NGFI-B

RORgamma-B	GCNF-A

LXRalpha	DAX

FXRalpha

FXRbeta

VDR-B

PXR

HNF4gamma

RXRalpha-A

RXRalpha-B

RXRgamma

TR4

PNR

TLL

COUP-TFalpha-A

COUP-TFalpha-B

COUPTFbeta

COUP-TFgamma

ERalpha

ERbeta-A

ERRbeta

ERRgamma

ERRdelta

MR

AR

NURR1

NOR1

LRH1

SF1-A

FF1C

SF1-B

GCNF-B

SHP-B

**ES**	TRalpha-A	TRalpha-B	RARalpha-A

TRbeta	PPARbeta-B	RARalpha-B

PPARalpha-A	PPARgamma	RARgamma-A

PPARalpha-B	Rev-erbgamma-A	RARgamma-B

PPARbeta-A	VDR-A	RORgamma-A

Rev-erbalpha	RXRbeta-A	HNF4alpha

Rev-erbbeta-A	RXRbeta-B	HNF4beta

Rev-erbbeta-B	TR2	RXRgamma

Rev-erbgamma-B	EAR2-A	TLL

RORalpha-A	ERbeta-B	COUP-TFalpha-A

RORalpha-B	ERRbeta/gamma	COUP-TFalpha-B

RORbeta	GR	COUP-TFbeta

RORgamma-B	PR	COUP-TFgamma

LXRalpha	NGFI-B	EAR2-B

FXRalpha	GCNF-A	ERRalpha

FXRbeta	DAX	ERRgamma

VDR-B	SHP-A

HNF4gamma

RXRalpha-A

RXRalpha-B

TR4

PNR

ERalpha

ERbeta-A

ERRbeta

ERRdelta

MR

AR

NURR1

NOR1

LRH1

SF1-A

FF1C

SF1-B

GCNF-B

SHP-B

**MS**	TRalpha-A	TRalpha-B	RARalpha-A

TRbeta	PPARbeta-B	RARalpha-B

PPARalpha-A	PPARgamma	RARgamma-A

PPARalpha-B	Rev-erbgamma-A	RARgamma-B

PPARbeta-A	VDR-A	RORalpha-A

Rev-erbalpha	RXRbeta-A	RORbeta

Rev-erbbeta-A	RXRbeta-B	RORgamma-A

Rev-erbbeta-B	TR2	RORgamma-B

Rev-erbgamma-B	EAR2-A	VDR-B

RORalpha-B	ERbeta-B	HNF4alpha

LXRalpha	ERRbeta/gamma	HNF4beta

FXRalpha	GR	RXRalpha-B

FXRbeta	PR	RXRgamma

PXR	NGFI-B	PNR

HNF4gamma	GCNF-A	TLL

RXRalpha-A	DAX	COUP-TFalpha-A

TR4	COUP-TFalpha-B
ERalpha	COUP-TFbeta

ERbeta-A	COUP-TFgamma

MR	EAR2-B

AR	ERRalpha

NURR1	ERRbeta

NOR1	ERRgamma

SF1-A	ERRdelta

FF1C	LRH1

SHP-B	SF1-B

GCNF-B

SHP-A

**24 hpf**	PPARalpha-A	TRalpha-A	TRbeta	PPARalpha-B	TRalpha-B	RARalpha-A

Rev-erbbeta-A	PPARbeta-A	RARalpha-B

Rev-erbgamma-B	PPARbeta-B	RARgamma-A

RORalpha-B	PPARgamma	RARgamma-B

LXRalpha	Rev-erbgamma-A	Rev-erbalpha

FXRalpha	VDR-A	Rev-erbbeta-B

HNF4gamma	RXRbeta-A	RORalpha-A

RXRalpha-A	RXRbeta-B	RORbeta

ERalpha	TR2	RORgamma-A

ERbeta-A	TR4	RORgamma-B

MR	EAR2-A	FXRbeta

AR	ERbeta-B	VDR-B

FF1C	ERRbeta/gamma	PXR

SHP-B	GR	HNF4alpha

PR	HNF4beta

NGFI-B	RXRalpha-B

GCNF-A	RXRgamma

DAX	PNR

TLL

COUP-TFalpha-A

COUP-TFalpha-B

COUP-TFbeta

COUP-TFgamma

EAR2-B

ERRalpha

ERRbeta

ERRgamma

ERRdelta

NURR1

NOR1

LRH1

SF1-A

SF1-B

GCNF-B

SHP-A

**36 hpf**	PPARalpha-A	TRalpha-A	TRbeta

PPARalpha-B	TRalpha-B	RARalpha-A

Rev-erbbeta-A	PPARbeta-A	RARalpha-B

Rev-erbbeta-B	Rev-erbgamma-A	RARgamma-A

LXRalpha	VDR-A	RARgamma-B

HNF4beta	RXRbeta-A	PPARbeta-B

HNF4gamma	RXRbeta-B	PPARgamma

ERalpha	TR2	Rev-erbalpha

MR	TR4	Rev-erbgamma-B

AR	EAR2-A	RORalpha-A

FF1C	ERbeta-B	RORalpha-B

GCNF-A	ERRbeta/gamma	RORbeta

SHP-A	GR	RORgamma-A

SHP-B	PR	RORgamma-B

NGFI-B	FXRalpha

DAX	FXRbeta

VDR-B

PXR

HNF4alpha

RXRalpha-A

RXRalpha-B

RXRgamma

PNR

TLL

COUP-TFalpha-A

COUP-TFalpha-B

COUP-TFbeta

COUP-TFgamma

EAR2-B

ERbeta-A

ERRalpha

ERRbeta

ERRgamma

ERRdelta

NURR1

NOR1

LRH1

SF1-A

SF1-B

GCNF-B

**48 hpf**	PPARalpha-A	TRalpha-B	TRalpha-A

PPARalpha-B	PPARbeta-A	TRbeta

LXRalpha	PPARbeta-B	RARalpha-A

HNF4beta	PPARgamma	RARalpha-B

HNF4gamma	Rev-erbgamma-A	RARgamma-A

ERalpha	VDR-A	RARgamma-B

MR	RXRbeta-A	Rev-erbalpha

AR	RXRbeta-B	Rev-erbbeta-A

FF1C	TR2	Rev-erbbeta-B

GCNF-A	TR4	Rev-erbgamma-B

SHP-A	EAR2-A	RORalpha-A

SHP-B	ERbeta-B	RORalpha-B

ERRbeta/gamma	RORbeta

GR	RORgamma-A

PR	RORgamma-B

DAX	FXRalpha

FXRbeta

VDR-B

PXR

HNF4alpha

RXRalpha-A

RXRalpha-B

RXRgamma

PNR

TLL

COUP-TFalpha-A

COUP-TFalpha-B

COUP-TFbeta

COUP-TFgamma

EAR2-B

ERbeta-A

ERRalpha

ERRbeta

ERRgamma

ERRdelta

NGFI-B

NURR1

NOR1

LRH1

SF1-A

SF1-B

GCNF-B
